# FLP‐Catalyzed Transfer Hydrogenation of Silyl Enol Ethers

**DOI:** 10.1002/anie.201808800

**Published:** 2018-08-24

**Authors:** Imtiaz Khan, Benjamin G. Reed‐Berendt, Rebecca L. Melen, Louis C. Morrill

**Affiliations:** ^1^ School of Chemistry Cardiff University Main Building Park Place Cardiff CF10 3AT UK

**Keywords:** dihydrogen surrogates, frustrated Lewis pairs, metal free catalysis, silyl enol ethers, transfer hydrogenation

## Abstract

Herein we report the first catalytic transfer hydrogenation of silyl enol ethers. This metal free approach employs tris(pentafluorophenyl)borane and 2,2,6,6‐tetramethylpiperidine (TMP) as a commercially available FLP catalyst system and naturally occurring γ‐terpinene as a dihydrogen surrogate. A variety of silyl enol ethers undergo efficient hydrogenation, with the reduced products isolated in excellent yields (29 examples, 82 % average yield).

Over the past decade, the development of Frustrated Lewis Pair (FLP) chemistry has received considerable attention.^1^ Representing an area of particular interest, FLPs can be employed as catalysts in metal free hydrogenation processes.^2^ Dihydrogen is typically employed as the reductant in such processes, however, recent advances have shown that amines,^3^ cyclohexadienes,^4^ ammonia borane,^5^ and Hantzsch esters^6^ can be employed as dihydrogen surrogates in B(C_6_F_5_)_3_‐catalyzed transfer hydrogenation. Systems employing an additional Lewis base, rendering it an FLP‐type process, have been developed by Du and co‐workers for the enantioselective transfer hydrogenation of ketimines and quinoxalines.^7^ Alternatively, metal free transfer hydrogenation via dehydocoupling catalysis has been developed using borane and phosphenium salt catalysts.^8^

Silyl enol ethers have often served as a test bed for the development of novel FLP catalytic systems (Scheme 1 A).^9^, ^10^ In contrast to imines and *N*‐heterocycles, which can serve the role of the Lewis base within an FLP‐type system,^2^ the lower basicity of silyl enol ethers necessitates an additional Lewis base for dihydrogen activation and subsequent hydrogenation. In 2008, Erker and co‐workers reported the first FLP‐catalyzed hydrogenation of silyl enol ethers using a 1,8‐bis(diphenylphosphino)naphthalene/B(C_6_F_5_)_3_ FLP system.9a In 2012, Paradies and co‐workers employed a [2.2]‐paracyclophane derived bisphosphine as the Lewis base component of an FLP for silyl enol ether hydrogenation.9c Du and co‐workers subsequently developed methods for enantioselective FLP‐catalyzed hydrogenation of silyl enol ethers using in situ generated axially chiral boranes as Lewis acids in combination with *t*‐Bu_3_P as the Lewis base.9d,9e Despite these notable advances, there exists no reports to date that describe the transfer hydrogenation of silyl enol ethers via any metal or metal free catalytic process. Furthermore, all previous reports of FLP‐catalyzed hydrogenation of silyl enol ethers employ highly specialized FLP systems, such as those shown in Scheme 1 A, and require >1 bar dihydrogen pressure. Taking inspiration from the aforementioned works, and as part of our ongoing investigations into novel applications of FLPs in catalysis,^11^ herein we report the first catalytic transfer hydrogenation of silyl enol ethers, which uses a commercially available 2,2,6,6‐tetramethylpiperidine/B(C_6_F_5_)_3_ FLP catalyst system^12^ and naturally occurring γ‐terpinene4a as a dihydrogen surrogate (Scheme 1 B).

**Scheme 1:**
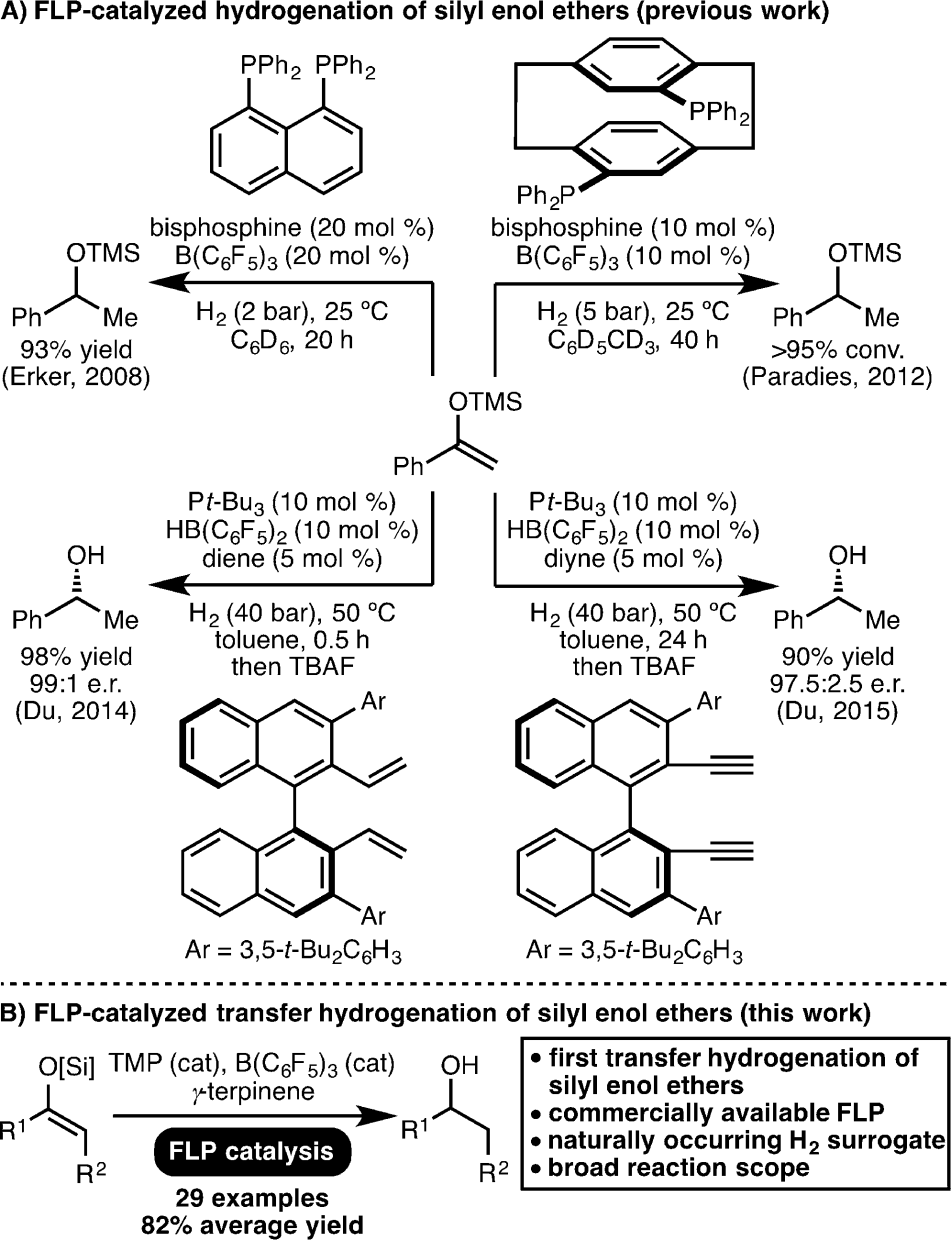
Previous work and outline of the FLP‐catalyzed transfer hydrogenation strategy.

To commence our studies, we selected silyl enol ether **1** as a model substrate (Table 1). After extensive optimization,^13^ it was found that a FLP system composed of B(C_6_F_5_)_3_ (10 mol %) and 2,2,6,6‐tetramethylpiperidine (TMP) (10 mol %) using γ‐terpinene **2 a** (1.3 equiv) as a dihydrogen surrogate in toluene ([**1**]=0.16 m) at 130 °C for 4 h, enabled the efficient transfer hydrogenation of **1**, giving silyl ether **3** in 96 % NMR yield (entry 1). No observable hydrogenation occurs in the absence of either Lewis acid or Lewis base, confirming FLP‐type catalysis is in operation (entries 2 and 3). Using the Childs’ method,^14^ Alcarazo and co‐workers have determined the relative Lewis acidity of the three boranes examined in this study, B(C_6_F_5_)_3_, B(2,4,6‐F_3_C_6_H_2_)_3_ and B(2,6‐F_2_C_6_H_3_)_3_, to be 100 %, 70 % and 56 % respectively.^15^ B(C_6_F_5_)_3_ proved to be optimal for this process, highlighting that a strong Lewis acid is required for efficient hydride abstraction from cyclohexa‐1,4‐dienes due to the formation of a high energy Wheland complex (entries 1, 4 and 5).^4^ Alternative Lewis bases, DABCO, 1,2,2,6,6‐pentamethylpiperidine (PMP) and *t*‐Bu_3_P, among others tested,^13^ gave lower conversions to **3** (entries 6–8). Using an alternative dihydrogen surrogate, namely 1,5‐dimethoxycyclohexa‐1,4‐diene **2 b**, resulted in only 23 % NMR yield of **3**, which is likely due to coordination of **2 b** to B(C_6_F_5_)_3_ (entry 9) via the ether oxygen atoms. Employing cyclohexa‐1,4‐diene **2 c** or ammonia borane **2 d** instead of **2 a** gave no observable product formation (entries 10 and 11). A range of solvents was examined,^13^ including benzene (entry 12), but none were advantageous over toluene. Increasing the concentration [**1**] to 0.32 m (entry 13), reducing the temperature to 60 °C (entry 14), shortening the reaction time to 2 h (entry 15) and reducing the catalyst loading to 5 mol % (entry 16) all resulted in decreased conversion to **3**, confirming that optimal reaction conditions (entry 1) had been determined.

**Table 1:** Optimization of the FLP‐catalyzed transfer hydrogenation.^[a]^ 
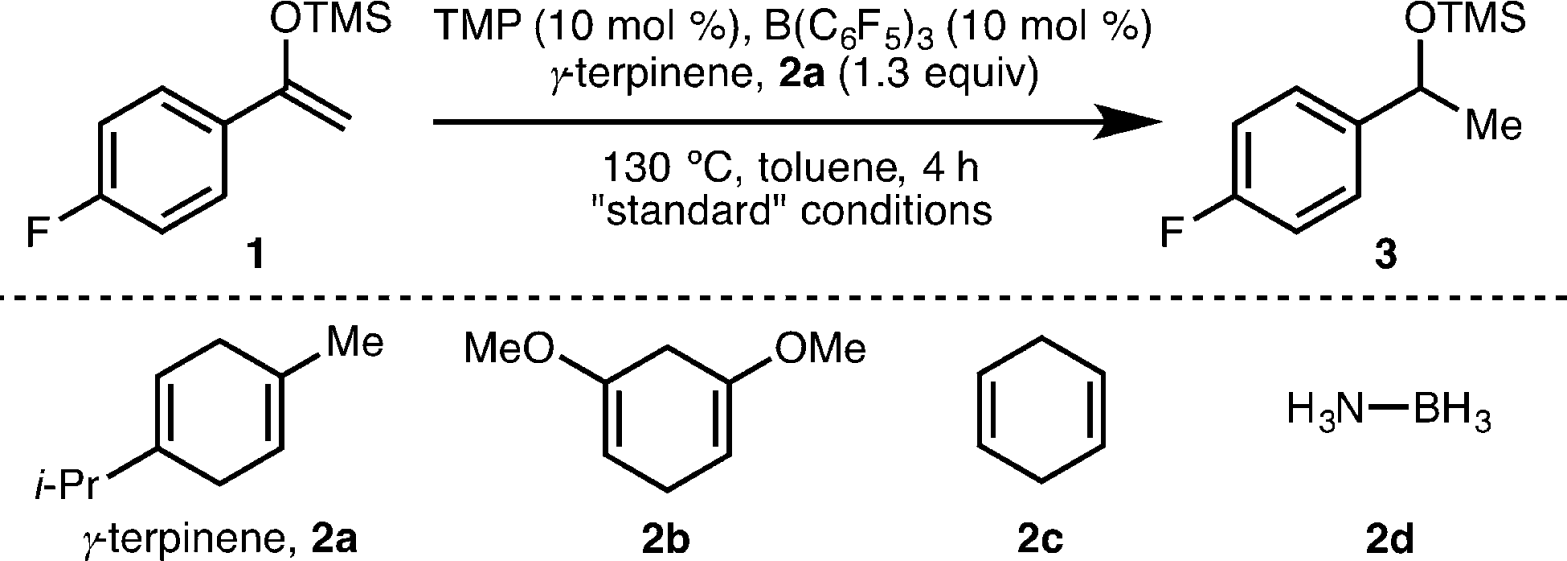

Entry	Variation from “standard” conditions	Yield^[b]^ [%]
**1**	**none**	**96**
2	no B(C_6_F_5_)_3_	<2
3	no TMP	<2
4	B(2,4,6‐F_3_C_6_H_2_)_3_ instead of B(C_6_F_5_)_3_	7
5	B(2,6‐F_2_C_6_H_3_)_3_ instead of B(C_6_F_5_)_3_	<2
6	DABCO instead of TMP	41
7	PMP instead of TMP	36
8	*t*‐Bu_3_P instead of TMP	43
9	**2 b** instead of **2 a**	23
10	**2 c** instead of **2 a**	<2
11	**2 d** instead of **2 a**	<2
12	benzene instead of toluene	46
13	[**1**]=0.32 m	87
14	60 °C	<2
15	2 h	47
16	5 mol % catalyst	23

[a] Reactions performed using 0.5 mmol of silyl enol ether **1** where [**1**]=0.16 m in toluene. [b] Determined by ^1^H NMR analysis of the crude reaction mixture with 1,3,5‐trimethylbenzene as the internal standard.

For the purposes of assessing the scope of this protocol, the standard reaction conditions (Table 1, entry 1) were used except the reaction time was extended to 16 h to ensure full conversion across a range of substrates (Scheme 2). Initially, the effect of varying the silicon group within the silyl enol ether on the FLP‐catalyzed transfer hydrogenation protocol was examined and it was found that TMS, TES, TBS, TIPS and TBDPS protected enol ethers were all tolerated. Due to their instability towards silica gel chromatographic purification, TMS, TES and TBS protected alcohols were deprotected in situ using TBAF (product **4**, 72–93 % yield), whereas TIPS and TBDPS protected alcohols **5** and **6** were isolated in 73 % and 74 % yields, respectively. In order to fully explore the substrate scope of this protocol, we initially produced a small library of TMS protected enol ethers. In some cases, when subjected to the optimized reaction conditions for transfer hydrogenation, significant quantities of silyl enol ether decomposition was observed, most likely due to the presence of a strong Lewis acid, B(C_6_F_5_)_3_, and the elevated reaction temperature (130 °C). This issue was addressed in such cases by simply employing the more robust TBS protected enol ether. Substitution of the aryl group within the silyl enol ether (R^1^ scope) was explored next, giving the corresponding secondary alcohols in excellent isolated yields (products **7**–**28**, 79 % average yield). Within the aryl unit, various 4‐, 3‐ and 2‐alkyl substitution was tolerated in addition to electron‐donating (4/3/2‐OMe) substituents. However, 4‐trifluoromethyl substitution resulted in only 8 % conversion to reduced product **17**. This result can be rationalized by the inductively electron‐withdrawing CF_3_ group reducing the basicity of the silyl enol ether, resulting in slow protonation by the protonated Lewis base (cf. Scheme 3 for proposed reaction mechanism). Halide substitution (4‐F, 4‐Cl, 4‐Br and 4‐I) within the starting materials was tolerated, incorporating an additional functional handle into the products for subsequent elaboration via cross‐coupling methods.^16^ Extended aromatic systems (1‐Np, 2‐Np and 9‐phenanthryl) and heteroaryls (2‐thiophenyl, 2‐benzothiophenyl and 2‐benzofuranyl) can also be present within the silyl enol ether substrate. Trisubstituted indanone‐ and cyclohexanone‐derived silyl enol ethers participated in the FLP‐catalyzed transfer hydrogenation protocol, giving secondary alcohols **28** and **29** in 84 % and 70 % isolated yields, respectively. Finally, a pinacolone‐derived TMS‐protected enol ether was fully converted to pinacolyl alcohol **30** using the optimized reaction conditions.

**Scheme 2:**
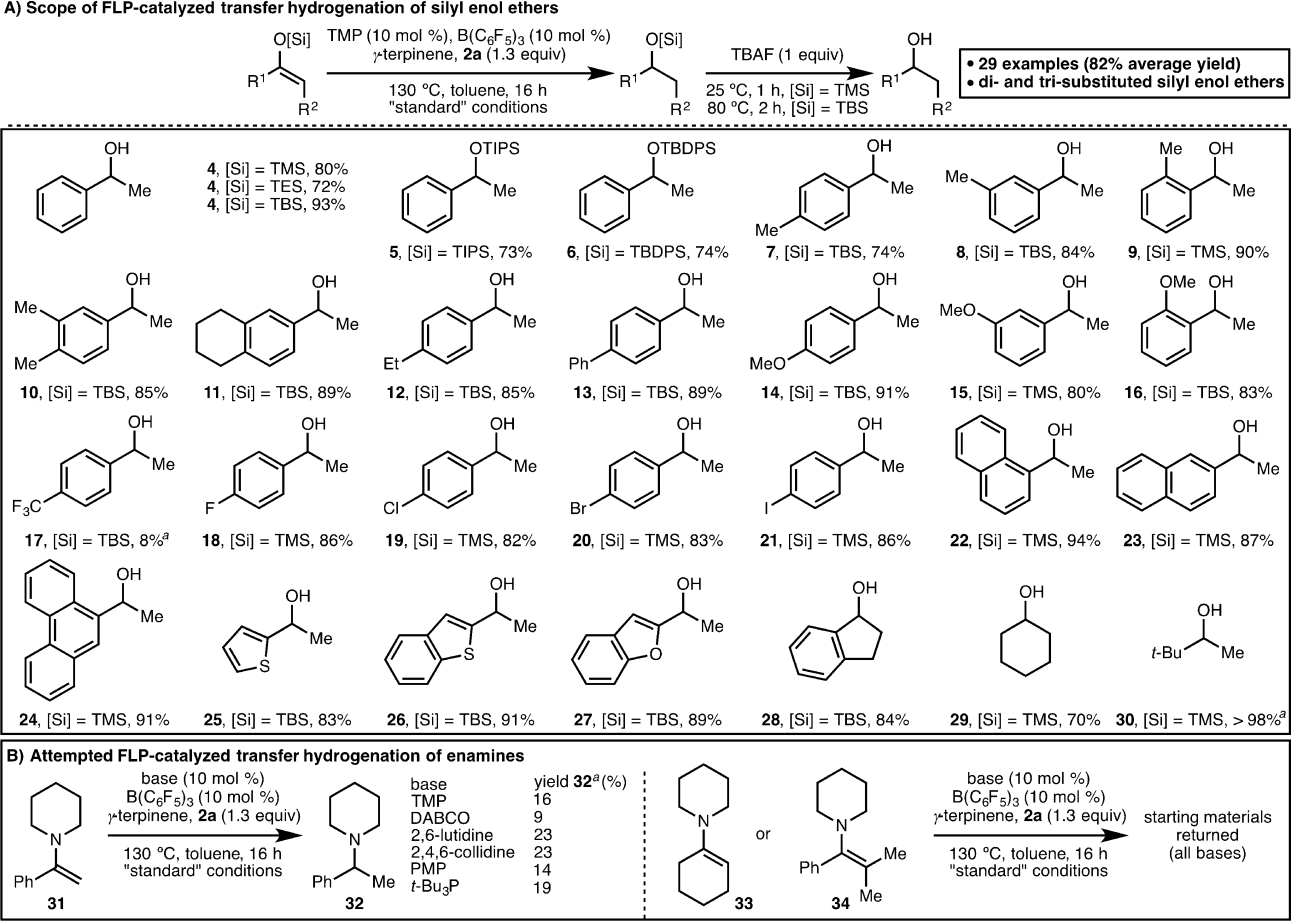
Scope of the FLP‐catalyzed transfer hydrogenation process. Reactions performed using 0.5 mmol of silyl enol ether starting material. All yields are isolated yields after chromatographic purification unless otherwise stated. [a] Determined by ^1^H NMR analysis of the crude reaction mixture with 1,3,5‐trimethylbenzene as the internal standard.

**Scheme 3:**
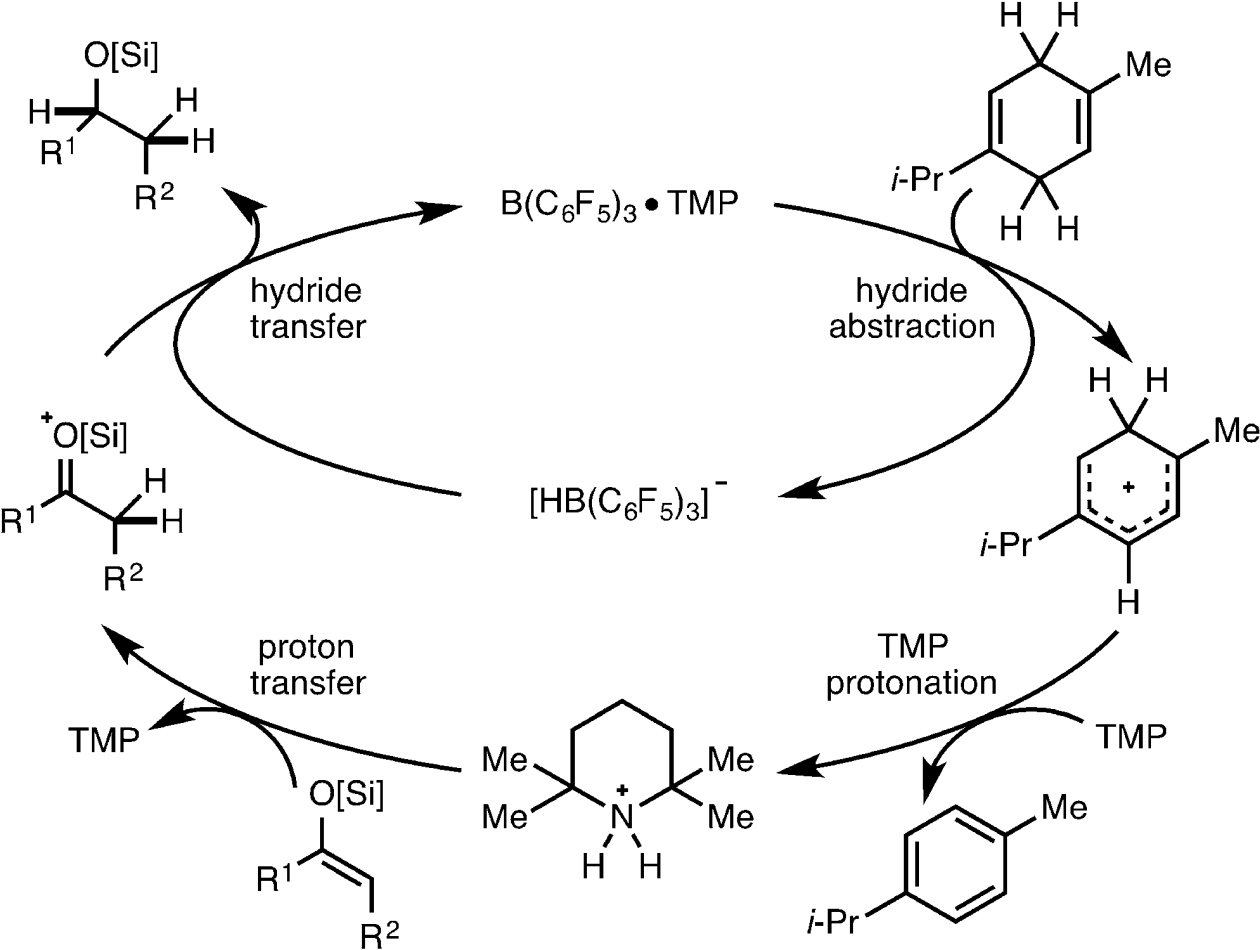
Proposed catalytic cycle for the FLP‐catalyzed transfer hydrogenation process.

Enamines are another class of enolate equivalent that have been studied as substrates for FLP‐catalyzed hydrogenation,^17^ but have never been employed in an FLP‐catalyzed transfer hydrogenation process. Encouraged by our success with silyl enol ethers, the previously optimized reaction conditions were employed using acetophenone‐derived disubstituted enamine **31** as substrate, giving 16 % conversion to tertiary amine **32**. Unfortunately, despite re‐optimization efforts, the maximum NMR yield of **32** observed was 23 % when 2,6‐lutidine or 2,4,6‐collidine was employed as the Lewis base. Similarly, suitable reaction conditions could not be identified to effect the FLP‐catalyzed transfer hydrogenation of tri‐ and tetrasubstituted enamines **33** and **34**, with starting materials returned in both cases. Previous reports of FLP‐catalyzed hydrogenation of enamines employ specialized boranes or borenium cations with lower relative Lewis acidity compared to B(C_6_F_5_)_3_.^17^ As such, the low conversions observed in this system are likely due to coordination of the nucleophilic enamine to B(C_6_F_5_)_3_.

The proposed mechanism for the FLP‐catalyzed transfer hydrogenation begins with initial hydride abstraction of γ‐terpinene by B(C_6_F_5_)_3_,^18^ giving a Wheland intermediate and [HB(C_6_F_5_)_3_]^−^ (Scheme 3).^4^ 2,2,6,6‐Tetramethylpiperidine (TMP) is then protonated by the Brønsted acidic Wheland intermediate,^19^ producing *p*‐cymene as a by product. This step is supported by evidence that no transfer hydrogenation occurs in the absence of TMP (cf. Table 1, Entry 3). Subsequent hydrogenation of the electron rich silyl enol ether occurs via proton transfer from [HTMP]^+^, to form a strongly electrophilic carbonyl moiety, followed by hydride transfer from [HB(C_6_F_5_)_3_]^−^ to complete the catalytic cycle.^20^

In conclusion, we have developed the first catalytic transfer hydrogenation of silyl enol ethers. This metal free approach employs a commercially available FLP system composed of B(C_6_F_5_)_3_ and 2,2,6,6‐tetramethylpiperidine (TMP) and uses naturally occurring γ‐terpinene as a dihydrogen surrogate. A diverse array of silyl enol ethers undergo efficient hydrogenation, accessing the reduced products in excellent isolated yields (29 examples, 82 % average yield). Ongoing studies are focused on further applications of FLPs in catalysis and these results will be reported in due course.

## Conflict of interest

The authors declare no conflict of interest.

## Supporting information

As a service to our authors and readers, this journal provides supporting information supplied by the authors. Such materials are peer reviewed and may be re‐organized for online delivery, but are not copy‐edited or typeset. Technical support issues arising from supporting information (other than missing files) should be addressed to the authors.

Supplementary
