# Supplementary material for: FLP‐Catalyzed Transfer Hydrogenation of Silyl Enol Ethers
Source: Angew Chem Int Ed Engl. 2018 Aug 24;57(38):12356–9. doi: 10.1002/anie.201808800 (PMC6207922; doi:10.1002/anie.201808800)
Supplement: Supplementary file 1 — Supplementary [file ANIE-57-12356-s001.pdf]

Angewandte Chemie
Eine Zeitschrift der Gesellschaft Deutscher Chemiker

Supporting Information

FLP-Catalyzed Transfer Hydrogenation of Silyl Enol Ethers

Imtiaz Khan, Benjamin G. Reed-Berendt, Rebecca L. Melen, and Louis C. Morrill**

anie_201808800_sm_miscellaneous_information.pdf

SUPPORTING INFORMATION

Table of Contents

1.1 General information 2

1.2 Experimental and characterization data 4

- **1.2.1 Synthesis of silyl enol ethers** 4
- **1.2.2. Synthesis of 1,5-dimethoxycyclohexa-1,4-diene** 52
- **1.2.3. Optimization studies** 55
- **1.2.4. FLP-catalyzed transfer hydrogenation of silyl enol ethers** 56
- **1.2.5. FLP-catalyzed transfer hydrogenation of enamines** 103

1.3 References 106

1.1 General information

Unless stated otherwise, all reactions were performed using oven-dried microwave vials sealed with an aluminium crimp caps and were stirred with Teflon-coated magnetic stirrer bars. Dry tetrahydrofuran (THF), toluene, hexanes and diethyl ether were obtained after passing these previously degassed solvents through activated alumina columns (Mbraun, SPS-800). All other solvents and commercial reagents were used as supplied without further purification unless stated otherwise.

A nitrogen-filled glove box (MBraun) was used to manipulate reagents including the storage of starting materials, catalysts and preparation of reactions.

Room temperature (rt) refers to 20-25 °C. Temperatures of 0 °C and –78 °C were obtained using ice/water and CO_2(s)/acetone baths respectively. All reactions involving heating were carried out using DrySyn blocks and a contact thermometer. *In vacuo* refers to the use of a rotary evaporator under reduced pressure.

Analytical thin layer chromatography was carried out using aluminium plates coated with silica (Kieselgel 60 F_{254} silica) and visualization was achieved using ultraviolet light (254 nm), followed by staining with a 1% aqueous $KMnO_4$ solution. Flash chromatography used Kieselgel 60 silica in the solvent system stated.

Melting points were recorded on an Electrothermal 100 apparatus and are reported corrected by linear calibration to benzophenone (47-49 °C) and benzoic acid (121-123 °C).

Infra red spectra were recorded on a Shimadzu IRAffinity-1 Fourier Transform ATIR spectrometer as thin films using a Pike MIRacle ATR accessory. Characteristic peaks are quoted (ν_{max} / cm^{-1}).

^{1}H, $^{13}C\{^{1}H\}$, $^{19}F\{^{1}H\}$ NMR spectra were obtained on either a Bruker Avance 400 (400 MHz ^{1}H, 101 MHz ^{13}C, 376 MHz ^{19}F, 128 MHz ^{11}B) or a Bruker Avance 500 (500 MHz ^{1}H, 126 MHz ^{13}C, 471 MHz ^{19}F) spectrometer at rt in the solvent stated. Chemical shifts are reported in parts per million (ppm) relative to the residual solvent signal. All coupling constants, *J*, are

quoted in Hz. Multiplicities are reported with the following symbols: s = singlet, d = doublet, t = triplet, q = quartet, m = multiplet and multiples thereof. The abbreviation Ph to denote phenyl, TMS to denote trimethylsilyl, TES to denote triethylsilyl, TBS to denote *tert*-butyldimethylsilyl, TIPS to denote triisopropylsilyl and TBDPS to denote *tert*-butyldiphenylsilyl.

High resolution mass spectrometry (HRMS, *m/z*) data was acquired at the EPSRC UK National Mass Spectrometry Facility at Swansea University.

Tris(fluorophenyl)boranes including tris(pentafluorophenyl)borane $B(C_6F_5)_3$, tris(2,4,6-trifluorophenyl)borane $B(2,4,6\text{-}F_3C_6H_2)_3$, tris(2,6-difluorophenyl)borane $B(2,6\text{-}F_2C_6H_3)_3$ and enamines **31**, **33** and **34** were prepared as described in literature.[1–3]

1.2 Experimental and characterization data

1.2.1. Synthesis of silyl enol ethers

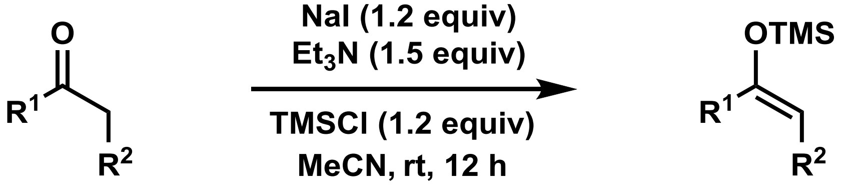

General procedure 1

A RBF containing a mixture of ketone (1 equiv) and pre-dried sodium iodide (1.2 equiv) was evacuated and filled with nitrogen three times, and dry acetonitrile (1.5 mL/mmol) was added, stirring for 5 min at rt. To the resulting solution, triethylamine (1.5 equiv) was added, followed by chlorotrimethylsilane (1.2 equiv). The reaction mixture was stirred overnight at room temperature. The reaction was quenched with a mixture of petroleum ether (50 mL) and saturated NH_4Cl (50 mL) at 0 °C. The organic phase was separated and the aqueous layer was extracted with petroleum ether (2 × 30 mL). The combined organic fractions were washed with ice-water (50 mL) and saturated NH_4Cl (50 mL), and then dried over anhydrous $MgSO_4$. The solvent was removed under reduced pressure and the residue was purified by Kugelrohr distillation.

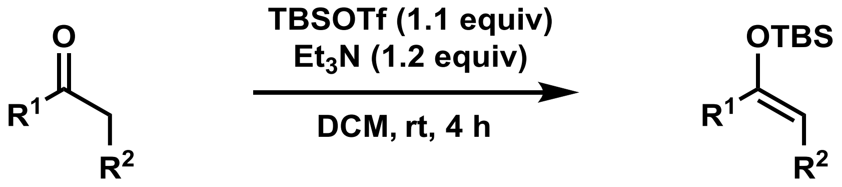

General Procedure 2

To an oven dried round bottom flask under nitrogen was added the corresponding ketone (1 equiv) in dry DCM (2 mL/mmol) followed by triethylamine (1.2 equiv). The reaction mixture was stirred for 1 h at room temperature. TBSOTf (1.1 equiv) was added dropwise and the reaction mixture was further allowed to stir for 2-3 h. The reaction was quenched with cold aqueous NH_4Cl (50 mL) and extracted with diethyl ether (2 × 30 mL). The combined organic fractions were dried ($MgSO_4$), filtered and evaporated under reduced pressure. The crude residue was purified by flash column chromatography on triethylamine deactivated silica (100% petroleum ether) to provide pure silyl enol ethers.

Trimethyl((1-phenylvinyl)oxy)silane

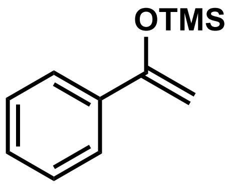

The title compound trimethyl((1-phenylvinyl)oxy)silane was prepared according to general procedure 1 from acetophenone (2.33 mL, 20.0 mmol, 1 equiv), triethylamine (4.18 mL, 30.0 mmol, 1.5 equiv), sodium iodide (3.60 g, 24.0 mmol, 1.2 equiv), and chlorotrimethylsilane (3.04 mL, 24.0 mmol, 1.2 equiv) and purified by Kugelrohr distillation (bp 90-91 °C (12 mmHg)) {Lit.[4] 92-94 °C (15 mmHg)} to give a colorless oil (3.63 g, 95% yield). R_f = 0.27 (eluent = 100% petroleum ether); ν_{max} / cm^{-1} (film) 2960, 1573, 1492, 1251, 839, 696; ^{1}H NMR (500 MHz, $CDCl_3$) δ_H: 0.34 (9H, s), 4.50 (1H, d, *J* 1.6), 4.99 (1H, d, *J* 1.6), 7.33-7.40 (3H, m), 7.66-7.68 (3H, m); ^{13}C{^{1}H} NMR (126 MHz, $CDCl_3$) δ_C: 0.2, 91.2, 125.3, 128.2, 128.3, 137.6, 155.7; HRMS (ASAP$^+$) calculated for $[C_{11}H_{15}OSi]^+$ $(M–H)^+$: m/z 191.0892, found 191.0894 (+1.0 ppm).

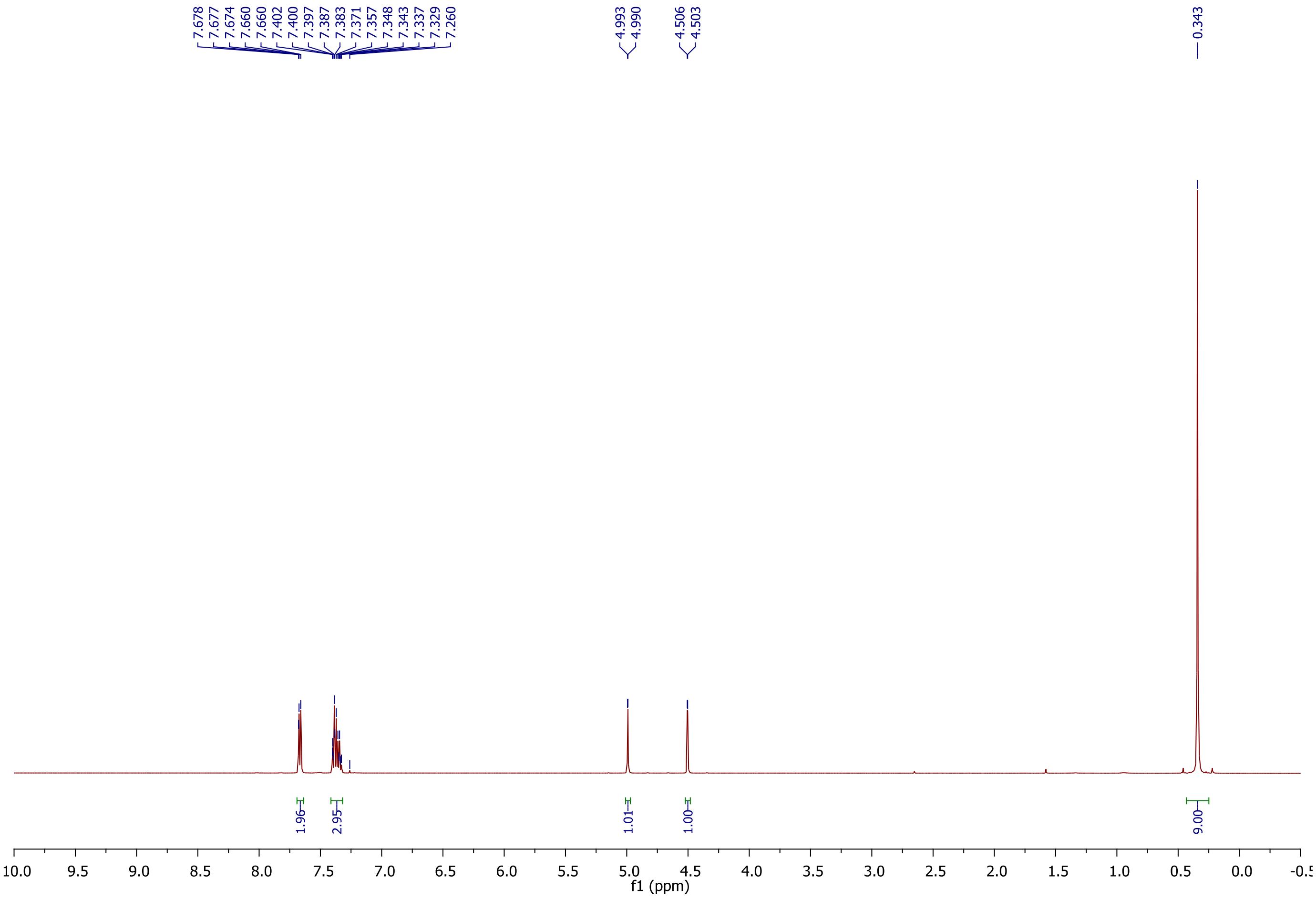

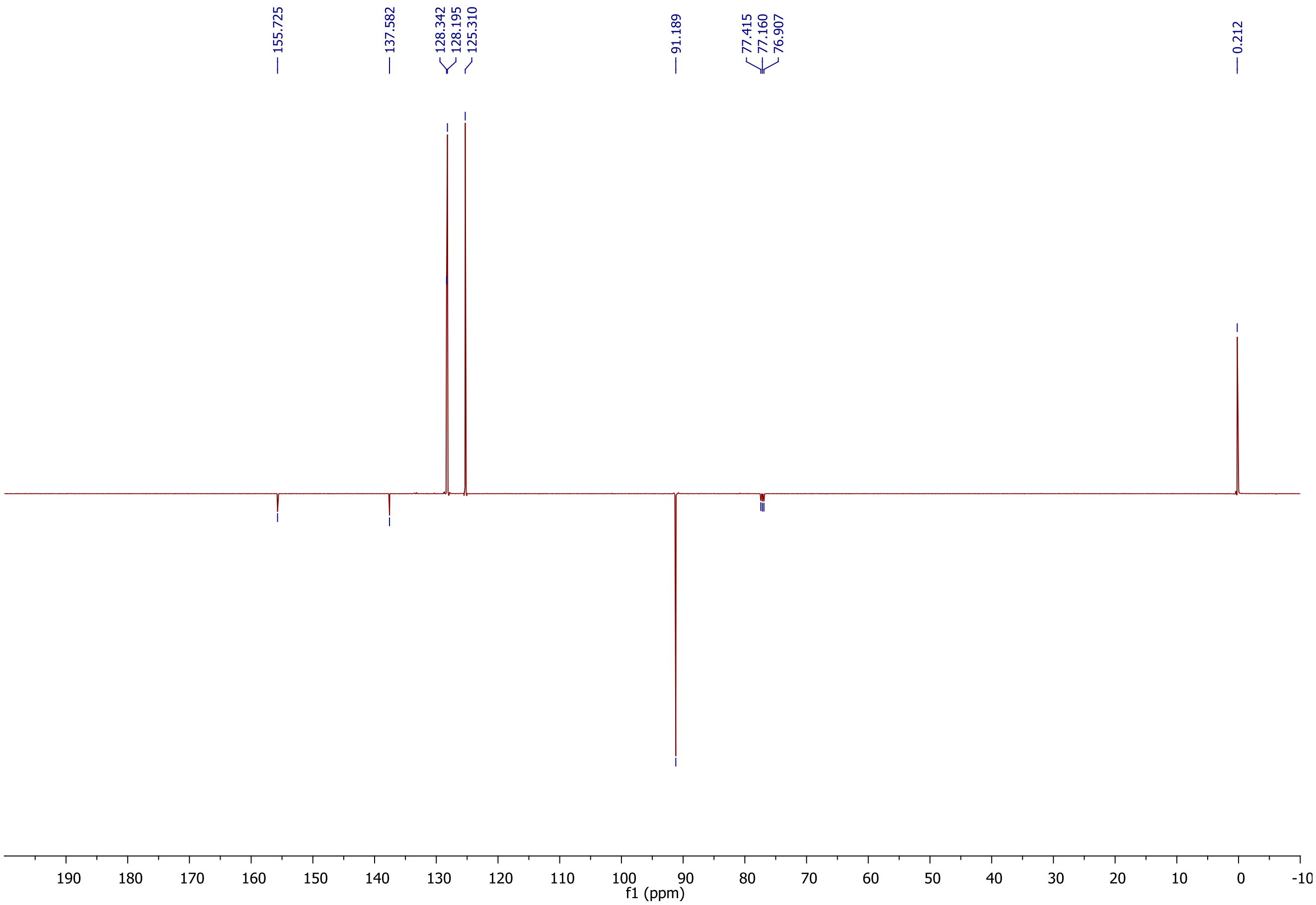

Triethyl((1-phenylvinyl)oxy)silane

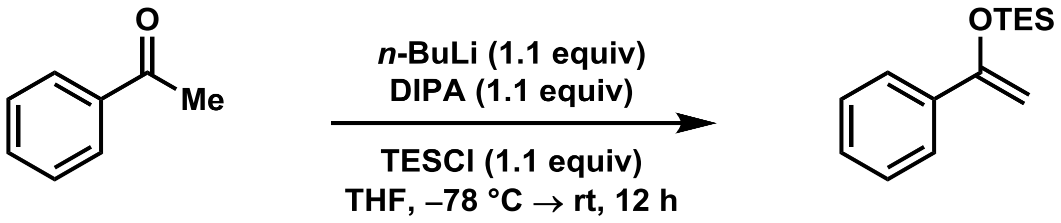

To a stirred solution of diisopropylamine (3.10 mL, 22.0 mmol, 1.1 equiv) in THF (40 mL) at –78 °C was added *n*-BuLi (8.80 mL, 22.0 mmol, 1.1 equiv) dropwise, and the solution was stirred for 30 min. To this solution acetophenone (2.33 mL, 20.0 mmol, 1 equiv) was added followed by chlorotriethylsilane (3.69 mL, 22.0 mmol, 1.1 equiv), and the reaction mixture was stirred at room temperature for 12 h. The reaction mixture was diluted with H_2O (40 mL), extracted with hexanes (2 × 60 mL), washed with brine (3 × 60 mL), dried ($MgSO_4$), filtered and evaporated under reduced pressure. The resulting crude oil was purified by flash silica column chromatography (eluent = 100% petroleum ether) to give a colorless oil (3.95 g, 84%

yield). R_f = 0.45 (eluent = 100% petroleum ether); ^{1}H NMR (500 MHz, $CDCl_3$) δ_H: 0.83 (6H, q, *J* 8.0), 1.07 (9H, t, *J* 8.0), 4.49 (1H, d, *J* 1.7), 4.94 (1H, d, *J* 1.8), 7.32-7.39 (3H, m), 7.67-7.69 (2H, m); $^{13}C\{^1H\}$ NMR (126 MHz, $CDCl_3$) δ_c: 5.1, 6.9, 90.5, 125.3, 128.2, 128.3, 137.7, 155.9. Spectroscopic data in accordance with that stated in the literature.[5]

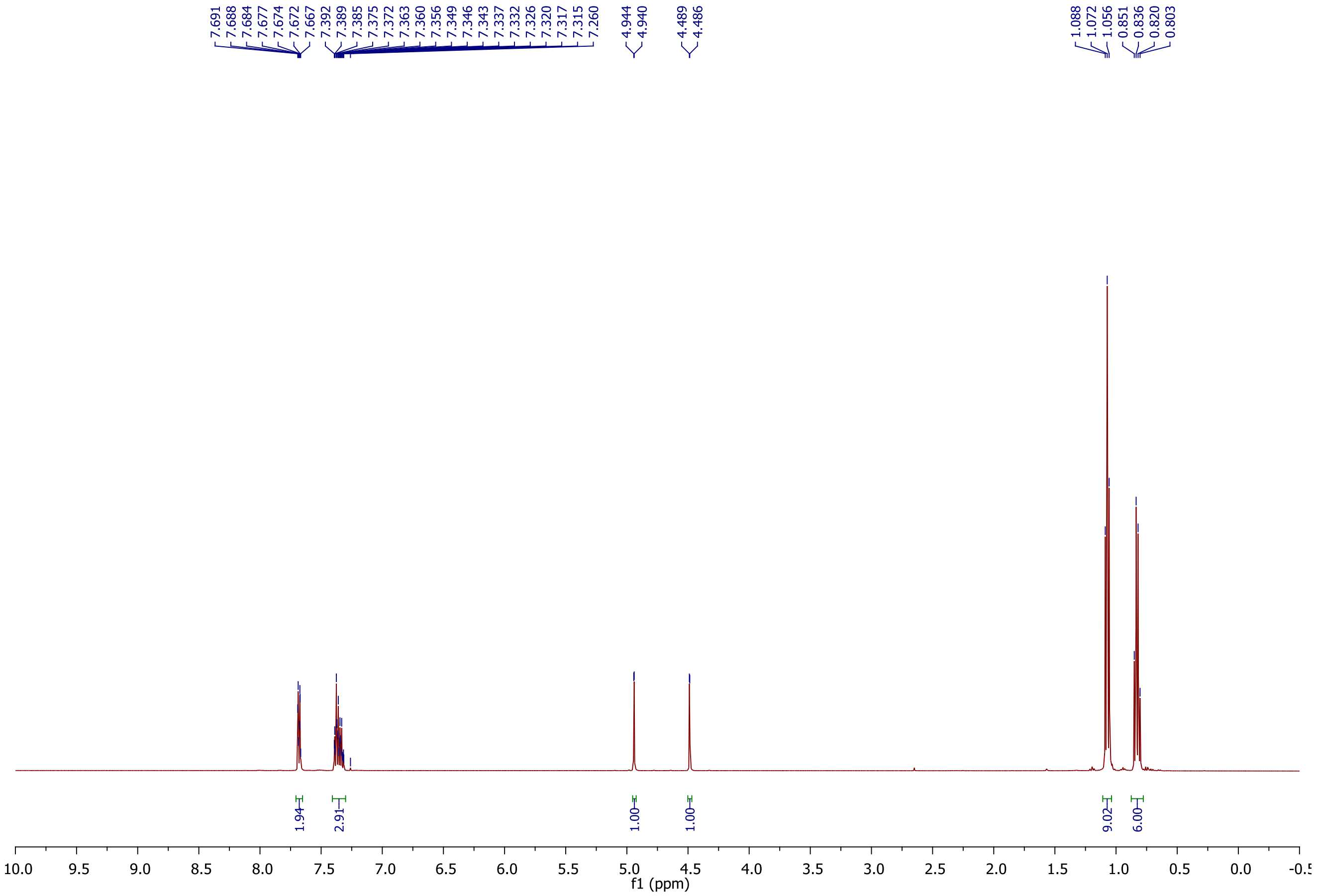

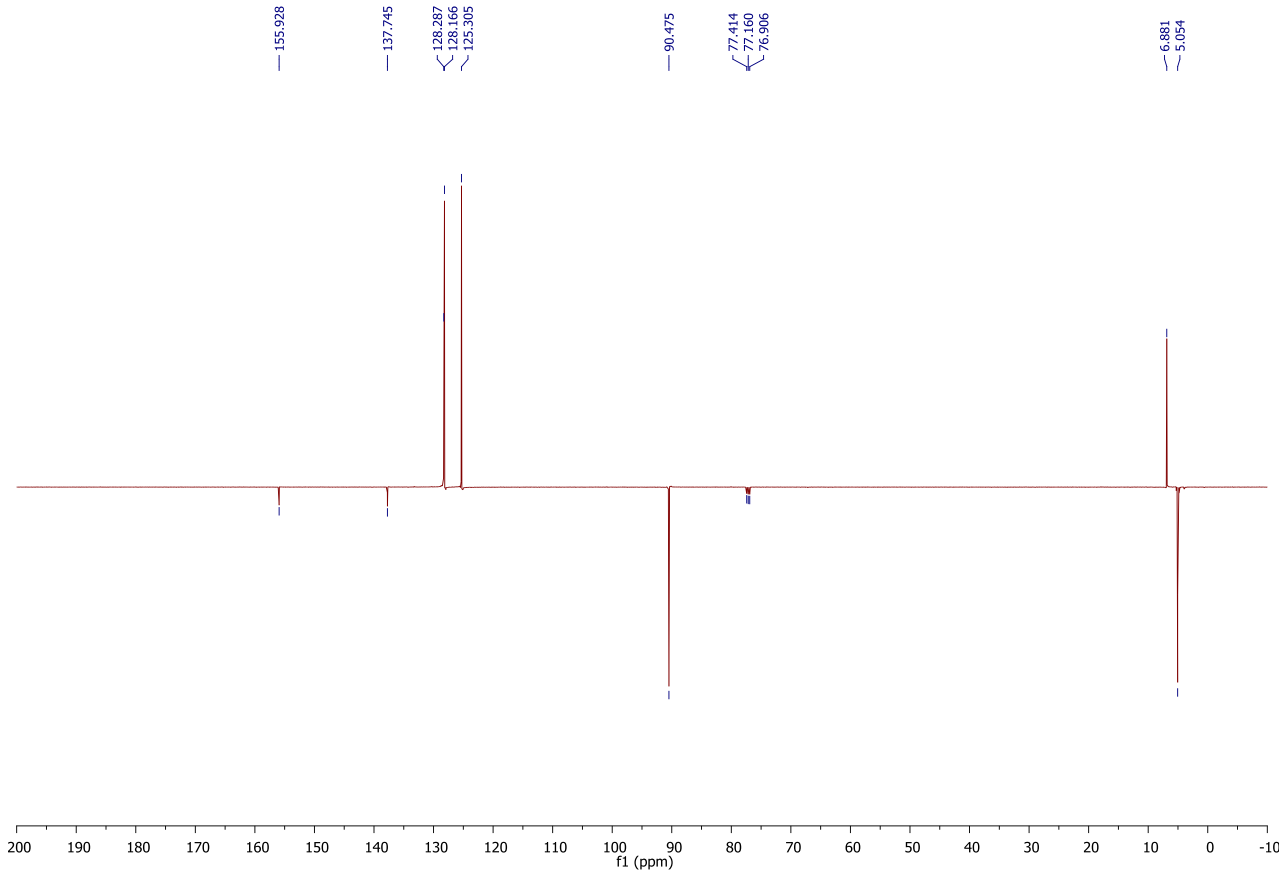

tert-Butyldimethyl((1-phenylvinyl)oxy)silane

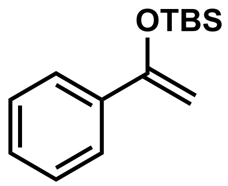

The title compound _tert_-butyldimethyl((1-phenylvinyl)oxy)silane was prepared according to general procedure 2 from acetophenone (1.17 mL, 10.0 mmol, 1 equiv), triethylamine (1.67 mL, 12.0 mmol, 1.2 equiv), and TBSOTf (2.52 mL, 11.0 mmol, 1.1 equiv) and purified by flash silica column chromatography to give a colorless oil (2.25 g, 96% yield). R_f = 0.32 (eluent = 100% petroleum ether); ^{1}H NMR (500 MHz, $CDCl_3$) δ_H: 0.27 (6H, s), 1.06 (9H, s), 4.47 (1H, d, _J_ 1.7), 4.94 (1H, d, _J_ 1.7), 7.31-7.39 (3H, m), 7.65-7.67 (2H, m); $^{13}C\{^1H\}$ NMR (126 MHz, $CDCl_3$) δ_c: -4.5, 18.5, 26.0, 91.0, 125.4, 128.2, 128.3, 137.9, 156.1. Spectroscopic data in accordance with that stated in the literature.[6]

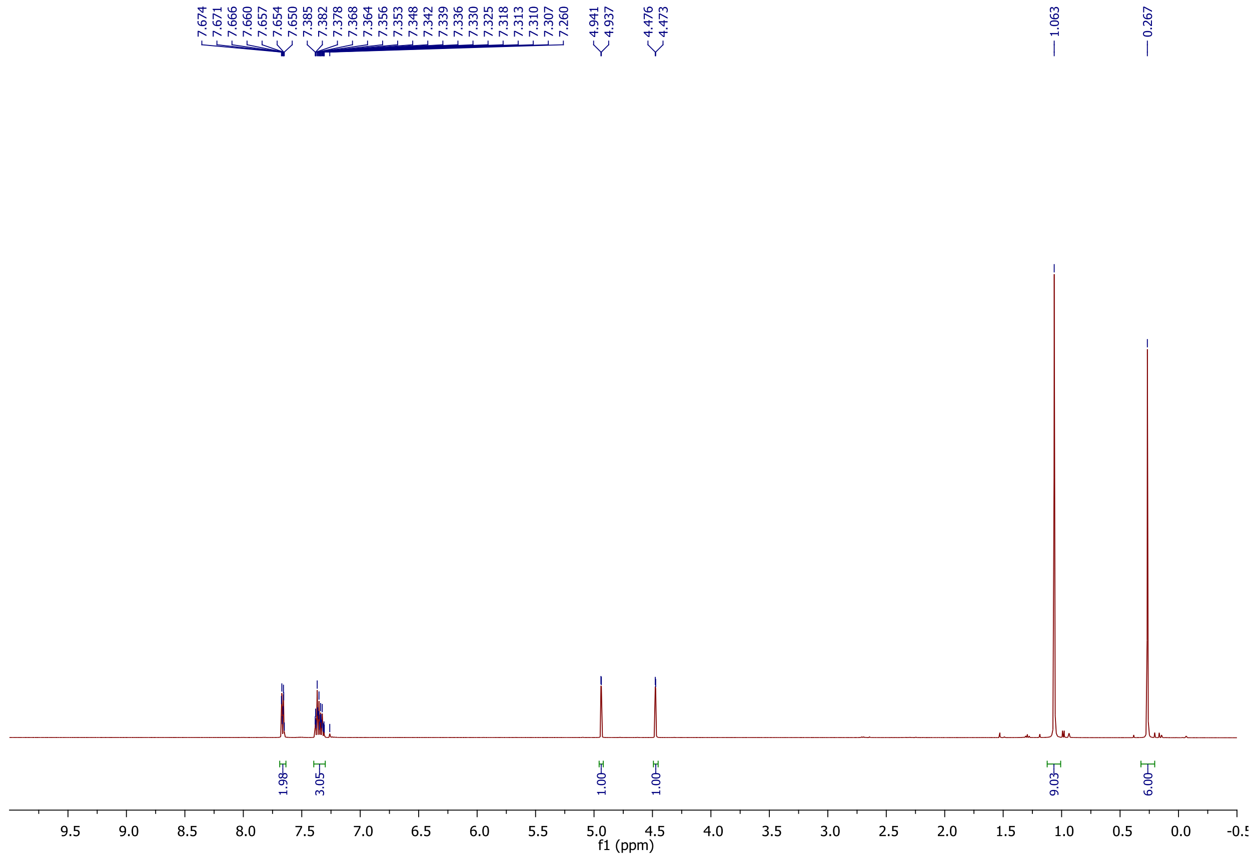
7.674
7.671
7.666
7.660
7.657
7.654
7.650
7.385
7.382
7.378
7.368
7.364
7.356
7.353
7.348
7.342
7.339
7.336
7.330
7.325
7.318
7.313
7.310
7.307
7.260
4.941
4.937
4.476
4.473
1.063
0.267
1.98
3.05
1.00
1.00
9.03
6.00
f1 (ppm)

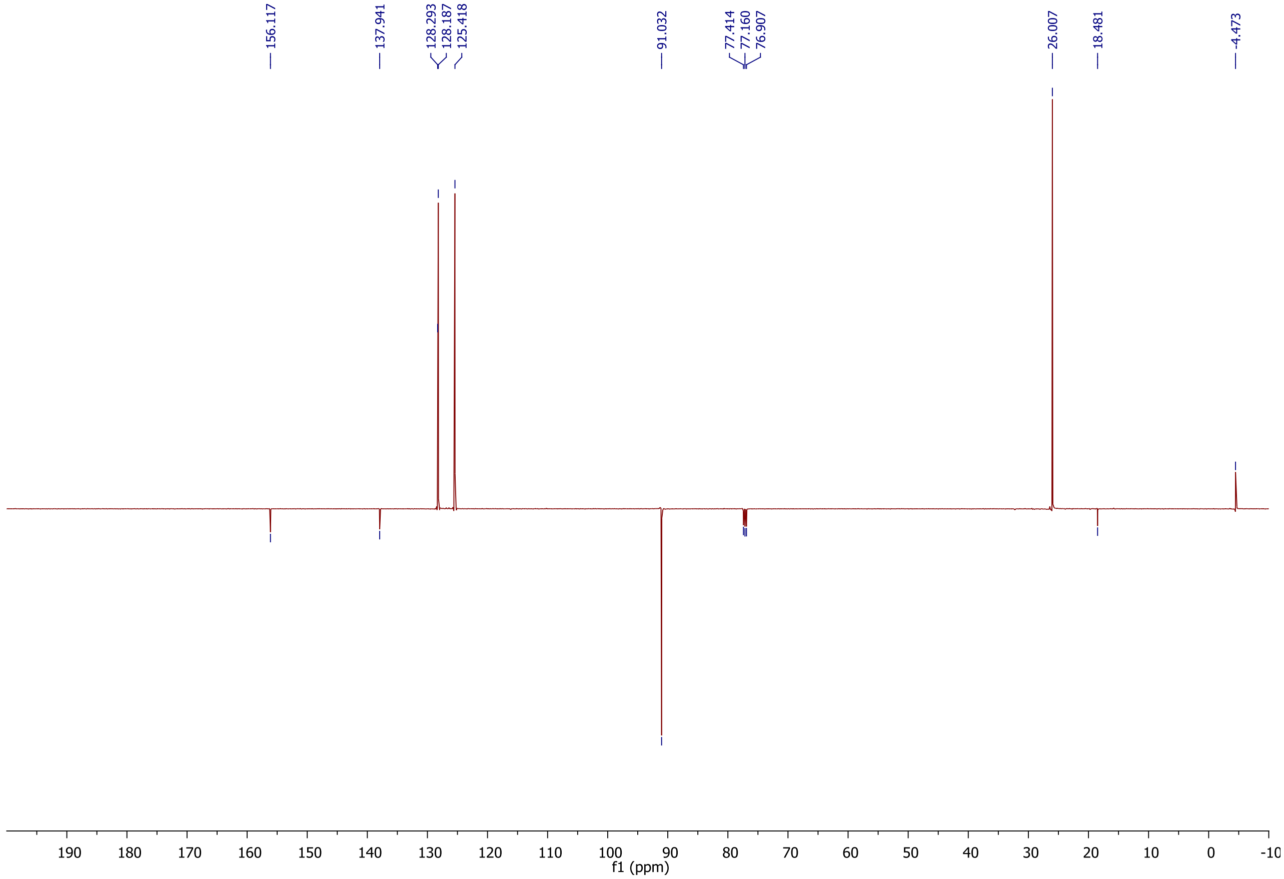
156.117
137.941
128.293
128.187
125.418
91.032
77.414
77.160
76.907
26.007
18.481
-4.473
f1 (ppm)

Triisopropyl((1-phenylvinyl)oxy)silane

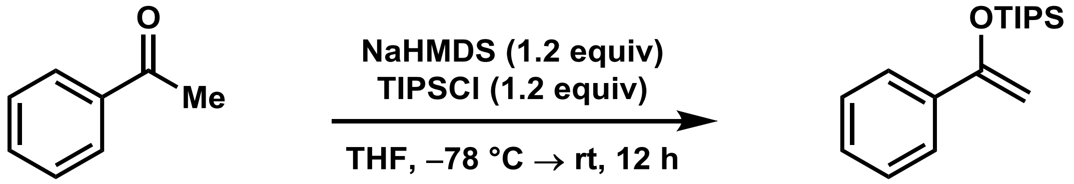

In an oven-dried flask sealed with a septum and under N_2 atmosphere, acetophenone (2.33 mL, 20.0 mmol, 1 equiv) was added in anhydrous THF (30 mL) followed by NaHMDS (24 mL, 24.0 mmol, 1.2 equiv) at –78 °C. The resulting pale yellow solution was stirred for 1 h at room temperature. The reaction mixture was cooled again to 0 °C and triisopropylsilyl chloride (5.13 mL, 24.0 mmol, 1.2 equiv) was added dropwise. The reaction mixture was stirred at room temperature for 12 h and then concentrated under reduced pressure. The resulting crude oil was purified by flash silica column chromatography (eluent = 100% petroleum ether) to give a colorless oil (5.02 g, 91% yield). R_f = 0.52 (eluent = 100% petroleum ether); ^{1}H NMR (500 MHz, $CDCl_3$) δ_H: 1.16 (18H, d, *J* 7.5), 1.28-1.38 (3H, m), 4.44 (1H, d, *J* 1.8), 4.88 (1H, d, *J* 1.8), 7.30-7.37 (3H, m), 7.68-7.69 (2H, m); ^{13}C{^{1}H} NMR (126 MHz, $CDCl_3$) δ_c: 12.9, 18.3, 90.0, 125.4, 128.2, 128.2, 138.0, 156.2. Spectroscopic data in accordance with that stated in the literature.[6]

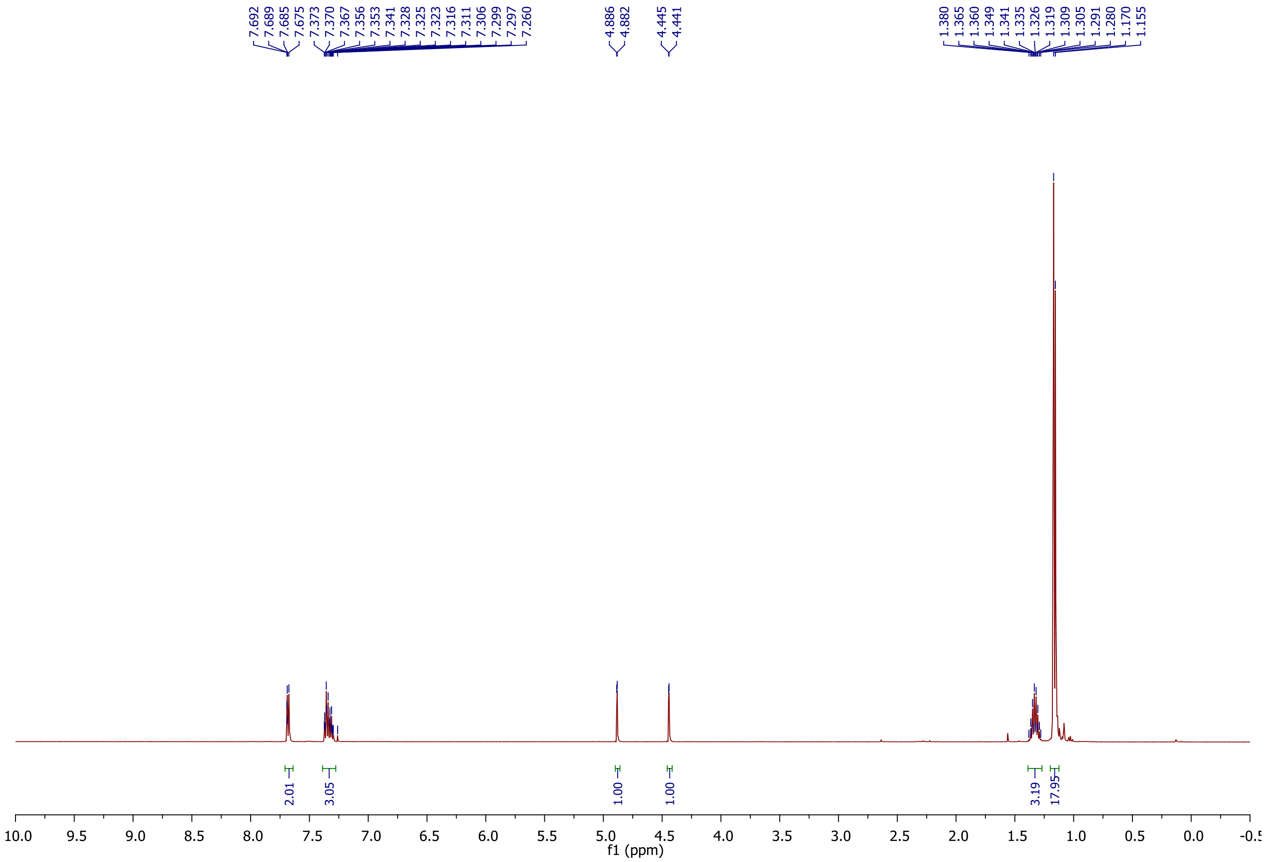
7.692
7.689
7.685
7.675
7.373
7.370
7.367
7.356
7.353
7.341
7.328
7.325
7.323
7.316
7.311
7.306
7.299
7.297
7.260
4.886
4.882
4.445
4.441
1.380
1.365
1.360
1.349
1.341
1.335
1.326
1.319
1.309
1.305
1.291
1.280
1.170
1.155
2.01
3.05
1.00
1.00
3.19
17.95
f1 (ppm)

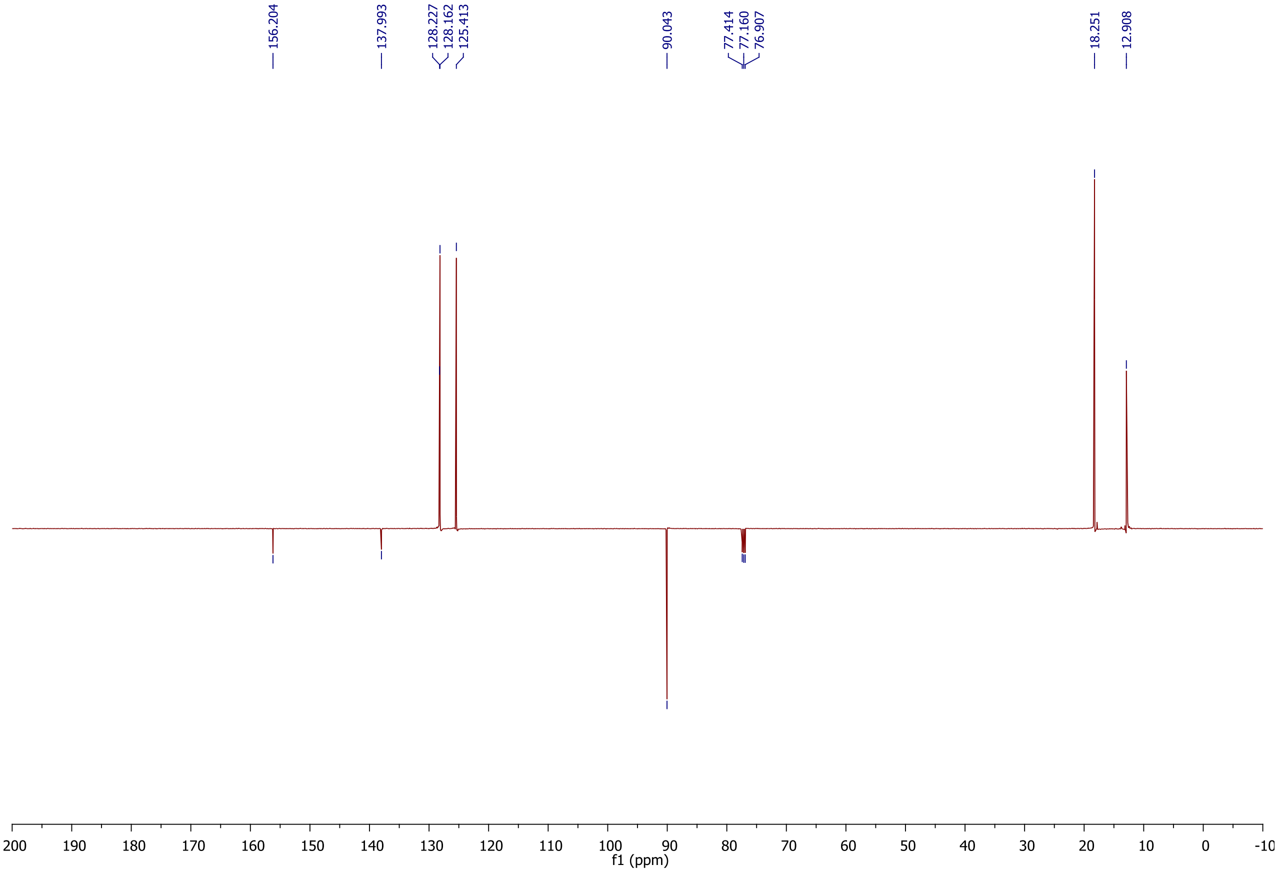
156.204
137.993
128.227
128.162
125.413
90.043
77.414
77.160
76.907
18.251
12.908
f1 (ppm)

tert-Butyldiphenyl((1-phenylvinyl)oxy)silane

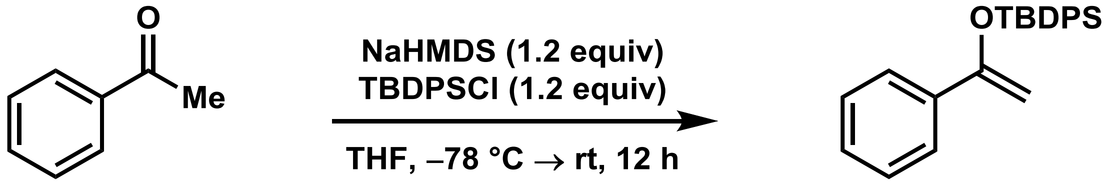

In an oven-dried flask sealed with a septum and under N_2 atmosphere, acetophenone (2.33 mL, 20.0 mmol, 1 equiv) was added in anhydrous THF (30 mL) followed by NaHMDS (24 mL, 24.0 mmol, 1.2 equiv) at –78 °C. The resulting pale yellow solution was stirred for 1 h at room temperature. The reaction mixture was cooled again to 0 °C and *tert*-butyl(chloro)diphenylsilane (6.24 mL, 24.0 mmol, 1.2 equiv) was added dropwise. The reaction mixture was stirred at room temperature for 12 h and then concentrated under reduced pressure. The resulting crude oil was purified by flash silica column chromatography (eluent = 100% petroleum ether) to give a colorless oil (5.96 g, 83% yield). R_f = 0.23 (eluent = 100% petroleum ether); ^{1}H NMR (500 MHz, $CDCl_3$) δ_H: 1.17 (9H, s), 4.08 (1H, d, *J* 2.3), 4.82 (1H, d, *J* 2.3), 7.37-7.51 (9H, m), 7.82-7.86 (6H, m); ^{13}C{^{1}H} NMR (126 MHz, $CDCl_3$) δ_c: 19.6, 26.7, 92.3, 125.3, 127.9, 128.3, 128.4, 130.0, 132.5, 135.6, 137.6, 155.1. Spectroscopic data in accordance with that stated in the literature.[7]

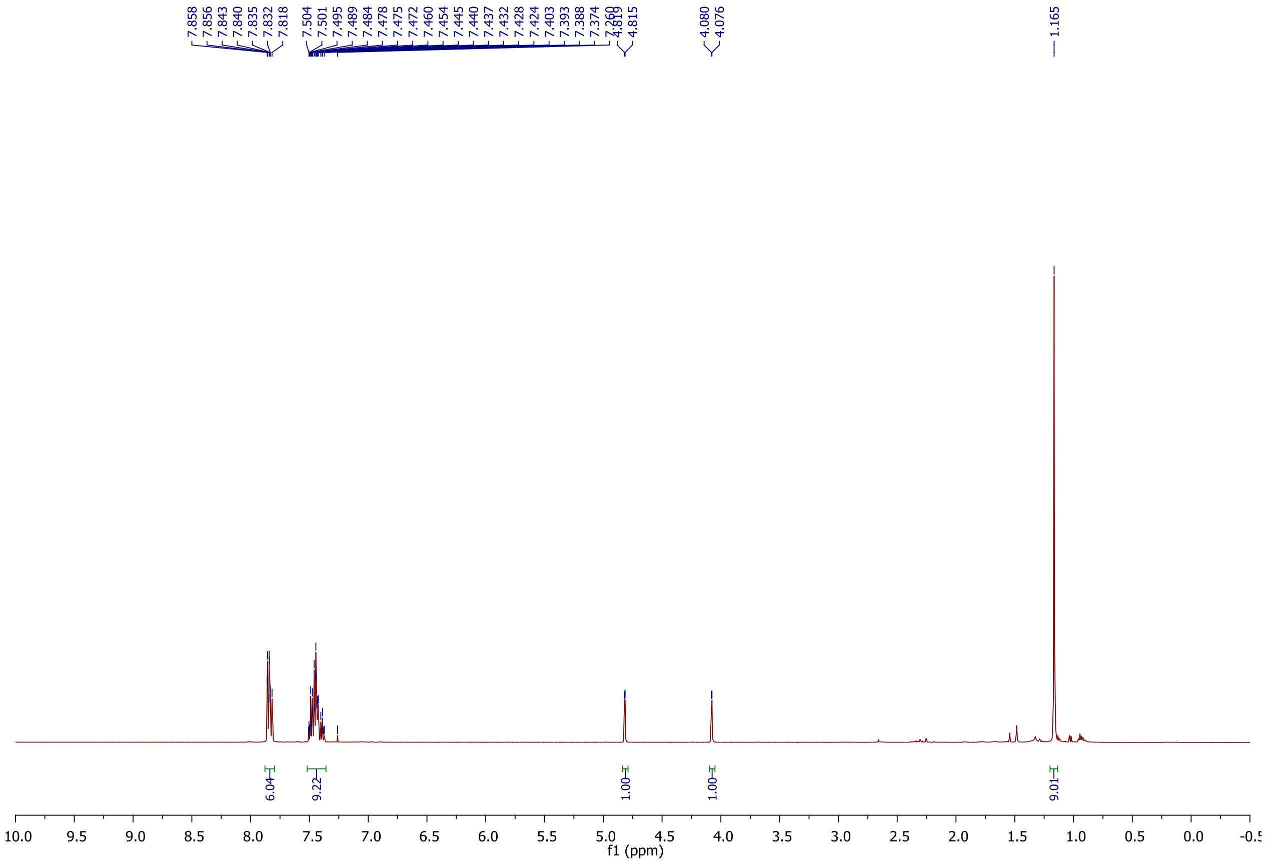
7.858
7.856
7.843
7.840
7.835
7.832
7.818
7.504
7.501
7.495
7.489
7.484
7.478
7.475
7.472
7.460
7.454
7.445
7.440
7.437
7.432
7.428
7.424
7.403
7.393
7.388
7.374
7.260
4.819
4.815
4.080
4.076
1.165
6.04
9.22
1.00
1.00
9.01
f1 (ppm)

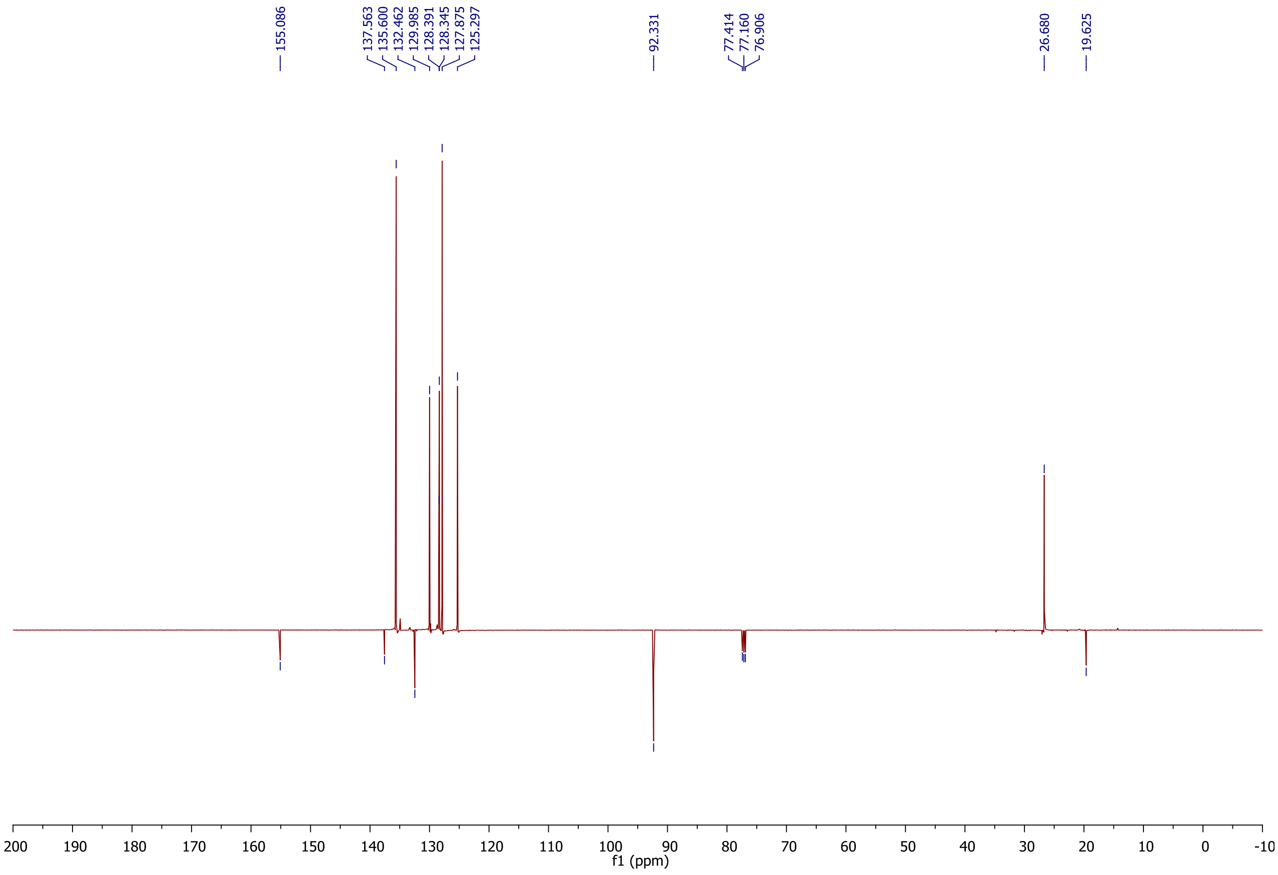
155.086
137.563
135.600
132.462
129.985
128.391
128.345
127.875
125.297
92.331
77.414
77.160
76.906
26.680
19.625
f1 (ppm)

tert-Butyldimethyl((1-(_p_-tolyl)vinyl)oxy)silane

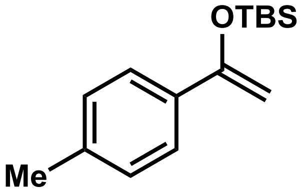

The title compound *tert*-butyldimethyl((1-(*p*-tolyl)vinyl)oxy)silane was prepared according to general procedure 2 from 4′-methylacetophenone (1.34 mL, 10.0 mmol, 1 equiv), triethylamine (1.67 mL, 12.0 mmol, 1.2 equiv), and TBSOTf (2.52 mL, 11.0 mmol, 1.1 equiv) and purified by flash silica column chromatography to give a colorless oil (1.81 g, 73% yield). R_f = 0.21 (eluent = 100% petroleum ether); ^{1}H NMR (400 MHz, $CDCl_3$) δ_H: 0.22 (6H, s), 1.02 (9H, s), 2.36 (3H, s), 4.39 (1H, d, *J* 1.6), 4.86 (1H, d, *J* 1.6), 7.13-7.16 (2H, m), 7.50-7.53 (2H, m); ^{13}C{^{1}H} NMR (101 MHz, $CDCl_3$) δ_c: -4.5, 18.5, 21.3, 26.0, 90.3, 125.4, 128.9, 135.2, 138.1, 156.2. Spectroscopic data in accordance with that stated in the literature.[8]

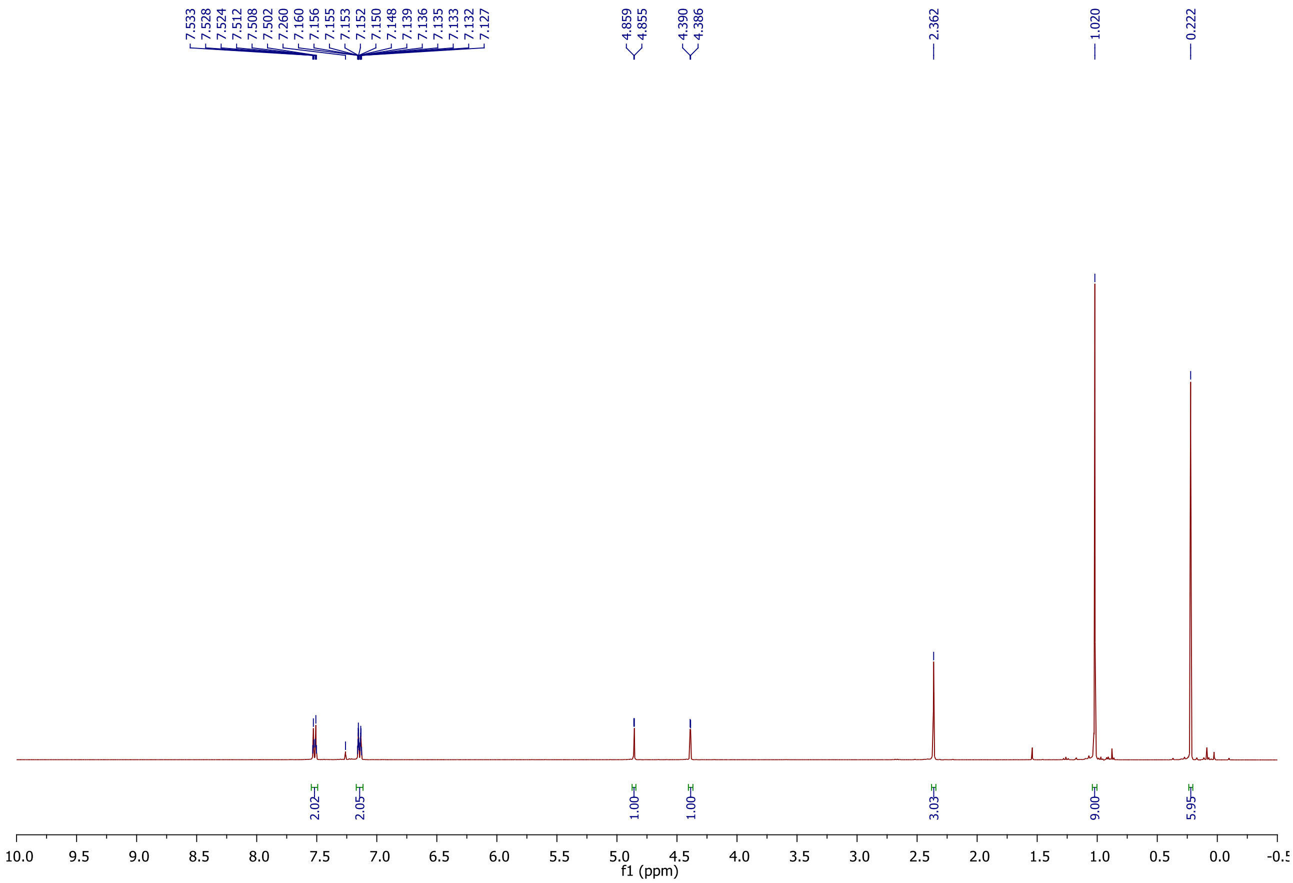

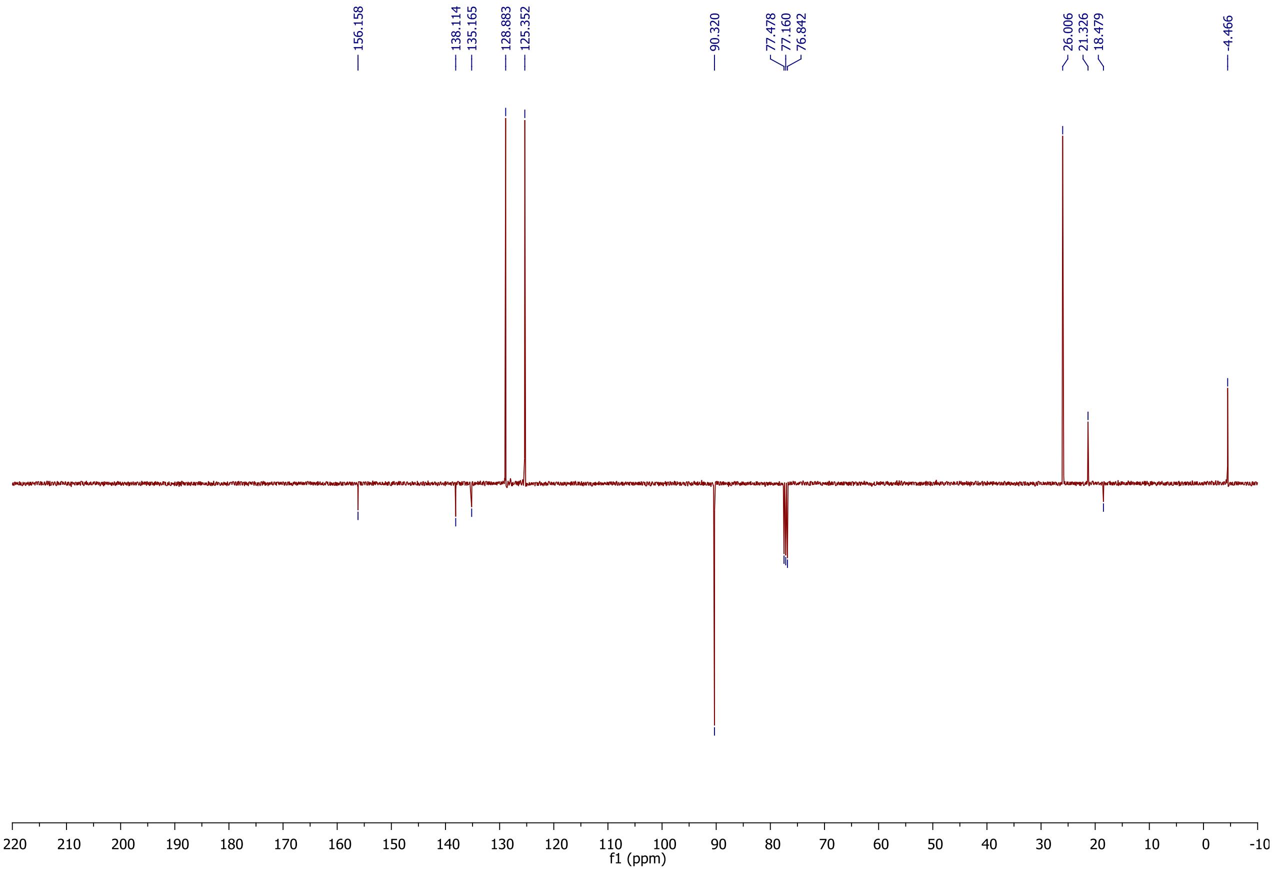

***tert*-Butyldimethyl((1-(*m*-tolyl)vinyl)oxy)silane**

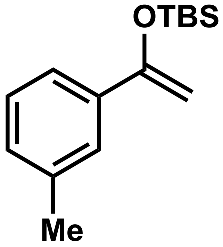

The title compound *tert*-butyldimethyl((1-(*m*-tolyl)vinyl)oxy)silane was prepared according to general procedure 2 from 3′-methylacetophenone (980 µL, 7.50 mmol, 1 equiv), triethylamine (1.25 mL, 9.00 mmol, 1.2 equiv), and TBSOTf (1.89 mL, 8.25 mmol, 1.1 equiv) and purified by flash silica column chromatography to give a colorless oil (1.70 g, 91% yield). R_f = 0.24 (eluent = 100% petroleum ether); ν_{max} / cm^{-1} (film) 2954, 1635, 1558, 1489, 1253, 1014, 829, 705; ^{1}H NMR (400 MHz, $CDCl_3$) δ_H: 0.23 (6H, s), 1.03 (9H, s), 2.38 (3H, s), 4.43 (1H, d, *J* 1.6), 4.89 (1H, d, *J* 1.6), 7.11-7.14 (1H, m), 7.23 (1H, t, *J* 7.6), 7.43-7.45 (2H, m); ^{13}C{^{1}H} NMR (101 MHz, $CDCl_3$) δ_c: -4.5, 18.5, 21.7, 26.0, 91.0, 122.6, 126.2, 128.1, 129.1, 137.7,

137.9, 156.3; HRMS (ASAP$^+$) calculated for $[C_{15}H_{25}OSi]^+$ $(M+H)^+$: m/z 249.1675, found 249.1681 (+2.4 ppm).

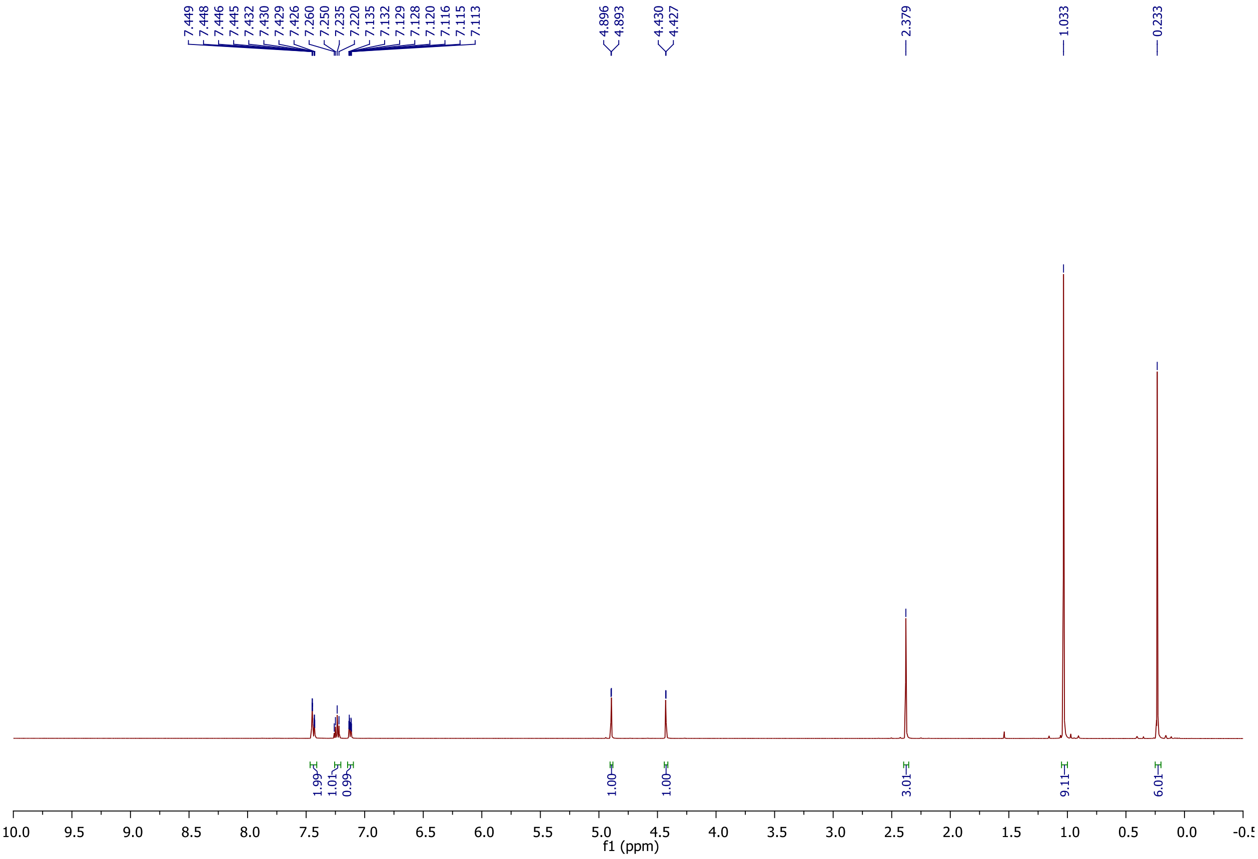

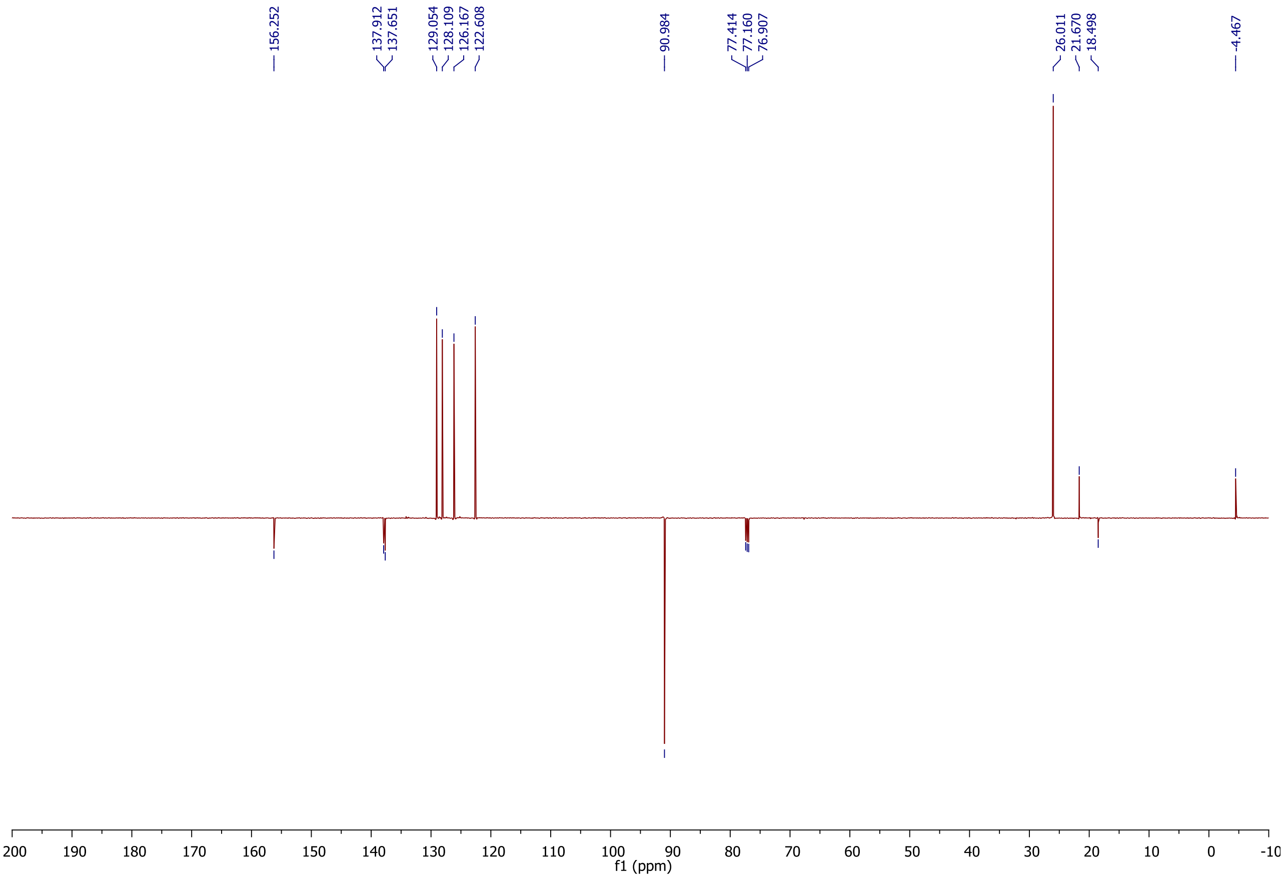

Trimethyl((1-(*o*-tolyl)vinyl)oxy)silane

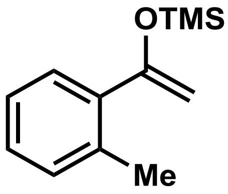

The title compound trimethyl((1-(*o*-tolyl)vinyl)oxy)silane was prepared according to general procedure 1 from 4'-methylacetophenone (1.30 mL, 10.0 mmol, 1 equiv), triethylamine (2.09 mL, 15.0 mmol, 1.5 equiv), sodium iodide (1.80 g, 12.0 mmol, 1.2 equiv), and chlorotrimethylsilane (1.52 mL, 12.0 mmol, 1.2 equiv) and purified by Kugelrohr distillation to give a colorless oil (1.75 g, 85% yield). R_f = 0.25 (eluent = 100% petroleum ether); ν_{max} / cm^{-1} (film) 2958, 1558, 1489, 1251, 1010, 839, 727; ^{1}H NMR (500 MHz, $CDCl_3$) δ_H: 0.21 (9H, s), 2.41 (3H, s), 4.42 (1H, d, *J* 1.0), 4.56 (1H, d, *J* 1.0), 7.14-7.17 (2H, m), 7.20-7.23 (1H, m), 7.32-7.34 (1H, m); $^{13}C\{^{1}H\}$ NMR (126 MHz, $CDCl_3$) δ_c: 0.2, 20.6, 95.0, 125.5, 128.2, 128.9, 130.5, 136.0, 139.1, 157.9. Spectroscopic data in accordance with that stated in the literature.[9]

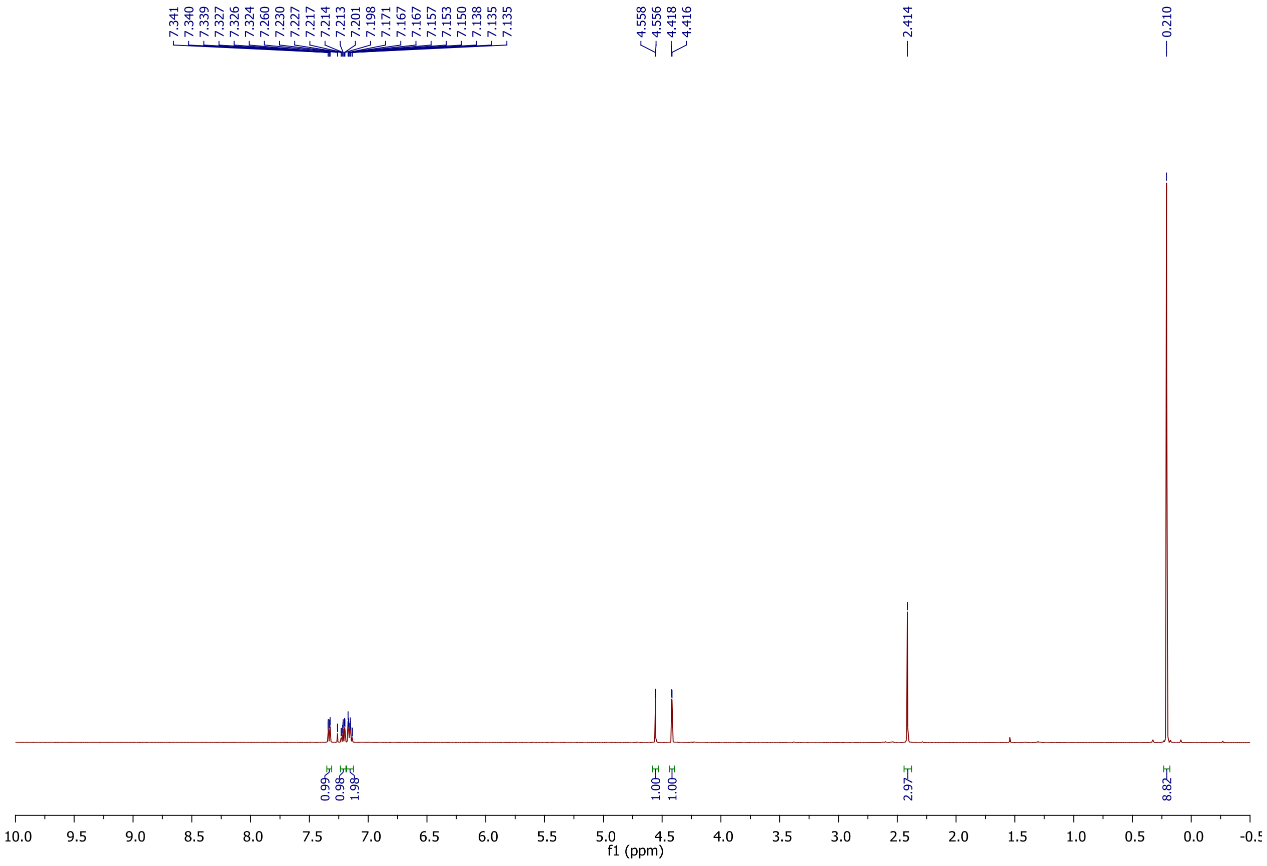
7.341
7.340
7.339
7.327
7.326
7.324
7.260
7.230
7.227
7.217
7.214
7.213
7.201
7.198
7.171
7.167
7.167
7.157
7.153
7.150
7.138
7.135
7.135
4.558
4.556
4.418
4.416
2.414
0.210
0.99
0.98
1.98
1.00
1.00
2.97
8.82
10.0 9.5 9.0 8.5 8.0 7.5 7.0 6.5 6.0 5.5 5.0 4.5 4.0 3.5 3.0 2.5 2.0 1.5 1.0 0.5 0.0
f1 (ppm)

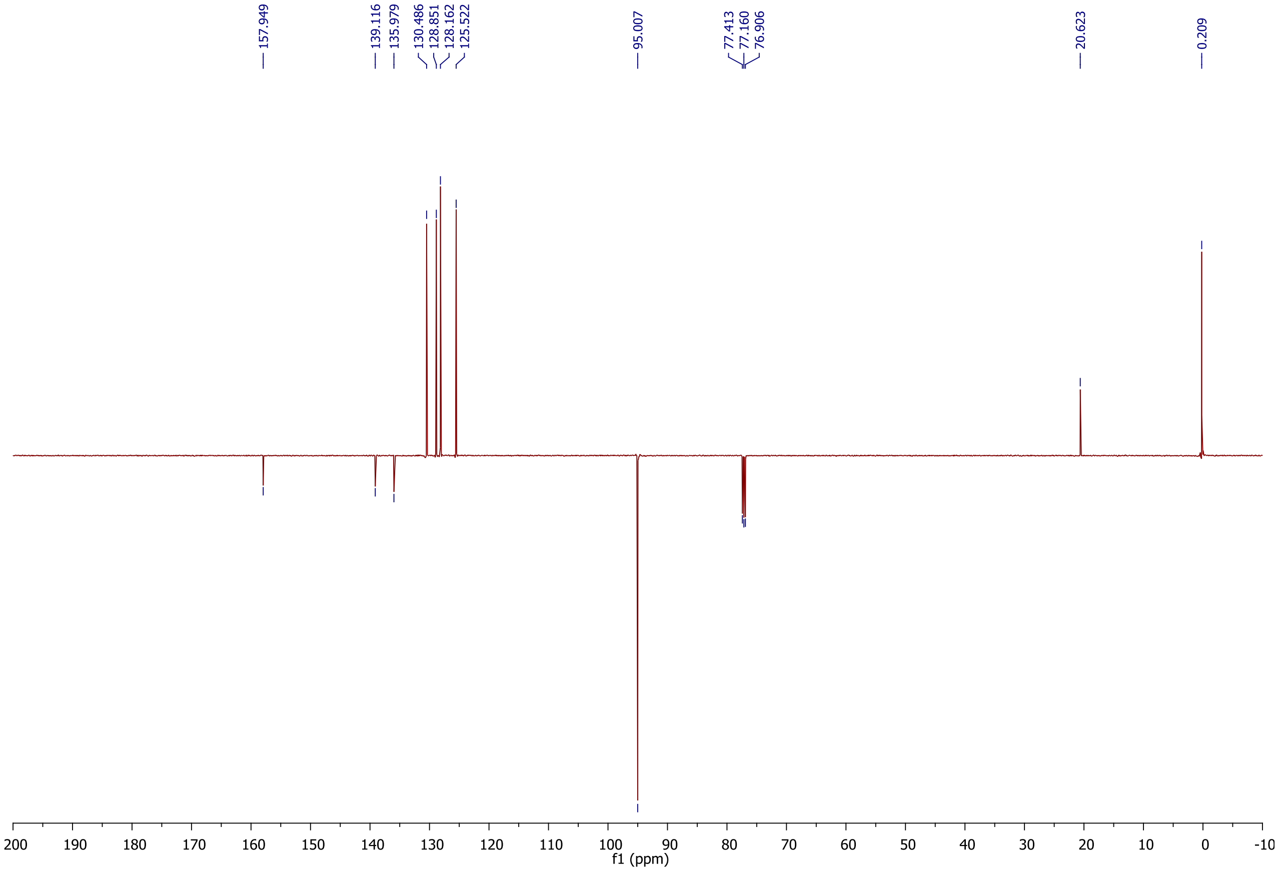
157.949
139.116
135.979
130.486
128.851
128.162
125.522
95.007
77.413
77.160
76.906
20.623
0.209
200 190 180 170 160 150 140 130 120 110 100 90 80 70 60 50 40 30 20 10 0 -10
f1 (ppm)

tert-Butyl((1-(3,4-dimethylphenyl)vinyl)oxy)dimethylsilane

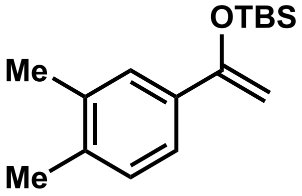

The title compound *tert*-butyl((1-(3,4-dimethylphenyl)vinyl)oxy)dimethylsilane was prepared according to general procedure 2 from 3′,4′-dimethylacetophenone (740 µL, 5.00 mmol, 1 equiv), triethylamine (836 µL, 6.00 mmol, 1.2 equiv), and TBSOTf (1.26 mL, 5.50 mmol, 1.1 equiv) and purified by flash silica column chromatography to give a colorless oil (1.12 g, 85% yield). R_f = 0.28 (eluent = 100% petroleum ether); ν_{max} / cm^{-1} (film) 2956, 2856, 1608, 1506, 1471, 1251, 1014, 827, 779; ^{1}H NMR (500 MHz, $CDCl_3$) δ_H: 0.23 (6H, s), 1.03 (9H, s), 2.28 (3H, s), 2.29 (3H, s), 4.38 (1H, d, *J* 1.5), 4.86 (1H, d, *J* 1.5), 7.10 (1H, d, *J* 7.9), 7.36-7.38 (1H, m), 7.41 (1H, s); ^{13}C{^{1}H} NMR (126 MHz, $CDCl_3$) δ_C: -4.5, 18.5, 19.7, 20.1, 26.0, 90.3, 122.9, 126.7, 129.5, 135.6, 136.2, 136.8, 156.3; HRMS (ASAP$^+$) calculated for $[C_{16}H_{27}OSi]^+$ $(M+H)^+$: m/z 263.1831, found 263.1838 (+2.7 ppm).

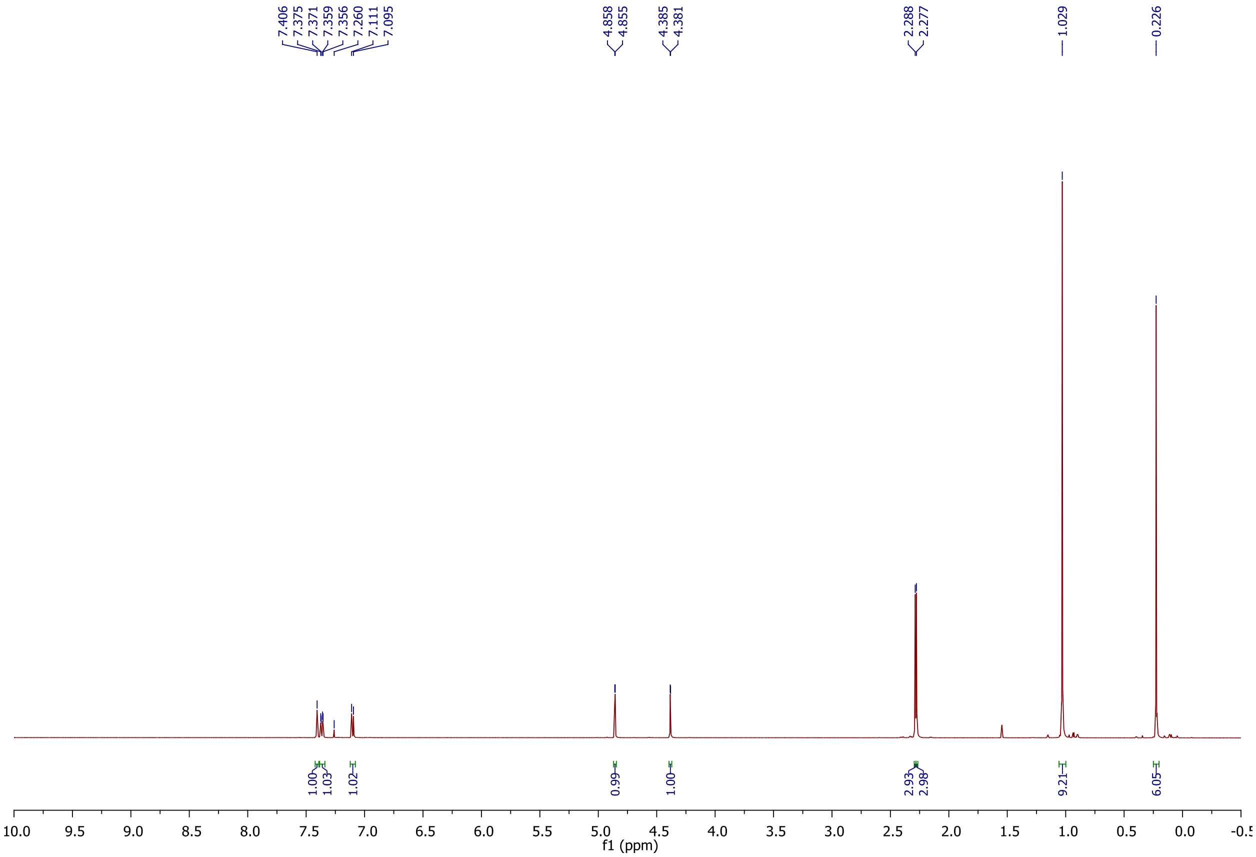

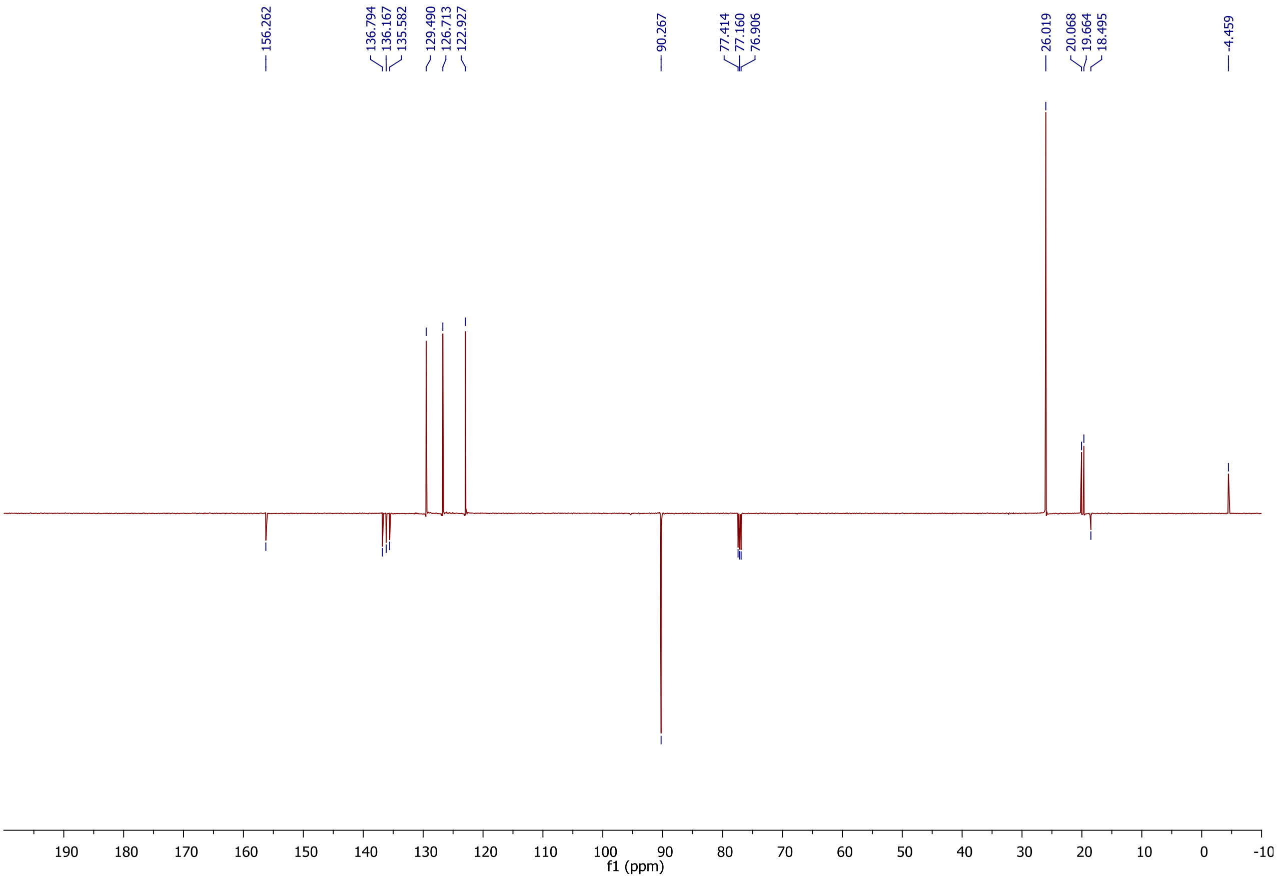

tert-Butyldimethyl((1-(5,6,7,8-tetrahydronaphthalen-2-yl)vinyl)oxy)silane

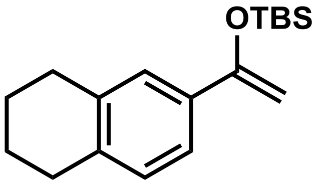

The title compound *tert*-butyldimethyl((1-(5,6,7,8-tetrahydronaphthalen-2-yl)vinyl)oxy)silane was prepared according to general procedure 2 from 6-acetyl-1,2,3,4-tetrahydronaphthalene (820 µL, 5.00 mmol, 1 equiv), triethylamine (836 µL, 6.00 mmol, 1.2 equiv), and TBSOTf (1.26 mL, 5.50 mmol, 1.1 equiv) and purified by flash silica column chromatography to give a colorless oil (1.31 g, 91% yield). R_f = 0.25 (eluent = 100% petroleum ether); ν_{max} / cm^{-1} (film) 2927, 2856, 1616, 1471, 1251, 1012, 827, 779; ^{1}H NMR (500 MHz, $CDCl_3$) δ_H: 0.23 (6H, s), 1.03 (9H, s), 1.80-1.83 (4H, m), 2.76-2.80 (4H, m), 4.37 (1H, d, *J* 1.4), 4.84 (1H, d, *J* 1.4), 7.03 (1H, d, *J* 7.9), 7.33-7.36 (2H, m); ^{13}C{^{1}H} NMR (126 MHz, $CDCl_3$) δ_c: -4.5, 18.5, 23.4, 23.4, 26.0, 29.4, 29.7, 90.2, 122.6, 126.1, 128.9, 135.2, 136.8, 137.5, 156.3; HRMS (ASAP$^+$) calculated for $[C_{18}H_{29}OSi]^+$ $(M+H)^+$: m/z 289.1988, found 289.1989 (+0.3 ppm).

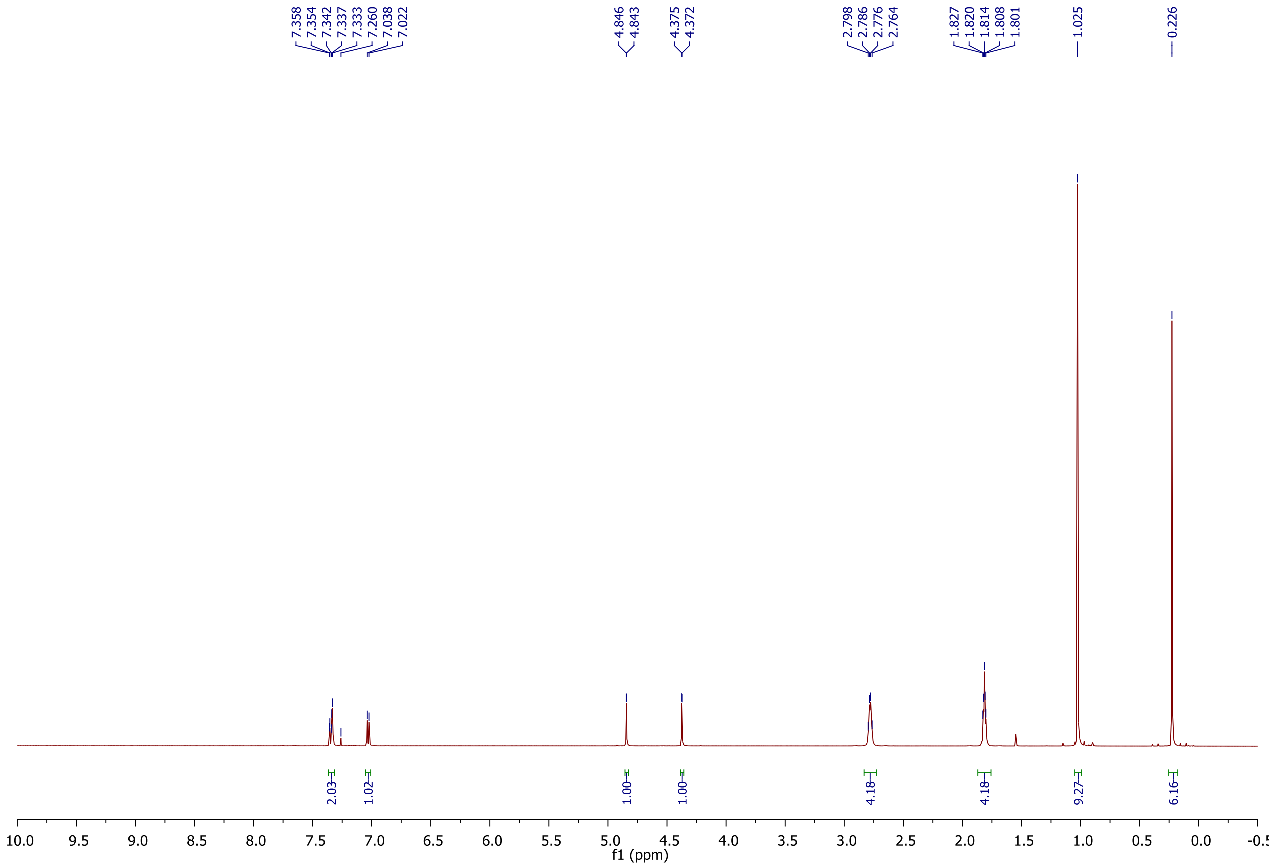
7.358
7.354
7.342
7.337
7.333
7.260
7.038
7.022
4.846
4.843
4.375
4.372
2.798
2.786
2.776
2.764
1.827
1.820
1.814
1.808
1.801
1.025
0.226
2.03
1.02
1.00
1.00
4.18
4.18
9.27
6.16
f1 (ppm)

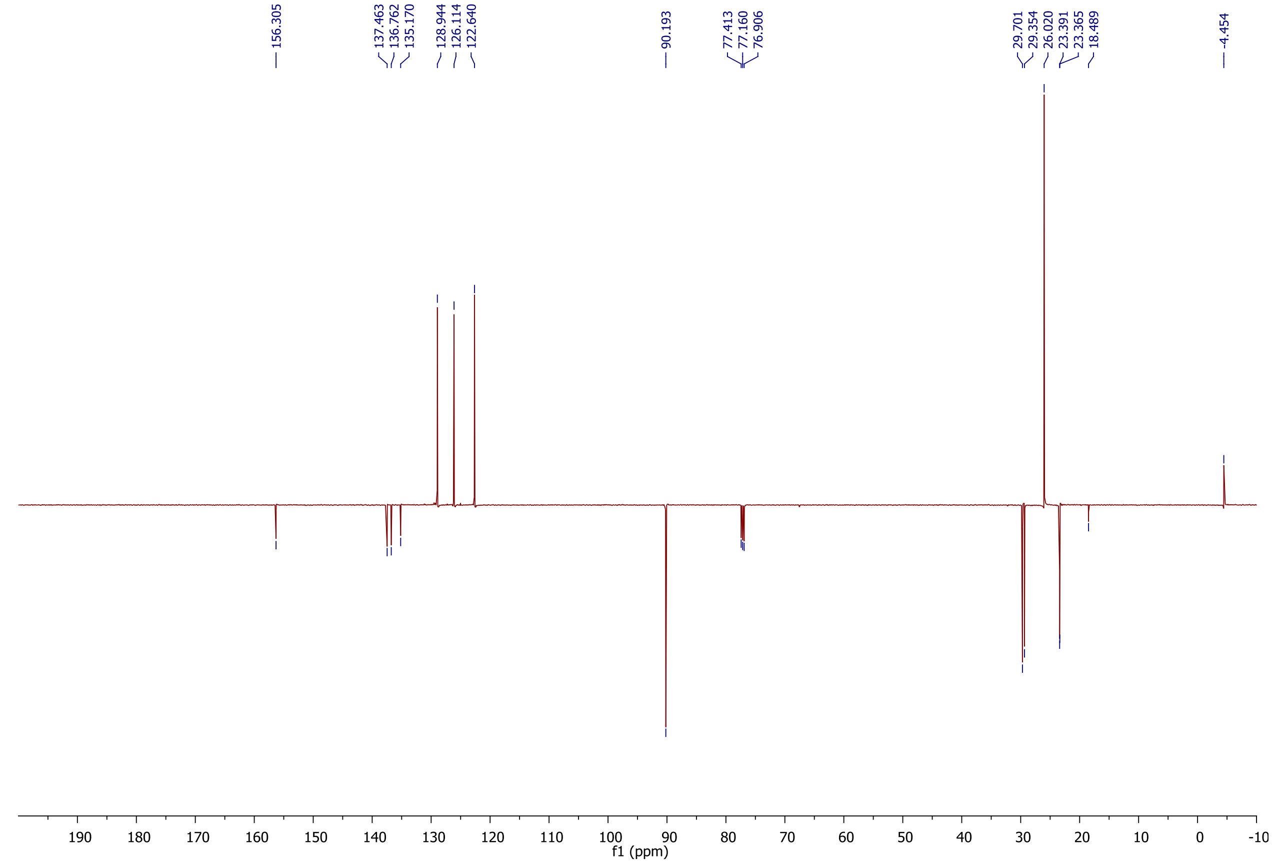
156.305
137.463
136.762
135.170
128.944
126.114
122.640
90.193
77.413
77.160
76.906
29.701
29.354
26.020
23.391
23.365
18.489
-4.454
f1 (ppm)

***tert*-Butyl((1-(4-ethylphenyl)vinyl)oxy)dimethylsilane**

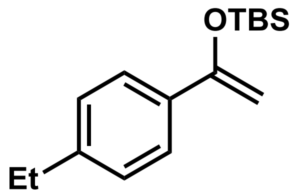

The title compound *tert*-butyl((1-(4-ethylphenyl)vinyl)oxy)dimethylsilane was prepared according to general procedure 2 from 4′-ethylacetophenone (746 µL, 5.00 mmol, 1 equiv), triethylamine (836 µL, 6.00 mmol, 1.2 equiv), and TBSOTf (1.26 mL, 5.50 mmol, 1.1 equiv) and purified by flash silica column chromatography to give a colorless oil (1.17 g, 89% yield). R_f = 0.26 (eluent = 100% petroleum ether); ν_{max} / cm^{-1} (film) 2958, 2858, 1616, 1508, 1471, 1251, 1002, 831, 779; ^{1}H NMR (500 MHz, $CDCl_3$) δ_H: 0.23 (6H, s), 1.03 (9H, s), 1.26 (3H, t, *J* 7.6), 2.67 (2H, q, *J* 7.6), 4.39 (1H, d, *J* 1.5), 4.87 (1H, d, *J* 1.5), 7.18 (2H, d, *J* 8.2), 7.55 (2H, d, *J* 8.2); ^{13}C{^{1}H} NMR (126 MHz, $CDCl_3$) δ_c: -4.5, 15.6, 18.5, 26.0, 28.7, 90.2, 125.4, 127.7, 135.4, 144.5, 156.2; HRMS (ASAP$^+$) calculated for $[C_{16}H_{27}OSi]^+$ $(M+H)^+$: m/z 263.1831, found 263.1839 (+3.0 ppm).

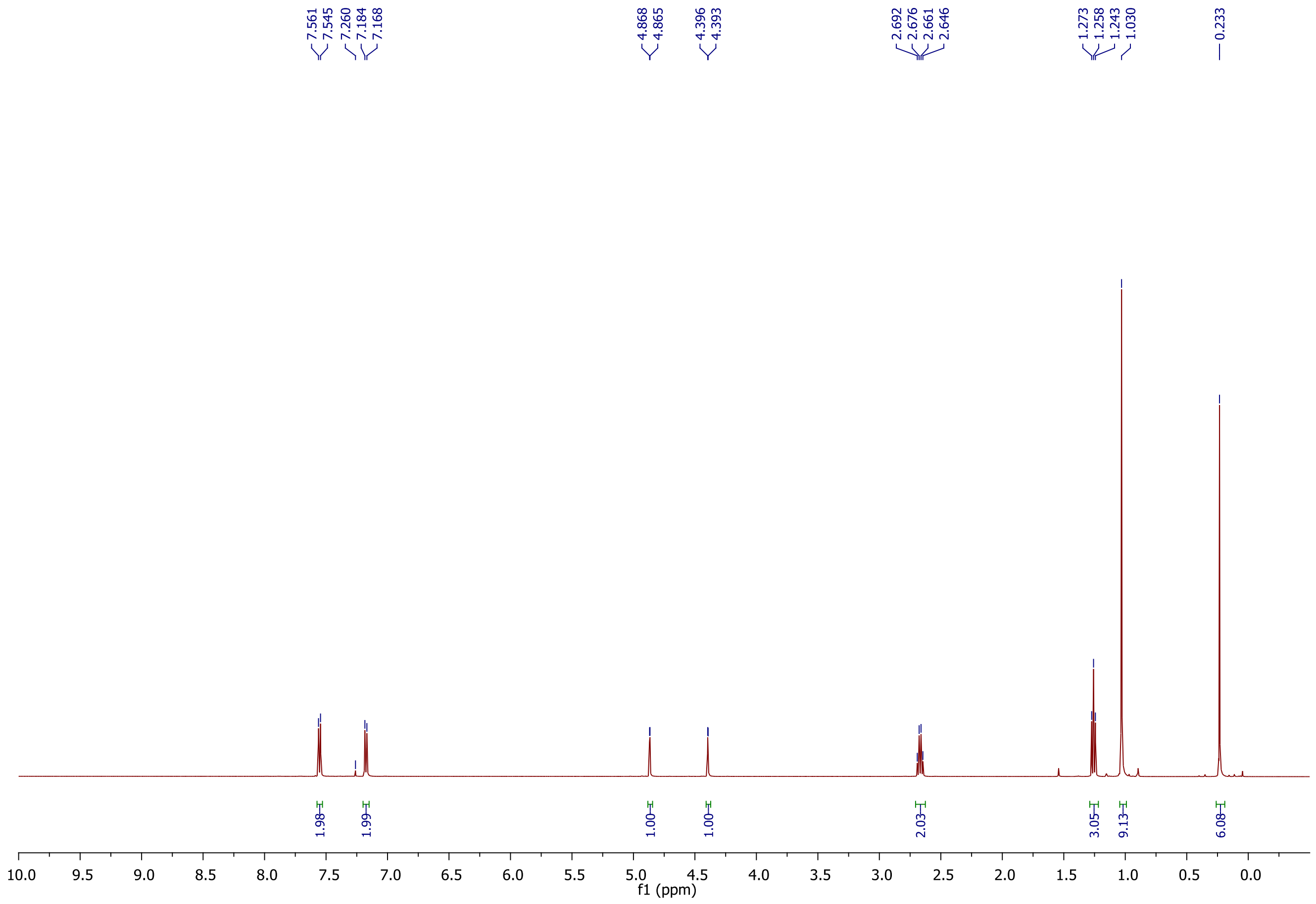

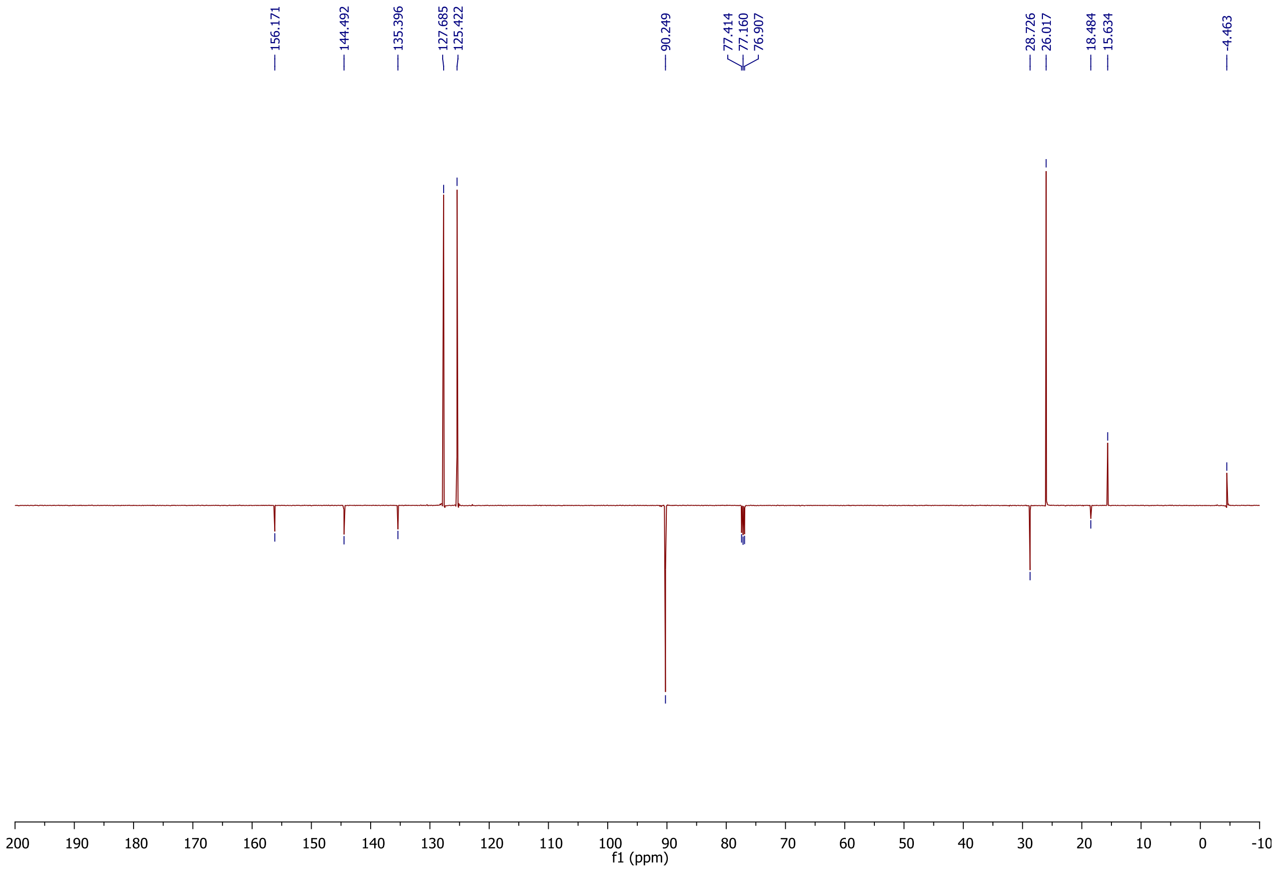

((1-([1,1'-Biphenyl]-4-yl)vinyl)oxy)(*tert*-butyl)dimethylsilane

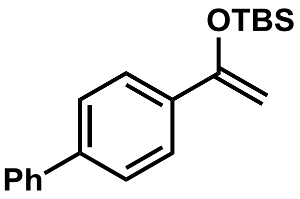

The title compound ((1-([1,1'-biphenyl]-4-yl)vinyl)oxy)(*tert*-butyl)dimethylsilane was prepared according to general procedure 2 from 4-phenylacetophenone (981 µL, 5.00 mmol, 1 equiv), triethylamine (836 µL, 6.00 mmol, 1.2 equiv), and TBSOTf (1.26 mL, 5.50 mmol, 1.1 equiv) and purified by flash silica column chromatography to give a white solid (1.41 g, 91% yield). mp 75-76 °C; R_f = 0.18 (eluent = 100% petroleum ether); ν_{max} / cm^{-1} (film) 2927, 2854, 1608, 1487, 1251, 1010, 833, 694; ^{1}H NMR (500 MHz, $CDCl_3$) δ_H: 0.27 (6H, s), 1.06 (9H, s), 4.48 (1H, d, *J* 1.8), 4.97 (1H, d, *J* 1.8), 7.36 (1H, t, *J* 7.4), 7.44-7.48 (2H, m), 7.58-7.60 (2H, m), 7.62-7.64 (2H, m), 7.70-7.72 (2H, m); $^{13}C\{^{1}H\}$ NMR (126 MHz, $CDCl_3$) δ_C: -4.5, 18.5, 26.0, 91.1, 125.8, 126.9, 127.1, 127.5, 128.9, 136.9, 140.9, 141.0, 155.8; HRMS (ASAP$^+$) calculated for $[C_{20}H_{26}OSi]^+$ $(M+H)^+$: m/z 311.1831, found 311.1837 (+1.9 ppm).

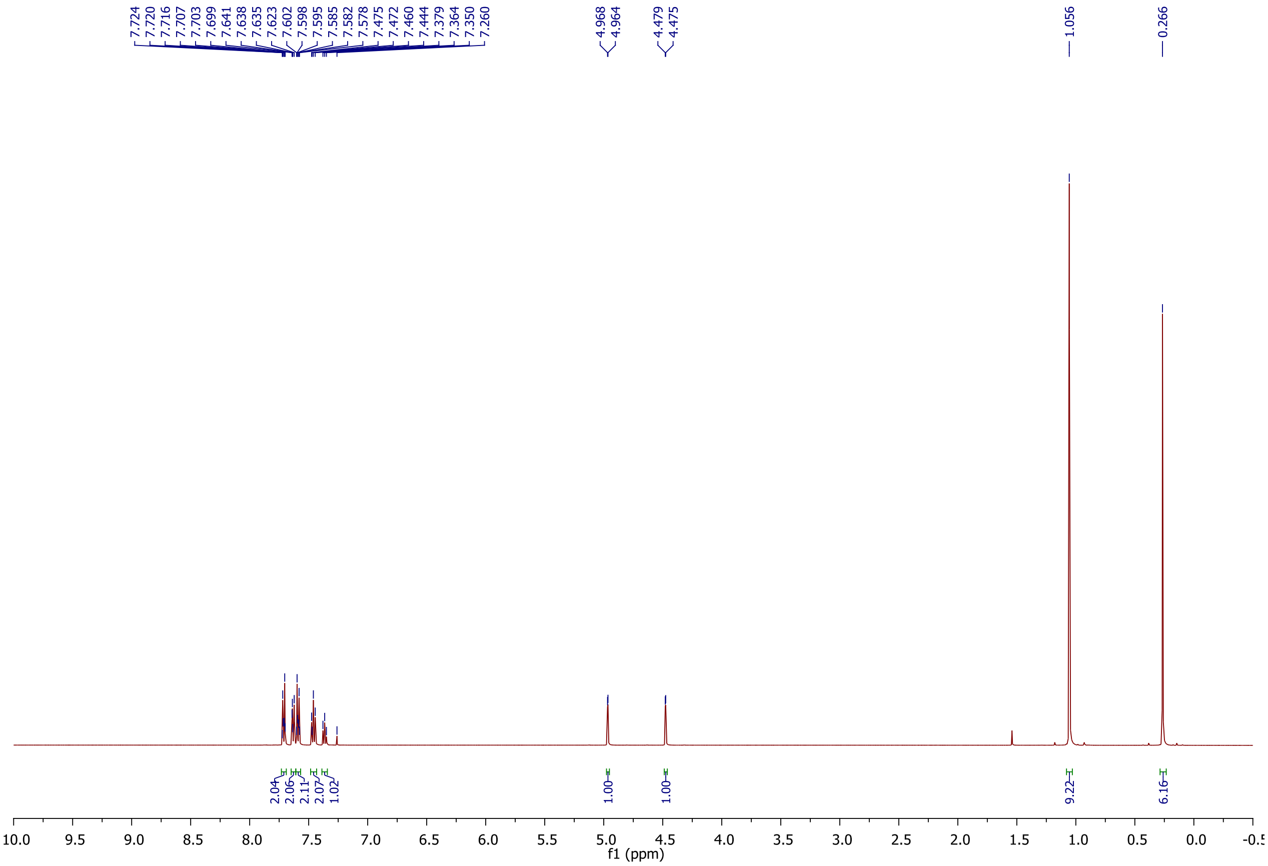
7.724
7.720
7.716
7.707
7.703
7.699
7.641
7.638
7.635
7.623
7.602
7.598
7.595
7.585
7.582
7.578
7.475
7.472
7.460
7.444
7.379
7.364
7.350
7.260
4.968
4.964
4.479
4.475
1.056
0.266
2.04
2.06
2.11
2.07
1.02
1.00
1.00
9.22
6.16
f1 (ppm)

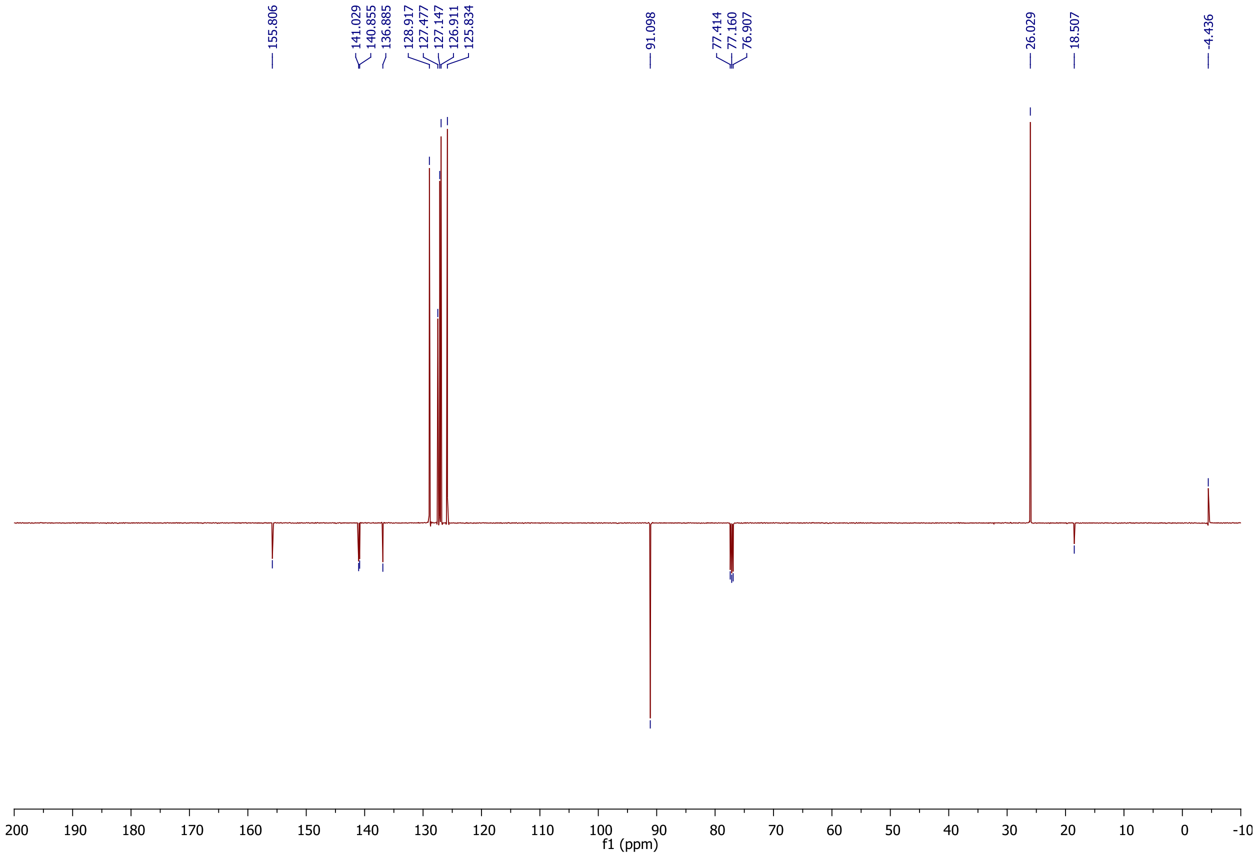
155.806
141.029
140.855
136.885
128.917
127.477
127.147
126.911
125.834
91.098
77.414
77.160
76.907
26.029
18.507
-4.436
f1 (ppm)

tert-Butyl((1-(4-methoxyphenyl)vinyl)oxy)dimethylsilane

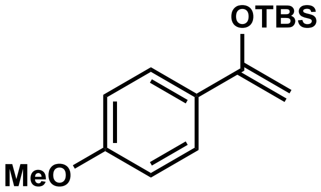

The title compound *tert*-butyl((1-(4-methoxyphenyl)vinyl)oxy)dimethylsilane was prepared according to general procedure 2 from 4′-methoxyacetophenone (1.50 g, 10.0 mmol, 1 equiv), triethylamine (1.67 mL, 12.0 mmol, 1.2 equiv), and TBSOTf (2.52 mL, 11.0 mmol, 1.1 equiv) and purified by flash silica column chromatography to give a colorless oil (2.50 g, 95% yield). R_f = 0.25 (eluent = 100% petroleum ether); ^{1}H NMR (500 MHz, $CDCl_3$) δ_H: 0.23 (6H, s), 1.02 (9H, s), 3.82 (3H, s), 4.35 (1H, d, *J* 1.6), 4.79 (1H, d, *J* 1.6), 6.85-6.88 (2H, m), 7.55-7.58 (2H, m); ^{13}C{^{1}H} NMR (126 MHz, $CDCl_3$) δ_c: -4.5, 18.5, 26.0, 55.4, 89.4, 113.5, 126.7, 130.6, 155.9, 159.8. Spectroscopic data in accordance with that stated in the literature.[7]

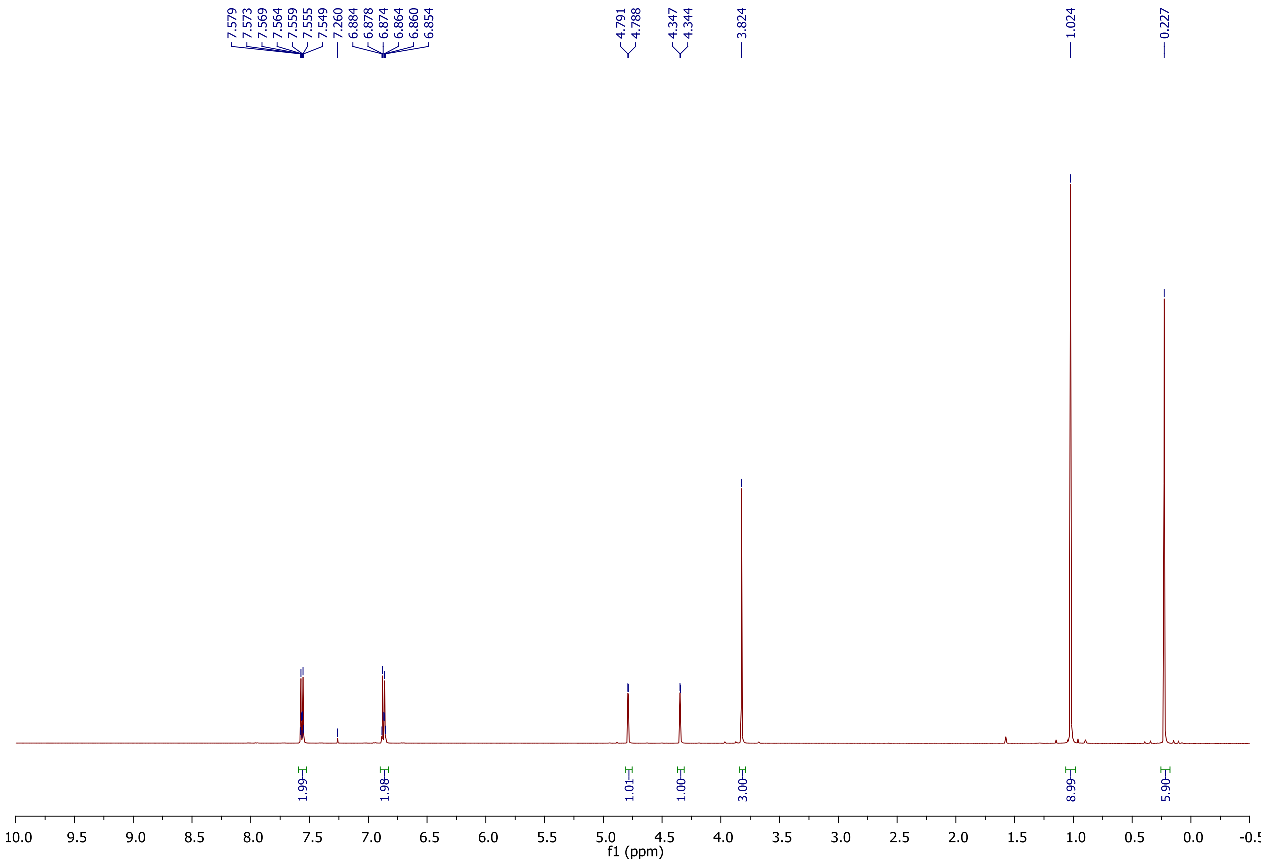

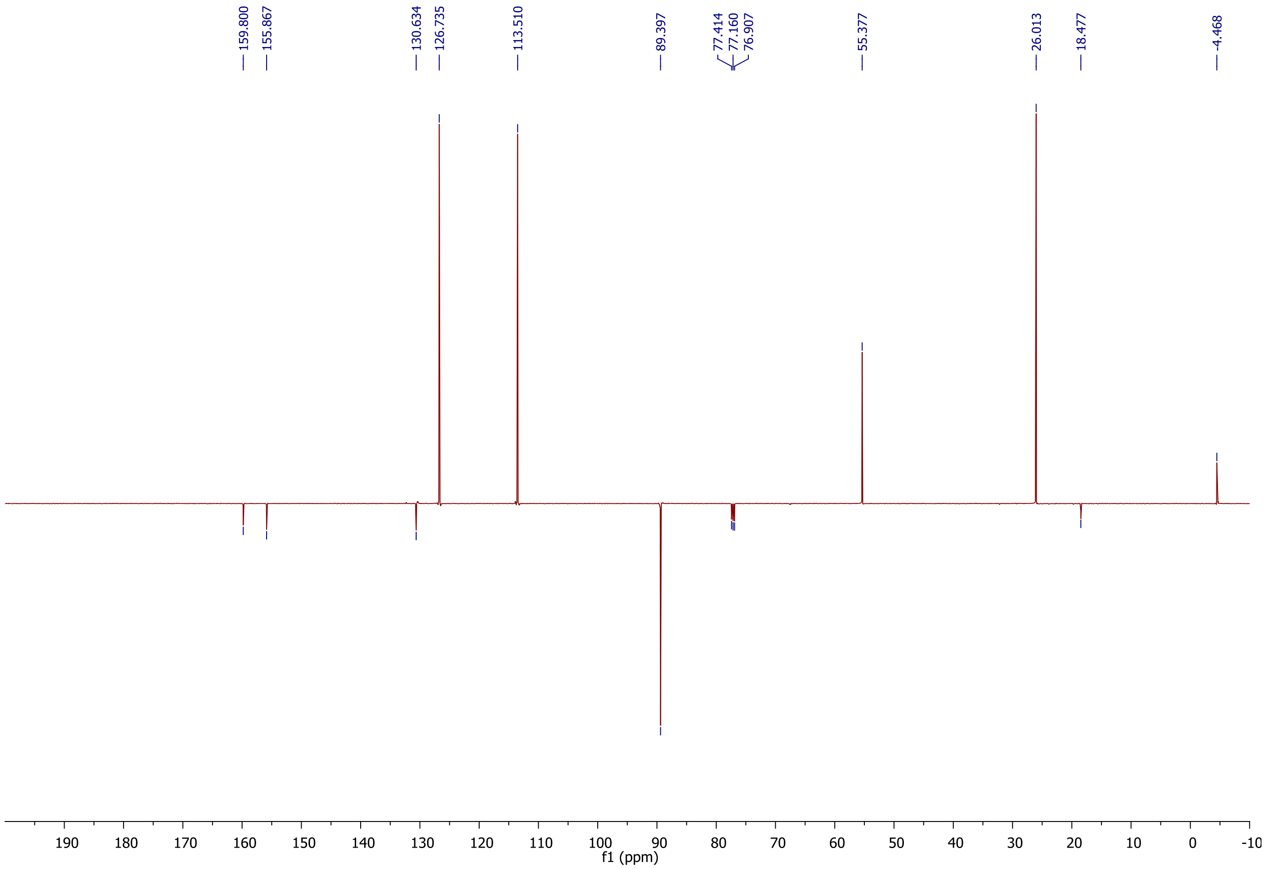

((1-(3-Methoxyphenyl)vinyl)oxy)trimethylsilane

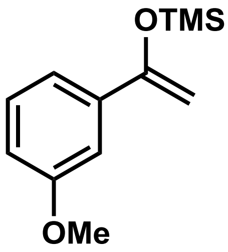

The title compound ((1-(3-methoxyphenyl)vinyl)oxy)trimethylsilane was prepared according to general procedure 1 from 3'-methoxyacetophenone (2.74 mL, 20.0 mmol, 1 equiv), triethylamine (4.18 mL, 30.0 mmol, 1.5 equiv), sodium iodide (3.60 g, 24.0 mmol, 1.2 equiv), and chlorotrimethylsilane (3.04 mL, 24.0 mmol, 1.2 equiv) and purified by Kugelrohr distillation to give a colorless oil (4.05 g, 91% yield). R_f = 0.27 (eluent = 100% petroleum ether); ν_{max} / cm^{-1} (film) 2958, 1653, 1575, 1489, 1251, 1008, 839, 752; ^{1}H NMR (500 MHz, $CDCl_3$) δ_H: 0.29 (9H, s), 3.83 (3H, s), 4.45 (1H, d, *J* 1.7), 4.93 (1H, d, *J* 1.7), 6.84-6.87 (1H, m), 7.15-7.16 (1H, m), 7.20-7.22 (1H, m), 7.23-7.27 (1H, m); ^{13}C{^{1}H} NMR (126 MHz, $CDCl_3$)

δ_C: 0.2, 55.3, 91.6, 111.1, 113.8, 118.0, 129.2, 139.2, 155.6, 159.6; HRMS (ASAP$^+$) calculated for $[C_{12}H_{19}O_2Si]^+$ $(M+H)^+$: m/z 223.1154, found 223.1158 (+1.8 ppm).

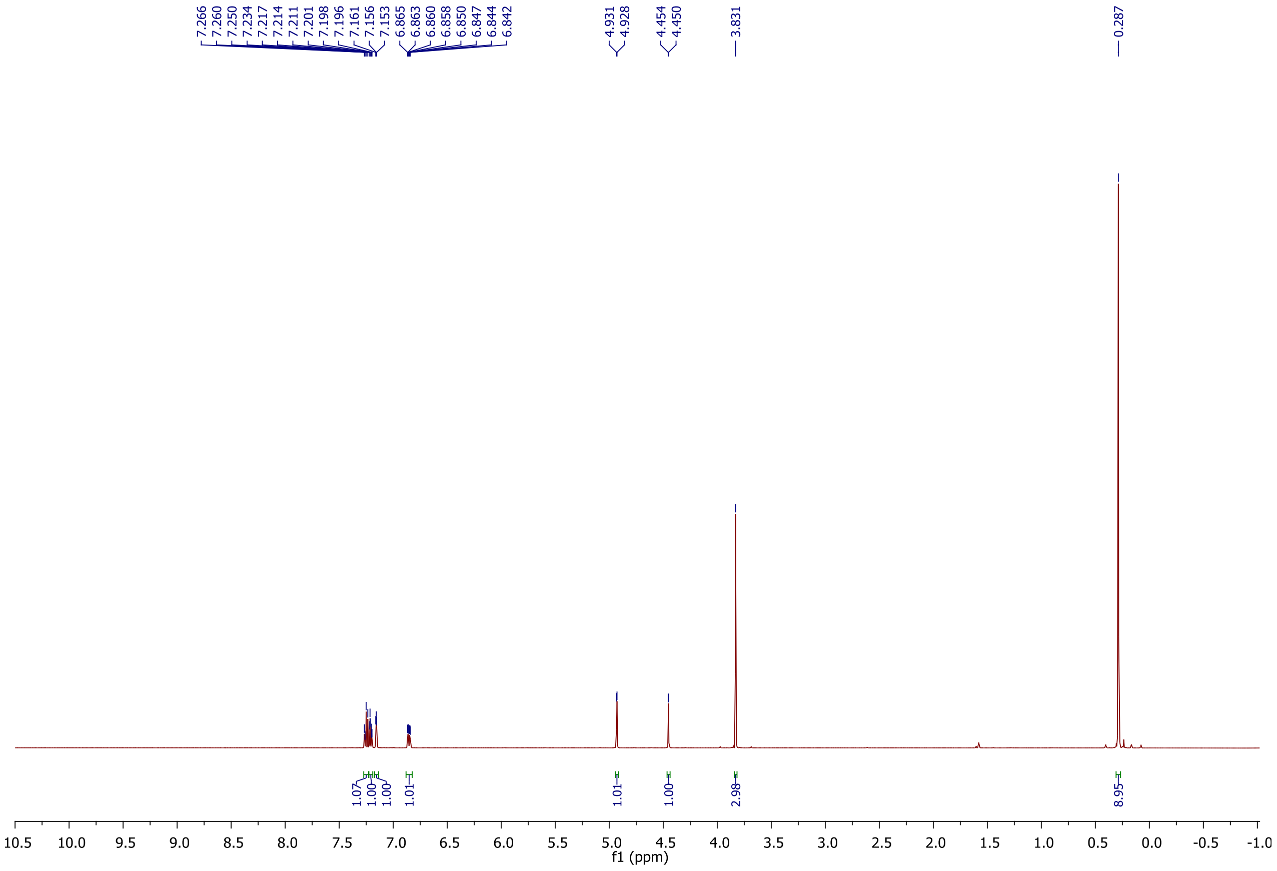

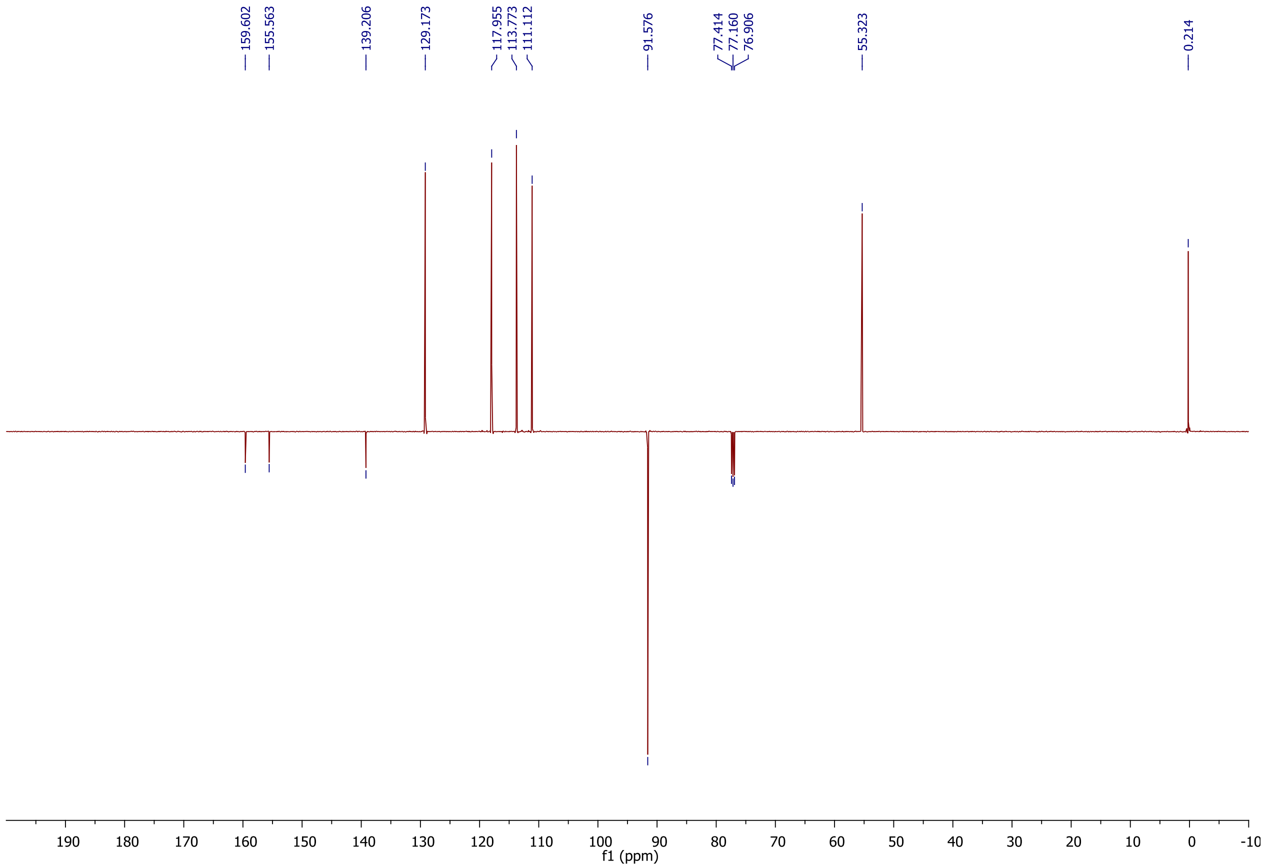

tert-Butyl((1-(2-methoxyphenyl)vinyl)oxy)dimethylsilane

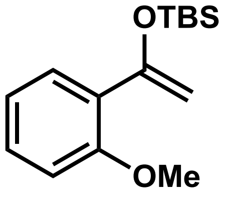

The title compound *tert*-butyl((1-(2-methoxyphenyl)vinyl)oxy)dimethylsilane was prepared according to general procedure 2 from 2′-methoxyacetophenone (1.03 mL, 7.50 mmol, 1 equiv), triethylamine (1.25 mL, 9.00 mmol, 1.2 equiv), and TBSOTf (1.89 mL, 8.25 mmol, 1.1 equiv) and purified by flash silica column chromatography to give a colorless oil (1.77 g, 89% yield). R_f = 0.21 (eluent = 100% petroleum ether); ν_{max} / cm^{-1} (film) 2956, 2929, 1610, 1506, 1489, 1242, 1014, 833, 748; ^1H NMR (500 MHz, $CDCl_3$) δ_H: 0.17 (6H, s), 0.98 (9H, s), 3.86 (3H, s), 4.67 (1H, d, *J* 0.5), 5.00 (1H, d, *J* 0.5), 6.90-6.96 (2H, m), 7.25-7.29 (1H, m), 7.54 (1H, dd, *J* 7.6, 1.8); $^{13}C\{^1H\}$ NMR (126 MHz, $CDCl_3$) δ_C: -4.5, 18.4, 25.9, 55.4, 96.5, 111.1, 120.2, 127.3, 129.2, 129.3, 153.5, 157.1; HRMS ($ASAP^+$) calculated for $[C_{15}H_{25}O_2Si]^+$ $(M+H)^+$: m/z 265.1624, found 265.1629 (+1.9 ppm).

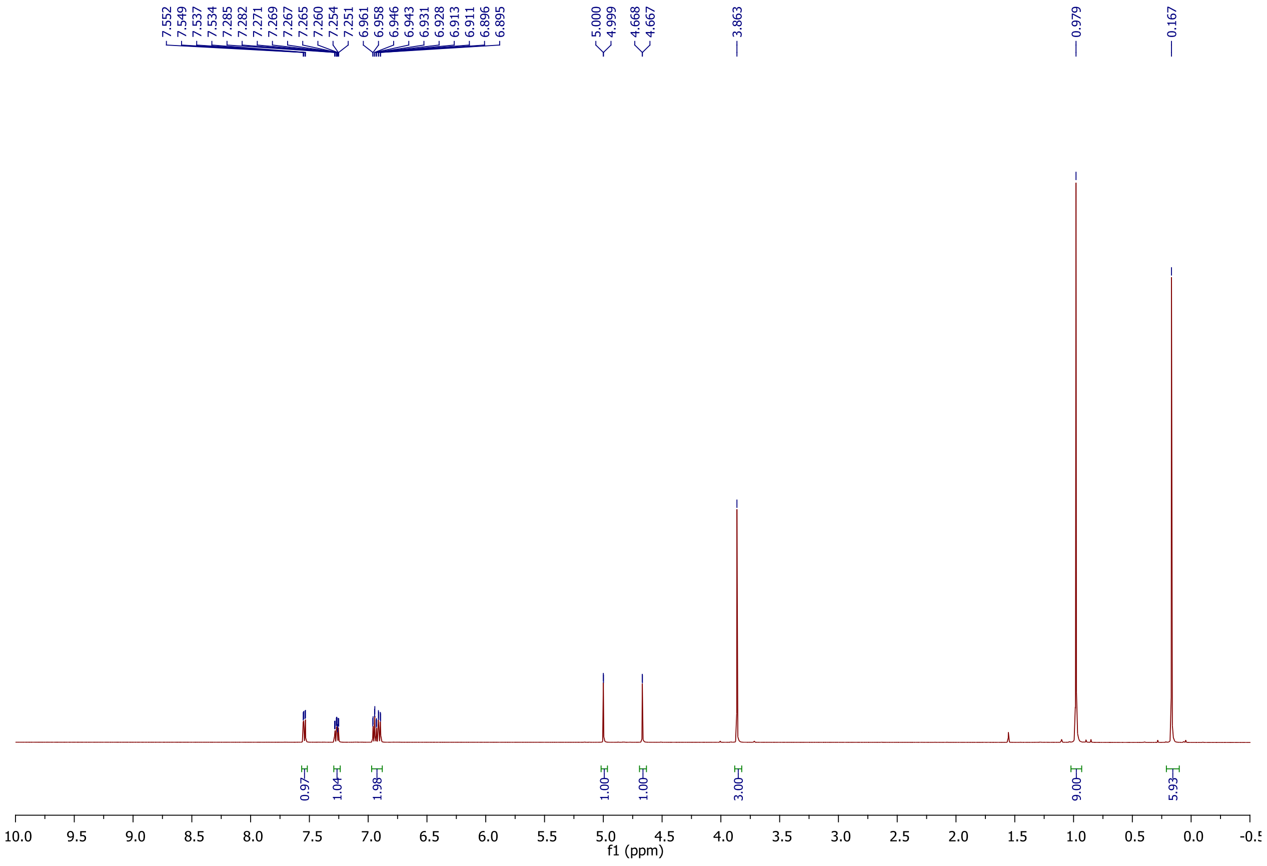
7.552
7.549
7.537
7.534
7.285
7.282
7.271
7.269
7.267
7.265
7.260
7.254
7.251
6.961
6.958
6.946
6.943
6.931
6.928
6.913
6.911
6.896
6.895
5.000
4.999
4.668
4.667
3.863
0.979
0.167
0.97
1.04
1.98
1.00
1.00
3.00
9.00
5.93
f1 (ppm)

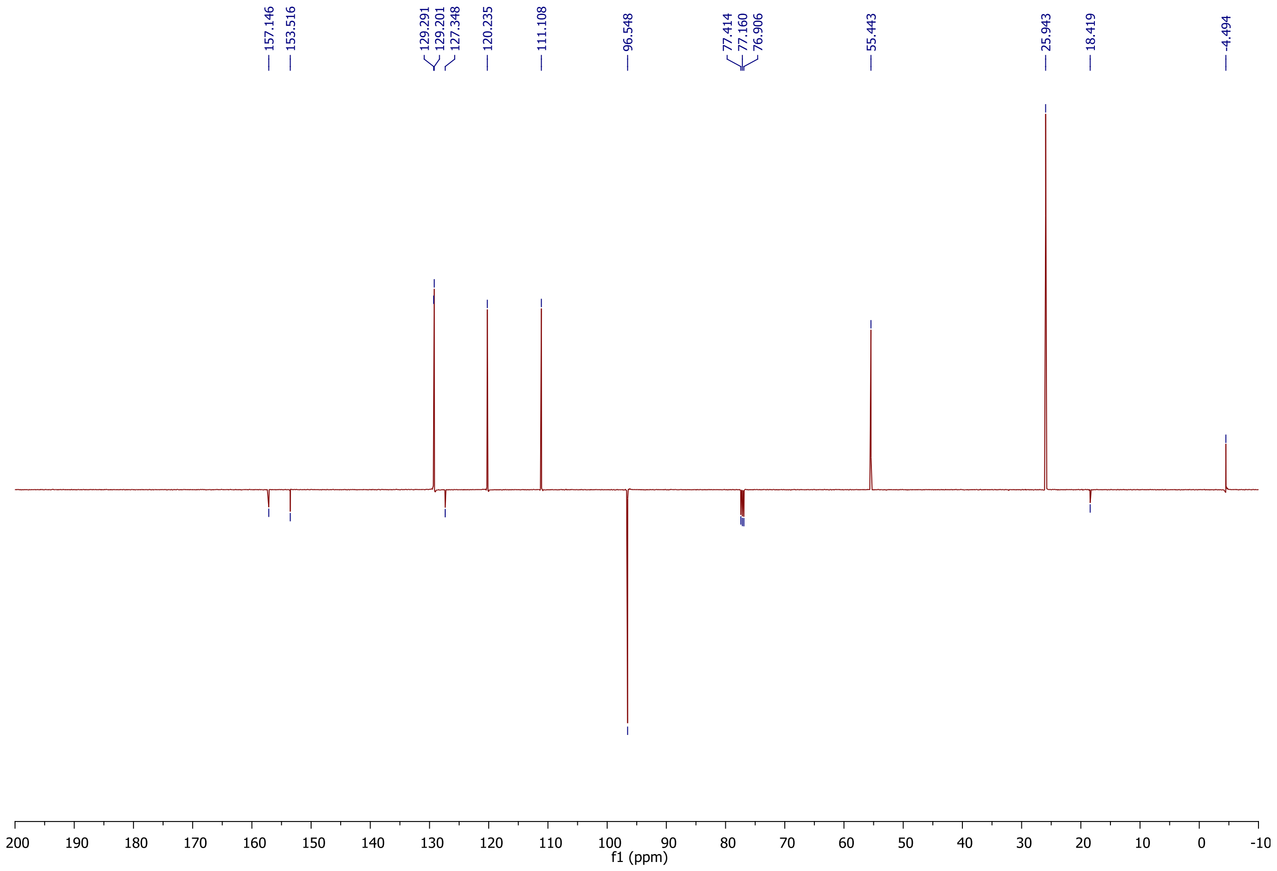
157.146
153.516
129.291
129.201
127.348
120.235
111.108
96.548
77.414
77.160
76.906
55.443
25.943
18.419
-4.494
f1 (ppm)

tert-Butyldimethyl((1-(4-(trifluoromethyl)phenyl)vinyl)oxy)silane

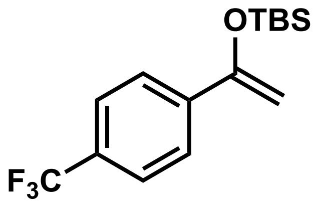

The title compound *tert*-butyldimethyl((1-(4-(trifluoromethyl)phenyl)vinyl)oxy)silane was prepared according to general procedure 2 from 4′-(trifluoromethyl)acetophenone (1.88 g, 10.0 mmol, 1 equiv), triethylamine (1.67 mL, 12.0 mmol, 1.2 equiv), and TBSOTf (2.52 mL, 11.0 mmol, 1.1 equiv) and purified by flash silica column chromatography to give a light yellow oil (2.60 g, 86% yield). R_f = 0.22 (eluent = 100% petroleum ether); ^{1}H NMR (500 MHz, $CDCl_3$) δ_H: 0.23 (6H, s), 1.01 (9H, s), 4.53 (1H, d, *J* 2.0), 4.98 (1H, d, *J* 2.0), 7.57-7.60 (2H, m), 7.70-7.73 (2H, m); ^{13}C{^{1}H} NMR (126 MHz, $CDCl_3$) δ_c: -4.5, 18.5, 25.9, 92.9, 124.3 (q, *J* 271.3), 125.2 (q, *J* 3.8), 125.6, 130.2 (q, *J* 32.4), 141.4 (q, *J* 1.2), 154.9; ^{19}F{^{1}H} NMR (470 MHz, $CDCl_3$) δ_F: -62.6. Spectroscopic data in accordance with that stated in the literature.[7]

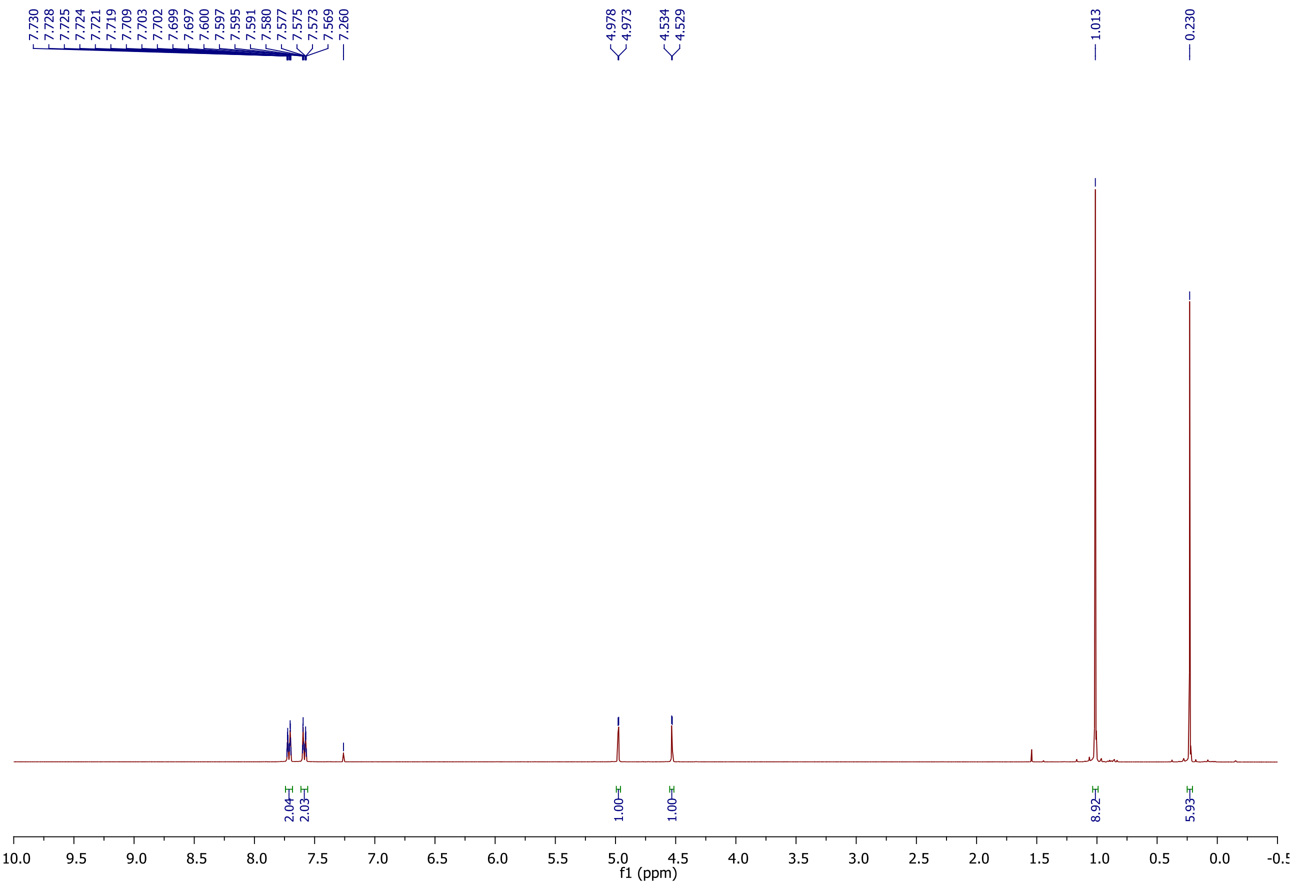

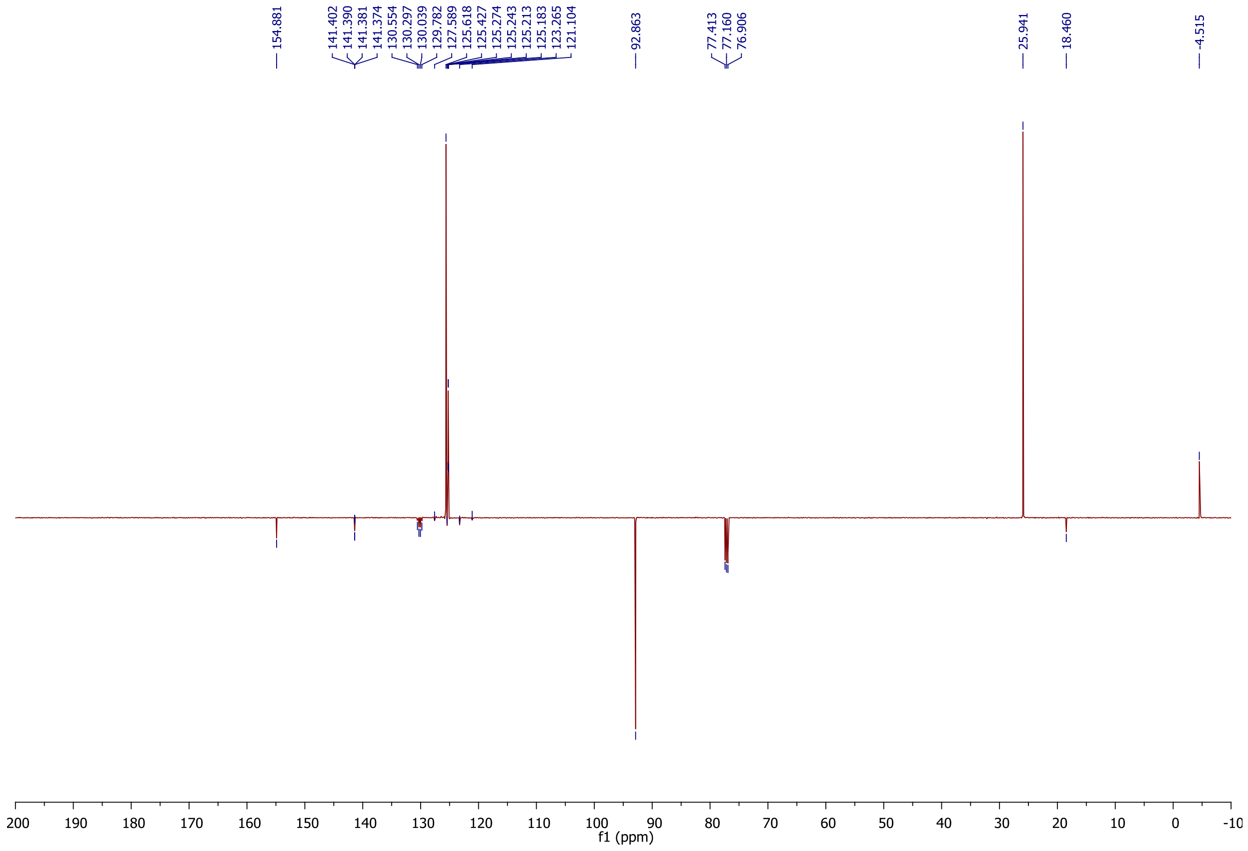
154.881
141.402
141.390
141.381
141.374
130.554
130.297
130.039
129.782
127.589
125.618
125.427
125.274
125.243
125.213
125.183
123.265
121.104
92.863
77.413
77.160
76.906
25.941
18.460
-4.515
f1 (ppm)

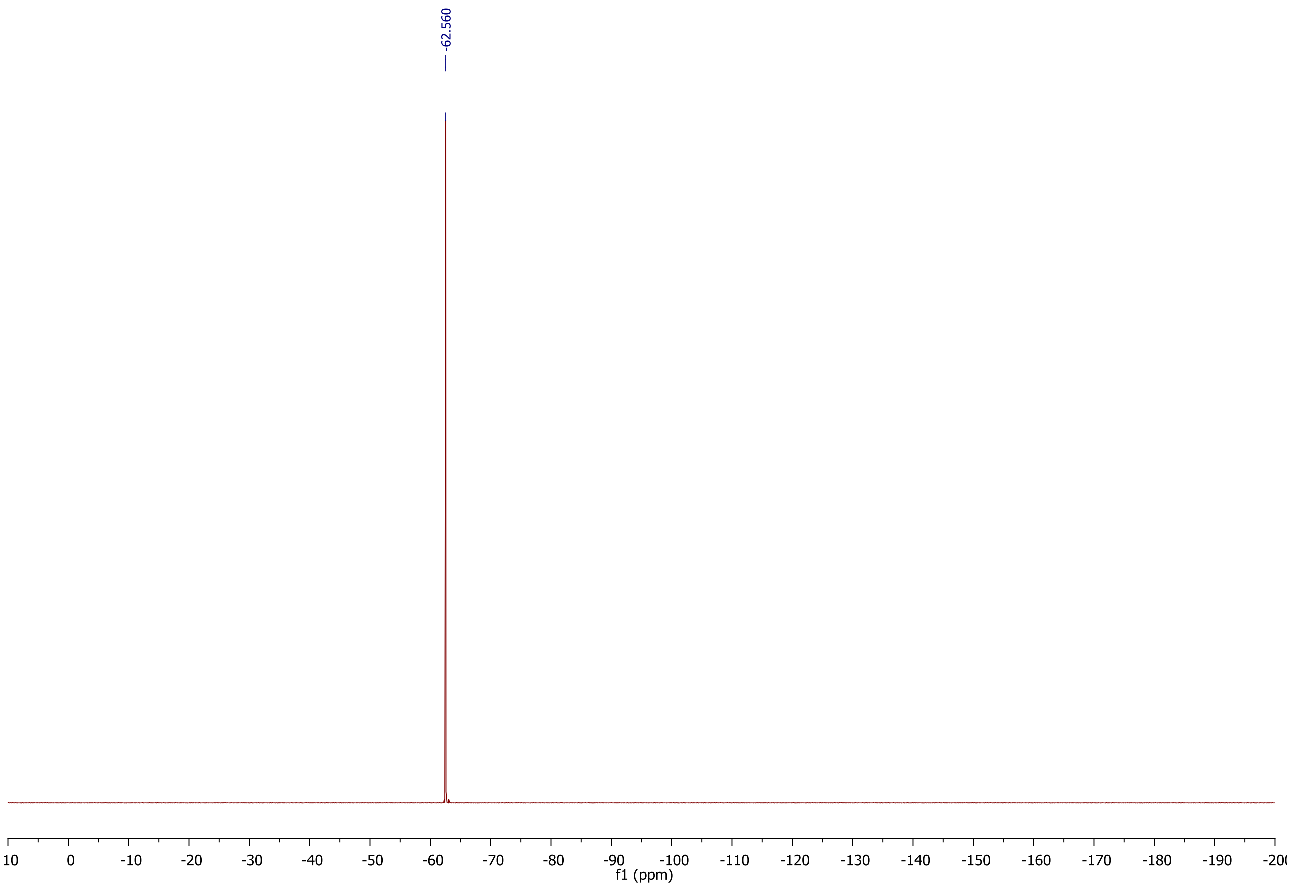
-62.560
f1 (ppm)

((1-(4-Fluorophenyl)vinyl)oxy)trimethylsilane

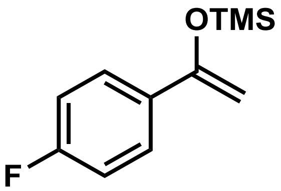

The title compound ((1-(4-fluorophenyl)vinyl)oxy)trimethylsilane was prepared according to general procedure 1 from 4'-fluoroacetophenone (2.43 mL, 20.0 mmol, 1 equiv), triethylamine (4.18 mL, 30.0 mmol, 1.5 equiv), sodium iodide (3.60 g, 24.0 mmol, 1.2 equiv), and chlorotrimethylsilane (3.04 mL, 24.0 mmol, 1.2 equiv) and purified by Kugelrohr distillation (bp 128-130 °C (10 mmHg)) {Lit.[10] 43-46 °C (0.5 mmHg)} to give a colorless oil (3.87 g, 92% yield). R_f = 0.26 (eluent = 100% petroleum ether); ν_{max} / cm^{-1} (film) 2960, 1618, 1506, 1251, 1008, 835, 750; ^{1}H NMR (500 MHz, $CDCl_3$) δ_H: 0.27 (9H, s), 4.40 (1H, d, *J* 1.8), 4.84 (1H, d, *J* 1.8), 6.97-7.03 (2H, m), 7.54-7.58 (2H, m); $^{13}C\{^1H\}$ NMR (126 MHz, $CDCl_3$) δ_c: 0.2, 90.8 (d, *J* 1.5), 115.1 (d, *J* 21.6), 127.1(d, *J* 8.1), 133.8 (d, *J* 3.2), 155.0, 163.0 (d, *J* 247.2); $^{19}F\{^1H\}$ NMR (470 MHz, $CDCl_3$) δ_F: -114.0; HRMS (ASAP$^+$) calculated for $[C_{11}H_{14}OFSi]^+$ $(M–H)^+$: m/z 209.0798, found 209.0804 (+2.9 ppm).

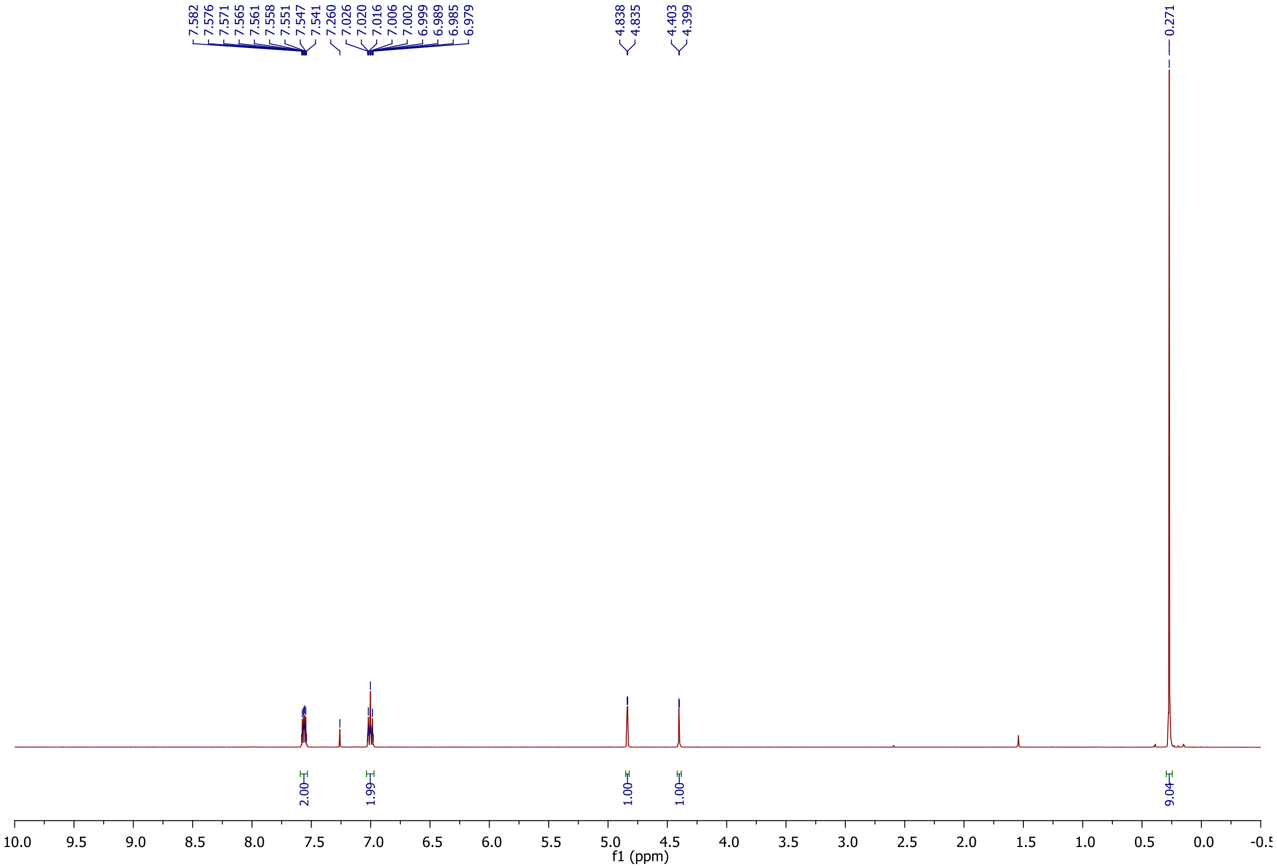

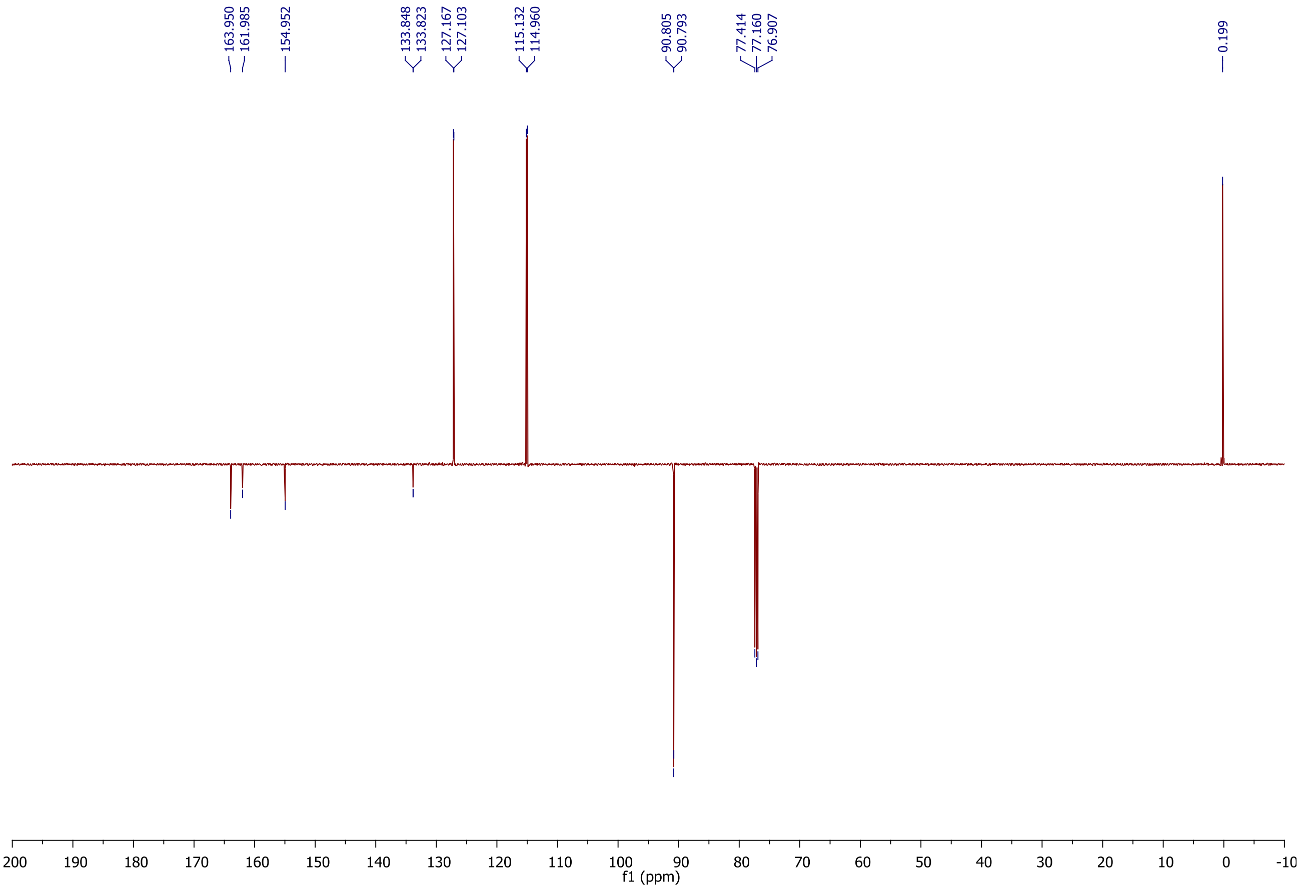
163.950
161.985
154.952
133.848
133.823
127.167
127.103
115.132
114.960
90.805
90.793
77.414
77.160
76.907
0.199
200 190 180 170 160 150 140 130 120 110 100 90 80 70 60 50 40 30 20 10 0 -10
f1 (ppm)

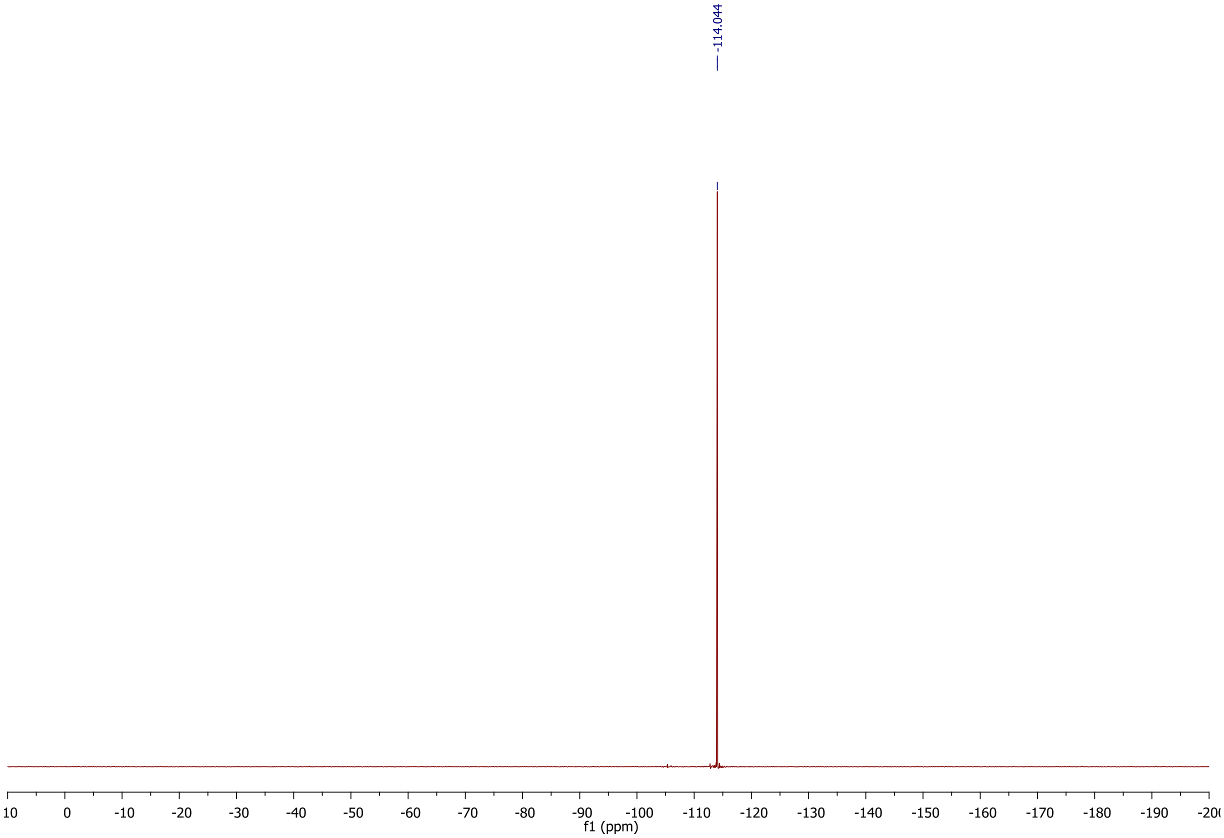
-114.044
10 0 -10 -20 -30 -40 -50 -60 -70 -80 -90 -100 -110 -120 -130 -140 -150 -160 -170 -180 -190 -200
f1 (ppm)

((1-(4-Chlorophenyl)vinyl)oxy)trimethylsilane

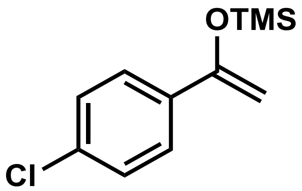

The title compound ((1-(4-chlorophenyl)vinyl)oxy)trimethylsilane was prepared according to general procedure 1 from 4'-chloroacetophenone (2.59 mL, 20.0 mmol, 1 equiv), triethylamine (4.18 mL, 30.0 mmol, 1.5 equiv), sodium iodide (3.60 g, 24.0 mmol, 1.2 equiv), and chlorotrimethylsilane (3.04 mL, 24.0 mmol, 1.2 equiv) and purified by Kugelrohr distillation (bp 130-132 °C (8 mmHg)) {Lit.[11] 98-102 °C (3.6 mmHg)} to give a colorless oil (4.06 g, 90% yield). R_f = 0.29 (eluent = 100% petroleum ether); ^{1}H NMR (500 MHz, $CDCl_3$) δ_H: 0.29 (9H, s), 4.46 (1H, d, *J* 1.9), 4.91 (1H, d, *J* 1.9), 7.28-7.32 (2H, m), 7.52-7.55 (2H, m); ^{13}C{^{1}H} NMR (126 MHz, $CDCl_3$) δ_C: 0.20, 91.5, 126.7, 128.4, 134.1, 136.2, 154.8. Spectroscopic data in accordance with that stated in the literature.[12]

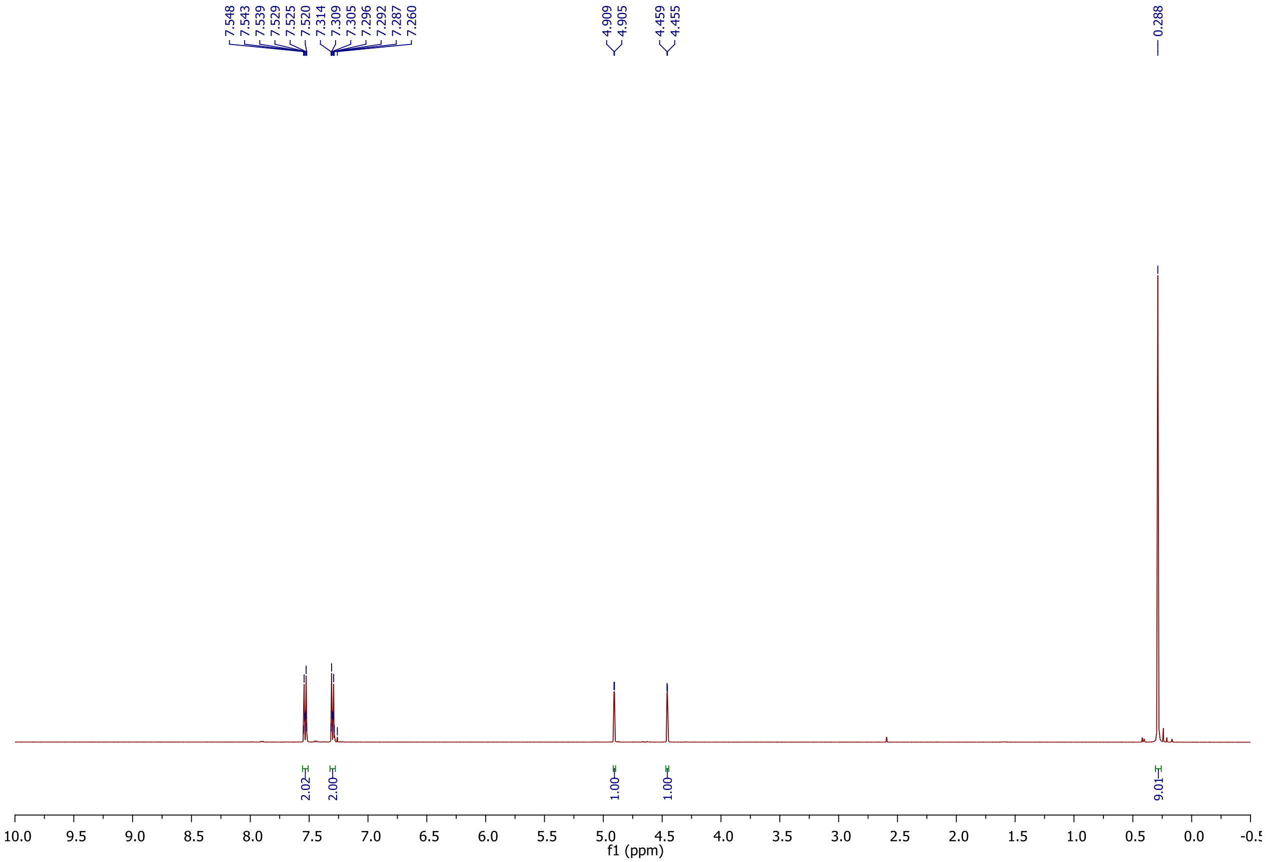

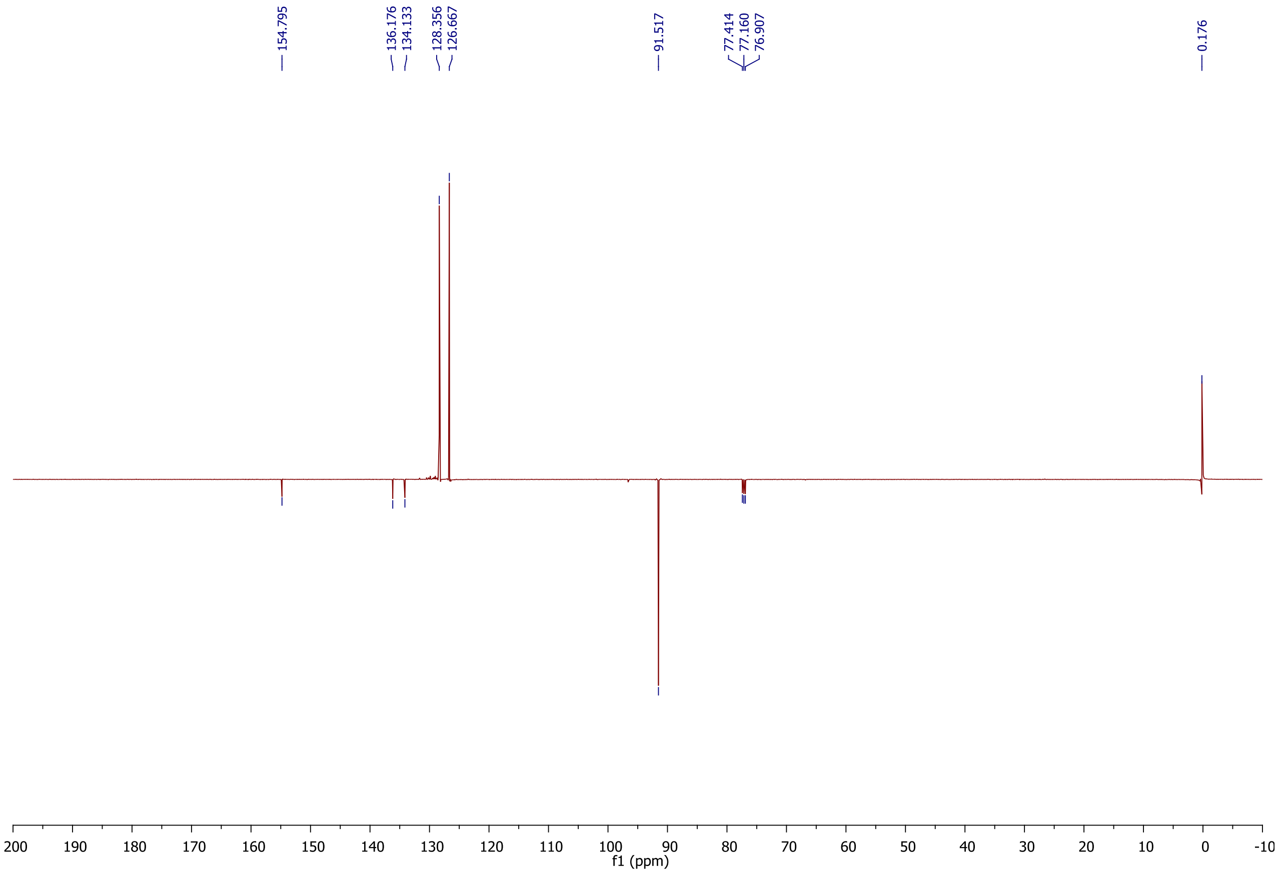

((1-(4-Bromophenyl)vinyl)oxy)trimethylsilane

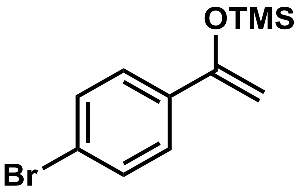

The title compound ((1-(4-bromophenyl)vinyl)oxy)trimethylsilane was prepared according to general procedure 1 from 4'-bromoacetophenone (1.99 mL, 10.0 mmol, 1 equiv), triethylamine (2.09 mL, 15.0 mmol, 1.5 equiv), sodium iodide (1.80 g, 12.0 mmol, 1.2 equiv), and chlorotrimethylsilane (1.52 mL, 12.0 mmol, 1.2 equiv) and purified by Kugelrohr distillation (bp 135-137 °C (12 mmHg)) {Lit.[13] 102 °C (2.25 mmHg)} to give a colorless oil (2.30 g, 85% yield). R_f = 0.37 (eluent = 100% petroleum ether); ν_{max} / cm^{-1} (film) 2958, 1614, 1485, 1251, 1006, 844; ^{1}H NMR (400 MHz, $CDCl_3$) δ_H: 0.29 (9H, s), 4.46 (1H, d, *J* 1.9), 4.92 (1H, d, *J* 1.9), 7.44-7.49 (4H, m); ^{13}C{^{1}H} NMR (101 MHz, $CDCl_3$) δ_c: 0.20, 91.6, 122.4, 127.0, 131.3, 136.6, 154.8. Spectroscopic data in accordance with that stated in the literature.[9]

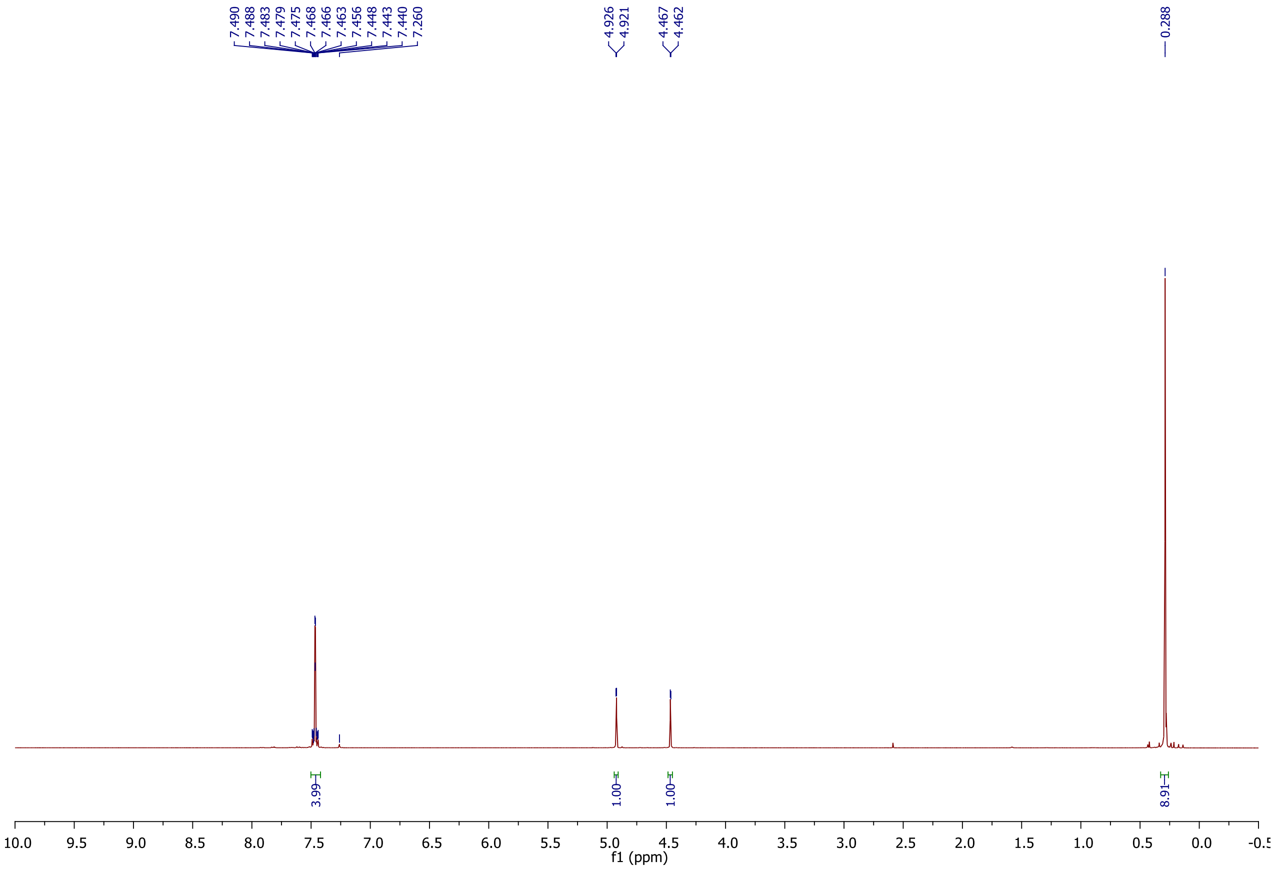
7.490
7.488
7.483
7.479
7.475
7.468
7.466
7.463
7.456
7.448
7.443
7.440
7.260
4.926
4.921
4.467
4.462
0.288
3.99
1.00
1.00
8.91
10.0 9.5 9.0 8.5 8.0 7.5 7.0 6.5 6.0 5.5 5.0 4.5 4.0 3.5 3.0 2.5 2.0 1.5 1.0 0.5 0.0
f1 (ppm)

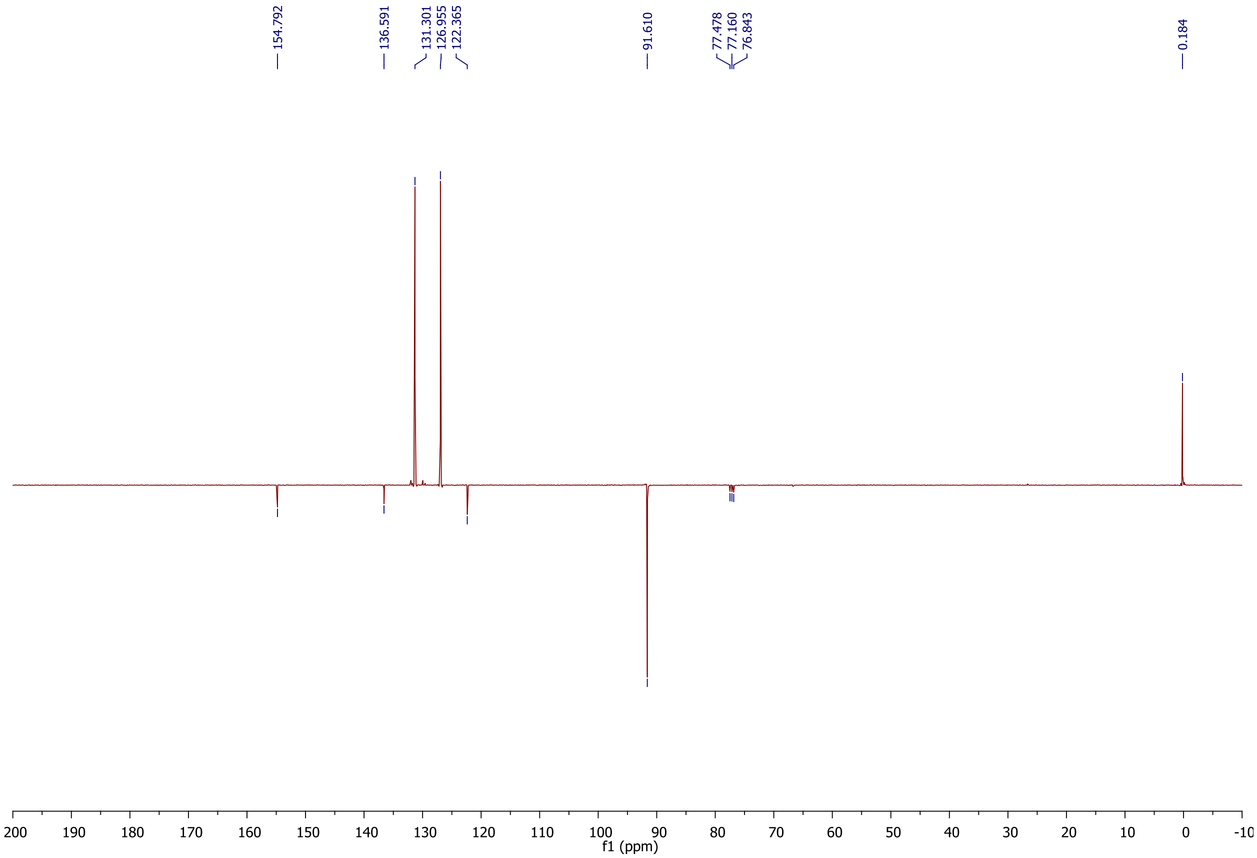
154.792
136.591
131.301
126.955
122.365
91.610
77.478
77.160
76.843
0.184
200 190 180 170 160 150 140 130 120 110 100 90 80 70 60 50 40 30 20 10 0 -10
f1 (ppm)

((1-(4-Iodophenyl)vinyl)oxy)trimethylsilane

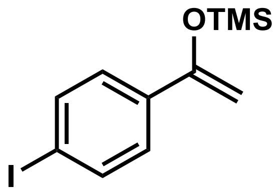

The title compound ((1-(4-iodophenyl)vinyl)oxy)trimethylsilane was prepared according to general procedure 1 from 4'-iodoacetophenone (1.23 g, 5.00 mmol, 1 equiv), triethylamine (1.04 mL, 7.50 mmol, 1.5 equiv), sodium iodide (899 mg, 6.00 mmol, 1.2 equiv), and chlorotrimethylsilane (760 µL, 6.00 mmol, 1.2 equiv) and purified by Kugelrohr distillation to give a colorless oil (1.41 g, 89% yield). R_f = 0.27 (eluent = 100% petroleum ether); ν_{max} / cm^{-1} (film) 2958, 1616, 1481, 1251, 1002, 840, 717; ^{1}H NMR (400 MHz, $CDCl_3$) δ_H: 0.27 (9H, s), 4.44 (1H, d, *J* 1.9), 4.92 (1H, d, *J* 1.9), 7.31-7.34 (2H, m), 7.63-7.67 (2H, m); $^{13}C\{^{1}H\}$ NMR (101 MHz, $CDCl_3$) δ_C: 0.2, 91.7, 94.1, 127.2, 137.2, 137.3, 154.9; HRMS ($ASAP^+$) calculated for $[C_{11}H_{16}IOSi]^+$ $(M+H)^+$: m/z 319.0015, found 319.0011 (-1.3 ppm).

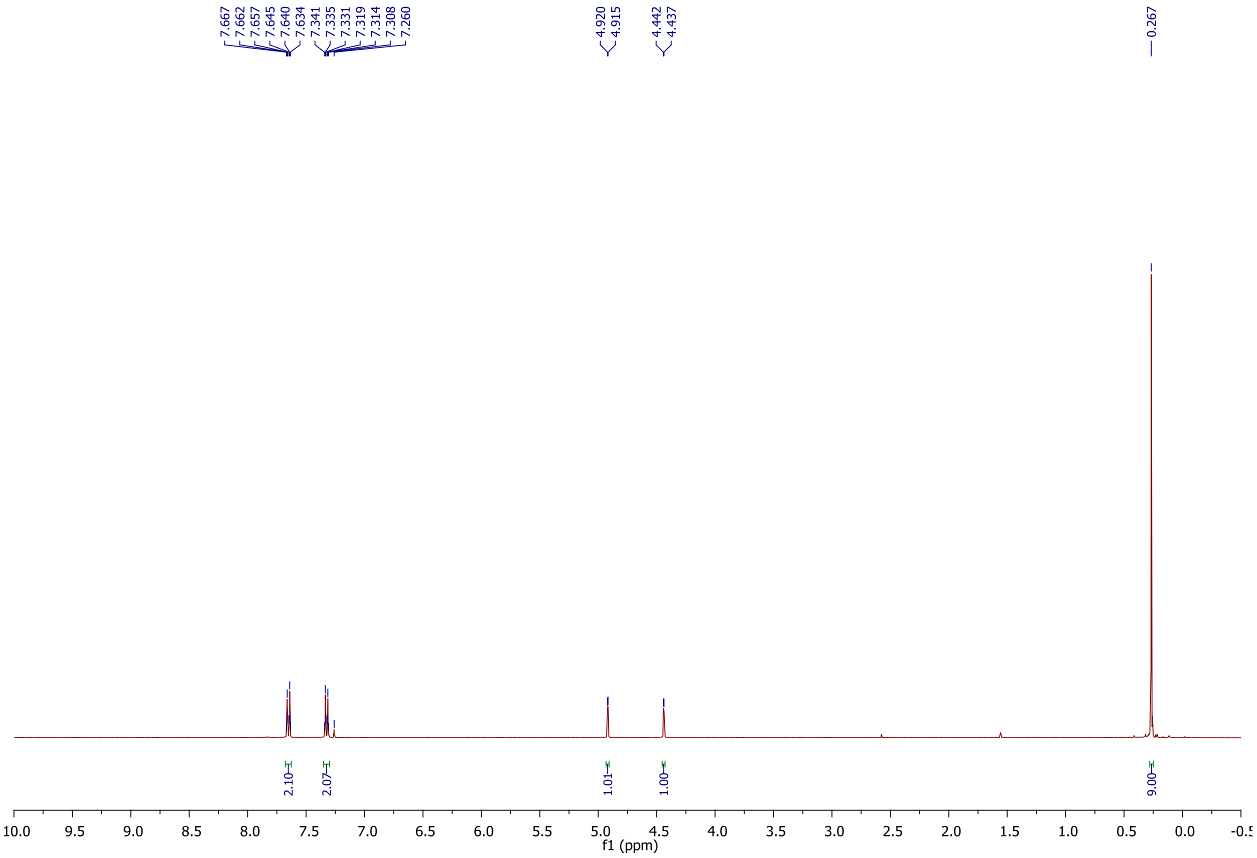

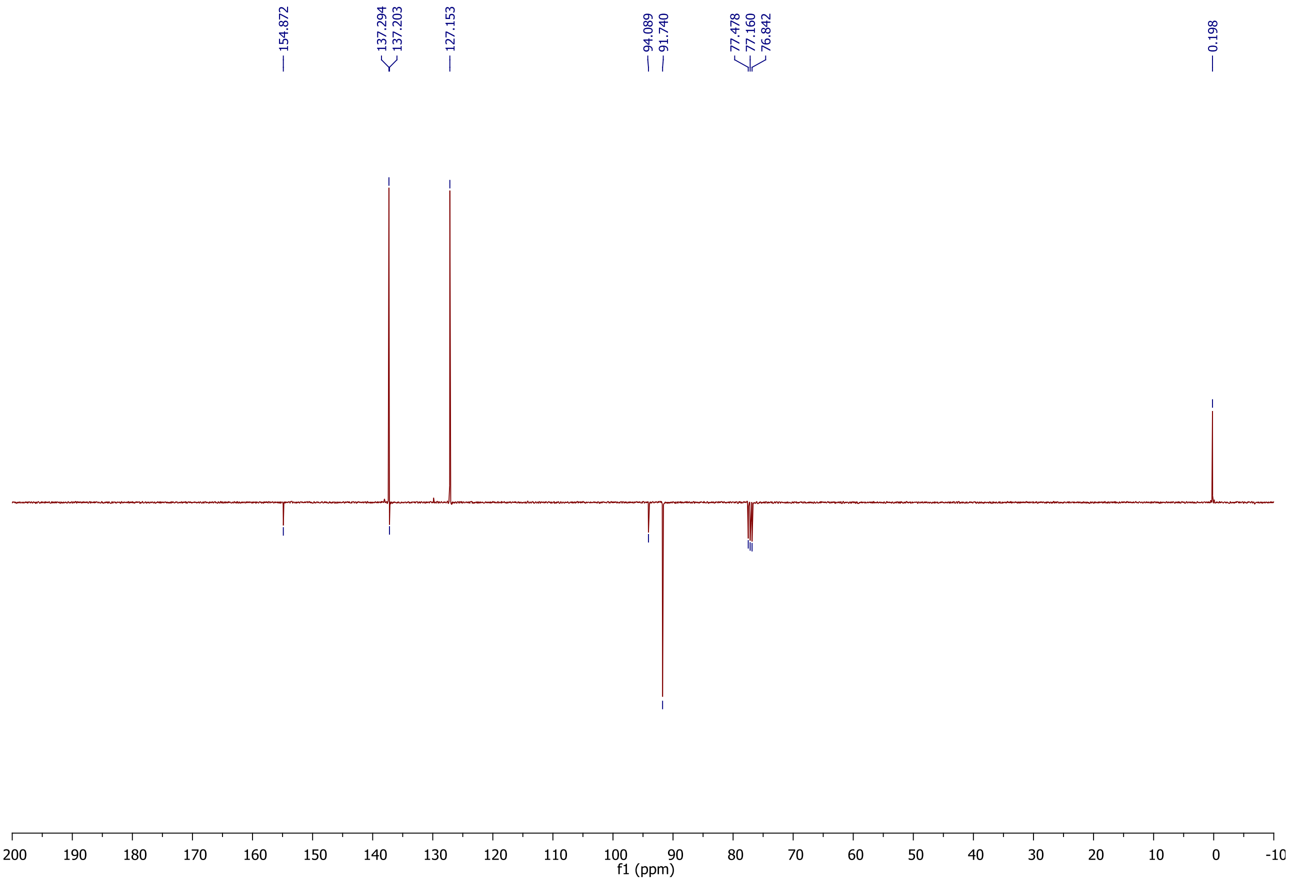

Trimethyl((1-(naphthalen-1-yl)vinyl)oxy)silane

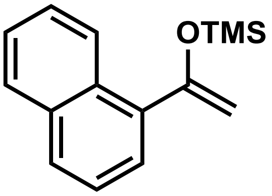

The title compound trimethyl((1-(naphthalen-1-yl)vinyl)oxy)silane was prepared according to general procedure 1 from 1-acetonaphthone (3.04 mL, 20.0 mmol, 1 equiv), triethylamine (4.18 mL, 30.0 mmol, 1.5 equiv), sodium iodide (3.60 g, 24.0 mmol, 1.2 equiv), and chlorotrimethylsilane (3.04 mL, 24.0 mmol, 1.2 equiv) and purified by Kugelrohr distillation to give a colorless oil (4.10 g, 85% yield). R_f = 0.22 (eluent = 100% petroleum ether); ν_{max} / cm^{-1} (film) 2956, 1558, 1506, 1251, 1012, 839, 777; ^1H NMR (500 MHz, $CDCl_3$) δ_H: 0.19 (9H, s), 4.67 (1H, d, *J* 0.9), 4.80 (1H, d, *J* 0.9), 7.44-7.47 (1H, m), 7.49-7.57 (3H, m), 7.83-7.88 (2H, m), 8.35-8.37 (1H, m); $^{13}C\{^1H\}$ NMR (126 MHz, $CDCl_3$) δ_C: 0.3, 96.8, 125.2, 125.8, 126.1, 126.3, 126.4, 128.3, 128.8, 131.1, 133.4, 137.4, 157.1; HRMS (ASAP$^+$) calculated for $[C_{15}H_{19}OSi]^+$ $(M+H)^+$: m/z 243.1205, found 243.1208 (+1.2 ppm).

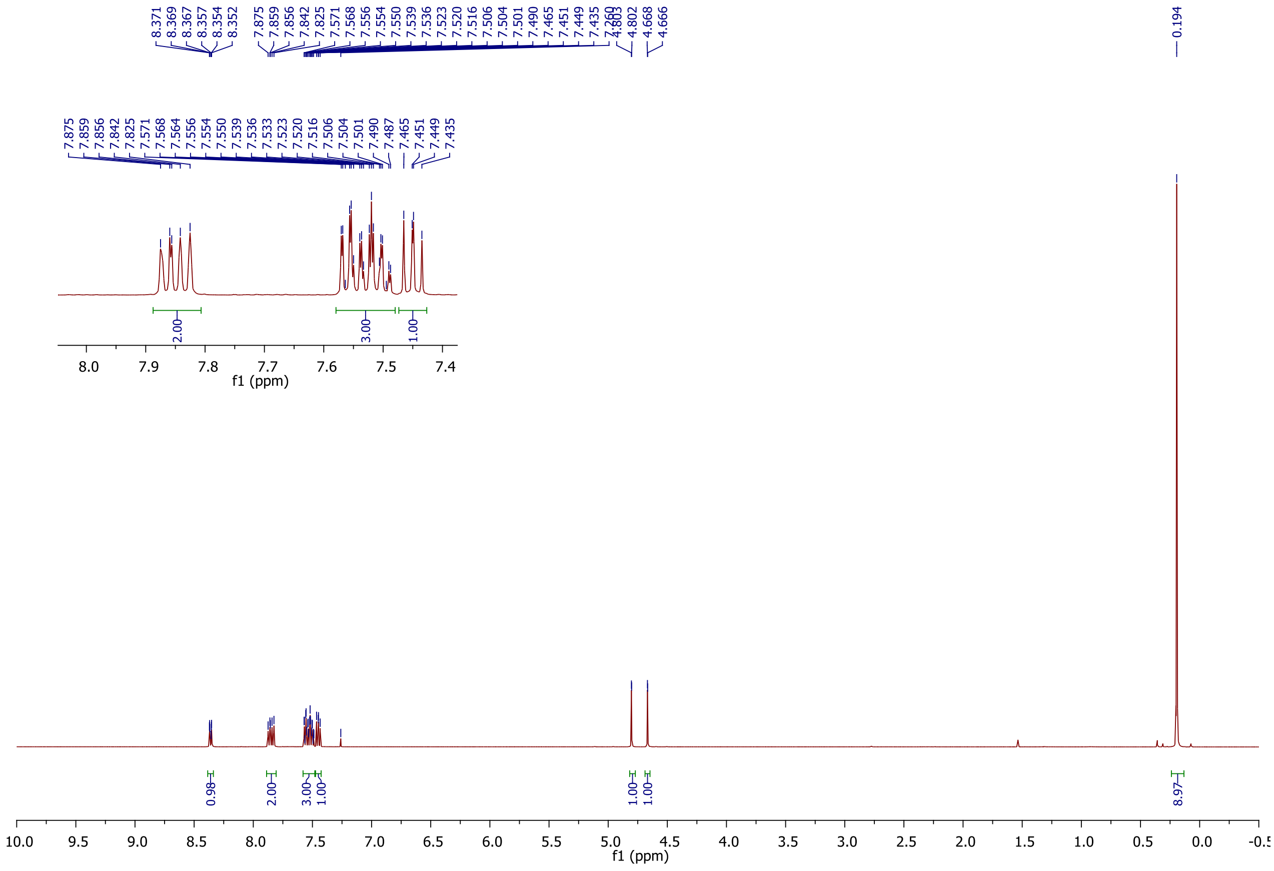

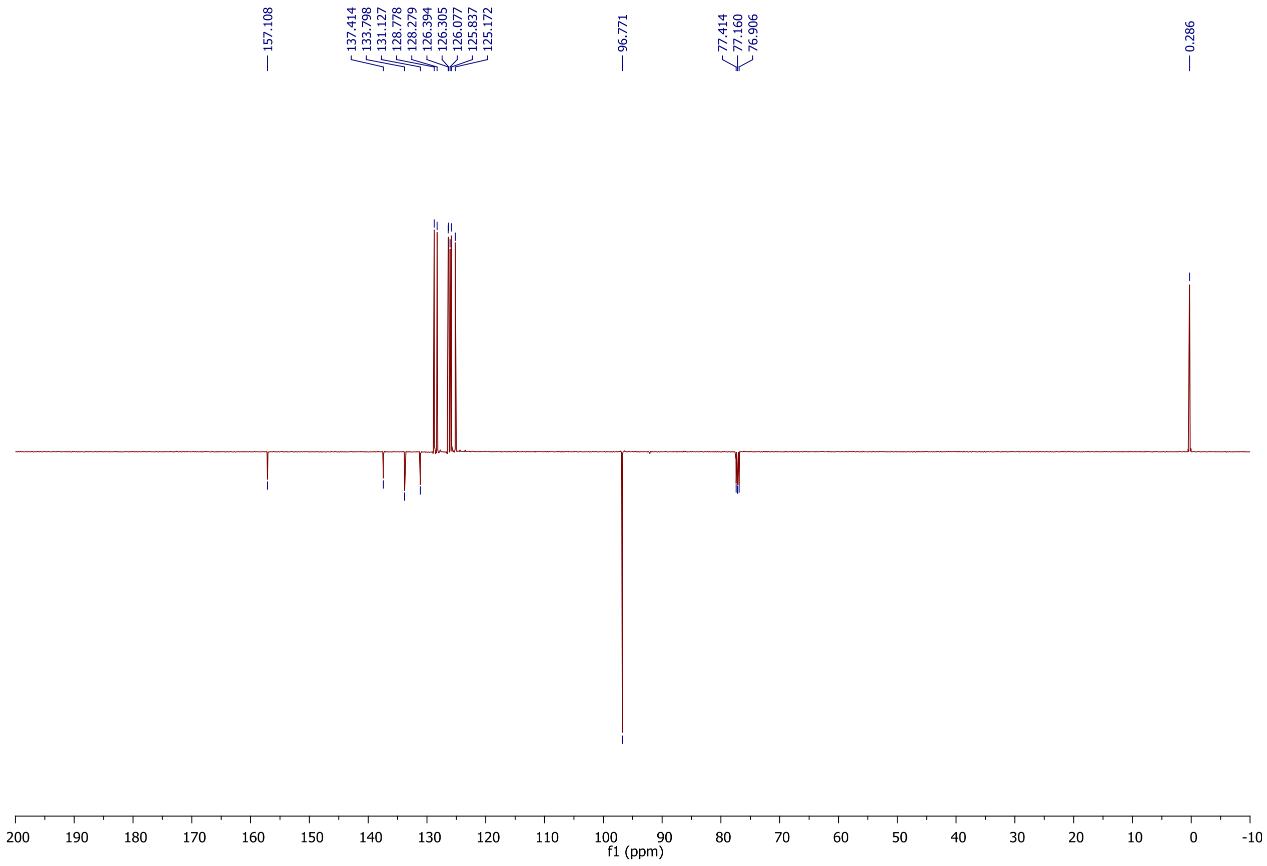

Trimethyl((1-(naphthalen-2-yl)vinyl)oxy)silane

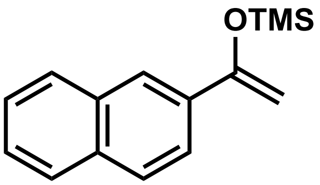

The title compound trimethyl((1-(naphthalen-2-yl)vinyl)oxy)silane was prepared according to general procedure 1 from 2-acetonaphthone (3.40 g, 20.0 mmol, 1 equiv), triethylamine (4.18 mL, 30.0 mmol, 1.5 equiv), sodium iodide (3.60 g, 24.0 mmol, 1.2 equiv), and chlorotrimethylsilane (3.04 mL, 24.0 mmol, 1.2 equiv) and purified by Kugelrohr distillation to give a colorless oil (4.03 g, 83% yield). R_f = 0.21 (eluent = 100% petroleum ether); ν_{max} / cm^{-1} (film) 2958, 1614, 1471, 1251, 1008, 839, 746; ^1H NMR (400 MHz, $CDCl_3$) δ_H: 0.44 (9H, s), 4.68 (1H, d, *J* 1.8), 5.20 (1H, d, *J* 1.8), 7.52-7.59 (2H, m), 7.81-7.83 (1H, m), 7.86-7.92 (2H, m), 7.95-7.99 (1H, m), 8.20 (1H, d, *J* 0.9); $^{13}C\{^1H\}$ NMR (101 MHz, $CDCl_3$) δ_c: 0.3, 92.0, 123.5, 124.4, 126.2, 126.3, 127.7, 127.8, 128.6, 133.3, 133.4, 134.9, 155.7; HRMS ($ASAP^+$) calculated for $[C_{15}H_{19}OSi]^+$ $(M+H)^+$: m/z 243.1205, found 243.1210 (+2.1 ppm).

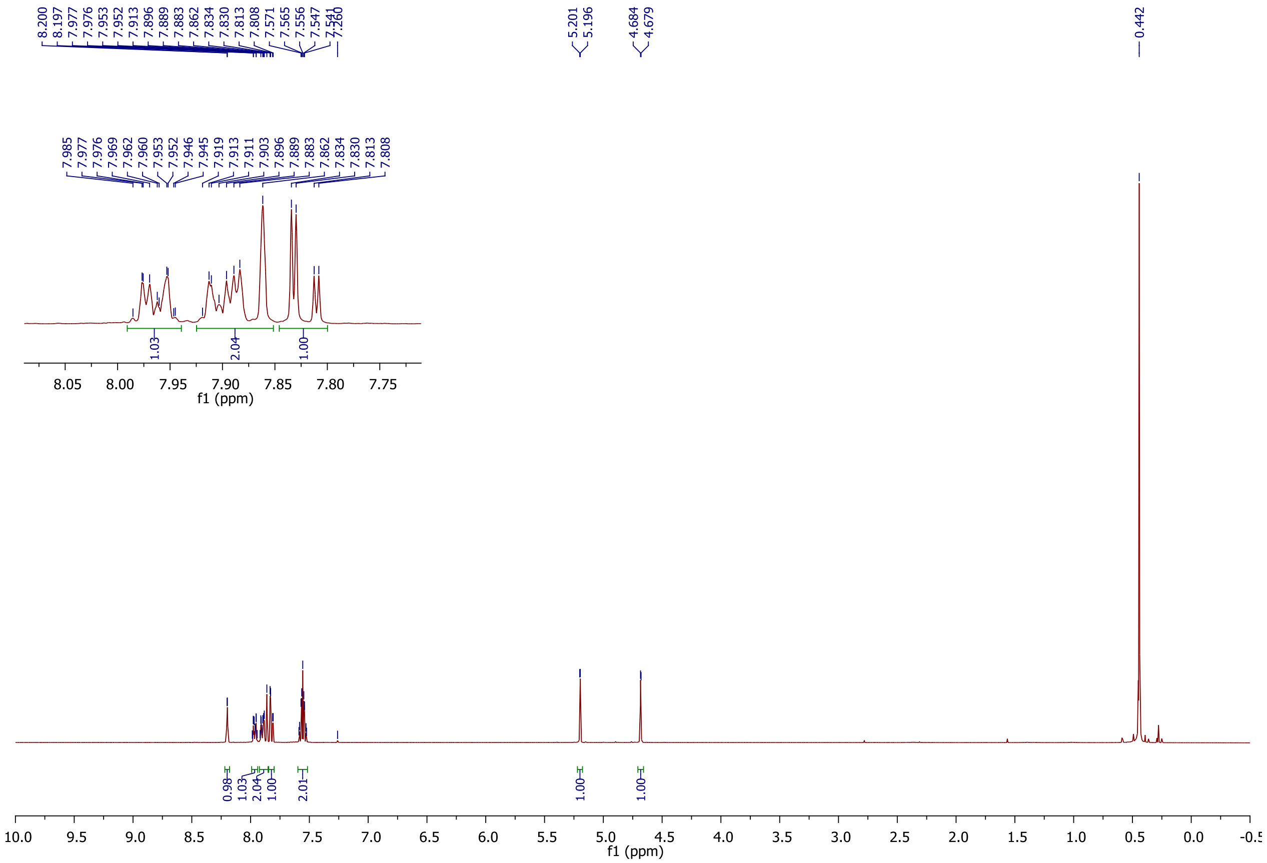

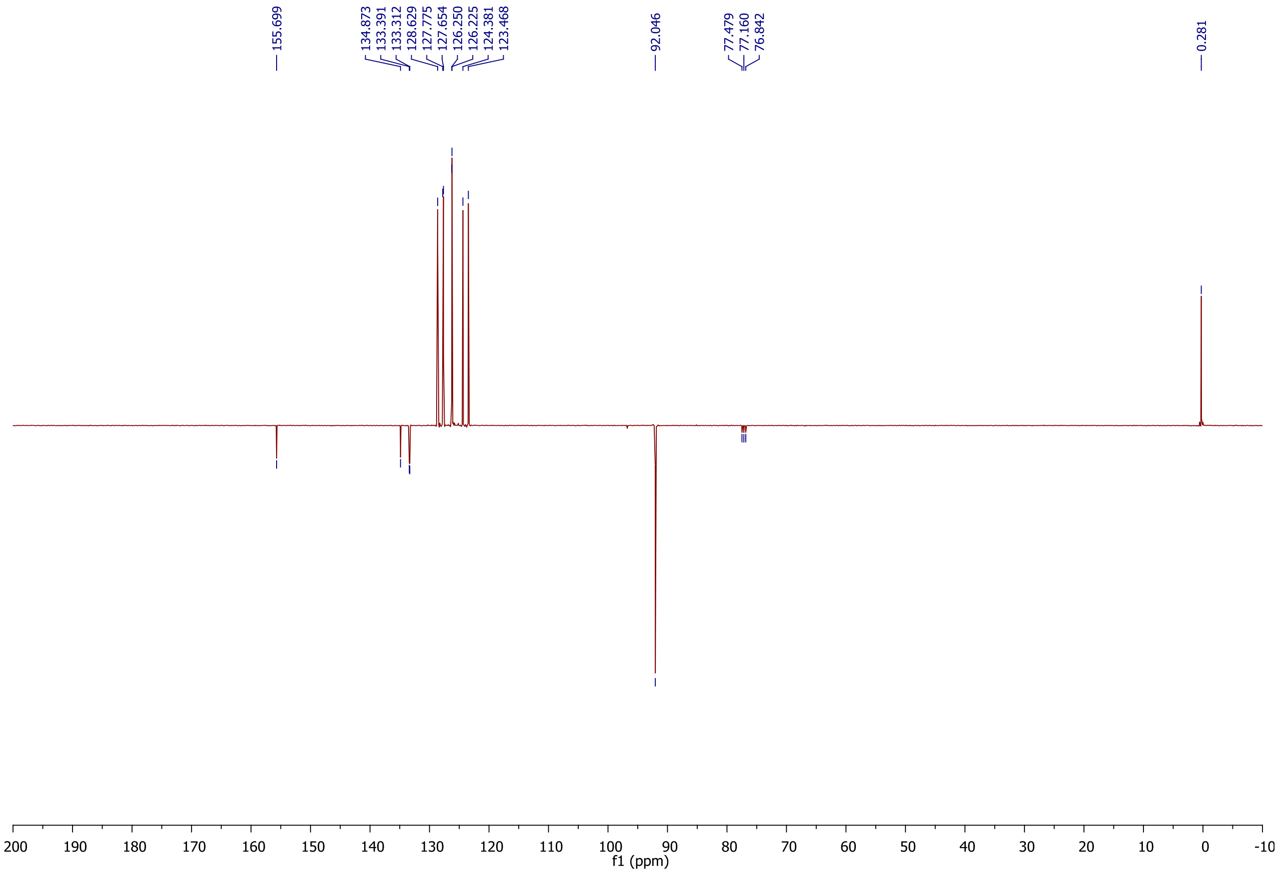

Trimethyl((1-(phenanthren-9-yl)vinyl)oxy)silane

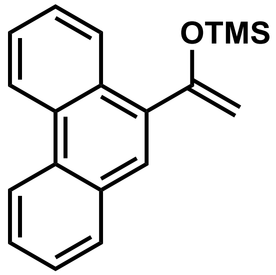

The title compound trimethyl((1-(phenanthren-9-yl)vinyl)oxy)silane was prepared according to general procedure 1 from 9-acetylphenanthrene (660 mg, 3.00 mmol, 1 equiv), triethylamine (630 µL, 4.50 mmol, 1.5 equiv), sodium iodide (540 mg, 3.60 mmol, 1.2 equiv), and chlorotrimethylsilane (460 µL, 3.60 mmol, 1.2 equiv) and purified by Kugelrohr distillation to give a light yellow oil (720 mg, 82% yield). R_f = 0.19 (eluent = 100% petroleum ether); ν_{max} / cm^{-1} (film) 2958, 1616, 1448, 1249, 1008, 839, 725; ^{1}H NMR (500 MHz, $CDCl_3$) δ_H: 0.18 (9H, s), 4.73 (1H, d, *J* 0.8), 4.80 (1H, d, *J* 0.8), 7.59-7.69 (4H, m), 7.80 (1H, s), 7.89-7.91 (1H, m), 8.35-8.37 (1H, m), 8.67-8.72 (2H, m); $^{13}C\{^1H\}$ NMR (126 MHz, $CDCl_3$) δ_c: 0.3, 96.8, 122.7, 122.8, 126.6, 126.6, 126.8, 127.0, 127.2, 127.3, 128.9, 130.3, 130.6, 130.6, 131.4, 136.2,

157.6; HRMS (ASAP$^+$) calculated for $[C_{19}H_{21}OSi]^+$ $(M+H)^+$: m/z 293.1362, found 293.1362 (0.0 ppm).

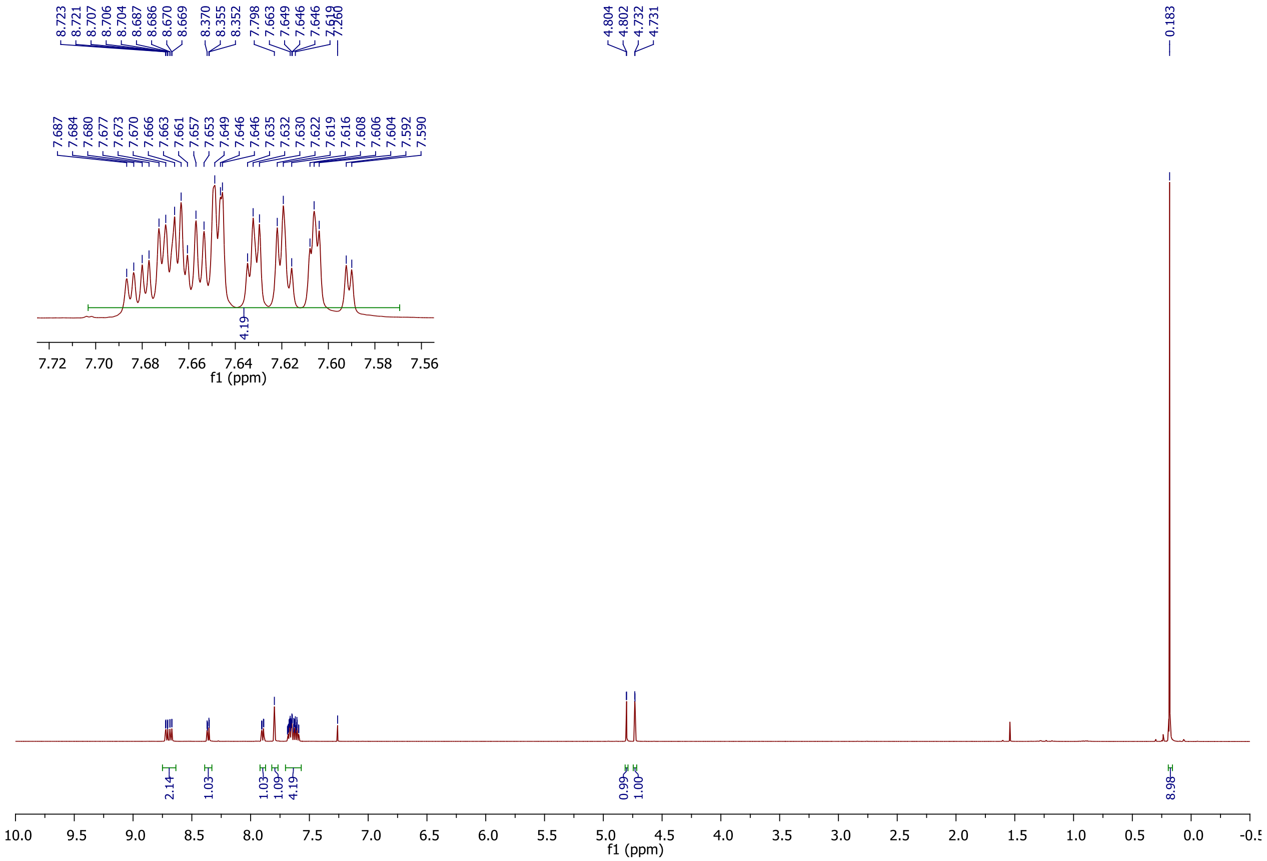

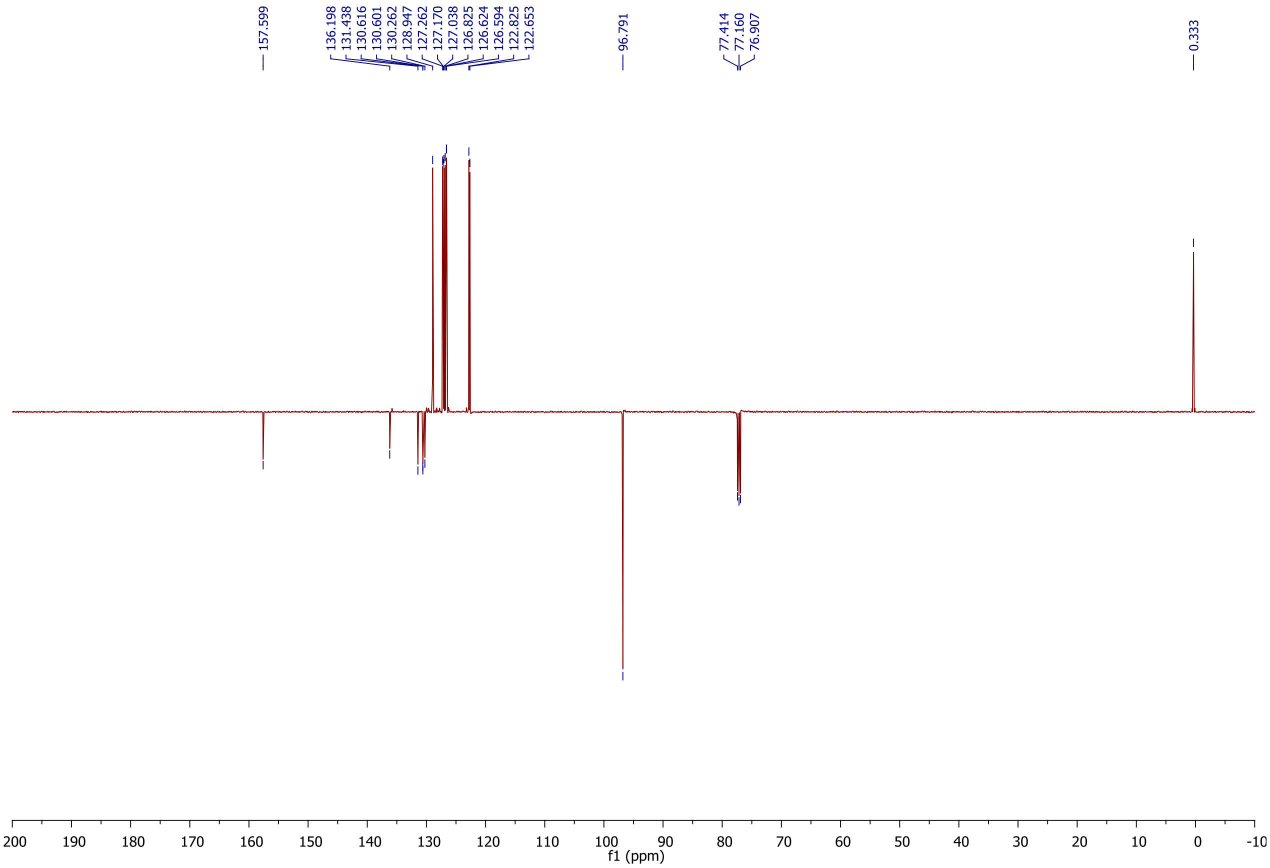

tert-Butyldimethyl((1-(thiophen-2-yl)vinyl)oxy)silane

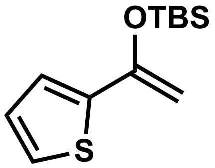

The title compound *tert*-butyldimethyl((1-(thiophen-2-yl)vinyl)oxy)silane was prepared according to general procedure 2 from 2-acetylthiophene (540 µL, 5.00 mmol, 1 equiv), triethylamine (836 µL, 6.00 mmol, 1.2 equiv), and TBSOTf (1.26 mL, 5.50 mmol, 1.1 equiv) and purified by flash silica column chromatography to give a colorless oil (1.07 g, 89% yield). R_f = 0.25 (eluent = 100% petroleum ether); ν_{max} / cm^{-1} (film) 2929, 2856, 1608, 1471, 1253, 1001, 827, 700; ^{1}H NMR (500 MHz, $CDCl_3$) δ_H: 0.24 (6H, s), 1.02 (9H, s), 4.31 (1H, d, *J* 1.9), 4.80 (1H, d, *J* 1.9), 6.96-6.98 (1H, m), 7.20 (2H, dd, 7.8, 4.3); ^{13}C{^{1}H} NMR (126 MHz, $CDCl_3$) δ_C: -4.5, 18.4, 25.9, 89.8, 123.9, 125.1, 127.4, 143.1, 151.3; HRMS (ASAP$^+$) calculated for $[C_{12}H_{21}OSiS]^+$ $(M+H)^+$: m/z 241.1082, found 241.1089 (+2.9 ppm).

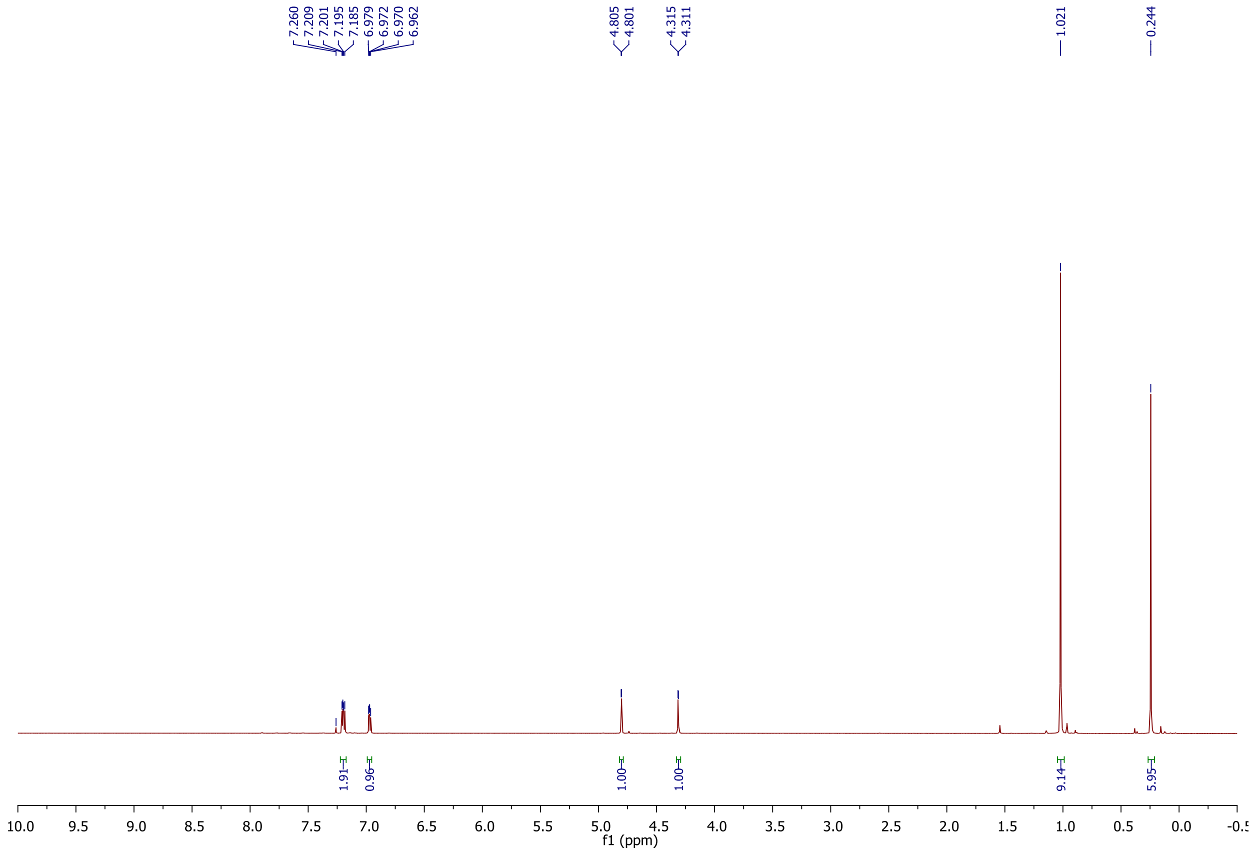
7.260
7.209
7.201
7.195
7.185
6.979
6.972
6.970
6.962
4.805
4.801
4.315
4.311
1.021
0.244
1.91
0.96
1.00
1.00
9.14
5.95
10.0 9.5 9.0 8.5 8.0 7.5 7.0 6.5 6.0 5.5 5.0 4.5 4.0 3.5 3.0 2.5 2.0 1.5 1.0 0.5 0.0
f1 (ppm)

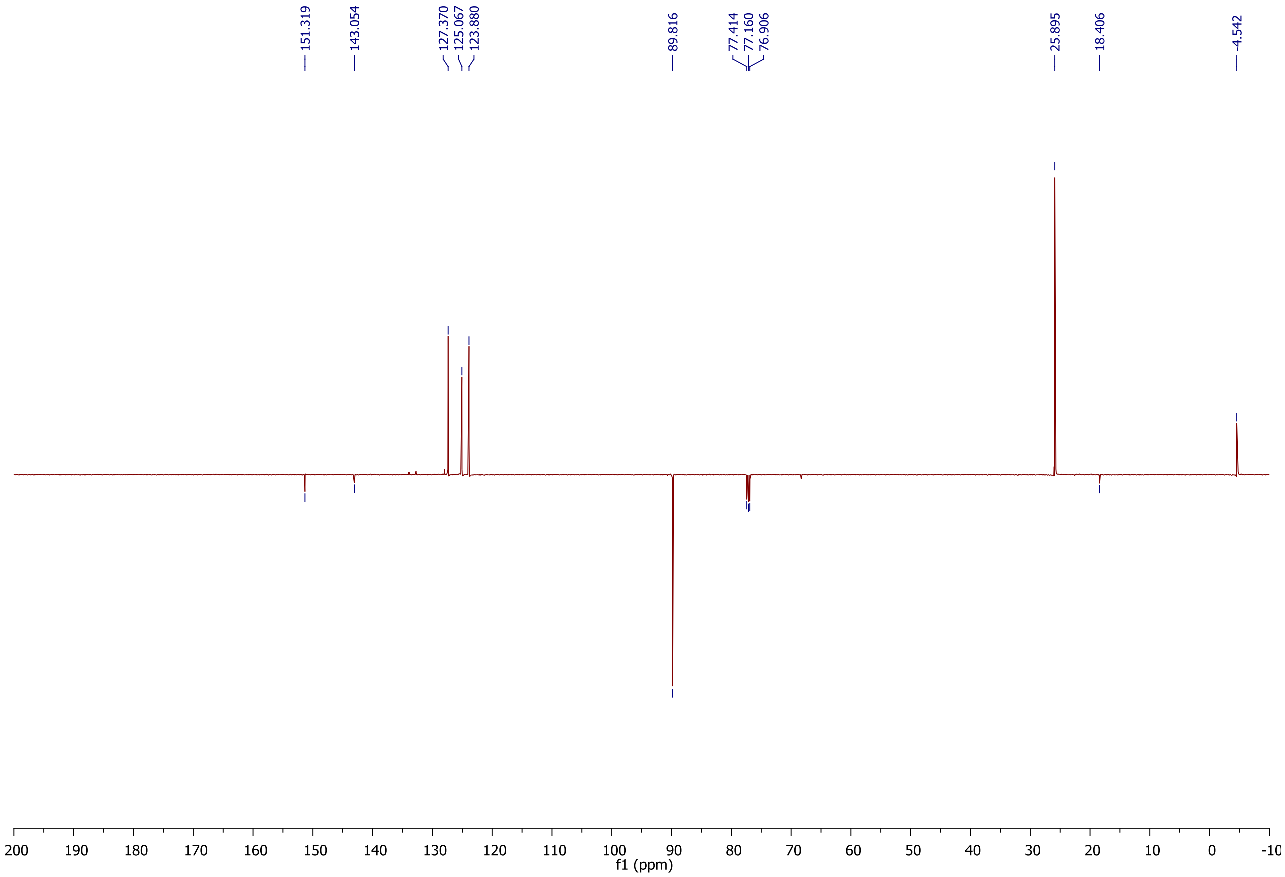
151.319
143.054
127.370
125.067
123.880
89.816
77.414
77.160
76.906
25.895
18.406
-4.542
200 190 180 170 160 150 140 130 120 110 100 90 80 70 60 50 40 30 20 10 0
f1 (ppm)

((1-(Benzo[*b*]thiophen-2-yl)vinyl)oxy)(*tert*-butyl)dimethylsilane

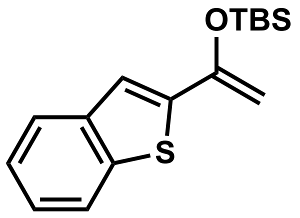

The title compound ((1-(benzo[*b*]thiophen-2-yl)vinyl)oxy)(*tert*-butyl)dimethylsilane was prepared according to general procedure 2 from 2-acetylbenzothiophene (881 µL, 5.00 mmol, 1 equiv), triethylamine (836 µL, 6.00 mmol, 1.2 equiv), and TBSOTf (1.26 mL, 5.50 mmol, 1.1 equiv) and purified by flash silica column chromatography to give a colorless oil (1.28 g, 88% yield). R_f = 0.27 (eluent = 100% petroleum ether); ν_{max} / cm^{-1} (film) 2956, 2856, 1583, 1482, 1251, 1002, 836, 779; ^{1}H NMR (500 MHz, $CDCl_3$) δ_H: 0.29 (6H, s), 1.07 (9H, s), 4.49 (1H, d, *J* 2.1), 4.94 (1H, d, *J* 2.1), 7.29-7.35 (2H, m), 7.43 (1H, s), 7.73-7.75 (1H, m), 7.77-7.79 (1H, m); ^{13}C{^{1}H} NMR (126 MHz, $CDCl_3$) δ_c: -4.5, 18.5, 25.9, 92.7, 120.7, 122.3, 123.9, 124.5, 124.7, 139.7, 140.1, 142.6, 151.4; HRMS (ASAP$^+$) calculated for $[C_{16}H_{23}OSiS]^+$ $(M+H)^+$: m/z 291.1239, found 291.1244 (+1.7 ppm).

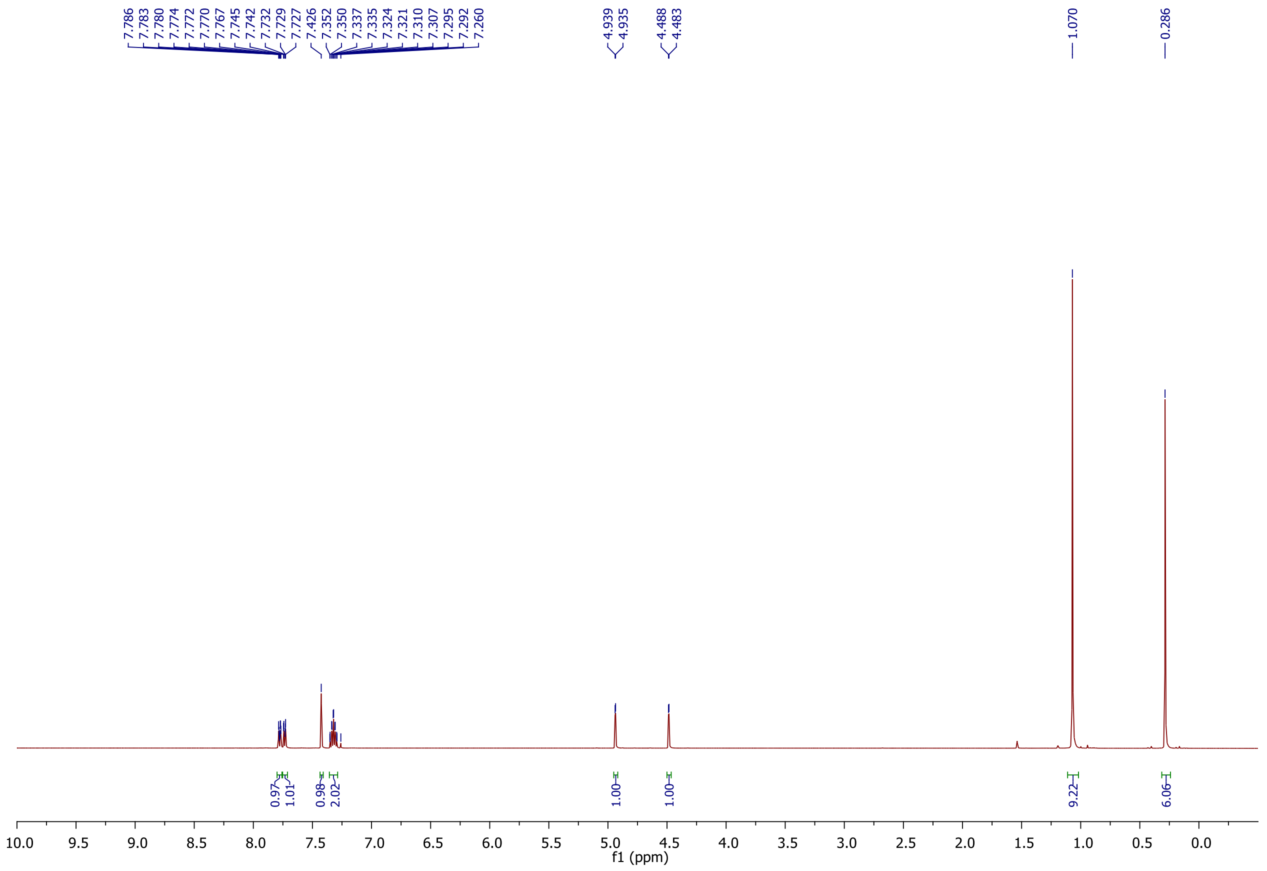

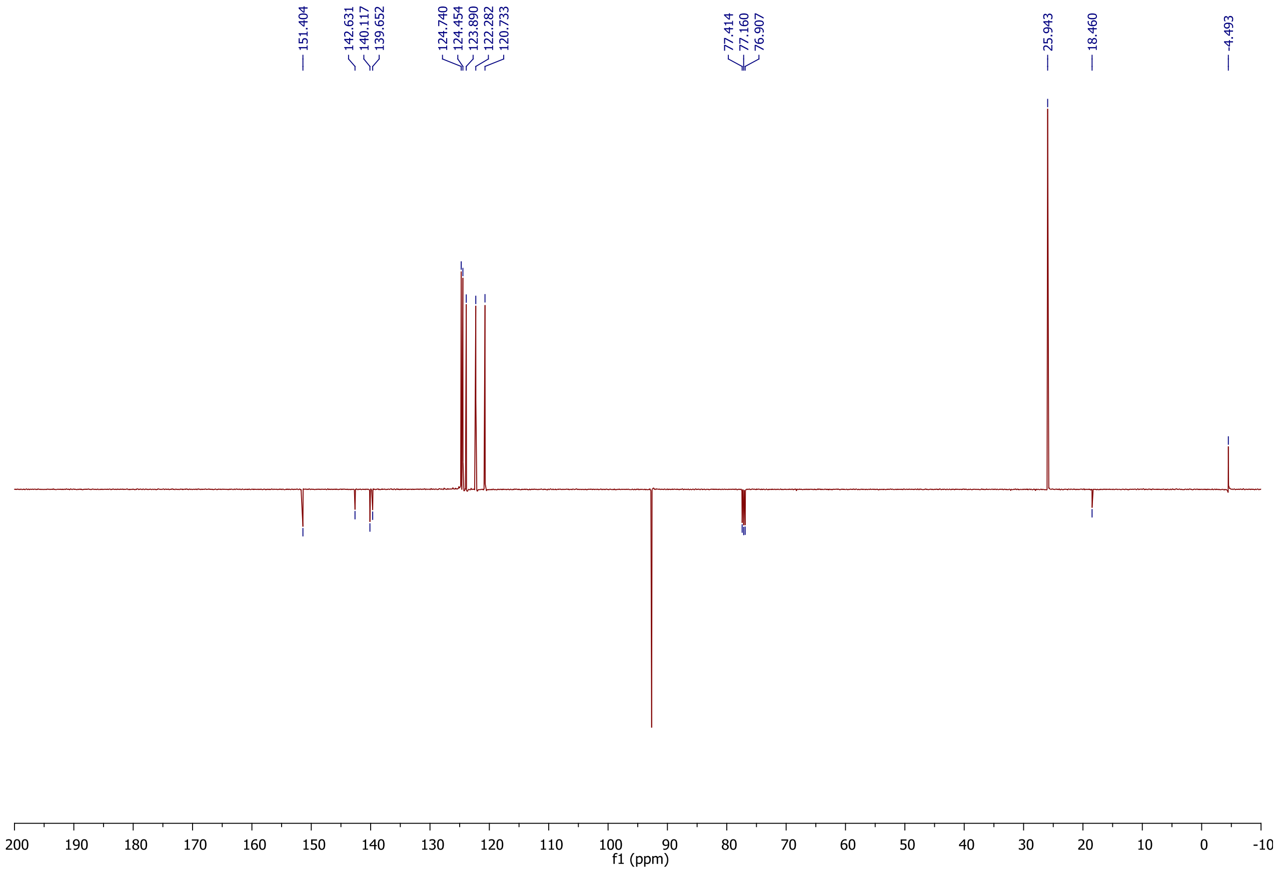

((1-(Benzofuran-2-yl)vinyl)oxy)(*tert*-butyl)dimethylsilane

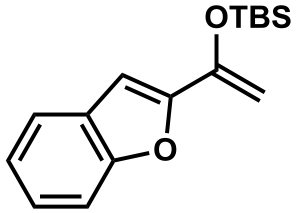

The title compound ((1-(benzofuran-2-yl)vinyl)oxy)(*tert*-butyl)dimethylsilane was prepared according to general procedure 2 from 2-acetylbenzofuran (800 µL, 5.00 mmol, 1 equiv), triethylamine (836 µL, 6.00 mmol, 1.2 equiv), and TBSOTf (1.26 mL, 5.50 mmol, 1.1 equiv) and purified by flash silica column chromatography to give a colorless oil (1.23 g, 90% yield). R_f = 0.28 (eluent = 100% petroleum ether); ν_{max} / cm^{-1} (film) 2954, 2854, 1629, 1552, 1448, 1253, 1001, 831, 740; ^{1}H NMR (500 MHz, $CDCl_3$) δ_H: 0.28 (6H, s), 1.06 (9H, s), 4.59 (1H, d, *J* 1.7), 5.21 (1H, d, *J* 1.7), 6.81 (1H, s), 7.21-7.24 (1H, m), 7.28-7.31 (1H, m), 7.46-7.47 (1H, m), 7.56-7.58 (1H, m); ^{13}C{^{1}H} NMR (126 MHz, $CDCl_3$) δ_c: -4.5, 18.4, 25.9, 93.1, 103.5, 111.2, 121.4, 123.0, 124.8, 128.8, 147.9, 154.2, 155.1; HRMS (ASAP$^+$) calculated for $[C_{16}H_{23}O_2Si]^+$ $(M+H)^+$: m/z 275.1467, found 275.1469 (+0.7 ppm).

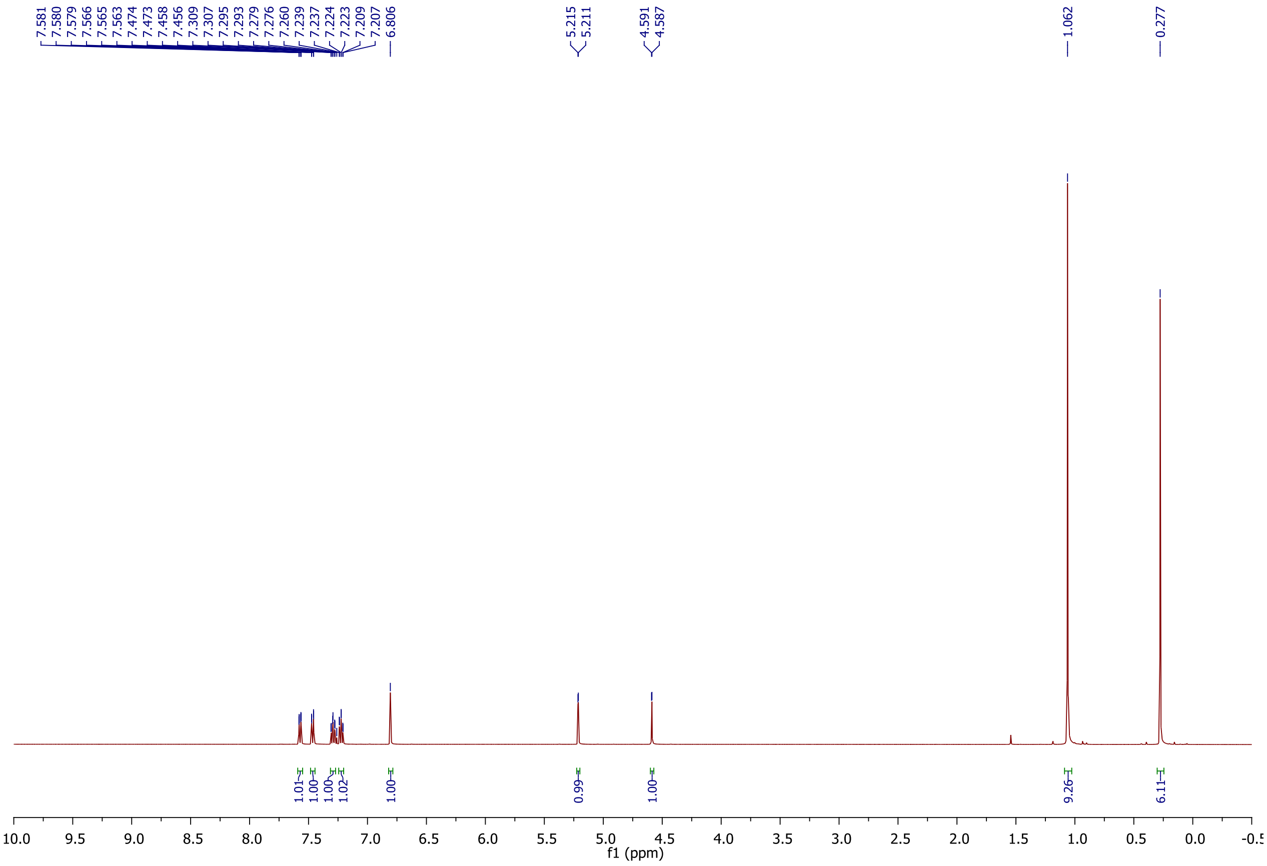
7.581
7.580
7.579
7.566
7.565
7.563
7.474
7.473
7.458
7.456
7.309
7.307
7.295
7.293
7.279
7.276
7.260
7.239
7.237
7.224
7.223
7.209
7.207
6.806
5.215
5.211
4.591
4.587
1.062
0.277
1.01
1.00
1.00
1.02
1.00
0.99
1.00
9.26
6.11
f1 (ppm)

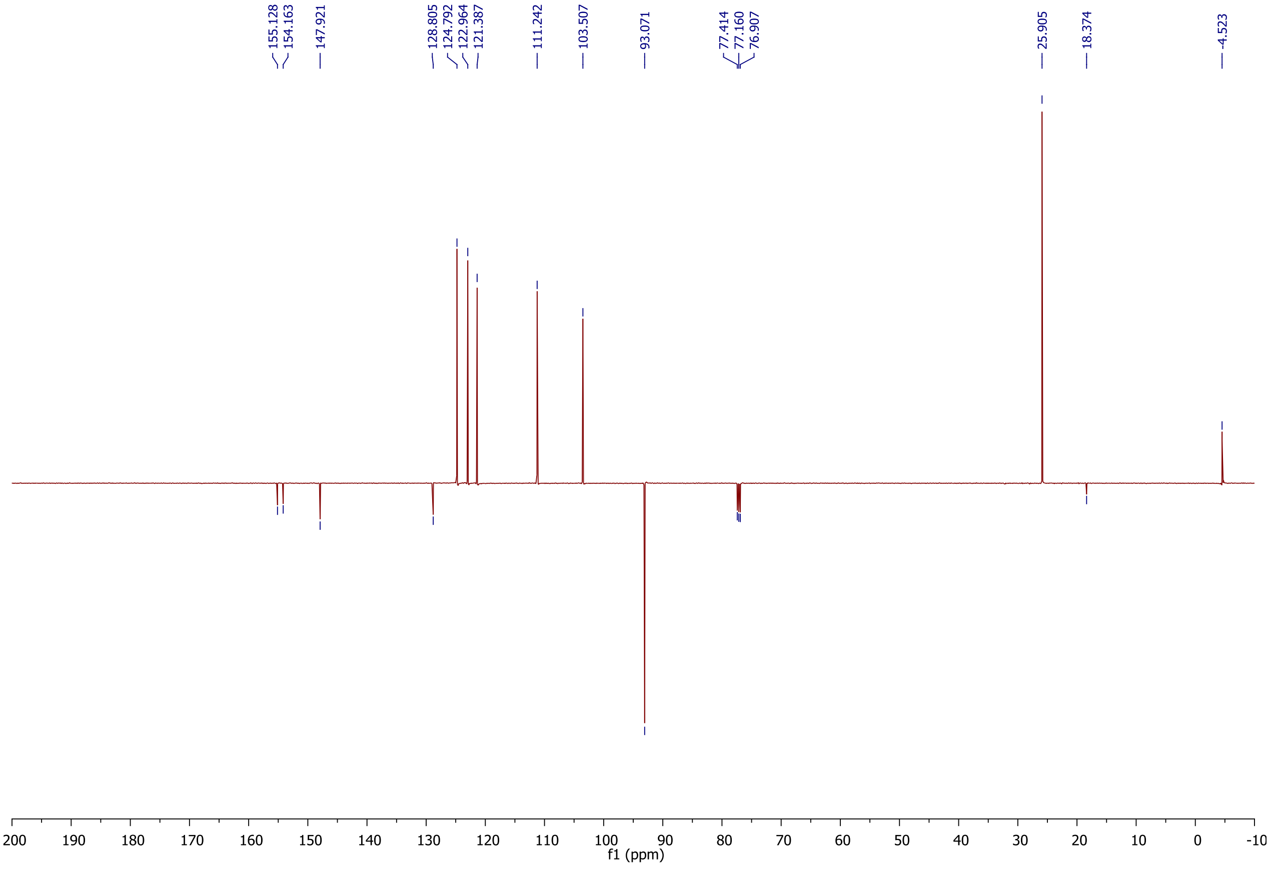
155.128
154.163
147.921
128.805
124.792
122.964
121.387
111.242
103.507
93.071
77.414
77.160
76.907
25.905
18.374
-4.523
f1 (ppm)

((1*H*-inden-3-yl)oxy)(*tert*-butyl)dimethylsilane

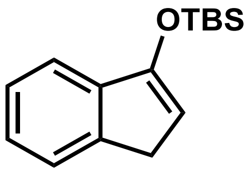

The title compound ((1*H*-inden-3-yl)oxy)(*tert*-butyl)dimethylsilane was prepared according to general procedure 2 from 1-indanone (660 µL, 5.00 mmol, 1 equiv), triethylamine (836 µL, 6.00 mmol, 1.2 equiv), and TBSOTf (1.26 mL, 5.50 mmol, 1.1 equiv) and purified by flash silica column chromatography to give a colorless oil (1.05 g, 85% yield). R_f = 0.20 (eluent = 100% petroleum ether); ν_{max} / cm^{-1} (film) 2954, 2856, 1602, 1573, 1471, 1244, 1080, 866, 678; ^{1}H NMR (500 MHz, $CDCl_3$) δ_H: 0.29 (6H, s), 1.07 (9H, s), 3.30 (2H, d, *J* 2.4), 5.44 (1H, t, *J* 2.4), 7.23-7.26 (1H, m), 7.31-7.33 (1H, m), 7.41-7.43 (2H, m); ^{13}C{^{1}H} NMR (126 MHz, $CDCl_3$) δ_c: -4.5, 18.4, 25.9, 34.1, 106.0, 118.3, 123.9, 125.2, 126.1, 142.1, 142.9, 153.9; HRMS (ASAP$^+$) calculated for $[C_{15}H_{23}OSi]^+$ $(M+H)^+$: m/z 247.1518, found 247.1522 (+1.6 ppm).

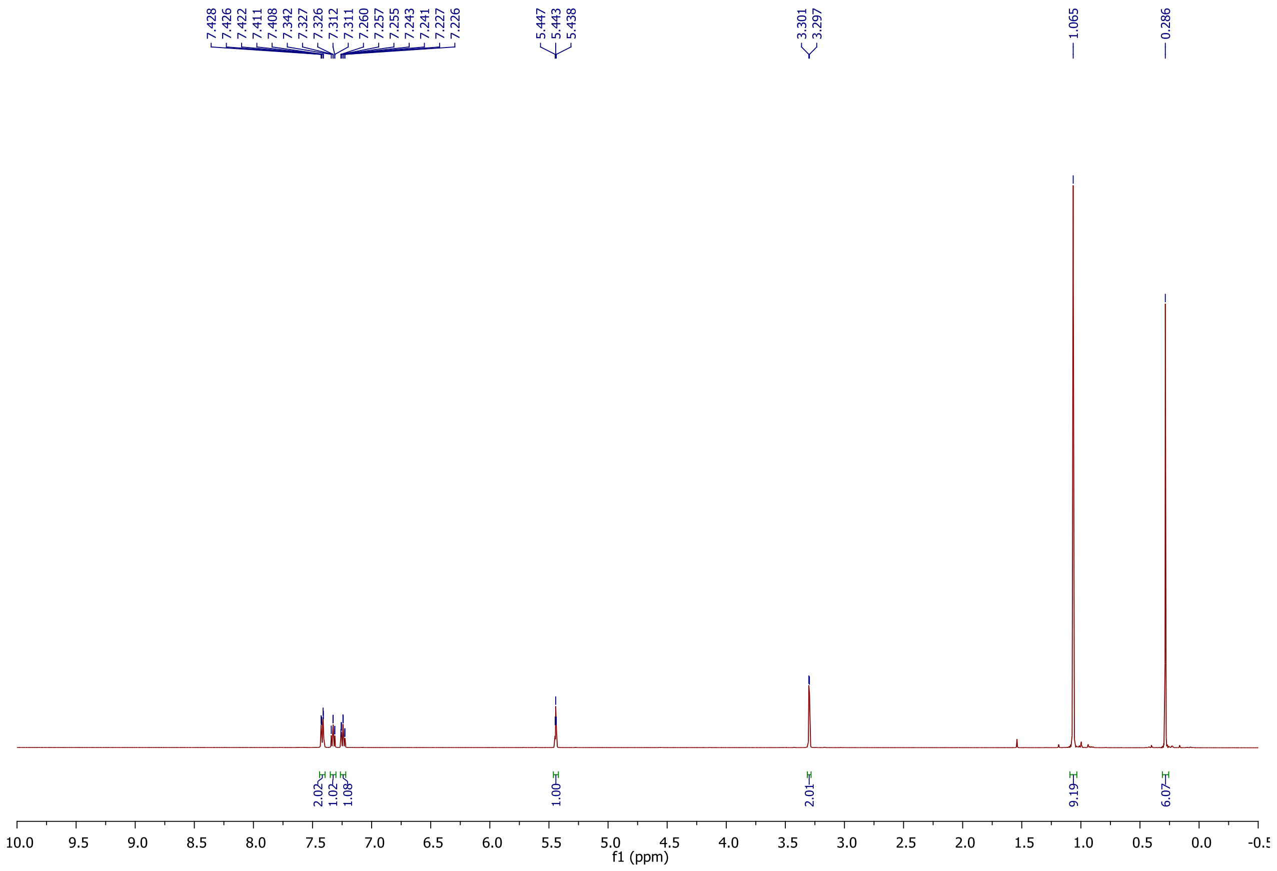

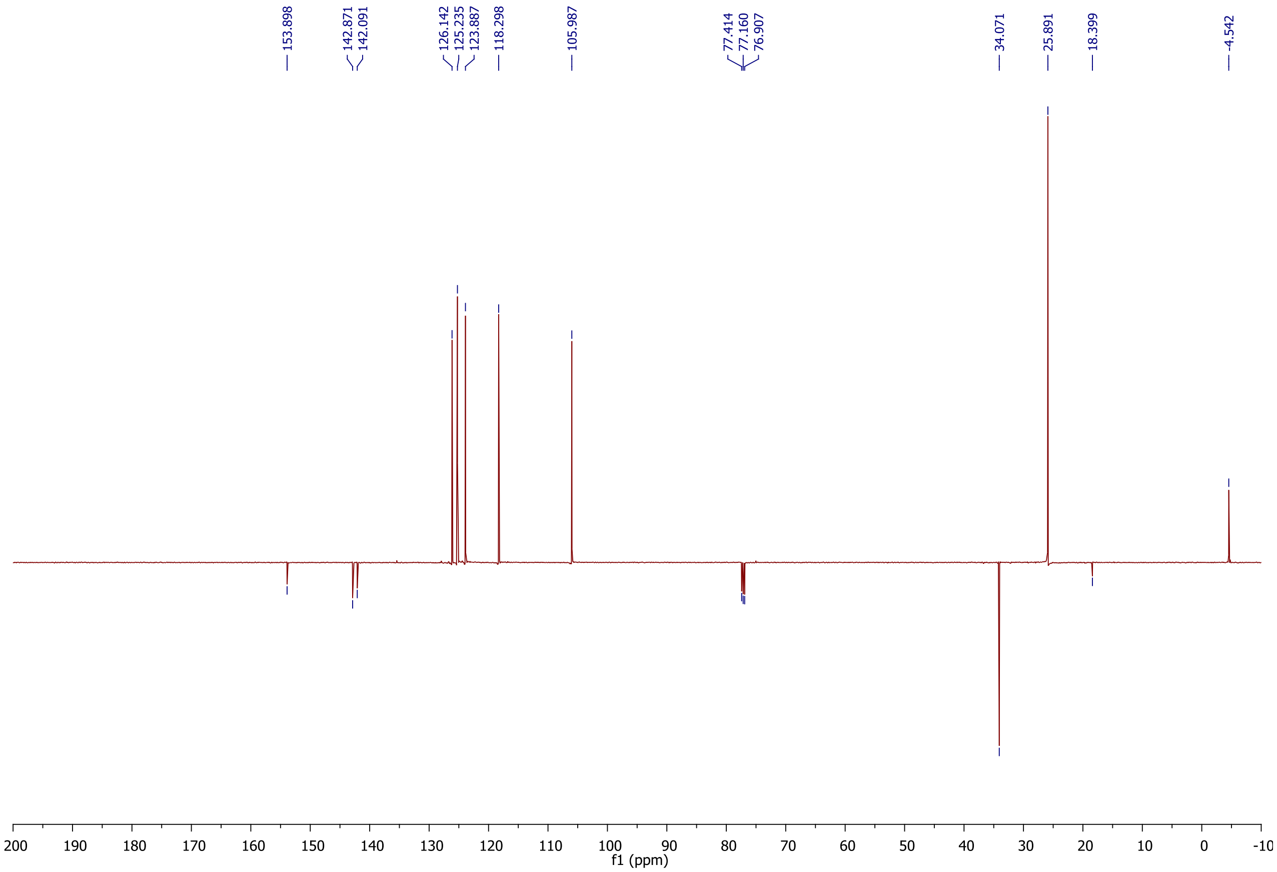

(Cyclohex-1-en-1-yloxy)trimethylsilane

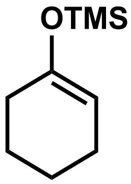

The title compound (cyclohex-1-en-1-yloxy)trimethylsilane was prepared according to general procedure 1 from cyclohexanone (2.07 mL, 20.0 mmol, 1 equiv), triethylamine (4.18 mL, 30.0 mmol, 1.5 equiv), sodium iodide (3.60 g, 24.0 mmol, 1.2 equiv), and chlorotrimethylsilane (3.04 mL, 24.0 mmol, 1.2 equiv) and purified by Kugelrohr distillation (bp 69-70 °C (18 mmHg)) {Lit.[14] 75-76 °C (20 mmHg)} to give a colorless oil (2.89 g, 85% yield). R_f = 0.28 (eluent = 100% petroleum ether); ν_{max} / cm^{-1} (film) 2929, 1668, 1367, 1249, 1184, 839, 750; ^{1}H NMR (400 MHz, $CDCl_3$) δ_H: 0.17 (9H, s), 1.47-1.54 (2H, m), 1.62-1.68 (2H, m), 1.96-2.02 (4H, m), 4.85-4.87 (1H, m); ^{13}C{^{1}H} NMR (101 MHz, $CDCl_3$) δ_C: 0.5, 22.5, 23.3, 23.9, 30.0, 104.4, 150.4. Spectroscopic data in accordance with that stated in the literature.[15]

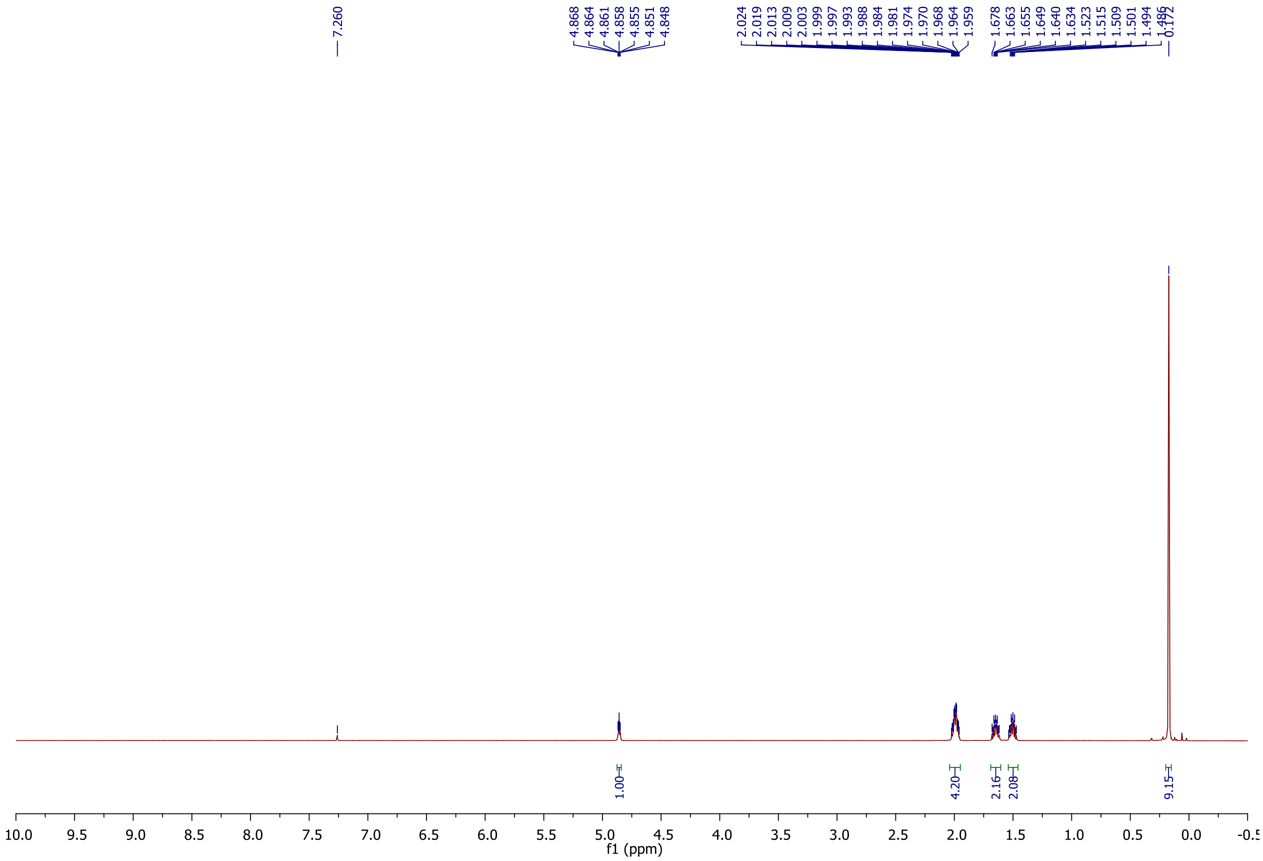
7.260
4.868
4.864
4.861
4.858
4.855
4.851
4.848
2.024
2.019
2.013
2.009
2.003
1.999
1.997
1.993
1.988
1.984
1.981
1.974
1.970
1.968
1.964
1.959
1.678
1.663
1.655
1.649
1.640
1.634
1.523
1.515
1.509
1.501
1.494
0.172
1.00
4.20
2.16
2.08
9.15
f1 (ppm)

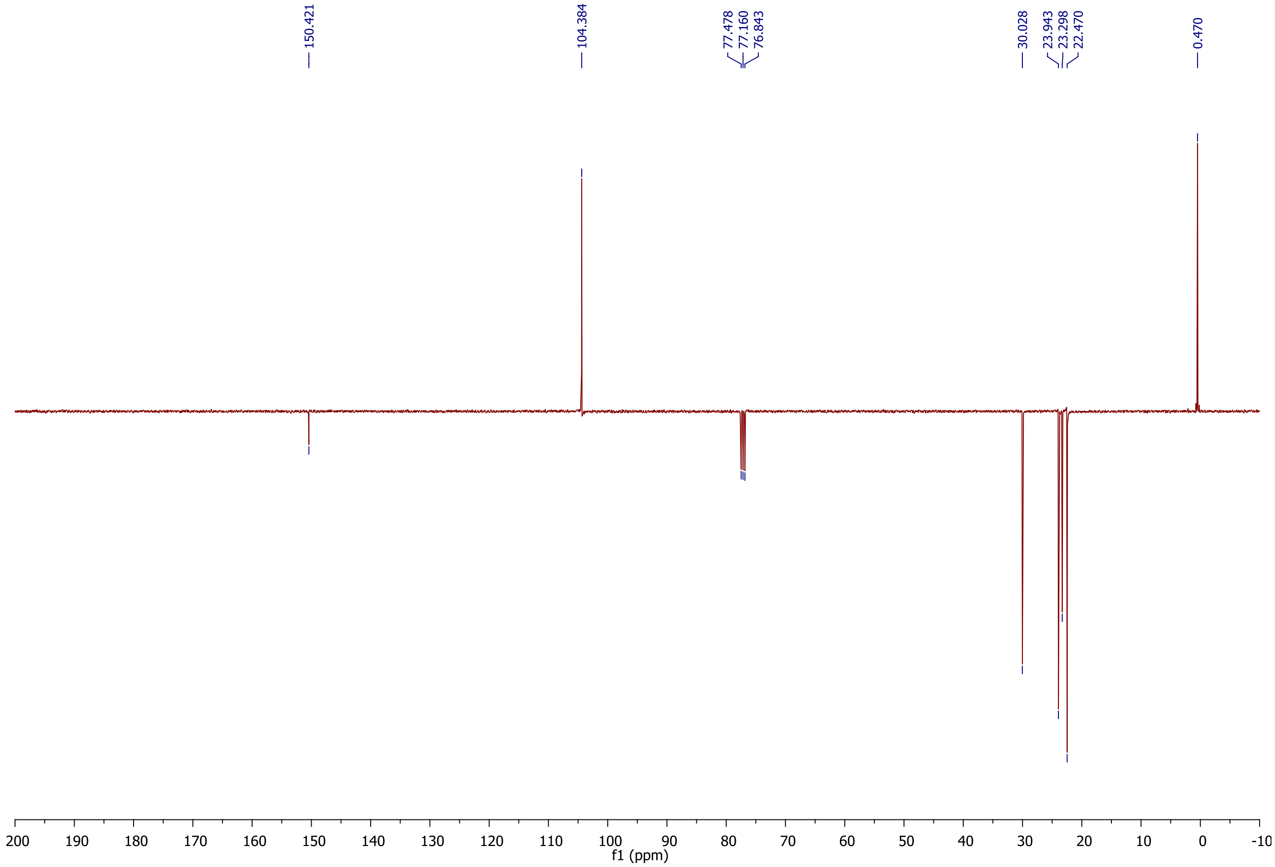
150.421
104.384
77.478
77.160
76.843
30.028
23.943
23.298
22.470
0.470
f1 (ppm)

((3,3-Dimethylbut-1-en-2-yl)oxy)trimethylsilane

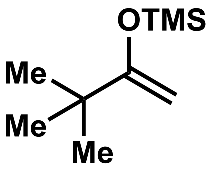

The title compound ((3,3-dimethylbut-1-en-2-yl)oxy)trimethylsilane was prepared according to general procedure 1 from 3,3-dimethyl-2-butanone (2.50 mL, 20.0 mmol, 1 equiv), triethylamine (4.18 mL, 30.0 mmol, 1.5 equiv), sodium iodide (3.60 g, 24.0 mmol, 1.2 equiv), and chlorotrimethylsilane (3.04 mL, 24.0 mmol, 1.2 equiv) and purified by Kugelrohr distillation (bp 65-66 °C (80 mmHg)) {Lit.[16] 68 °C (80 mmHg)} to give a colorless oil (2.00 g, 58% yield). R_f = 0.44 (eluent = 100% petroleum ether); ^{1}H NMR (500 MHz, $CDCl_3$) δ_H: 0.21 (9H, s), 1.05 (9H, s), 3.93 (1H, d, *J* 1.3), 4.09 (1H, d, *J* 1.3); ^{13}C{^{1}H} NMR (126 MHz, $CDCl_3$) δ_C: 0.3, 28.2, 36.6, 85.9, 167.4. Spectroscopic data in accordance with that stated in the literature.[15]

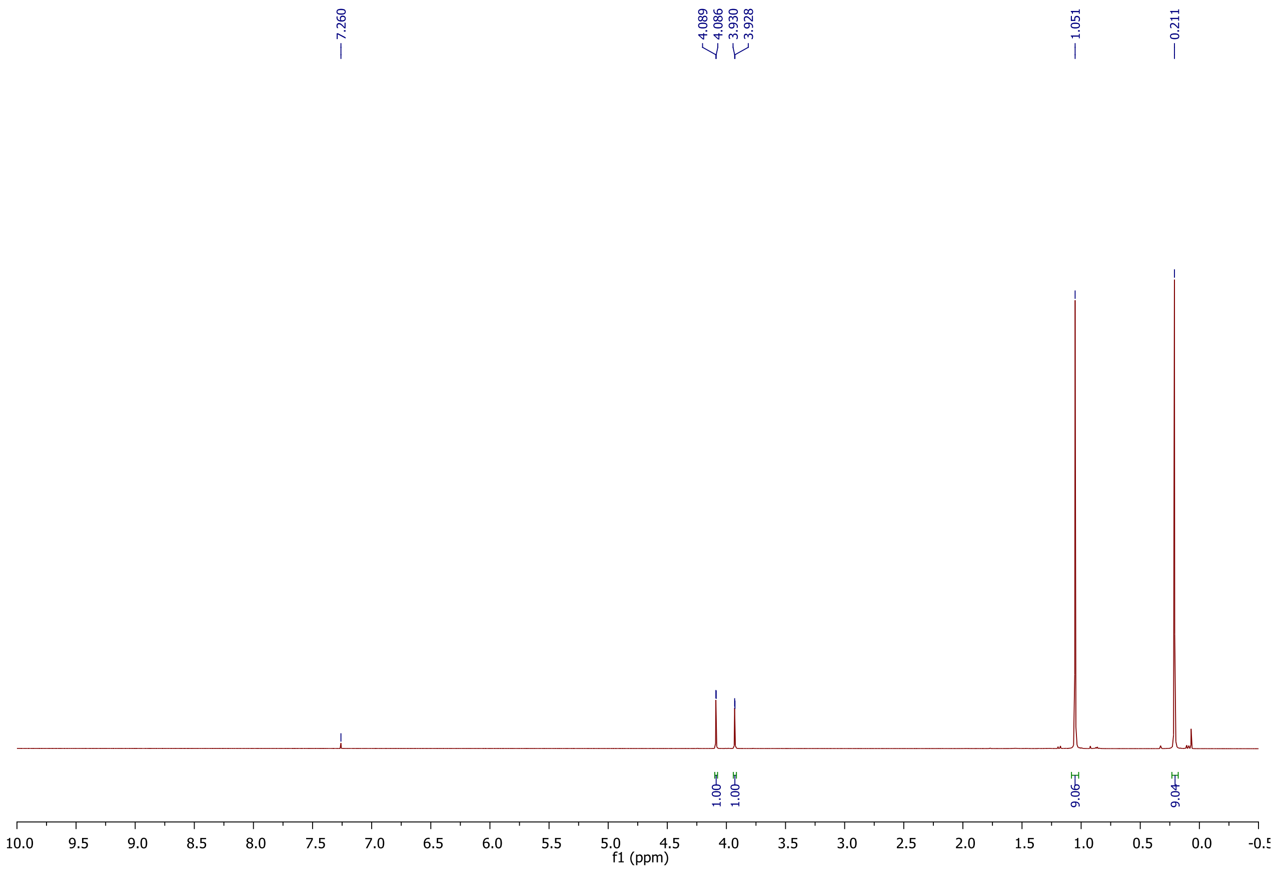

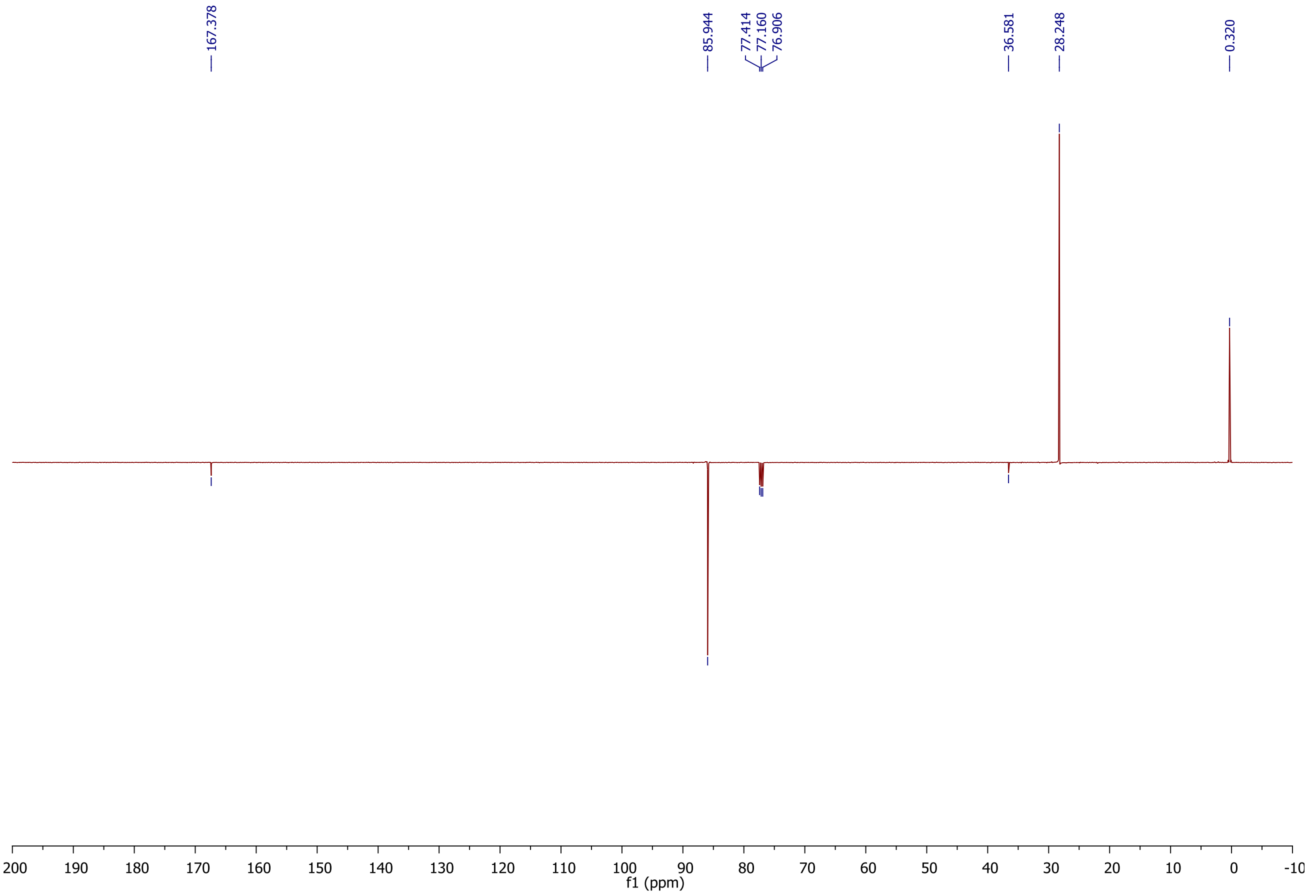

1.2.2. Synthesis of 1,5-dimethoxycyclohexa-1,4-diene

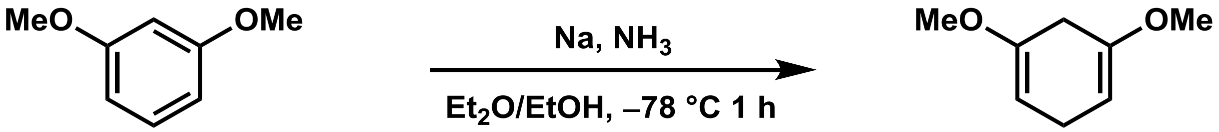

Ammonia (103 mL, 2.94 mL/mmol) was condensed into a three-necked 2 L-flask equipped with a Schlenk valve and a Dewar condenser at –78 °C. Sodium metal (5.63 g, 245 mmol, 7.0 equiv) was cut into pieces of approximately 1 cm^3 and added portionwise over 20 min at –78 °C. After complete addition the dark blue solution was vigorously stirred for 30 minutes at –78 °C. A solution of 1,3-dimethoxybenzene (4.58 mL, 35 mmol, 1 equiv) in a mixture of ethanol (11.2 mL, 0.32 mL/mmol) and diethyl ether (18 mL, 0.51 mL/mmol) was added at –78 °C within 30 min. The dark blue suspension was stirred for another hour and then, sat. aq. NH_4Cl (50 mL) was added. The colorless solution was allowed to warm to –20 °C. A mixture of ethanol/distilled water (1:1, 5 mL) and then distilled water (50 mL) was added. After warming to room temperature, the ammonia was evaporated overnight under a stream of argon. By

sparging the solution with argon for 10 minutes, the last traces of ammonia were removed and the aqueous layer was extracted with n-hexane/ethyl acetate (1:1, 5 × 50 mL). The combined organic extracts were washed with brine (3 × 50 mL), dried over anhydrous $MgSO_4$ and filtered. Removal of the solvent under reduced pressure afforded the product as a colorless liquid (4.90 g, 84% yield). ^{1}H NMR (400 MHz, $CDCl_3$) δ_H: 2.74-2.79 (2H, m), 2.81-2.87 (2H, m), 3.55 (6H, s), 4.63-4.65 (2H, m); ^{13}C{^{1}H} NMR (101 MHz, $CDCl_3$) δ_c: 25.0, 31.1, 54.2, 90.8, 152.1. Spectroscopic data in accordance with that stated in the literature.[17]

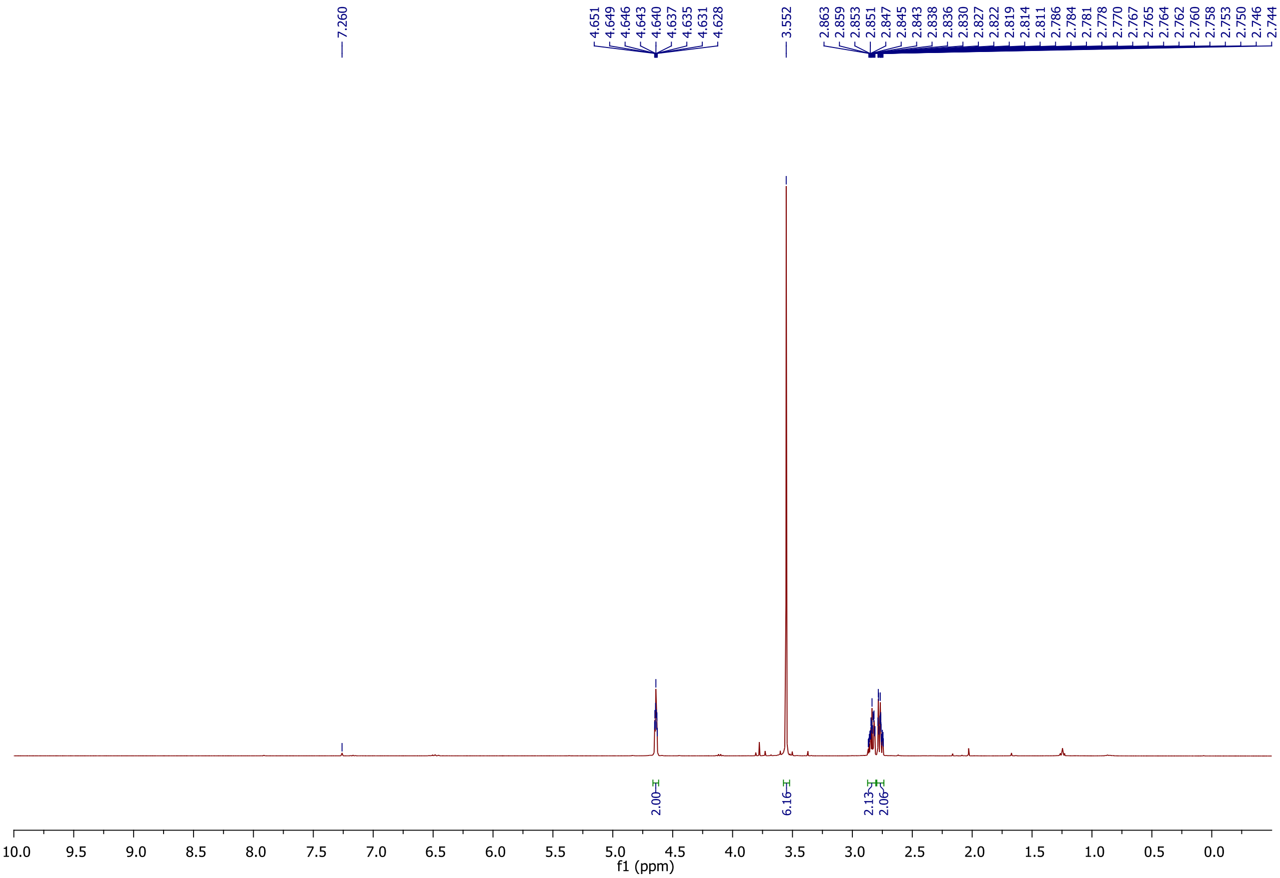

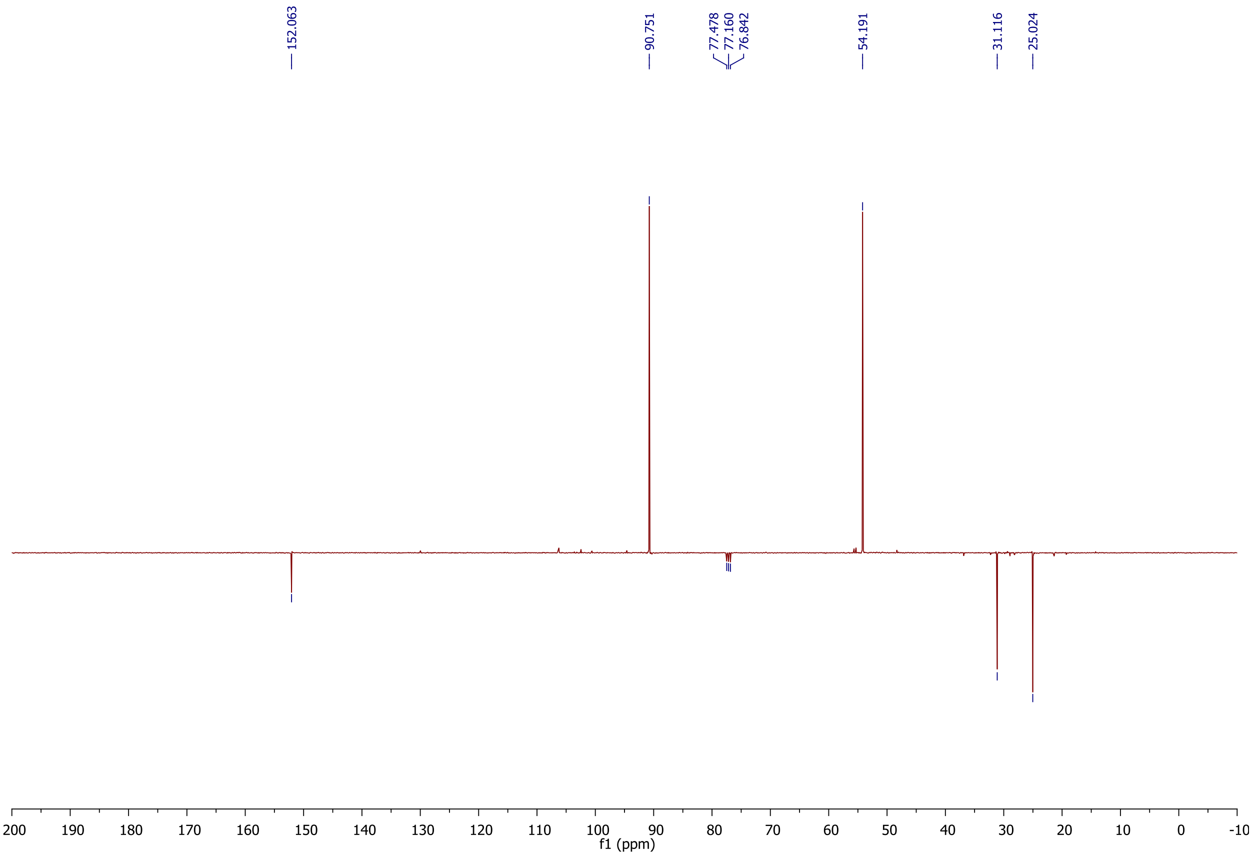
152.063
90.751
77.478
77.160
76.842
54.191
31.116
25.024
200 190 180 170 160 150 140 130 120 110 100 90 80 70 60 50 40 30 20 10 0 -10
f1 (ppm)

1.2.3 Optimization studies

Table 1. Optimization of the FLP-catalysed transfer hydrogenation[a]

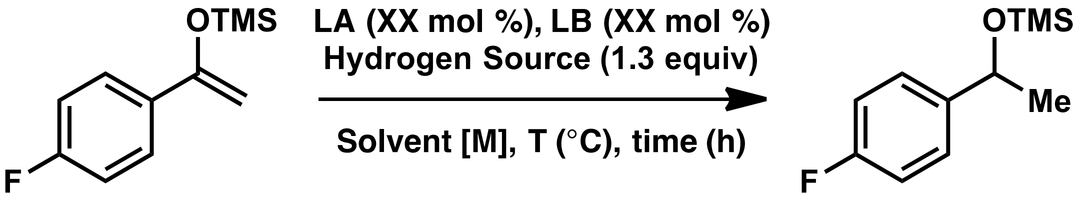

Entry	LA (mol%)	LB (mol%)	Hydrogen Source (equiv)	Solvent ([M])	T (°C)	Time (h)	Conv (%)[b]
**Starting Point**							
1	BCF (10)	TMP (10)	γ-terpinene (1.3)	Toluene ([0.16)]	130	4	96
**Lewis Acid**							
2	2,4,6-(10)	TMP (10)	γ-terpinene (1.3)	Toluene ([0.16)]	130	4	7
3	2,6-(10)	TMP (10)	γ-terpinene (1.3)	Toluene ([0.16)]	130	4	<2
4	None	TMP (10)	γ-terpinene (1.3)	Toluene ([0.16)]	130	4	<2
**Lewis Base**							
5	BCF (10)	DABCO (10)	γ-terpinene (1.3)	Toluene ([0.16)]	130	4	41
6	BCF (10)	Quinuclidine (10)	γ-terpinene (1.3)	Toluene ([0.16)]	130	4	17
7	BCF (10)	2,6-lutidine (10)	γ-terpinene (1.3)	Toluene ([0.16)]	130	4	62
8	BCF (10)	2,4,6-collidine (10)	γ-terpinene (1.3)	Toluene ([0.16)]	130	4	52
9	BCF (10)	PMP (10)	γ-terpinene (1.3)	Toluene ([0.16)]	130	4	36
10	BCF (10)	tBu_3P (10)	γ-terpinene (1.3)	Toluene ([0.16)]	130	4	43
11	BCF (10)	None	γ-terpinene (1.3)	Toluene ([0.16)]	130	4	<2
**Hydrogen Source**							
12	BCF (10)	TMP (10)	dimethoxy (1.3)	Toluene ([0.16)]	130	4	23
13	BCF (10)	TMP (10)	cyclohexadiene (1.3)	Toluene ([0.16)]	130	4	<2
14	BCF (10)	TMP (10)	Ammonia borane (1.3)	Toluene ([0.16)]	130	4	<2
**Solvent**							
15	BCF (10)	TMP (10)	γ-terpinene (1.3)	Benzene ([0.16)]	130	4	46
16	BCF (10)	TMP (10)	γ-terpinene (1.3)	THF ([0.16)]	80	4	<2
17	BCF (10)	TMP (10)	γ-terpinene (1.3)	Dioxane ([0.16)]	130	4	<2
18	BCF (10)	TMP (10)	γ-terpinene (1.3)	Chlorobenzene ([0.16)]	130	4	61
19	BCF (10)	TMP (10)	γ-terpinene (1.3)	Mesitylene ([0.16)]	130	4	90[c]
20	BCF (10)	TMP (10)	γ-terpinene (1.3)	DCE ([0.16)]	120	4	30
21	BCF (10)	TMP (10)	γ-terpinene (1.3)	1,2-$F_2C_6H_4$ ([0.16)]	130	4	8
22	BCF (10)	TMP (10)	γ-terpinene (1.3)	1,2-$F_2C_6H_4$ ([0.16)]	25	4	<2
23	BCF (10)	TMP (10)	γ-terpinene (1.3)	DCM ([0.16)]	60	4	<2
24	BCF (10)	TMP (10)	γ-terpinene (1.3)	DCM ([0.16)]	25	4	<2
**Concentration**							
25	BCF (10)	TMP (10)	γ-terpinene (1.3)	Toluene ([0.32)]	130	4	87
26	BCF (10)	TMP (10)	γ-terpinene (1.3)	Toluene ([0.64)]	130	4	87
27	BCF (10)	TMP (10)	γ-terpinene (1.3)	Toluene ([1.28)]	130	4	80
**Temperature**							
28	BCF (10)	TMP (10)	γ-terpinene (1.3)	Toluene ([0.16)]	60	4	<2
29	BCF (10)	TMP (10)	γ-terpinene (1.3)	Toluene ([0.16)]	25	4	<2
**Time**							
30	BCF (10)	TMP (10)	γ-terpinene (1.3)	Toluene ([0.16)]	130	2	47
31	BCF (10)	TMP (10)	γ-terpinene (1.3)	Toluene ([0.16)]	130	1	34
32	BCF (10)	TMP (10)	γ-terpinene (1.3)	Toluene ([0.16)]	130	16	93
**Catalyst Loading**							
33	BCF (5)	TMP (5)	γ-terpinene (1.3)	Toluene ([0.16)]	130	4	23

[a]Reactions performed using 0.5 mmol of silyl enol ether starting material. [b]Conversion determined by ^1H NMR spectroscopy using 1,3,5-trimethylbenzene as the internal standard. [c]Trimethoxybenzene as the internal standard.

1.2.4. FLP-catalyzed transfer hydrogenation of silyl enol ethers

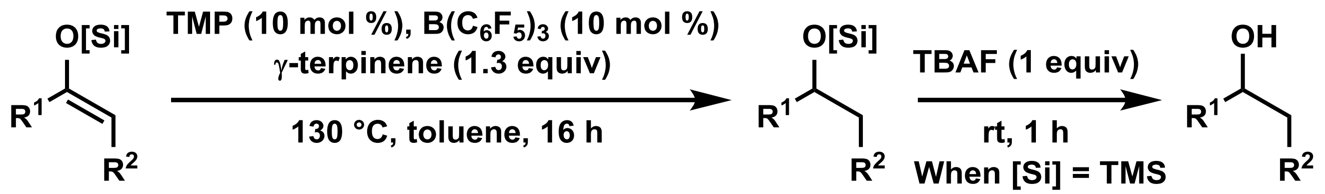

General procedure 3

In a nitrogen-filled glove box, to an appropriate reaction vessel containing a magnetic follower was charged the requisite silyl enol ether (0.50 mmol, 1 equiv), tris(pentafluorophenyl)borane (0.05 mmol, 10 mol %), 2,2,6,6-tetramethylpiperidine (0.05 mmol, 10 mol %) and γ-terpinene (0.65 mmol, 1.3 equiv) in toluene (3 mL). The reaction mixture was heated to 130 °C for 16 h. The reaction mixture was cooled to rt and TBAF (0.50 mmol, 0.5 mL, 1.0 M in THF) was added and stirred at rt for 1 h. The reaction mixture was washed with water (5 mL) and extracted with EtOAc (2 × 5 mL). The combined organic fractions were dried over anhydrous $MgSO_4$. The solvent was removed under reduced pressure and the residue was purified by flash silica column chromatography.

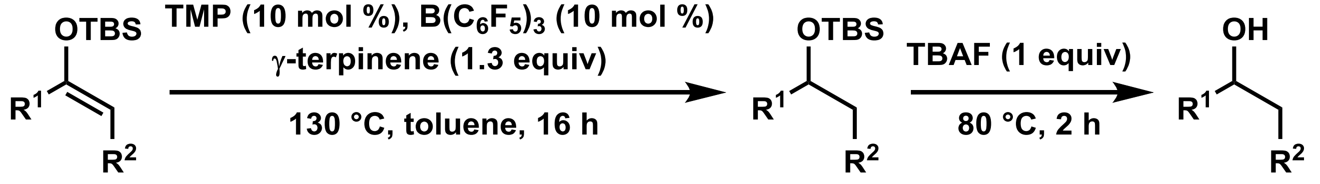

General procedure 4

In a nitrogen-filled glove box, to an appropriate reaction vessel containing a magnetic follower was charged the requisite silyl enol ether (0.50 mmol, 1 equiv), tris(pentafluorophenyl)borane (0.05 mmol, 10 mol %), 2,2,6,6-tetramethylpiperidine (0.05 mmol, 10 mol %) and γ-terpinene (0.65 mmol, 1.3 equiv) in toluene (3 mL). The reaction mixture was heated to 130 °C for 16 h. The reaction mixture was cooled to rt and TBAF (0.50 mmol, 0.5 mL, 1.0 M in THF) was added and stirred at 80 °C for 2 h. The reaction mixture was washed with water (5 mL) and extracted with EtOAc (2 × 5 mL). The combined organic fractions were dried over anhydrous $MgSO_4$. The solvent was removed under reduced pressure and the residue was purified by flash silica column chromatography.

1-Phenylethan-1-ol

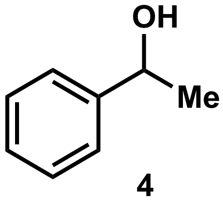

4

The title compound 1-phenylethan-1-ol **4** was prepared according to general procedure 3 from trimethyl((1-phenylvinyl)oxy)silane (96 mg, 0.50 mmol, 1 equiv), tris(pentafluorophenyl)borane (25.6 mg, 0.05 mmol, 10 mol %), 2,2,6,6-tetramethylpiperidine (8.4 μL, 0.05 mmol, 10 mol %) and γ-terpinene (104 μL, 0.65 mmol, 1.3 equiv), followed by TBAF (0.50 mmol, 0.5 mL, 1 equiv) and purified by flash silica column chromatography (eluent = 10% EtOAc in hexanes) to give a colorless oil (49 mg, 80% yield). R_f = 0.33 (eluent = 20% EtOAc in hexanes); ν_{max} / cm^{-1} (film) 3336, 2972, 1492, 1450, 1076, 696; ^{1}H NMR (500 MHz, $CDCl_3$) δ_H: 1.48 (3H, t, *J* 6.5), 2.04 (1H, br s), 4.87 (1H, q, *J* 6.5), 7.24-7.28 (1H, m), 7.32-7.37 (4H, m); $^{13}C\{^{1}H\}$ NMR (126 MHz, $CDCl_3$) δ_c: 25.3, 70.5, 125.5, 127.6, 128.6, 145.9; HRMS (CI^+) calculated for $[C_8H_{14}ON]^+$ $(M+NH_4)^+$: m/z 140.1070, found 140.1069 (-0.6 ppm).

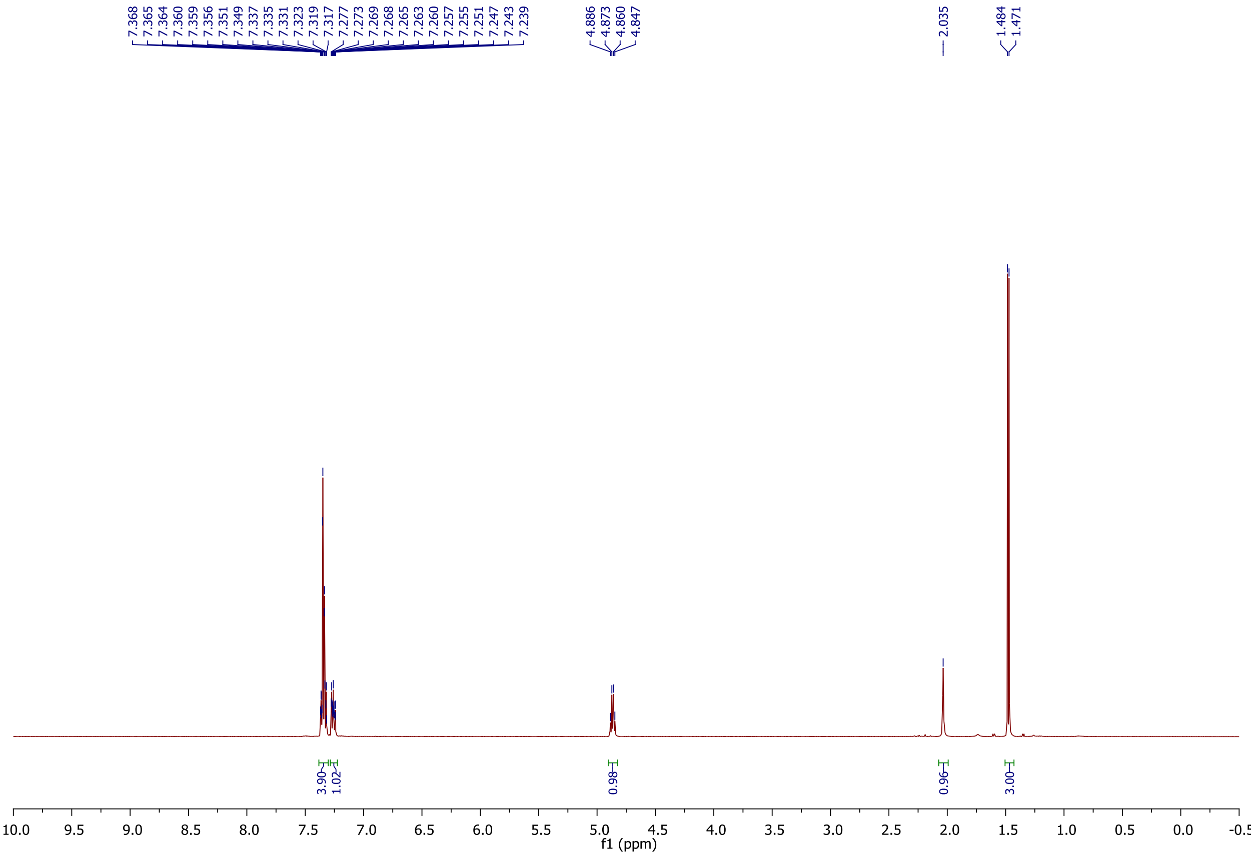

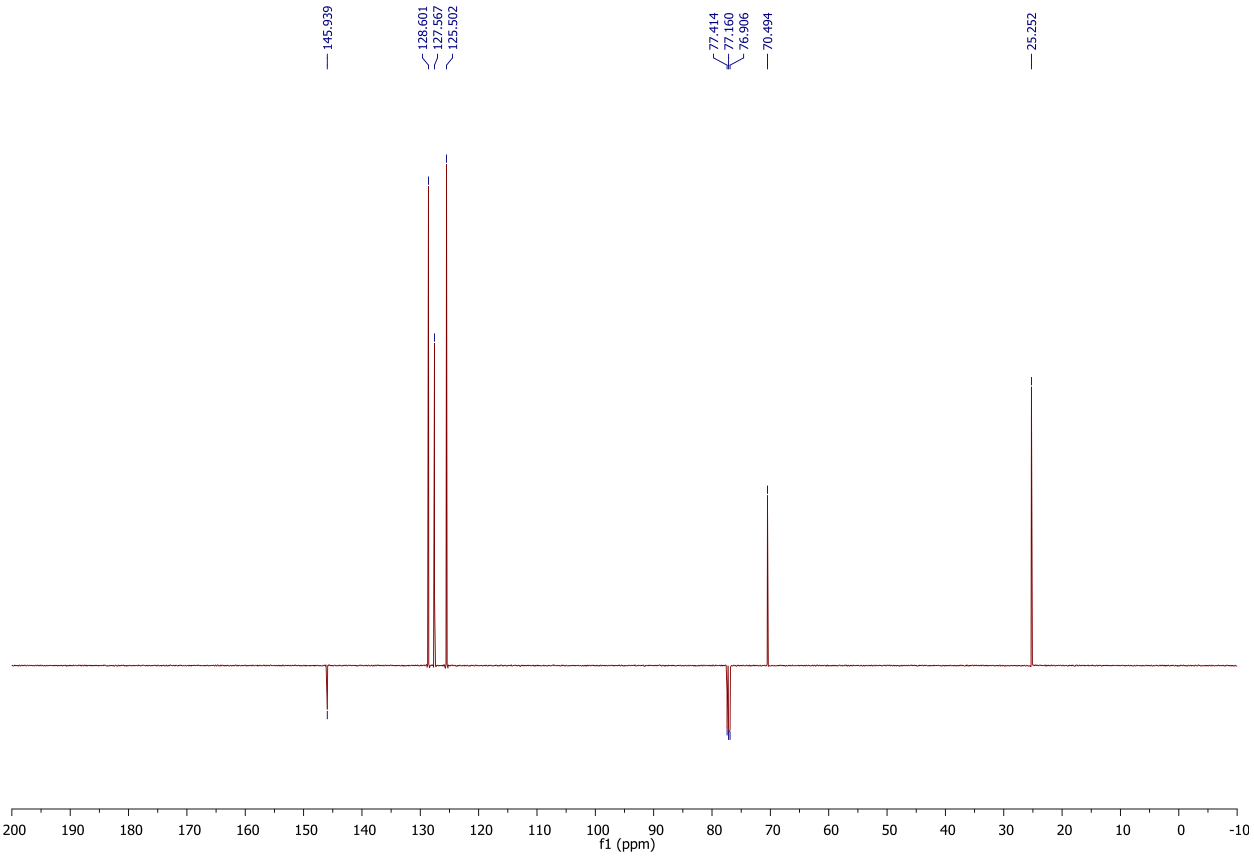

1-Phenylethan-1-ol

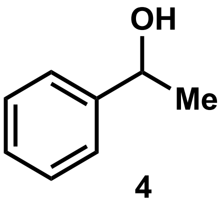

4

The title compound 1-phenylethan-1-ol was prepared according to general procedure 3 from triethyl((1-phenylvinyl)oxy)silane (117 mg, 0.50 mmol, 1 equiv), tris(pentafluorophenyl)borane (25.6 mg, 0.05 mmol, 10 mol %), 2,2,6,6-tetramethylpiperidine (8.4 µL, 0.05 mmol, 10 mol %) and γ-terpinene (104 µL, 0.65 mmol, 1.3 equiv), followed by TBAF (0.50 mmol, 0.5 mL, 1 equiv) and purified by flash silica column chromatography (eluent = 10% EtOAc in hexanes) to give a colorless oil (44 mg, 72% yield), with physical properties and spectroscopic data in accordance with those obtained previously.

1-Phenylethan-1-ol

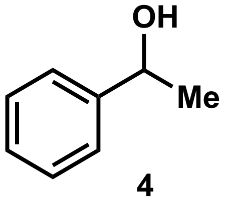

4

The title compound 1-phenylethan-1-ol was prepared according to general procedure 4 from *tert*-butyldimethyl((1-phenylvinyl)oxy)silane (117 mg, 0.50 mmol, 1 equiv), tris(pentafluorophenyl)borane (25.6 mg, 0.05 mmol, 10 mol %), 2,2,6,6-tetramethylpiperidine (8.4 µL, 0.05 mmol, 10 mol %) and γ-terpinene (104 µL, 0.65 mmol, 1.3 equiv), followed by TBAF (0.50 mmol, 0.5 mL, 1 equiv) and purified by flash silica column chromatography (eluent = 10% EtOAc in hexanes) to give a colorless oil (57 mg, 93% yield), with physical properties and spectroscopic data in accordance with those obtained previously.

Triisopropyl(1-phenylethoxy)silane

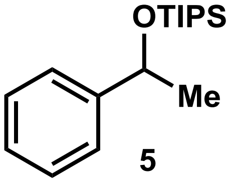

5

The title compound triisopropyl(1-phenylethoxy)silane **5** was prepared according to general procedure 3 from triisopropyl((1-phenylvinyl)oxy)silane (138 mg, 0.50 mmol, 1 equiv), tris(pentafluorophenyl)borane (25.6 mg, 0.05 mmol, 10 mol %), 2,2,6,6-tetramethylpiperidine (8.4 µL, 0.05 mmol, 10 mol %) and γ-terpinene (104 µL, 0.65 mmol, 1.3 equiv) and purified by flash silica column chromatography (eluent = 100% petroleum ether) to give a colorless oil (101 mg, 73% yield). R_f = 0.64 (eluent = 100% *n*-pentane); ν_{max} / cm^{-1} (film) 2941, 2866, 1490, 1205, 1093, 881, 698; ^1H NMR (500 MHz, $CDCl_3$) δ_H: 0.99 (9H, d, *J* 6.7), 1.04 (9H, d, *J* 6.5),1.06-1.12 (3H, m), 1.43 (3H, d, *J* 6.3), 4.95 (1H, q, *J* 6.3), 7.19-7.23 (1H, m), 7.28-7.32 (2H, m), 7.34-7.36 (2H, m); $^{13}C\{^1H\}$ NMR (126 MHz, $CDCl_3$) δ_c: 12.4, 18.1, 18.2, 28.0, 71.2, 125.4, 126.8, 128.2, 147.5; HRMS (ASAP$^+$) calculated for $[C_{17}H_{29}OSi]^+$ $(M–H)^+$: m/z 277.1988, found 277.1992 (+1.4 ppm).

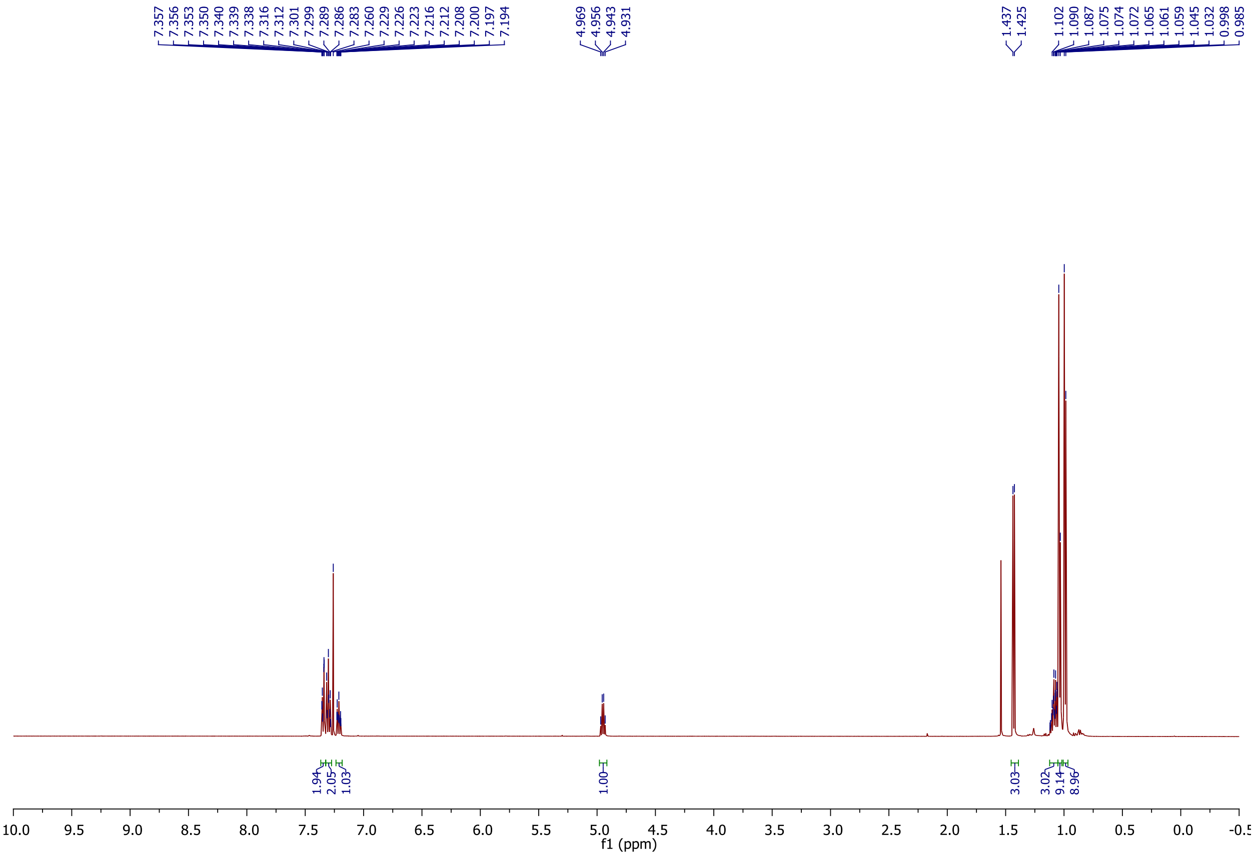
7.357
7.356
7.353
7.350
7.340
7.339
7.338
7.316
7.312
7.301
7.299
7.289
7.286
7.283
7.260
7.229
7.226
7.223
7.216
7.212
7.208
7.200
7.197
7.194
4.969
4.956
4.943
4.931
1.437
1.425
1.102
1.090
1.087
1.075
1.074
1.072
1.065
1.061
1.059
1.045
1.032
0.998
0.985
1.94
2.05
1.03
1.00
3.03
3.02
9.14
8.96
f1 (ppm)

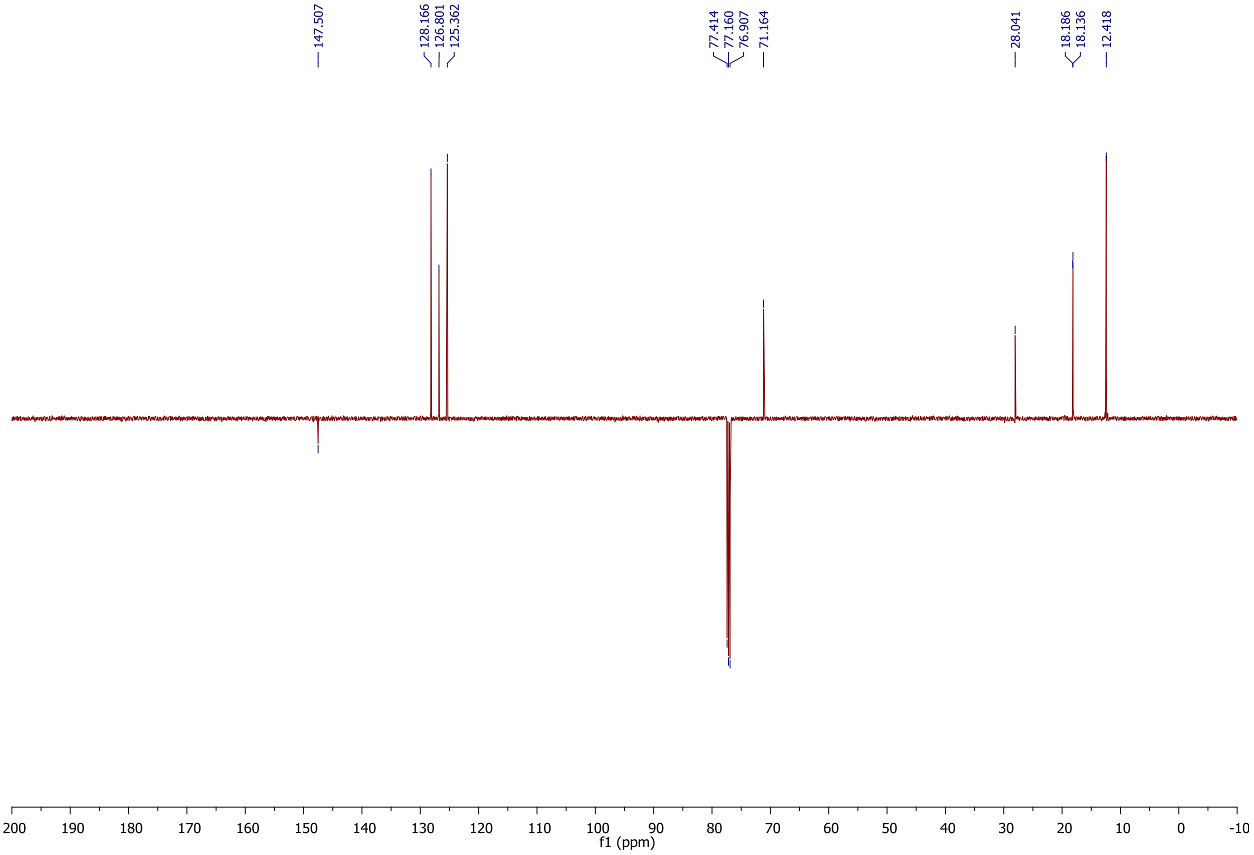
147.507
128.166
126.801
125.362
77.414
77.160
76.907
71.164
28.041
18.186
18.136
12.418
f1 (ppm)

tert-Butyldiphenyl(1-phenylethoxy)silane

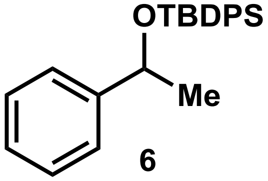

6

The title compound *tert*-butyldiphenyl(1-phenylethoxy)silane **6** was prepared according to general procedure 3 from *tert*-butyldiphenyl((1-phenylvinyl)oxy)silane (179 mg, 0.50 mmol, 1 equiv), tris(pentafluorophenyl)borane (25.6 mg, 0.05 mmol, 10 mol %), 2,2,6,6-tetramethylpiperidine (8.4 μL, 0.05 mmol, 10 mol %) and γ-terpinene (104 μL, 0.65 mmol, 1.3 equiv) and purified by flash silica column chromatography (eluent = 100% petroleum ether) to give a colorless oil (134 mg, 74% yield). R_f = 0.24 (eluent = 100% *n*-pentane); ν_{max} / cm^{-1} (film) 2926, 2856, 1427, 1369, 1087, 821, 700, 505; ^1H NMR (500 MHz, $CDCl_3$) δ_H: 1.06 (9H, s), 1.31 (3H, d, *J* 6.3), 4.83 (1H, q, *J* 6.3), 7.18-7.21 (1H, m), 7.22-7.28 (6H, m), 7.32-7.38 (3H, m), 7.40-7.44 (1H, m), 7.48-7.51 (2H, m), 7.69-7.72 (2H, m); $^{13}C\{^1H\}$ NMR (126 MHz, $CDCl_3$) δ_C: 19.4, 27.1, 27.3, 72.0, 125.5, 126.9, 127.5, 128.2, 129.6, 129.7, 133.9, 134.7, 136.0, 136.0, 146.6; HRMS ($ASAP^+$) calculated for $[C_{24}H_{27}OSi]^+$ $(M–H)^+$: m/z 359.1831, found 359.1829 (-0.6 ppm).

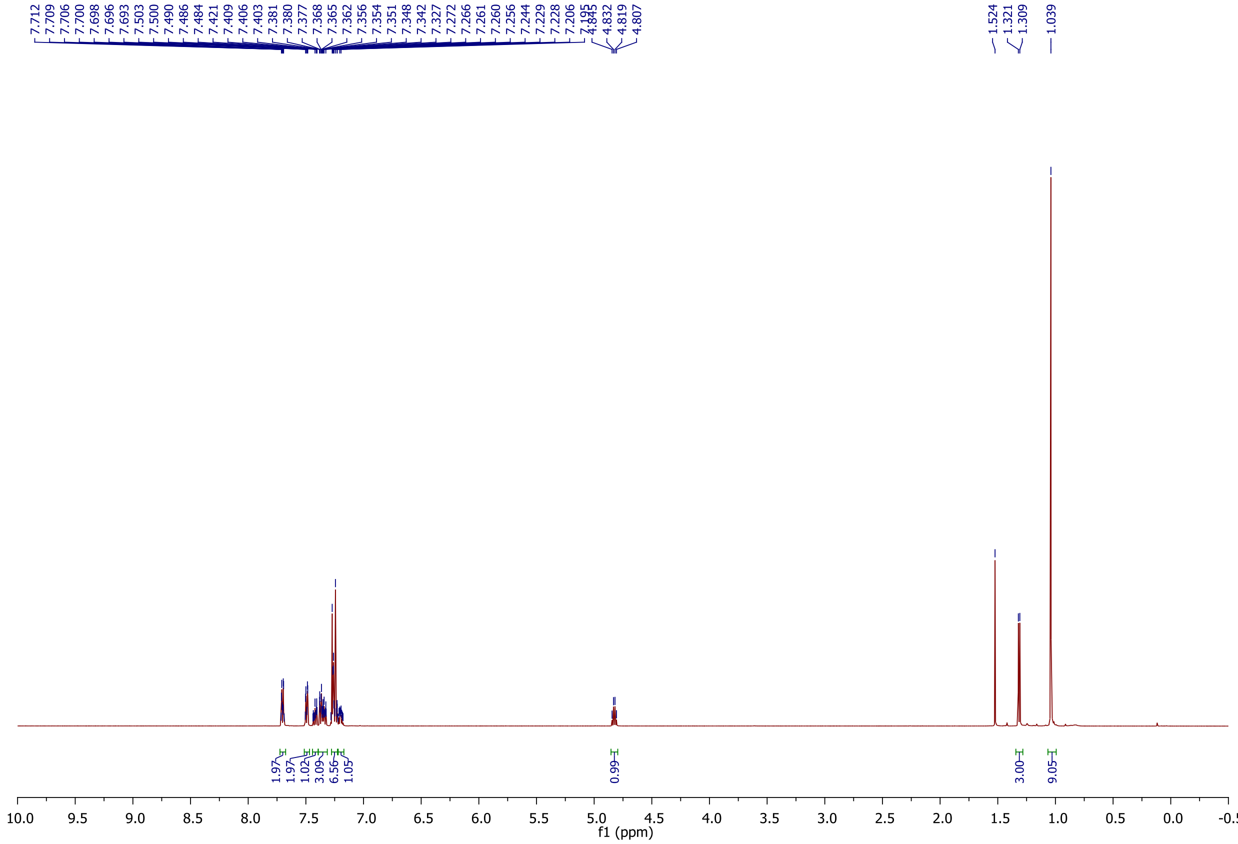

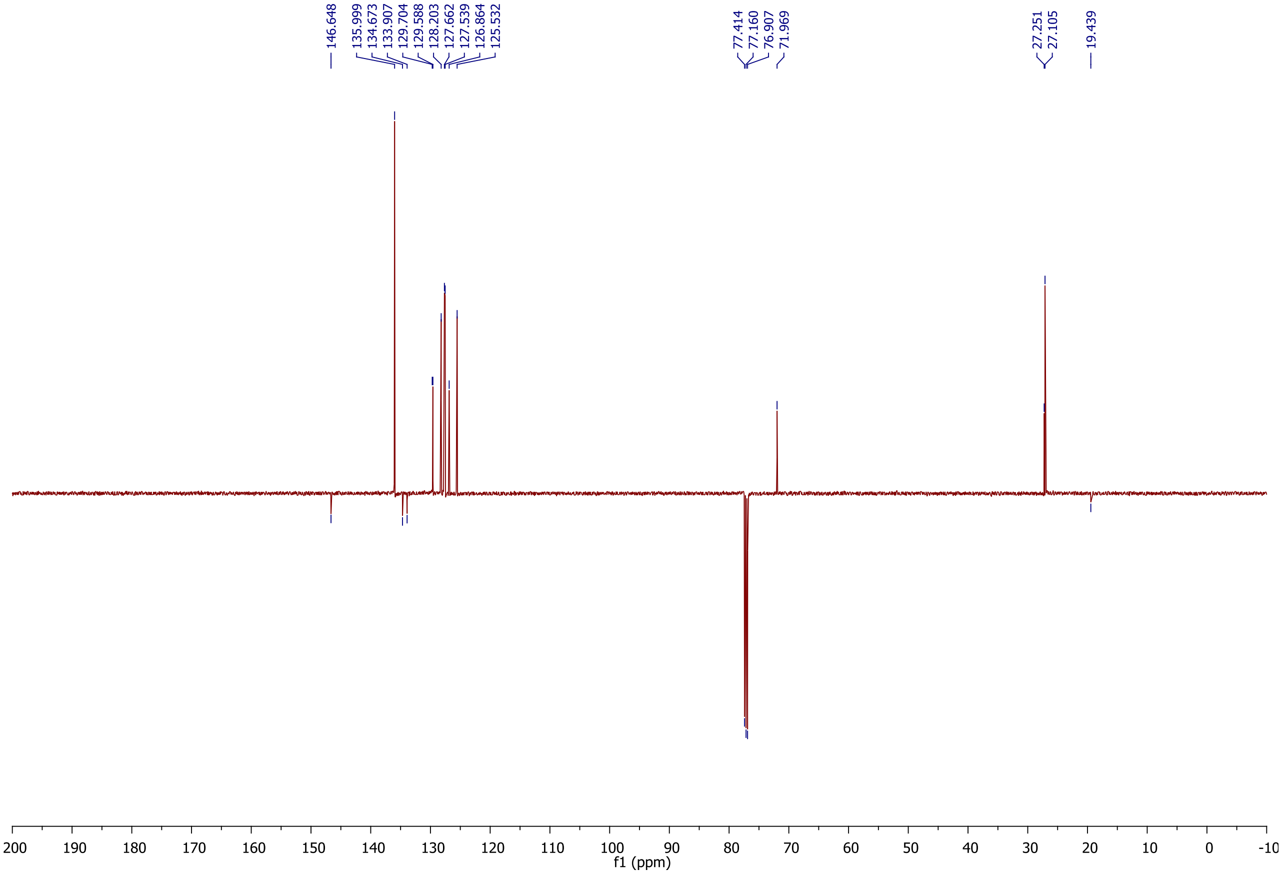

1-(*p*-Tolyl)ethan-1-ol

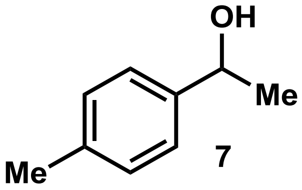

7

The title compound 1-(*p*-tolyl)ethan-1-ol **7** was prepared according to general procedure 4 from *tert*-butyldimethyl((1-(*p*-tolyl)vinyl)oxy)silane (124 mg, 0.50 mmol, 1 equiv), tris(pentafluorophenyl)borane (25.6 mg, 0.05 mmol, 10 mol %), 2,2,6,6-tetramethylpiperidine (8.4 µL, 0.05 mmol, 10 mol %) and γ-terpinene (104 µL, 0.65 mmol, 1.3 equiv), followed by TBAF (0.50 mmol, 0.5 mL, 1 equiv) and purified by flash silica column chromatography (eluent = 10% EtOAc in hexanes) to give a colorless oil (50 mg, 74% yield). R_f = 0.22 (eluent = 20% EtOAc in hexanes); ν_{max} / cm^{-1} (film) 3334, 2970, 1512, 1367, 1201, 1072, 815, 542; ^{1}H NMR (500 MHz, $CDCl_3$) δ_H: 1.48 (3H, t, *J* 6.5), 2.05 (1H, br s), 2.36 (3H, s), 4.85 (1H, q, *J* 6.5), 7.17 (2H, d, *J* 7.8), 7.25-7.28 (2H, m); $^{13}C\{^{1}H\}$ NMR (126 MHz, $CDCl_3$) δ_c: 21.2, 25.2, 70.3, 125.5, 129.3, 137.2, 143.0; HRMS (CI^+) calculated for $[C_9H_{16}ON]^+$ $(M+NH_4)^+$: m/z 154.1226, found 154.1225 (-0.9 ppm).

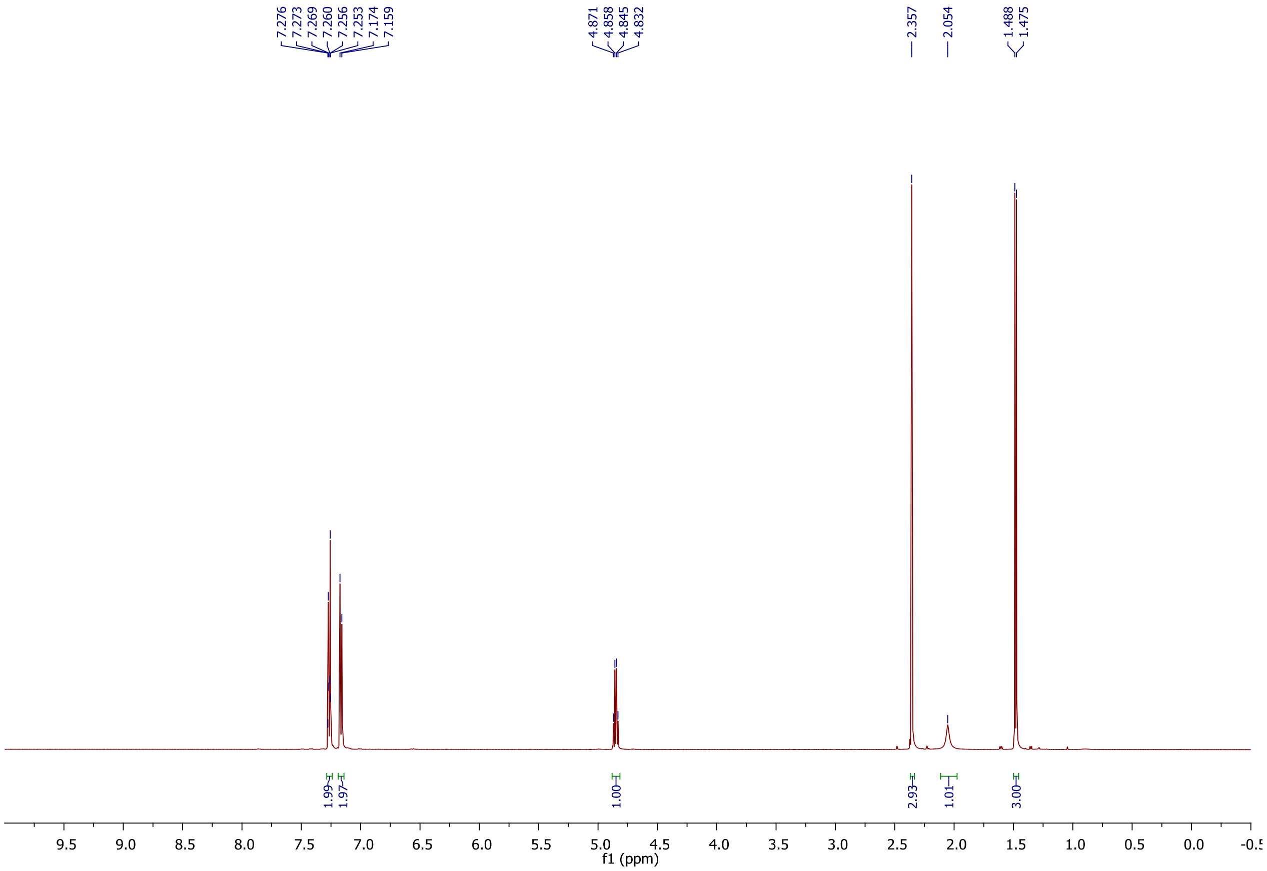

7.276
7.273
7.269
7.260
7.256
7.253
7.174
7.159
4.871
4.858
4.845
4.832
2.357
2.054
1.488
1.475
1.99
1.97
1.00
2.93
1.01
3.00
9.5 9.0 8.5 8.0 7.5 7.0 6.5 6.0 5.5 5.0 4.5 4.0 3.5 3.0 2.5 2.0 1.5 1.0 0.5 0.0
f1 (ppm)

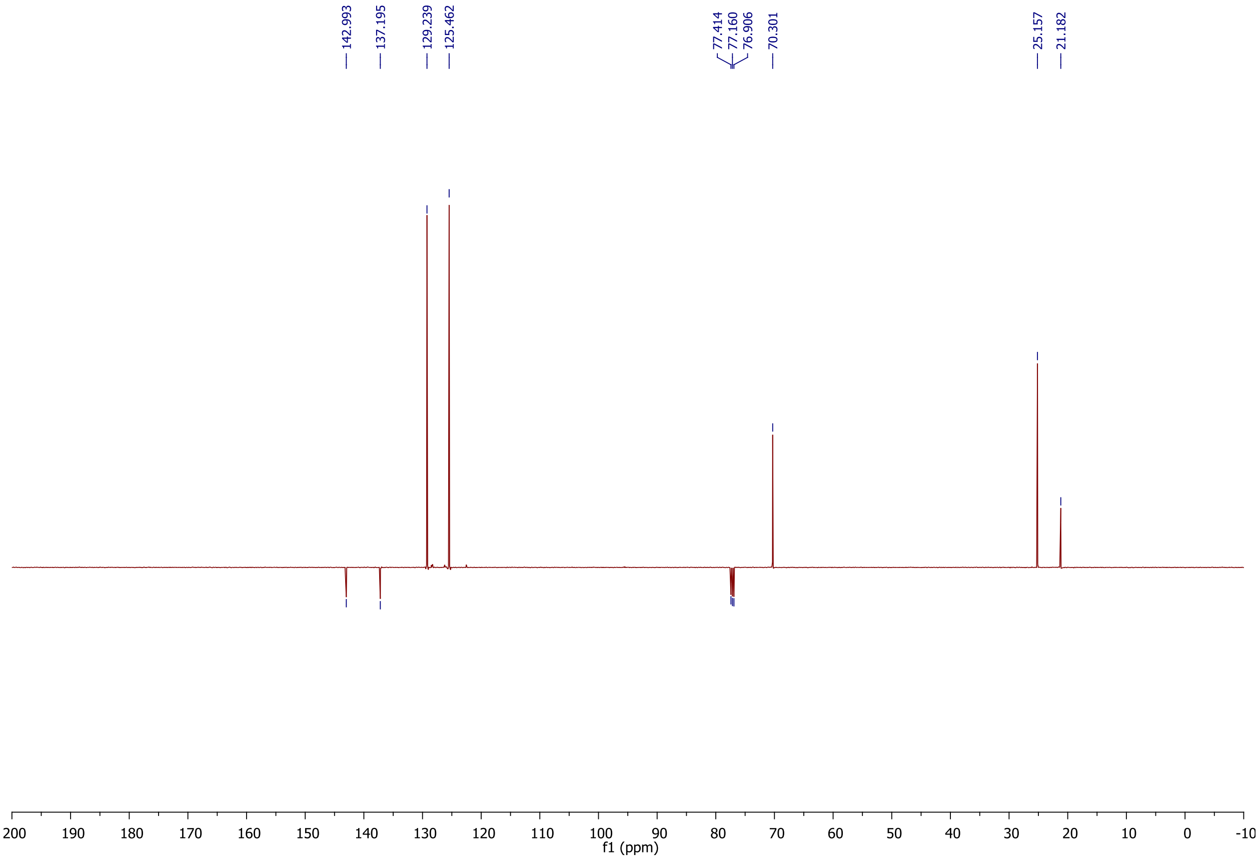

142.993
137.195
129.239
125.462
77.414
77.160
76.906
70.301
25.157
21.182
200 190 180 170 160 150 140 130 120 110 100 90 80 70 60 50 40 30 20 10 0 -10
f1 (ppm)

1-(*m*-Tolyl)ethan-1-ol

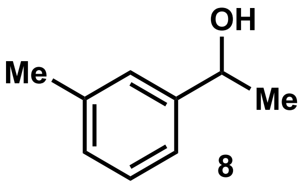

8

The title compound 1-(*m*-tolyl)ethan-1-ol **8** was prepared according to general procedure 4 from *tert*-butyldimethyl((1-(*m*-tolyl)vinyl)oxy)silane (124 mg, 0.50 mmol, 1 equiv), tris(pentafluorophenyl)borane (25.6 mg, 0.05 mmol, 10 mol %), 2,2,6,6-tetramethylpiperidine (8.4 µL, 0.05 mmol, 10 mol %) and γ-terpinene (104 µL, 0.65 mmol, 1.3 equiv), followed by TBAF (0.50 mmol, 0.5 mL, 1 equiv) and purified by flash silica column chromatography (eluent = 10% EtOAc in hexanes) to give a light yellow oil (57 mg, 84% yield). R_f = 0.32 (eluent = 20% EtOAc in hexanes); ν_{max} / cm^{-1} (film) 3336, 2980, 1608, 1489, 1369, 1159, 1072, 785, 702; ^1H NMR (500 MHz, $CDCl_3$) δ_H: 1.49 (3H, t, *J* 6.5), 1.94 (1H, br s), 2.37 (3H, s), 4.86 (1H, q, *J* 6.5), 7.10 (1H, d, *J* 7.5), 7.16-7.18 (1H, m), 7.20-7.21 (1H, m), 7.23-7.26 (1H, m); $^{13}C\{^1H\}$ NMR (126 MHz, $CDCl_3$) δ_c: 21.6, 25.2, 70.5, 122.5, 126.2, 128.3, 128.5, 138.3, 145.9; HRMS (CI^+) calculated for $[C_9H_{16}ON]^+$ $(M+NH_4)^+$: m/z 154.1226, found 154.1228 (+1.0 ppm).

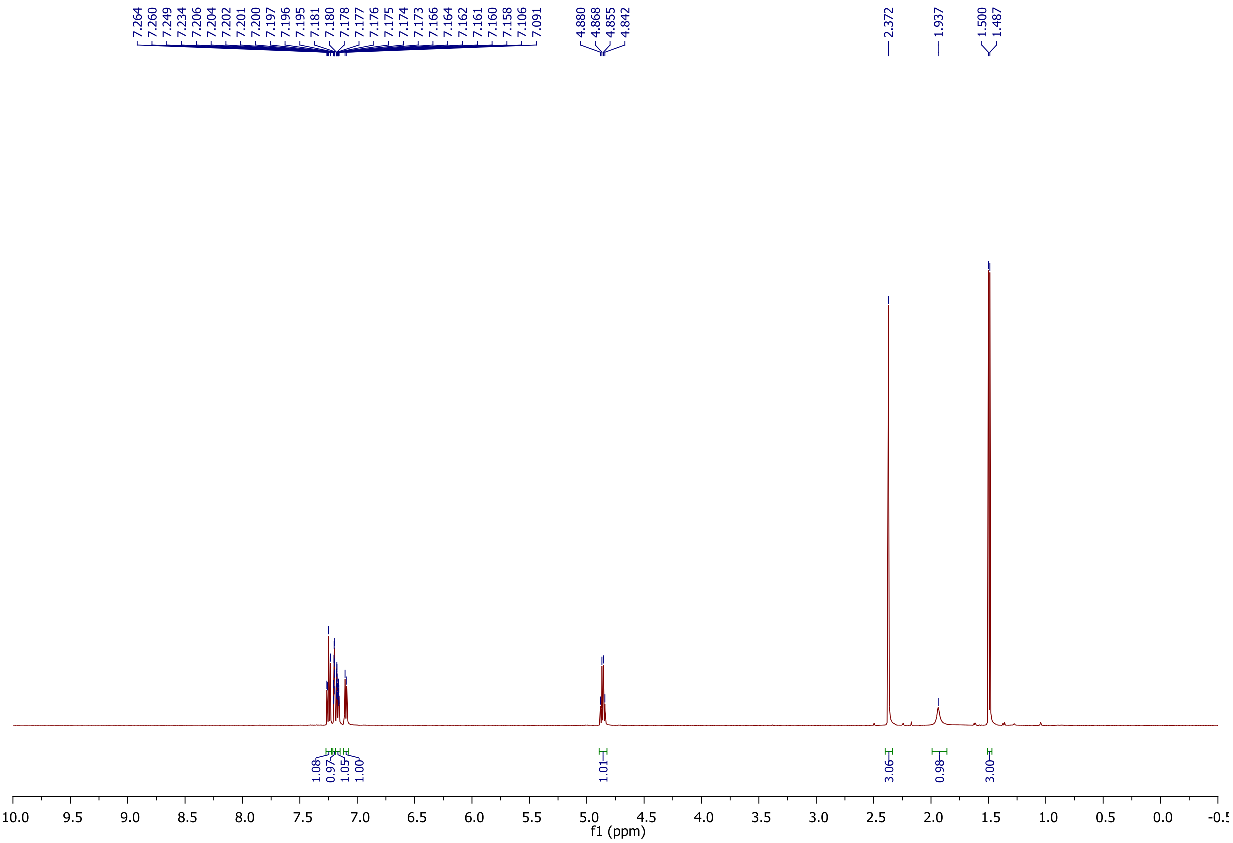

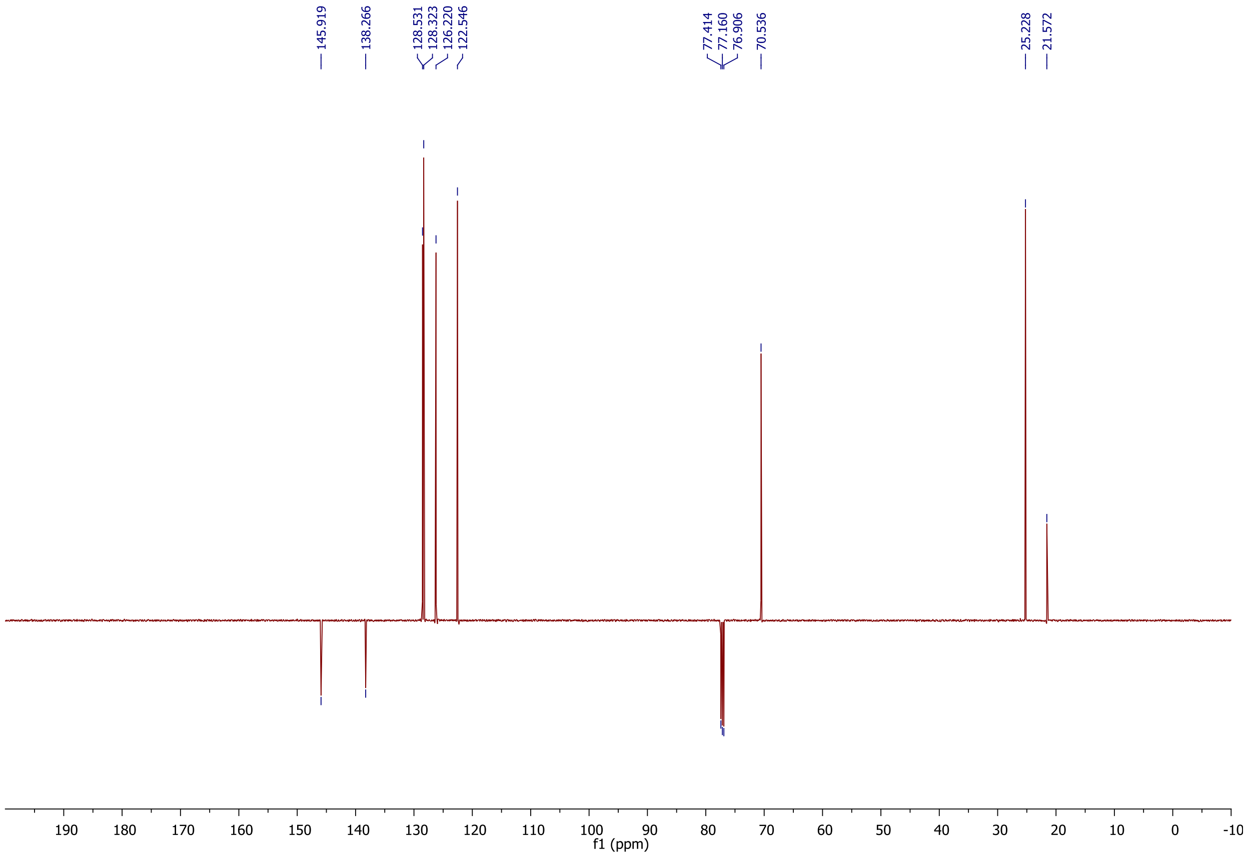

1-(*o*-Tolyl)ethan-1-ol

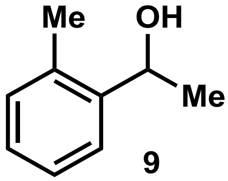

9

The title compound 1-(*o*-tolyl)ethan-1-ol **9** was prepared according to general procedure 3 from trimethyl((1-(*o*-tolyl)vinyl)oxy)silane (103 mg, 0.50 mmol, 1 equiv), tris(pentafluorophenyl)borane (25.6 mg, 0.05 mmol, 10 mol %), 2,2,6,6-tetramethylpiperidine (8.4 µL, 0.05 mmol, 10 mol %) and γ-terpinene (104 µL, 0.65 mmol, 1.3 equiv), followed by TBAF (0.50 mmol, 0.5 mL, 1 equiv) and purified by flash silica column chromatography (eluent = 10% EtOAc in hexanes) to give a colorless oil (61 mg, 90% yield). R_f = 0.35 (eluent = 20% EtOAc in hexanes); ν_{max} / cm^{-1} (film) 3321, 2970, 1489, 1215, 1074, 758, 557; ^{1}H NMR (500 MHz, $CDCl_3$) δ_H: 1.47 (3H, t, *J* 6.4), 2.00 (1H, br s), 2.35 (3H, s), 5.12 (1H, q, *J* 6.4), 7.14-7.15 (1H, m), 7.18 (1H, td, *J* 7.4, 1.4), 7.23-7.26 (1H, m), 7.52 (1H, dd, *J* 7.7, 0.9); $^{13}C\{^1H\}$ NMR (126 MHz, $CDCl_3$) δ_c: 19.0, 24.0, 66.9, 124.6, 126.5, 127.2, 130.5, 134.3, 144.0; HRMS (CI^+) calculated for $[C_9H_{16}ON]^+$ $(M+NH_4)^+$: m/z 154.1226, found 154.1228 (+1.0 ppm).

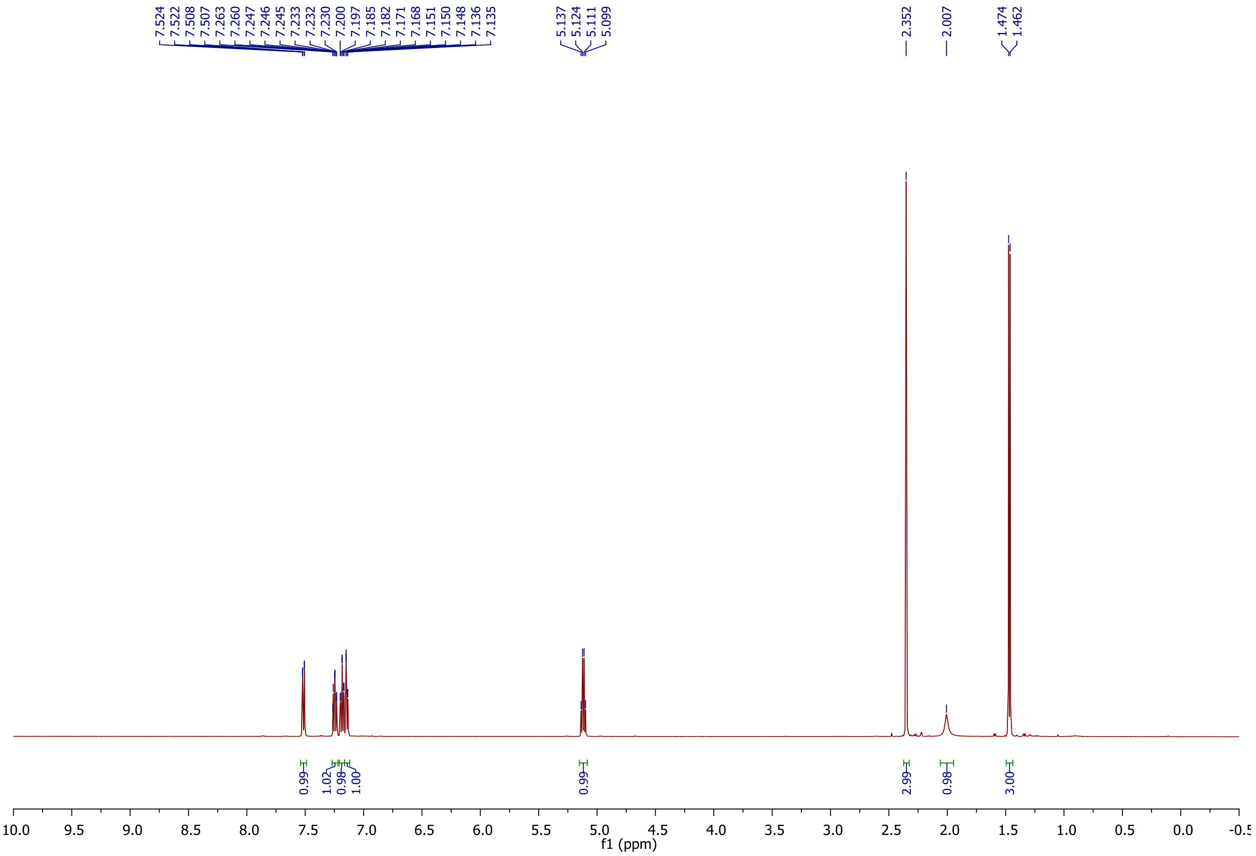

7.524 7.522 7.508 7.507 7.263 7.260 7.247 7.246 7.245 7.233 7.232 7.230 7.200 7.197 7.185 7.182 7.171 7.168 7.151 7.150 7.148 7.136 7.135
5.137 5.124 5.111 5.099
2.352
2.007
1.474 1.462
0.99 1.02 0.98 1.00 0.99 2.99 0.98 3.00
10.0 9.5 9.0 8.5 8.0 7.5 7.0 6.5 6.0 5.5 5.0 4.5 4.0 3.5 3.0 2.5 2.0 1.5 1.0 0.5 0.0 -0.5
f1 (ppm)

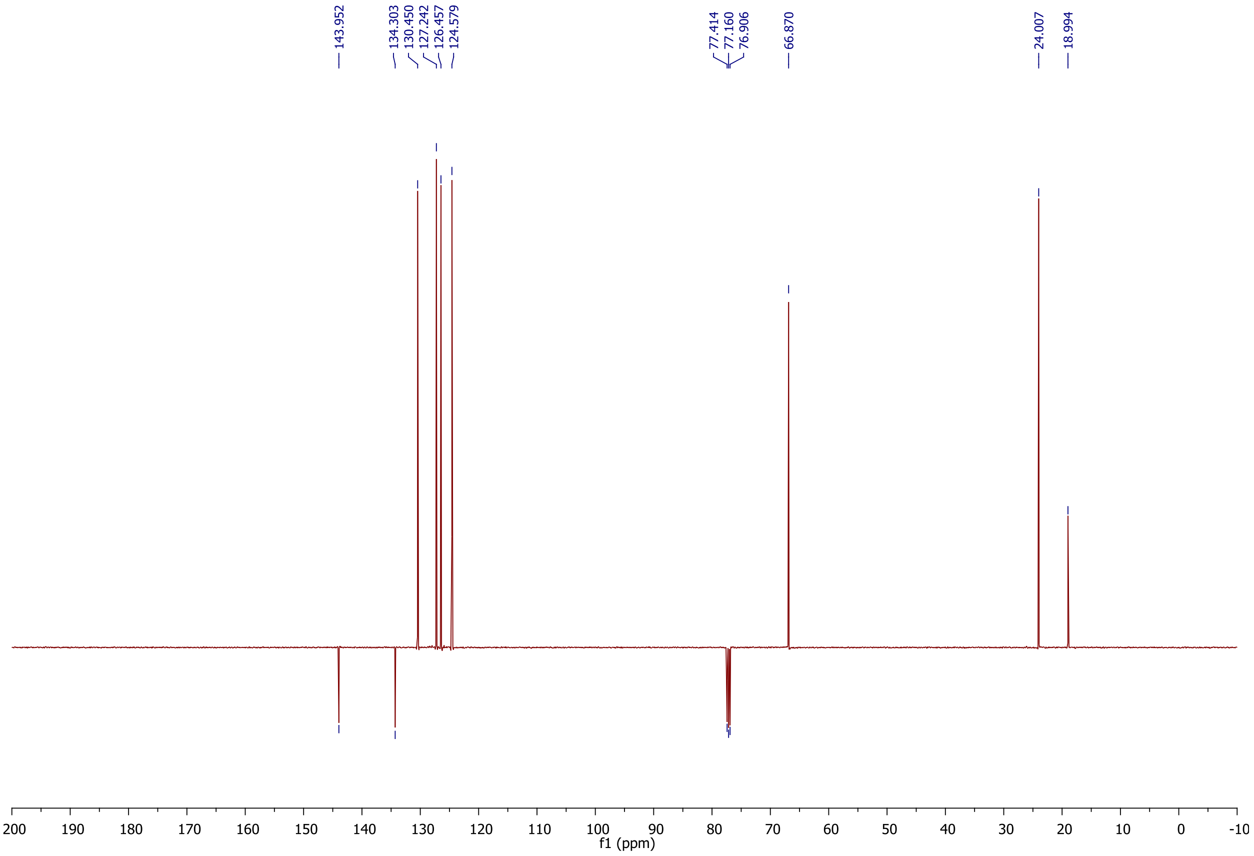

143.952
134.303 130.450 127.242 126.457 124.579
77.414 77.160 76.906
66.870
24.007
18.994
200 190 180 170 160 150 140 130 120 110 100 90 80 70 60 50 40 30 20 10 0 -10
f1 (ppm)

1-(3,4-Dimethylphenyl)ethan-1-ol

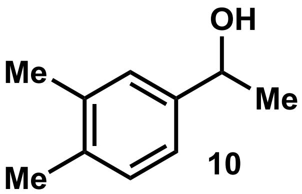

10

The title compound 1-(3,4-dimethylphenyl)ethan-1-ol **10** was prepared according to general procedure 4 from *tert*-butyl((1-(3,4-dimethylphenyl)vinyl)oxy)dimethylsilane (131 mg, 0.50 mmol, 1 equiv), tris(pentafluorophenyl)borane (25.6 mg, 0.05 mmol, 10 mol %), 2,2,6,6-tetramethylpiperidine (8.4 μL, 0.05 mmol, 10 mol %) and γ-terpinene (104 μL, 0.65 mmol, 1.3 equiv), followed by TBAF (0.50 mmol, 0.5 mL, 1 equiv) and purified by flash silica column chromatography (eluent = 10% EtOAc in hexanes) to give a colorless oil (64 mg, 85% yield). R_f = 0.33 (eluent = 20% EtOAc in hexanes); ν_{max} / cm^{-1} (film) 3336, 2970, 1506, 1448, 1157, 1072, 819, 731; ^{1}H NMR (500 MHz, $CDCl_3$) δ_H: 1.49 (3H, t, *J* 6.5), 1.91 (1H, br s), 2.27 (3H, s), 2.29 (3H, s), 4.84 (1H, q, *J* 6.5), 7.10-7.14 (2H, m), 7.16 (1H, br s); $^{13}C\{^{1}H\}$ NMR (126 MHz, $CDCl_3$) δ_c: 19.5, 19.9, 25.2, 70.4, 122.9, 126.9, 129.8, 135.9, 136.8, 143.5; HRMS (CI^+) calculated for $[C_{10}H_{16}ON]^+$ $(M\text{-}H\text{+}NH_3)^+$: m/z 166.1226, found 166.1220 (-3.9 ppm).

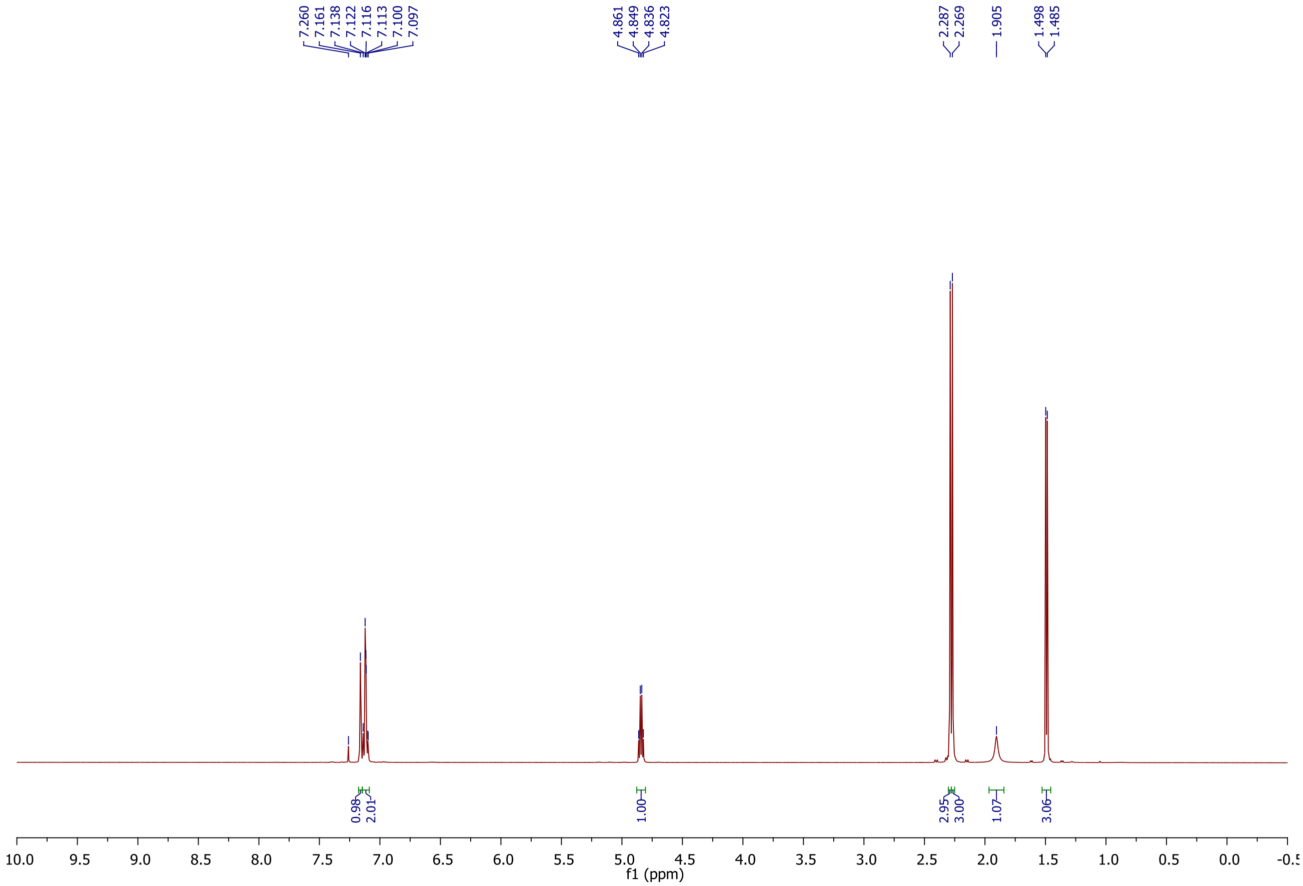

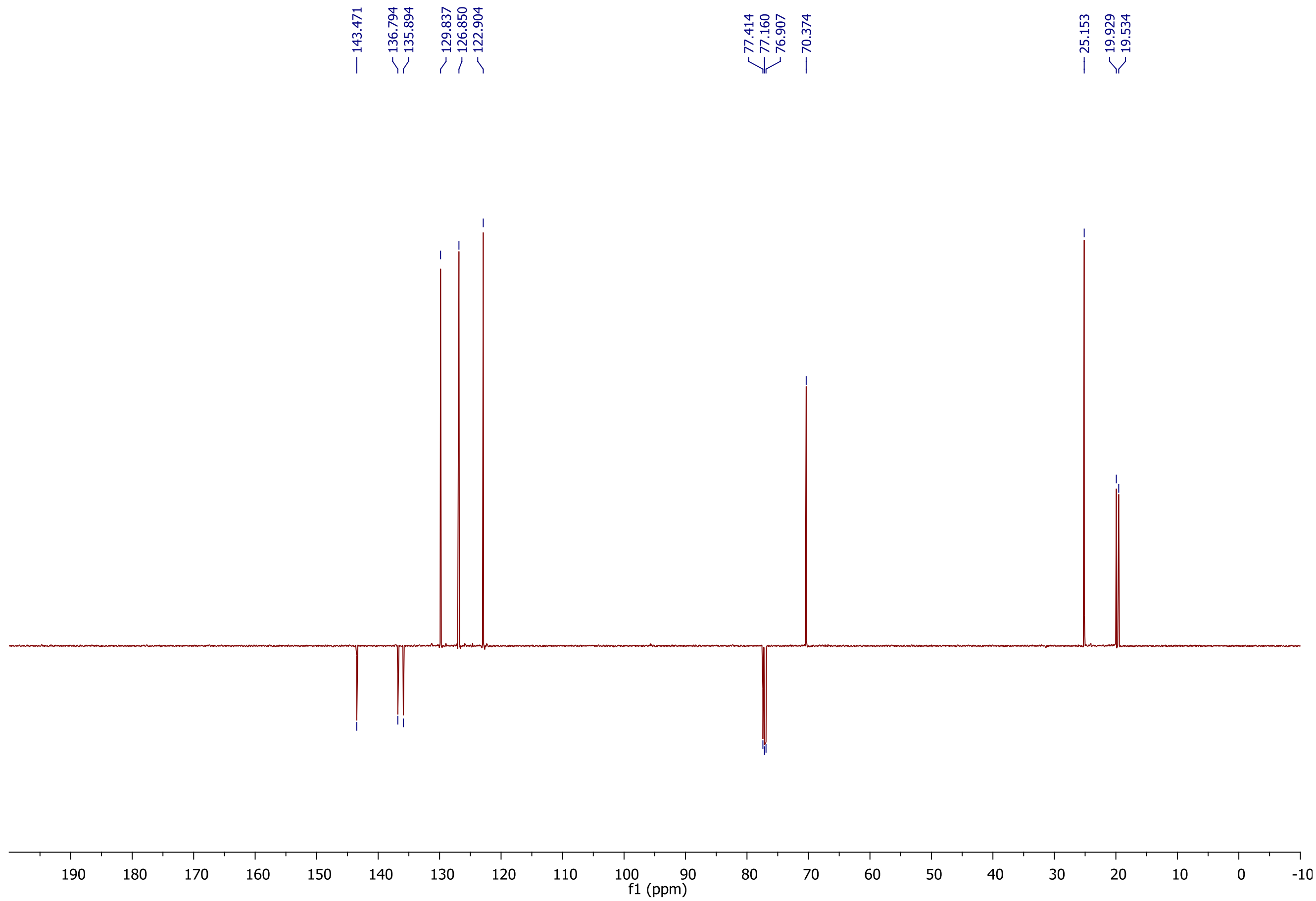

1-(5,6,7,8-Tetrahydronaphthalen-2-yl)ethan-1-ol

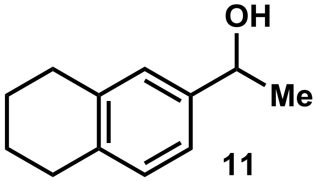

11

The title compound 1-(5,6,7,8-tetrahydronaphthalen-2-yl)ethan-1-ol **11** was prepared according to general procedure 4 from *tert*-butyldimethyl((1-(5,6,7,8-tetrahydronaphthalen-2-yl)vinyl)oxy)silane (144 mg, 0.50 mmol, 1 equiv), tris(pentafluorophenyl)borane (25.6 mg, 0.05 mmol, 10 mol %), 2,2,6,6-tetramethylpiperidine (8.4 µL, 0.05 mmol, 10 mol %) and γ-terpinene (104 µL, 0.65 mmol, 1.3 equiv), followed by TBAF (0.50 mmol, 0.5 mL, 1 equiv) and purified by flash silica column chromatography (eluent = 10% EtOAc in hexanes) to give a colorless oil (78 mg, 89% yield). R_f = 0.39 (eluent = 20% EtOAc in hexanes); ν_{max} / cm^{-1} (film) 3334, 2980, 2926, 1500, 1436, 1155, 1072, 827, 732; ^{1}H NMR (500 MHz, $CDCl_3$) δ_H: 1.51 (3H, t, *J* 6.5), 1.82-1.85 (4H, m), 2.16 (1H, br s), 2.78-2.82 (4H, m), 4.83 (1H, q, *J* 6.5), 7.07-7.13 (3H, m); $^{13}C\{^{1}H\}$ NMR (126 MHz, $CDCl_3$) δ_c: 23.3, 23.3, 25.1, 29.2, 29.6, 70.3, 122.7,

126.2, 129.3, 136.4, 137.3, 143.1; HRMS (ASAP$^+$) calculated for $[C_{12}H_{15}O]^+$ (M–H)$^+$: m/z 175.1123, found 175.1124 (+0.6 ppm).

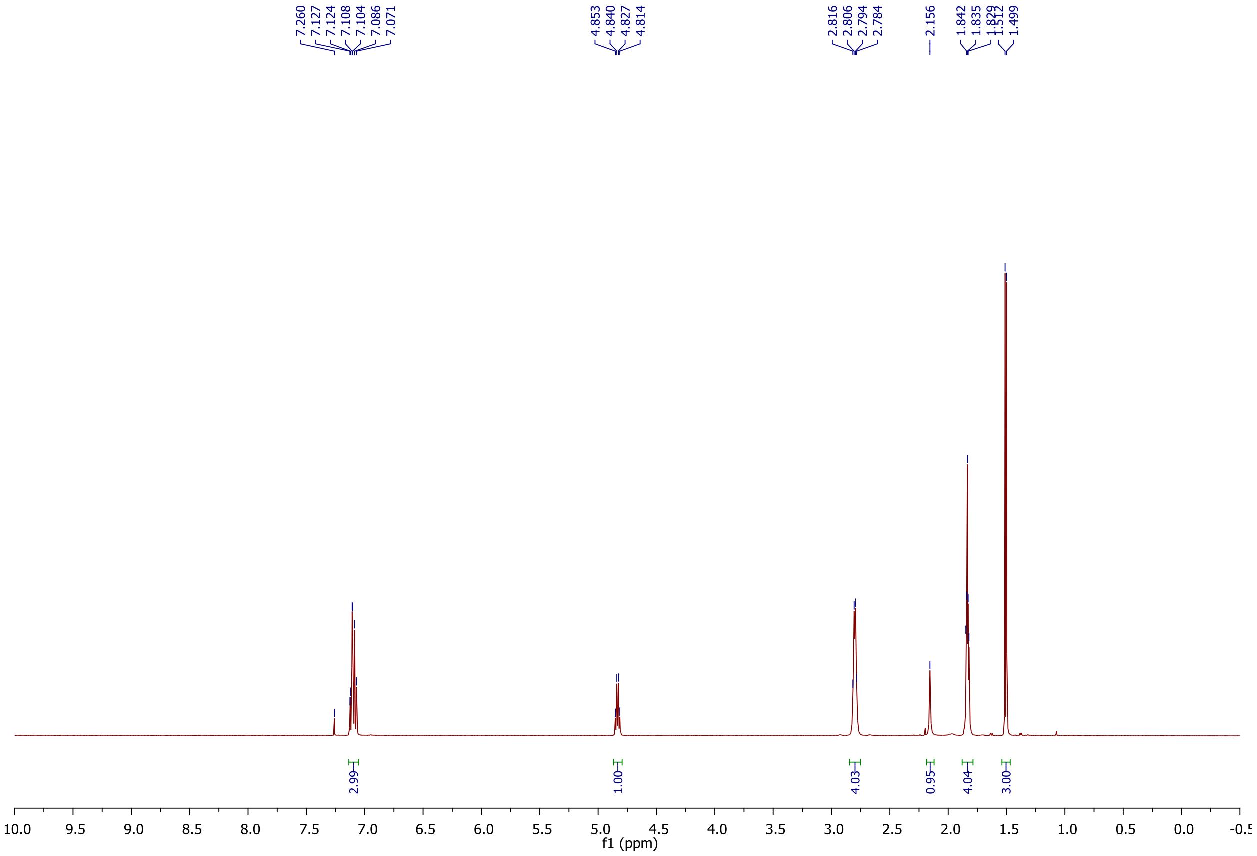

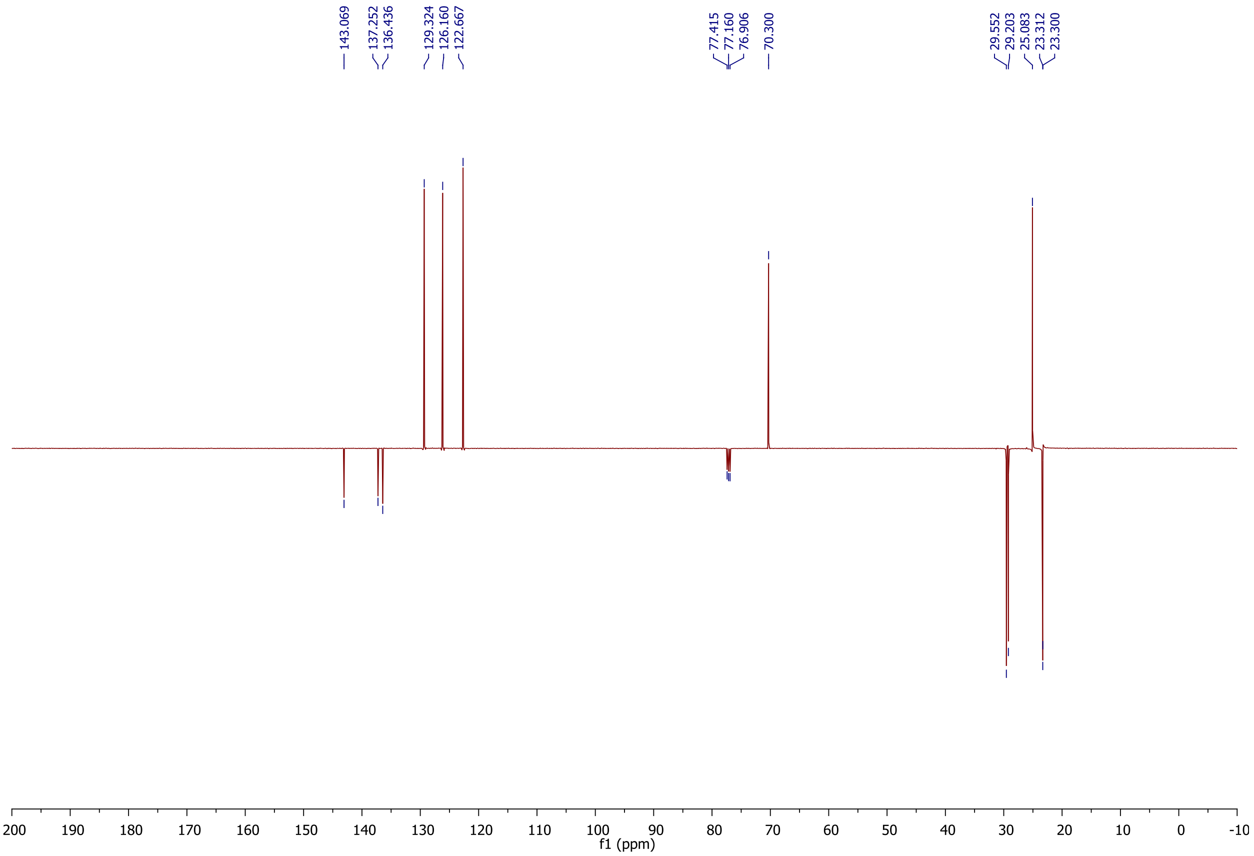

1-(4-Ethylphenyl)ethan-1-ol

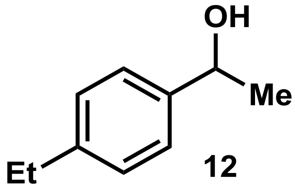

12

The title compound 1-(4-ethylphenyl)ethan-1-ol **12** was prepared according to general procedure 4 from *tert*-butyl((1-(4-ethylphenyl)vinyl)oxy)dimethylsilane (131 mg, 0.50 mmol, 1 equiv), tris(pentafluorophenyl)borane (25.6 mg, 0.05 mmol, 10 mol %), 2,2,6,6-tetramethylpiperidine (8.4 μL, 0.05 mmol, 10 mol %) and γ-terpinene (104 μL, 0.65 mmol, 1.3 equiv), followed by TBAF (0.50 mmol, 0.5 mL, 1 equiv) and purified by flash silica column chromatography (eluent = 8% EtOAc in hexanes) to give a colorless oil (64 mg, 85% yield). R_f = 0.41 (eluent = 20% EtOAc in hexanes); ν_{max} / cm^{-1} (film) 3331, 2964, 1512, 1369, 1072, 831, 732; ^{1}H NMR (500 MHz, $CDCl_3$) δ_H: 1.25 (3H, t, *J* 7.6), 1.49 (3H, d, *J* 6.5), 1.89 (1H, br s), 2.66 (2H, q, *J* 7.6), 4.87 (1H, q, *J* 6.5), 7.20 (2H, d, *J* 8.1), 7.30 (2H, d, *J* 8.1); ^{13}C{^{1}H} NMR (126 MHz, $CDCl_3$) δ_C: 15.7, 25.1, 28.6, 70.4, 125.6, 128.1, 143.2, 143.7; HRMS (ASAP$^+$) calculated for $[C_{10}H_{13}O]^+$ (M–H)$^+$: m/z 149.0966, found 149.0968 (+1.3 ppm).

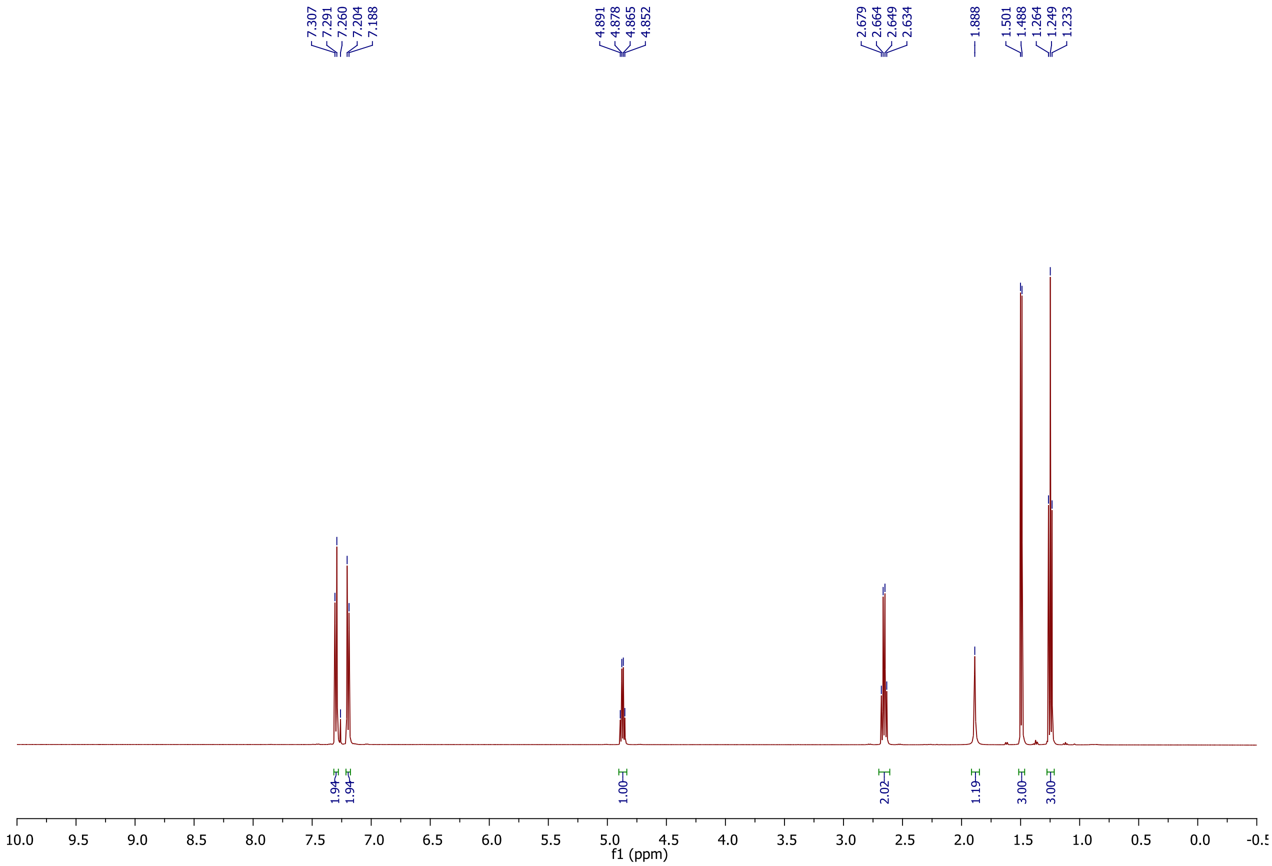
7.307
7.291
7.260
7.204
7.188
4.891
4.878
4.865
4.852
2.679
2.664
2.649
2.634
1.888
1.501
1.488
1.264
1.249
1.233
1.94
1.94
1.00
2.02
1.19
3.00
3.00
10.0 9.5 9.0 8.5 8.0 7.5 7.0 6.5 6.0 5.5 5.0 4.5 4.0 3.5 3.0 2.5 2.0 1.5 1.0 0.5 0.0
f1 (ppm)

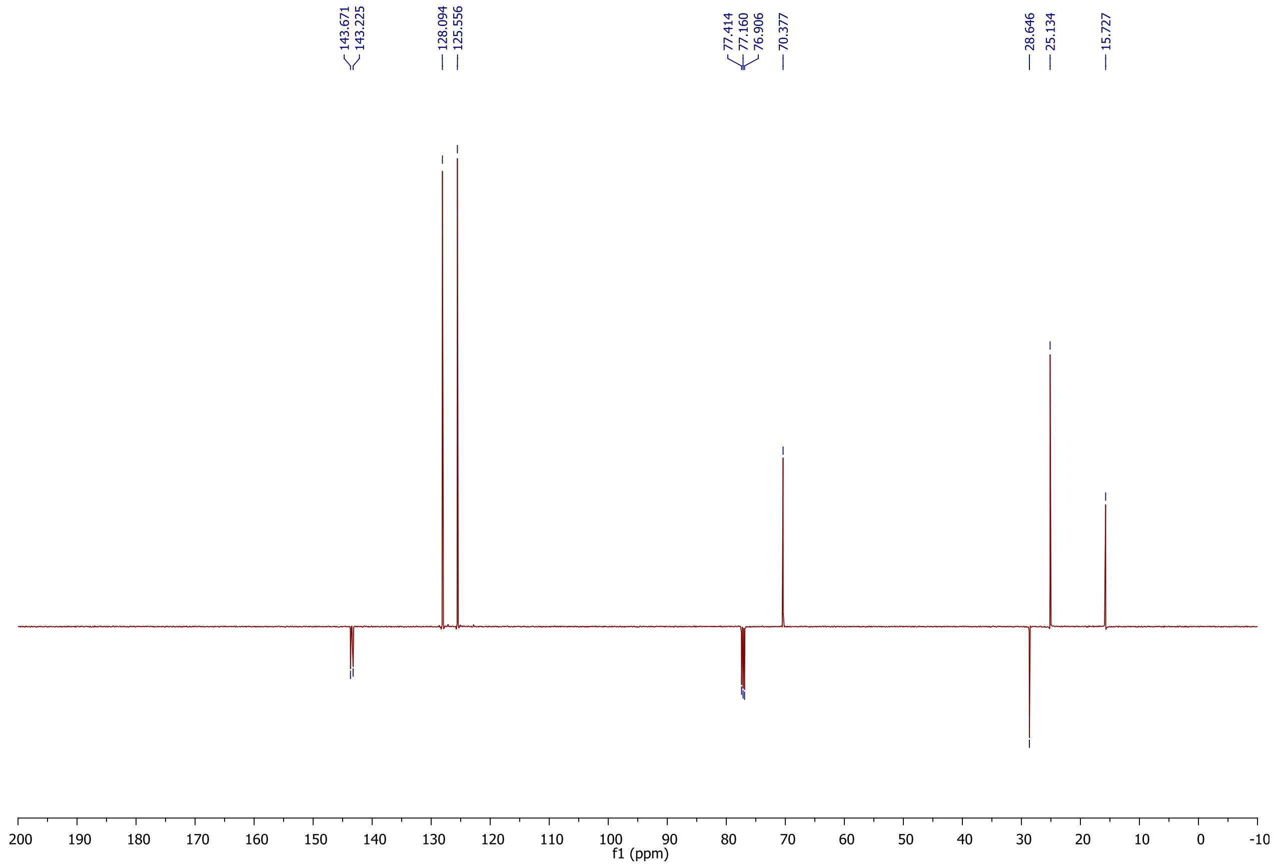
143.671
143.225
128.094
125.556
77.414
77.160
76.906
70.377
28.646
25.134
15.727
200 190 180 170 160 150 140 130 120 110 100 90 80 70 60 50 40 30 20 10 0 -10
f1 (ppm)

1-([1,1'-Biphenyl]-4-yl)ethan-1-ol

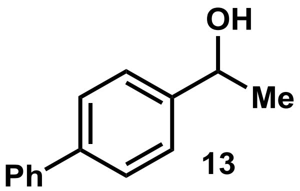

13

The title compound 1-([1,1'-biphenyl]-4-yl)ethan-1-ol **13** was prepared according to general procedure 4 from ((1-([1,1'-biphenyl]-4-yl)vinyl)oxy)(*tert*-butyl)dimethylsilane (155 mg, 0.50 mmol, 1 equiv), tris(pentafluorophenyl)borane (25.6 mg, 0.05 mmol, 10 mol %), 2,2,6,6-tetramethylpiperidine (8.4 µL, 0.05 mmol, 10 mol %) and γ-terpinene (104 µL, 0.65 mmol, 1.3 equiv), followed by TBAF (0.50 mmol, 0.5 mL, 1 equiv) and purified by flash silica column chromatography (eluent = 10% EtOAc in hexanes) to give a white solid (88 mg, 89% yield). mp 105-106 °C; R_f = 0.28 (eluent = 20% EtOAc in hexanes); ν_{max} / cm^{-1} (film) 3300, 2970, 1483, 1193, 1068, 833, 759, 686, 505; ^1H NMR (500 MHz, $CDCl_3$) δ_H: 1.55 (3H, t, *J* 6.5), 1.99 (1H, br s), 4.96 (1H, q, *J* 6.5), 7.34-7.38 (1H, m), 7.44-7.48 (4H, m), 7.59-7.61 (4H, m); $^{13}C\{^1H\}$ NMR (126 MHz, $CDCl_3$) δ_c: 25.3, 70.3, 126.0, 127.2, 127.4, 127.4, 128.9, 140.6, 141.0, 144.9; HRMS (ASAP$^+$) calculated for $[C_{14}H_{13}O]^+$ $(M–H)^+$: m/z 197.0966, found 197.0971 (+2.5 ppm).

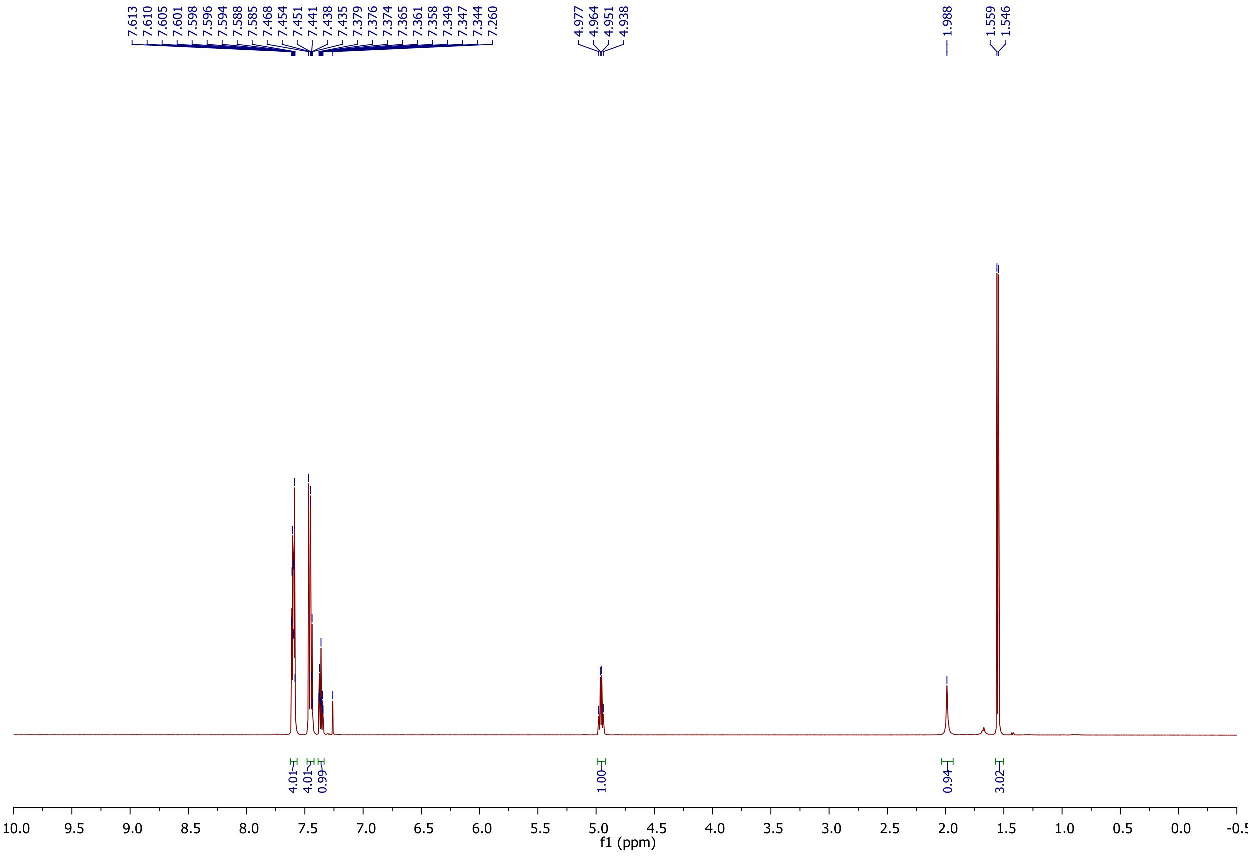
7.613
7.610
7.605
7.601
7.598
7.596
7.594
7.588
7.585
7.468
7.454
7.451
7.441
7.438
7.435
7.379
7.376
7.374
7.365
7.361
7.358
7.349
7.347
7.344
7.260
4.977
4.964
4.951
4.938
1.988
1.559
1.546
4.01
4.01
0.99
1.00
0.94
3.02
f1 (ppm)

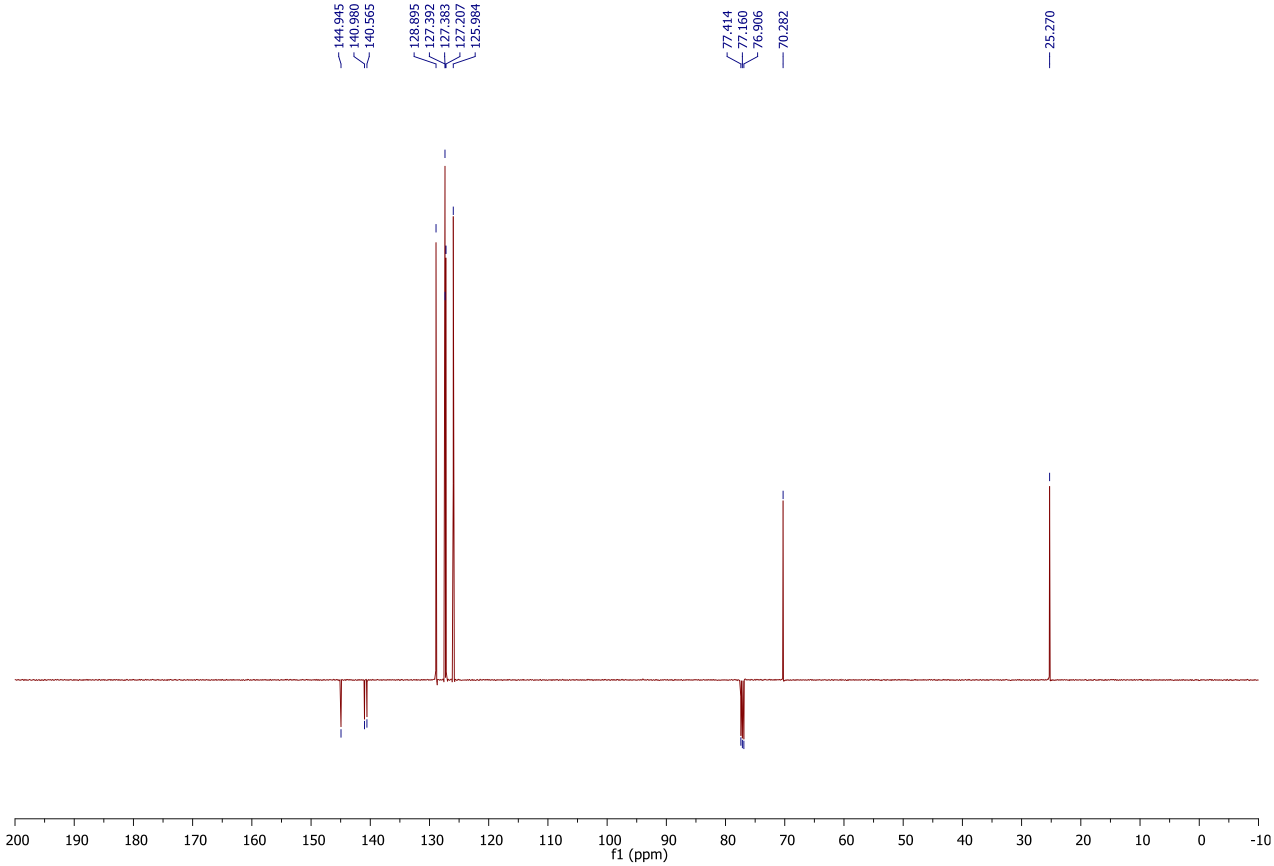
144.945
140.980
140.565
128.895
127.392
127.383
127.207
125.984
77.414
77.160
76.906
70.282
25.270
f1 (ppm)

1-(4-Methoxyphenyl)ethan-1-ol

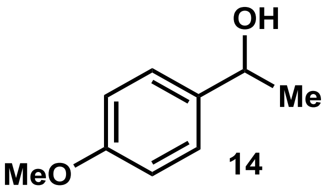

14

The title compound 1-(4-methoxyphenyl)ethan-1-ol **14** was prepared according to general procedure 4 from *tert*-butyl((1-(4-methoxyphenyl)vinyl)oxy)dimethylsilane (132 mg, 0.50 mmol, 1 equiv), tris(pentafluorophenyl)borane (25.6 mg, 0.05 mmol, 10 mol %), 2,2,6,6-tetramethylpiperidine (8.4 μL, 0.05 mmol, 10 mol %) and γ-terpinene (104 μL, 0.65 mmol, 1.3 equiv), followed by TBAF (0.50 mmol, 0.5 mL, 1 equiv) and purified by flash silica column chromatography (eluent = 8→12% EtOAc in hexanes) to give a colorless oil (61 mg, 91% yield). R_f = 0.23 (eluent = 20% EtOAc in hexanes); ν_{max} / cm^{-1} (film) 3367, 2970, 1610, 1510, 1240, 1174, 1031, 829, 547; ^{1}H NMR (500 MHz, CD_3OD) δ_H: 1.41 (3H, t, *J* 6.5), 3.77 (3H, s), 4.85 (1H, br s), 4.77 (1H, q, *J* 6.5), 6.86-6.89 (2H, m), 7.26-7.29 (2H, m); $^{13}C\{^1H\}$ NMR (126 MHz, CD_3OD) δ_C: 25.5, 55.7, 70.5, 114.6, 127.7, 139.6, 160.3; HRMS (ASAP$^+$) calculated for $[C_9H_{11}O_2]^+$ $(M–H)^+$: m/z 151.0759, found 151.0761 (+1.3 ppm).

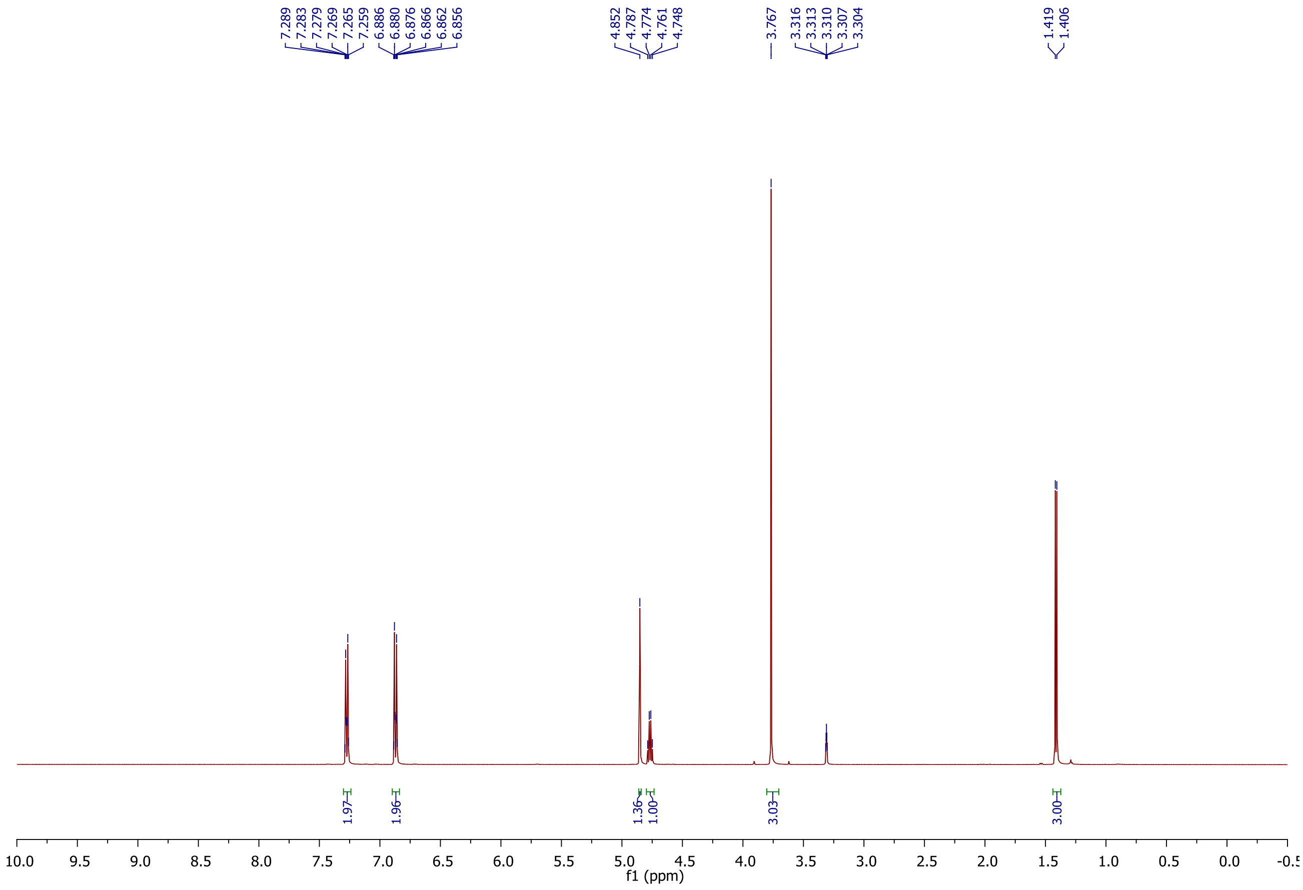

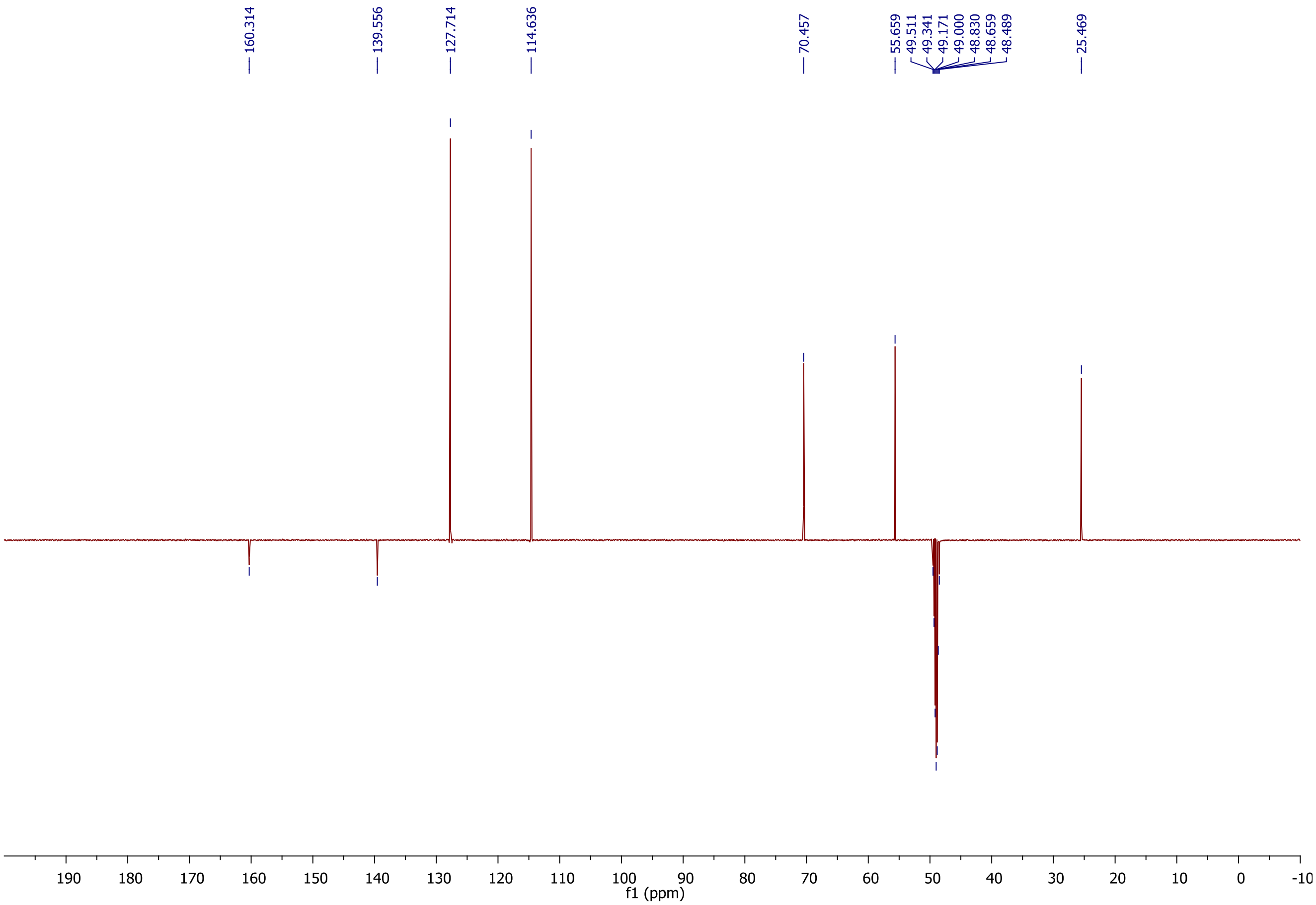

1-(3-Methoxyphenyl)ethan-1-ol

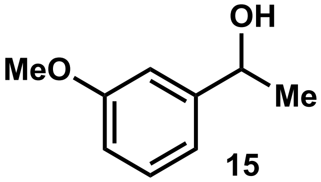

15

The title compound 1-(3-methoxyphenyl)ethan-1-ol **15** was prepared according to general procedure 3 from ((1-(3-methoxyphenyl)vinyl)oxy)trimethylsilane (111 mg, 0.50 mmol, 1 equiv), tris(pentafluorophenyl)borane (25.6 mg, 0.05 mmol, 10 mol %), 2,2,6,6-tetramethylpiperidine (8.4 μL, 0.05 mmol, 10 mol %) and γ-terpinene (104 μL, 0.65 mmol, 1.3 equiv), followed by TBAF (0.50 mmol, 0.5 mL, 1 equiv) and purified by flash silica column chromatography (eluent = 10% EtOAc in hexanes) to give a colorless oil (61 mg, 80% yield). R_f = 0.33 (eluent = 20% EtOAc in hexanes); ν_{max} / cm^{-1} (film) 3350, 2970, 1585, 1487, 1255, 1043, 856, 700; ^{1}H NMR (500 MHz, $CDCl_3$) δ_H: 1.47 (3H, t, *J* 6.5), 2.03 (1H, br s), 3.80 (3H, s), 4.84 (1H, q, *J* 6.5), 6.78-6.81 (1H, m), 6.92-6.93 (2H, m), 7.23-7.25 (1H, m); ^{13}C{^{1}H} NMR

(126 MHz, $CDCl_3$) δ_C: 25.2, 55.3, 70.4, 111.0, 113.0, 117.8, 129.6, 147.7, 159.9; HRMS (ASAP$^+$) calculated for $[C_9H_{11}O_2]^+$ $(M–H)^+$: m/z 151.0759, found 151.0760 (+0.7 ppm).

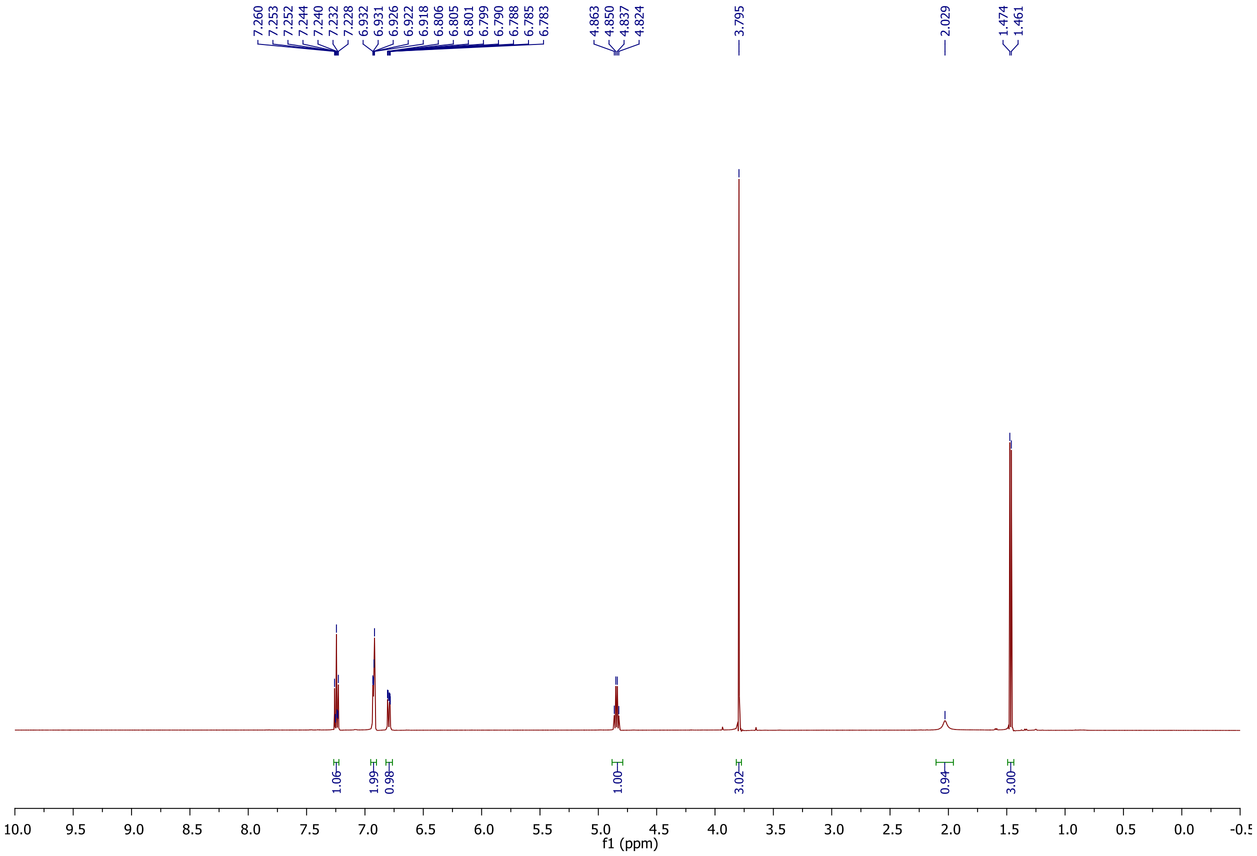

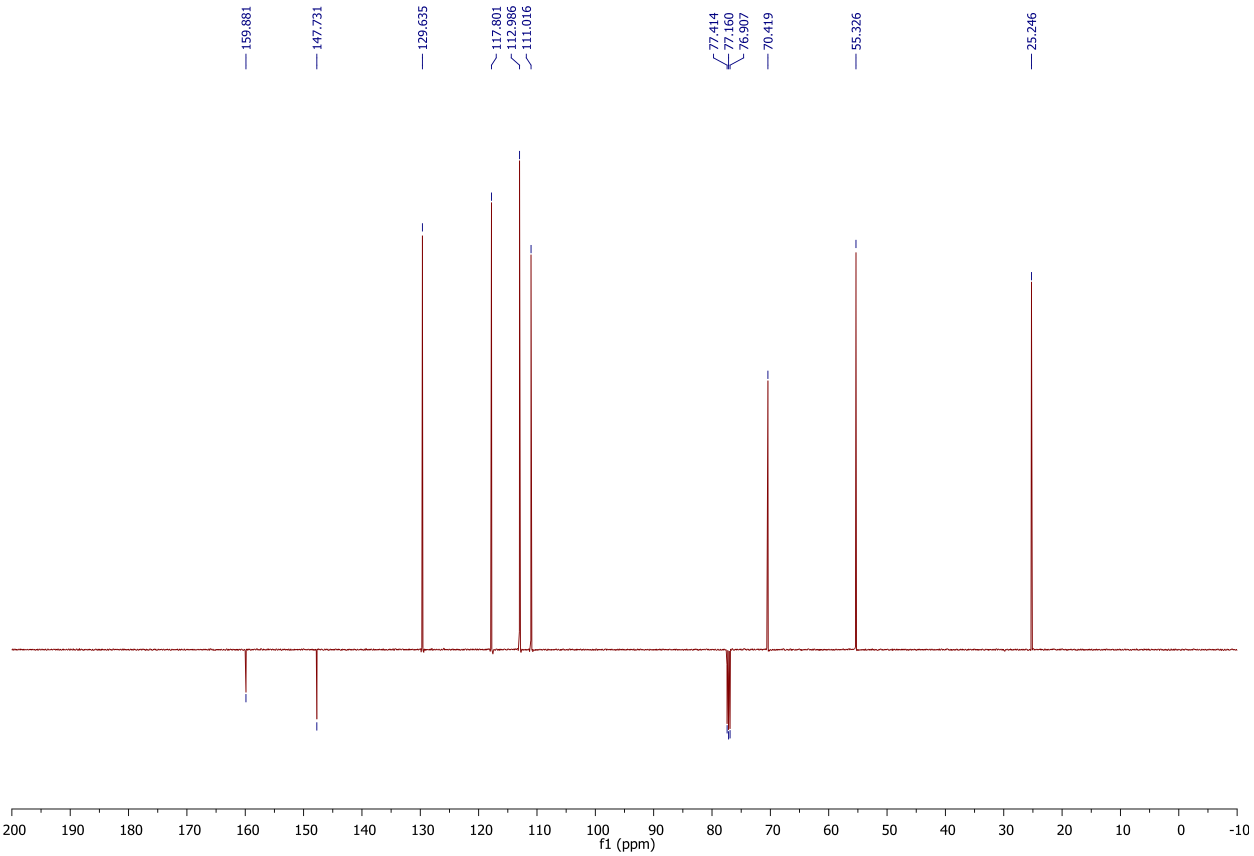

1-(2-Methoxyphenyl)ethan-1-ol

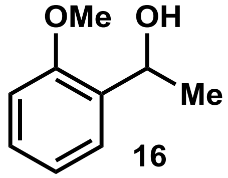

16

The title compound 1-(2-methoxyphenyl)ethan-1-ol **16** was prepared according to general procedure 4 from *tert*-butyl((1-(2-methoxyphenyl)vinyl)oxy)dimethylsilane (132 mg, 0.50 mmol, 1 equiv), tris(pentafluorophenyl)borane (25.6 mg, 0.05 mmol, 10 mol %), 2,2,6,6-tetramethylpiperidine (8.4 μL, 0.05 mmol, 10 mol %) and γ-terpinene (104 μL, 0.65 mmol, 1.3 equiv), followed by TBAF (0.50 mmol, 0.5 mL, 1 equiv) and purified by flash silica column chromatography (eluent = 10% EtOAc in hexanes) to give a colorless oil (63 mg, 83% yield). R_f = 0.29 (eluent = 20% EtOAc in hexanes); ν_{max} / cm^{-1} (film) 3365, 2980, 1600, 1489, 1236, 1028, 800, 752; ^{1}H NMR (500 MHz, $CDCl_3$) δ_H: 1.51 (3H, t, *J* 6.5), 2.68 (1H, d, *J* 4.4), 3.87 (3H, s), 5.08-5.12 (1H, m), 6.89 (1H, dd, *J* 8.2, 0.9), 6.95-6.98 (1H, m), 7.23-7.27 (1H, m), 7.35 (1H, dd, *J* 7.5,1.7); ^{13}C{^{1}H} NMR (126 MHz, $CDCl_3$) δ_c: 23.0, 55.4, 66.6, 110.5, 120.9,

126.2, 128.4, 133.6, 156.7; HRMS (ASAP$^+$) calculated for $[C_9H_{11}O_2]^+$ (M–H)$^+$: m/z 151.0759, found 151.0759 (0.0 ppm).

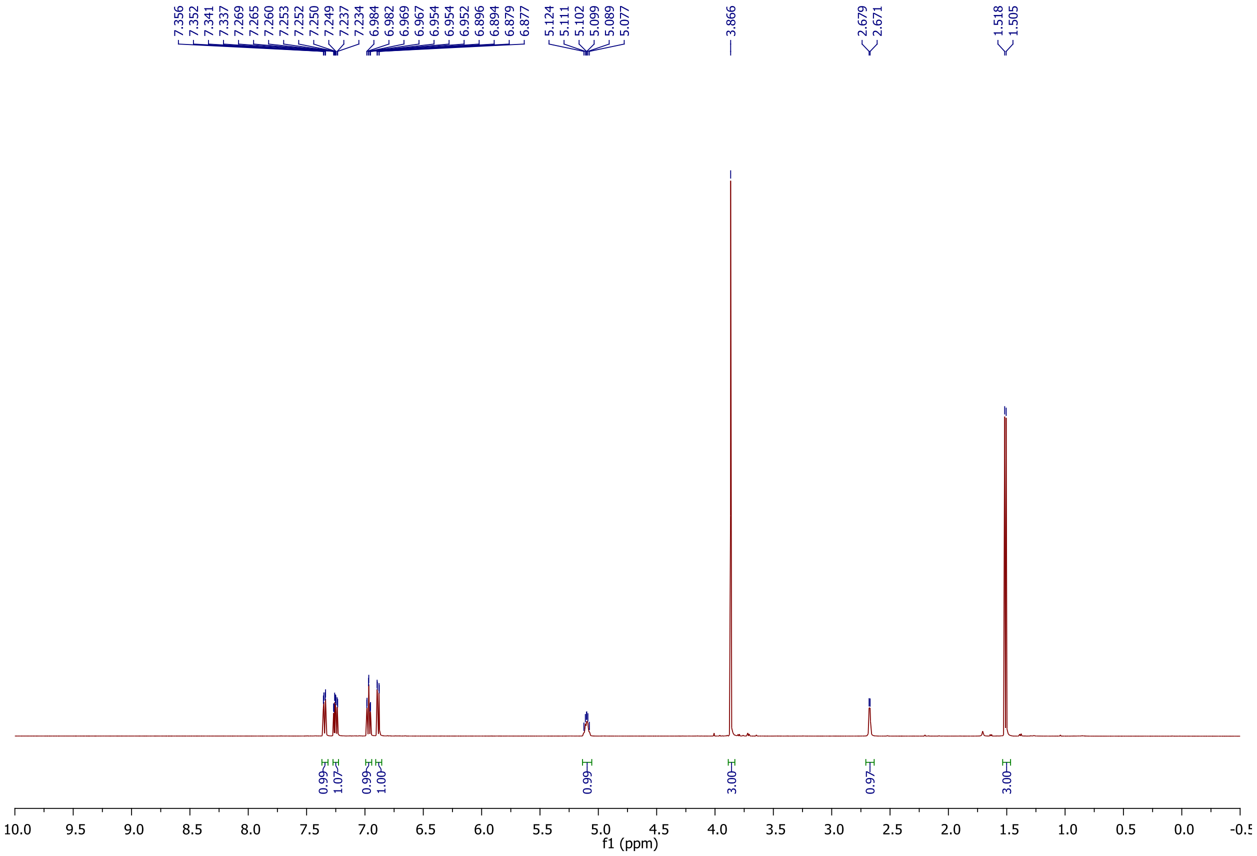

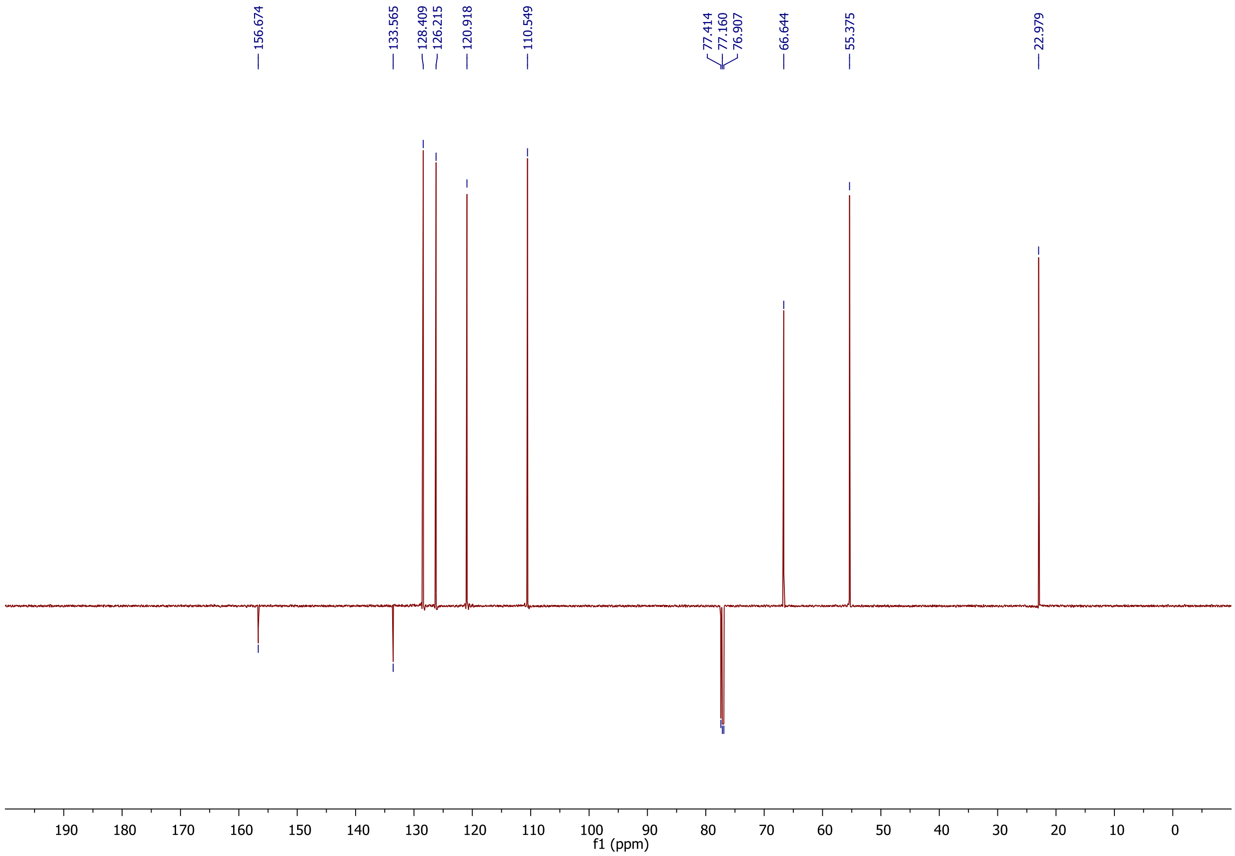

1-(4-(Trifluoromethyl)phenyl)ethan-1-ol

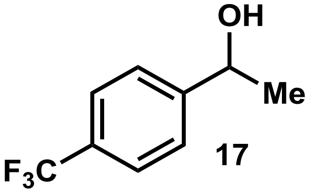

The title compound 1-(4-(trifluoromethyl)phenyl)ethan-1-ol **17** was prepared according to general procedure 4 from *tert*-butyldimethyl((1-(4-(trifluoromethyl)phenyl)vinyl)oxy)silane (151 mg, 0.50 mmol, 1 equiv), tris(pentafluorophenyl)borane (25.6 mg, 0.05 mmol, 10 mol %), 2,2,6,6-tetramethylpiperidine (8.4 µL, 0.05 mmol, 10 mol %) and γ-terpinene (104 µL, 0.65 mmol, 1.3 equiv), followed by TBAF (0.50 mmol, 0.5 mL, 1 equiv). The reaction resulted in 8% conversion to reduced product **17**. ^{1}H NMR (500 MHz, $CDCl_3$) δ_H: 4.92 (1H, q, *J* 6.3).

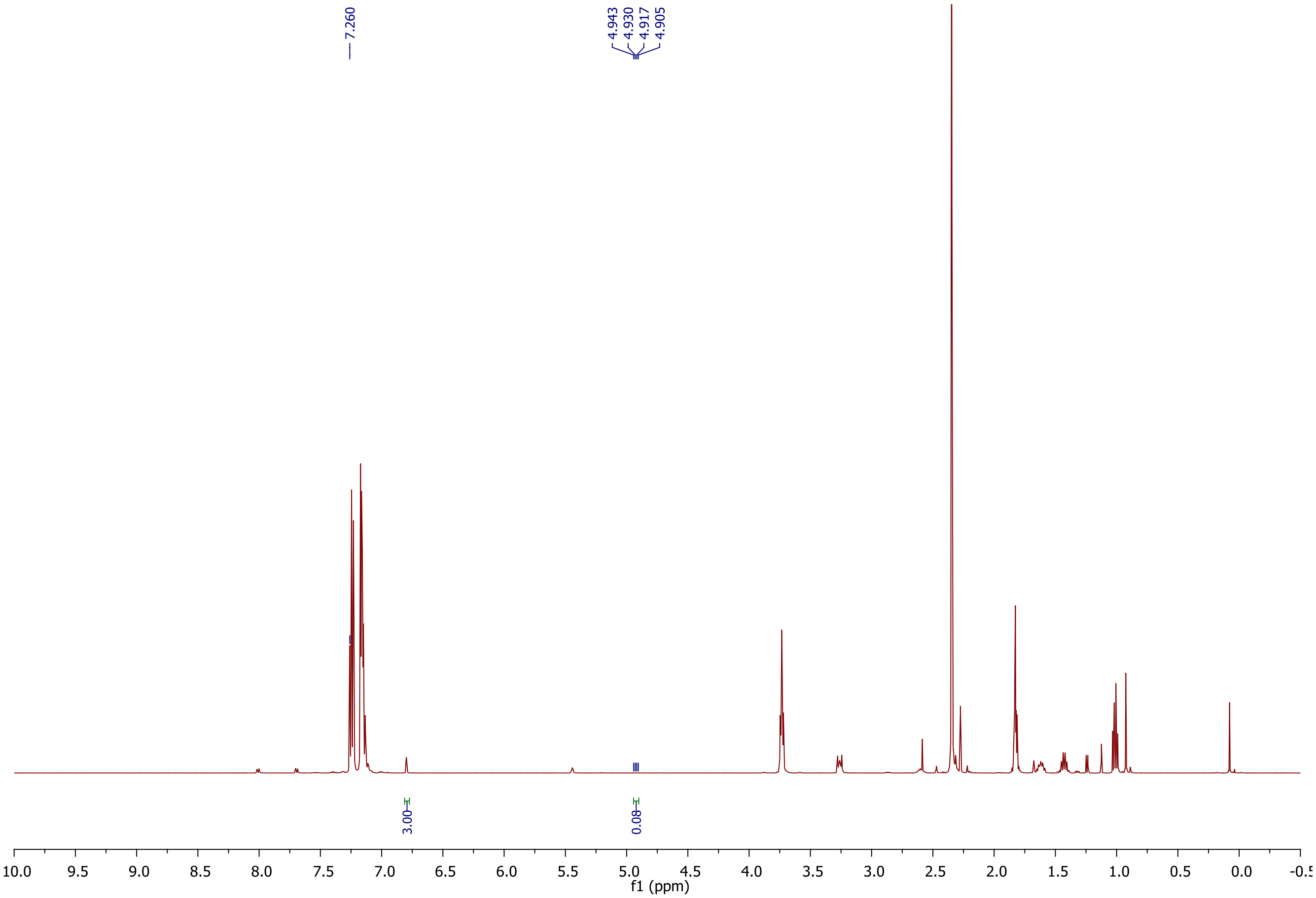

1-(4-Fluorophenyl)ethan-1-ol

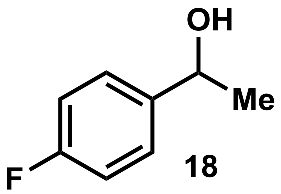

18

The title compound 1-(4-fluorophenyl)ethan-1-ol **18** was prepared according to general procedure 3 from ((1-(4-fluorophenyl)vinyl)oxy)trimethylsilane (105 mg, 0.50 mmol, 1 equiv), tris(pentafluorophenyl)borane (25.6 mg, 0.05 mmol, 10 mol %), 2,2,6,6-tetramethylpiperidine (8.4 μL, 0.05 mmol, 10 mol %) and γ-terpinene (104 μL, 0.65 mmol, 1.3 equiv), followed by TBAF (0.50 mmol, 0.5 mL, 1 equiv) and purified by flash silica column chromatography (eluent = 10→20% EtOAc in hexanes) to give a colorless oil (60 mg, 86% yield). R_f = 0.15 (eluent = 20% EtOAc in hexanes); ν_{max} / cm^{-1} (film) 3332, 2974, 1602, 1508, 1219, 1082, 833, 542; ^{1}H NMR (500 MHz, $CDCl_3$) δ_H: 1.47 (3H, t, *J* 6.5), 1.94 (1H, br s), 4.87 (1H, q, *J* 6.5), 7.00-7.05 (2H, m), 7.31-7.35 (2H, m); $^{13}C\{^{1}H\}$ NMR (126 MHz, $CDCl_3$) δ_c: 25.4, 69.9, 115.4 (d, *J* 21.3), 127.2 (d, *J* 8.0), 141.6 (d, *J* 2.1), 162.2 (d, *J* 245.1); $^{19}F\{^{1}H\}$ NMR (470 MHz, $CDCl_3$)

δ_F: -115.4; HRMS (CI$^+$) calculated for $[C_8H_{13}ONF]^+$ $(M+NH_4)^+$: m/z 158.0976, found 158.0977 (+0.8 ppm).

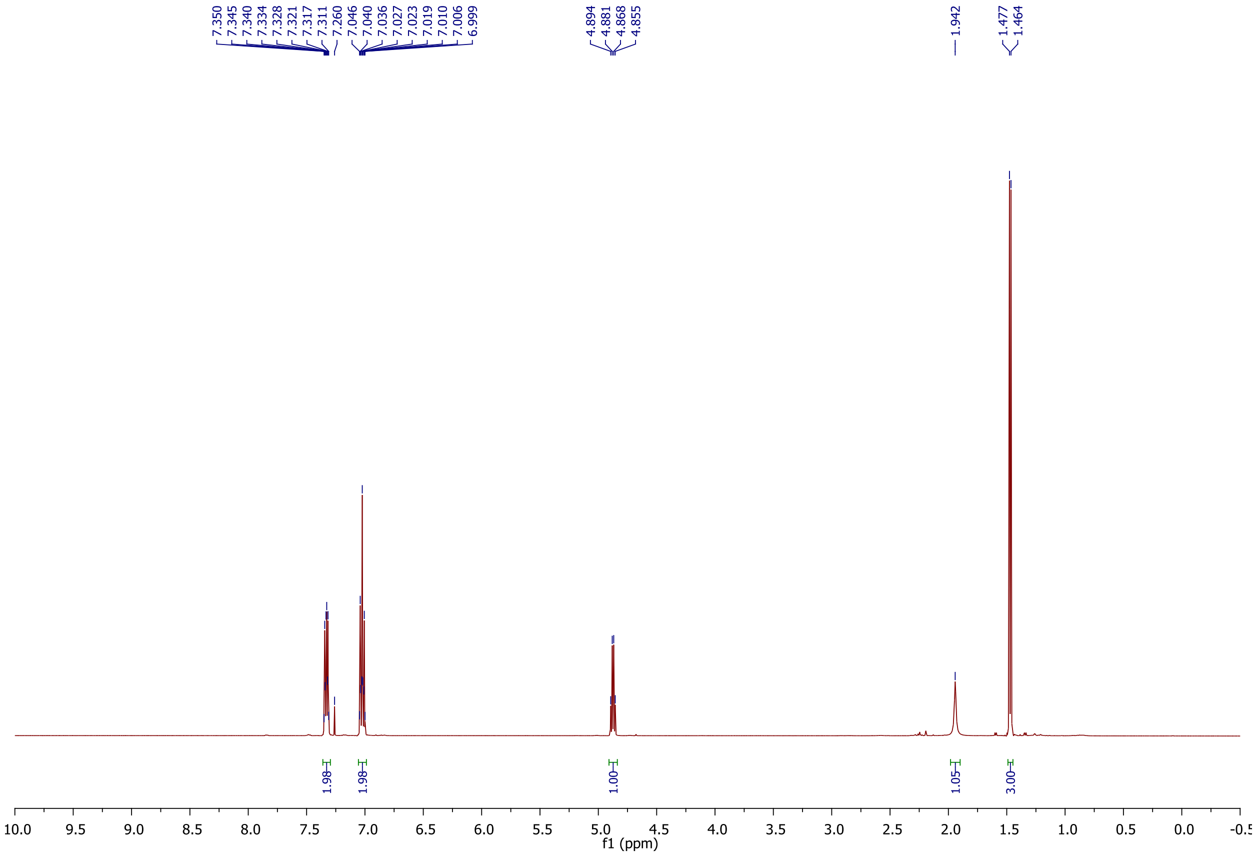

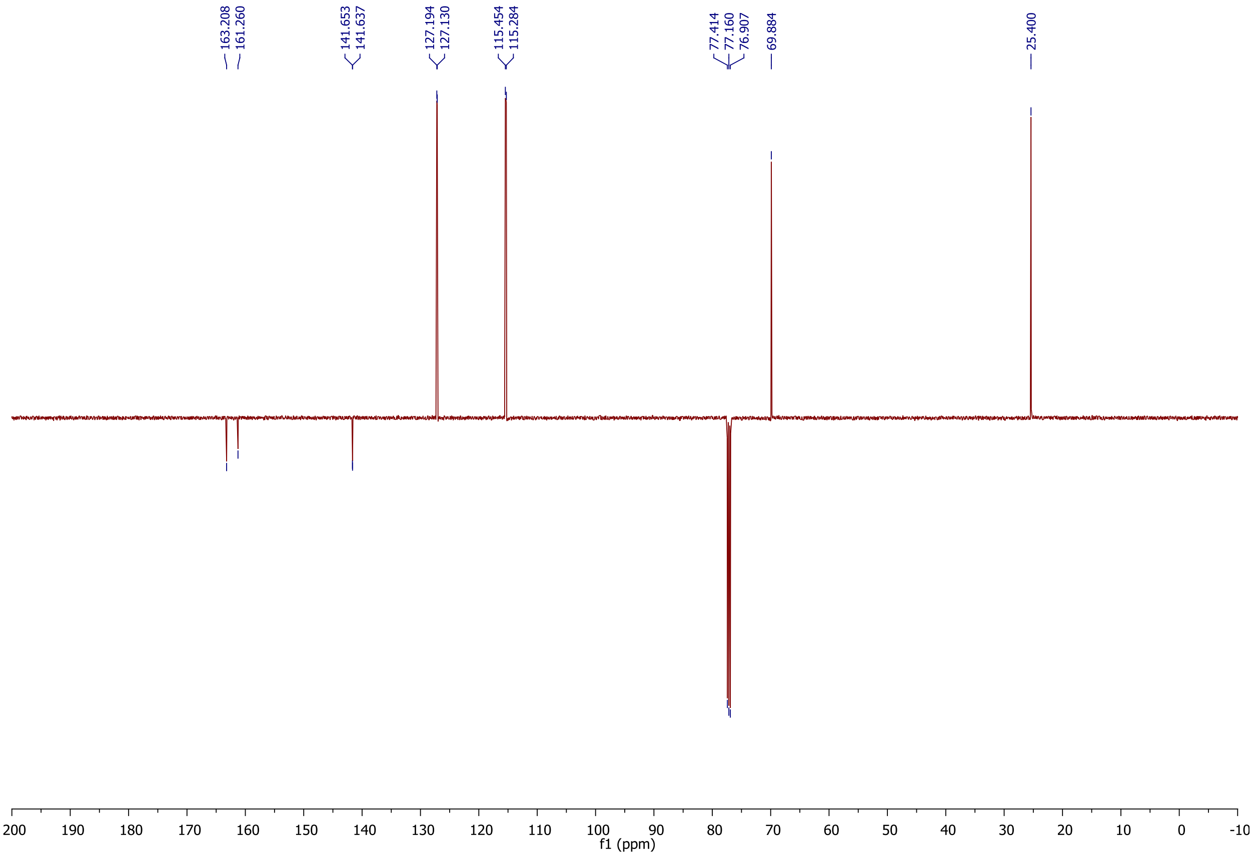
163.208
161.260
141.653
141.637
127.194
127.130
115.454
115.284
77.414
77.160
76.907
69.884
25.400
200
190
180
170
160
150
140
130
120
110
100
90
80
70
60
50
40
30
20
10
0
-10
f1 (ppm)

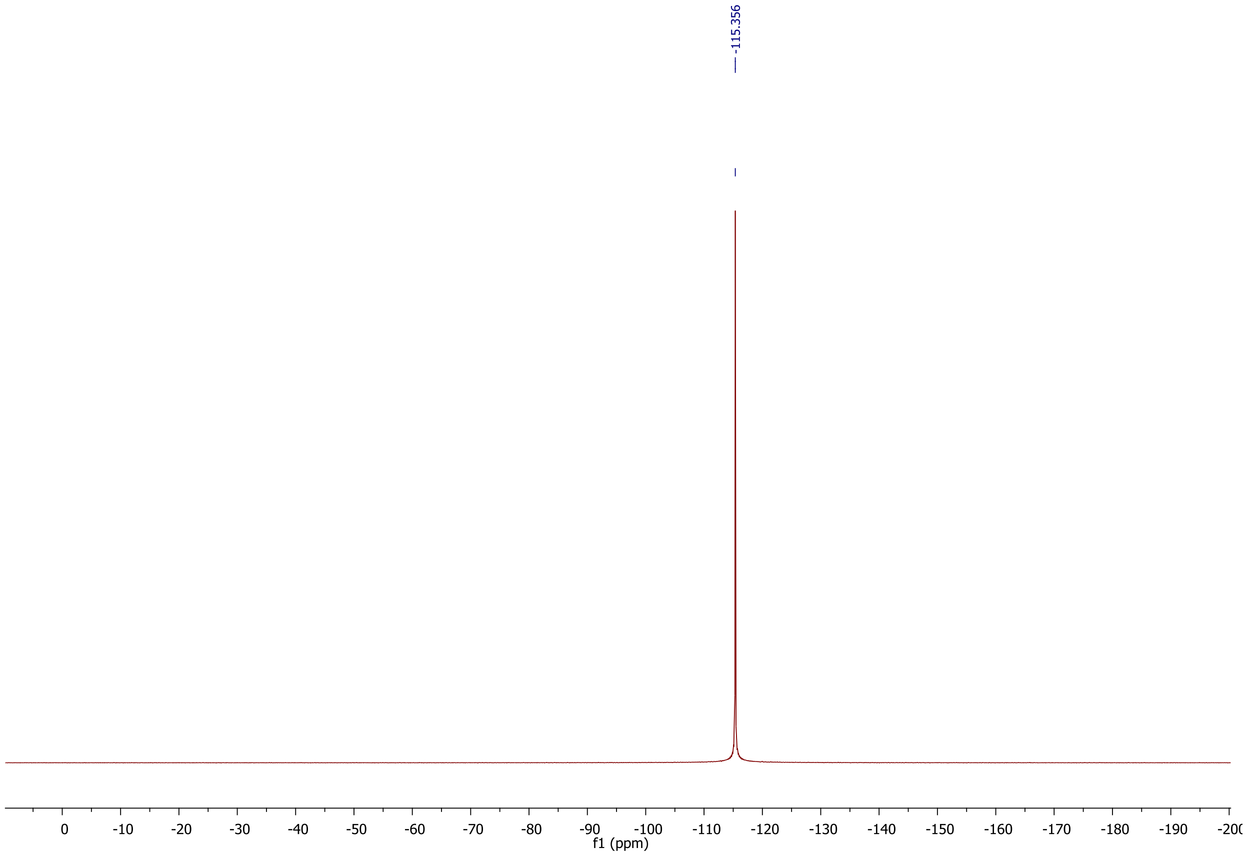
-115.356
0
-10
-20
-30
-40
-50
-60
-70
-80
-90
-100
-110
-120
-130
-140
-150
-160
-170
-180
-190
-200
f1 (ppm)

1-(4-Chlorophenyl)ethan-1-ol

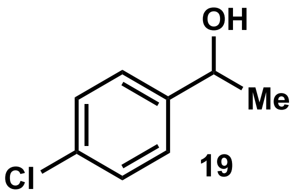

19

The title compound 1-(4-chlorophenyl)ethan-1-ol **19** was prepared according to general procedure 3 from ((1-(4-chlorophenyl)vinyl)oxy)trimethylsilane (113 mg, 0.50 mmol, 1 equiv), tris(pentafluorophenyl)borane (25.6 mg, 0.05 mmol, 10 mol %), 2,2,6,6-tetramethylpiperidine (8.4 µL, 0.05 mmol, 10 mol %) and γ-terpinene (104 µL, 0.65 mmol, 1.3 equiv), followed by TBAF (0.50 mmol, 0.5 mL, 1 equiv) and purified by flash silica column chromatography (eluent = 10% EtOAc in hexanes) to give a colorless oil (64 mg, 82% yield). R_f = 0.28 (eluent = 20% EtOAc in hexanes); ν_{max} / cm^{-1} (film) 3313, 2972, 1490, 1371, 1085, 827, 540; ^{1}H NMR (500 MHz, $CDCl_3$) δ_H: 1.39 (3H, t, *J* 6.5), 2.59 (1H, br s), 4.77 (1H, q, *J* 6.5), 7.21-7.27 (4H, m); $^{13}C\{^1H\}$ NMR (126 MHz, $CDCl_3$) δ_c: 25.3, 69.7, 126.9, 128.6, 133.0, 144.3; HRMS (ASAP$^+$) calculated for $[C_8H_8OCl]^+$ (M–H)$^+$: m/z 155.0264, found 155.0268 (+2.6 ppm).

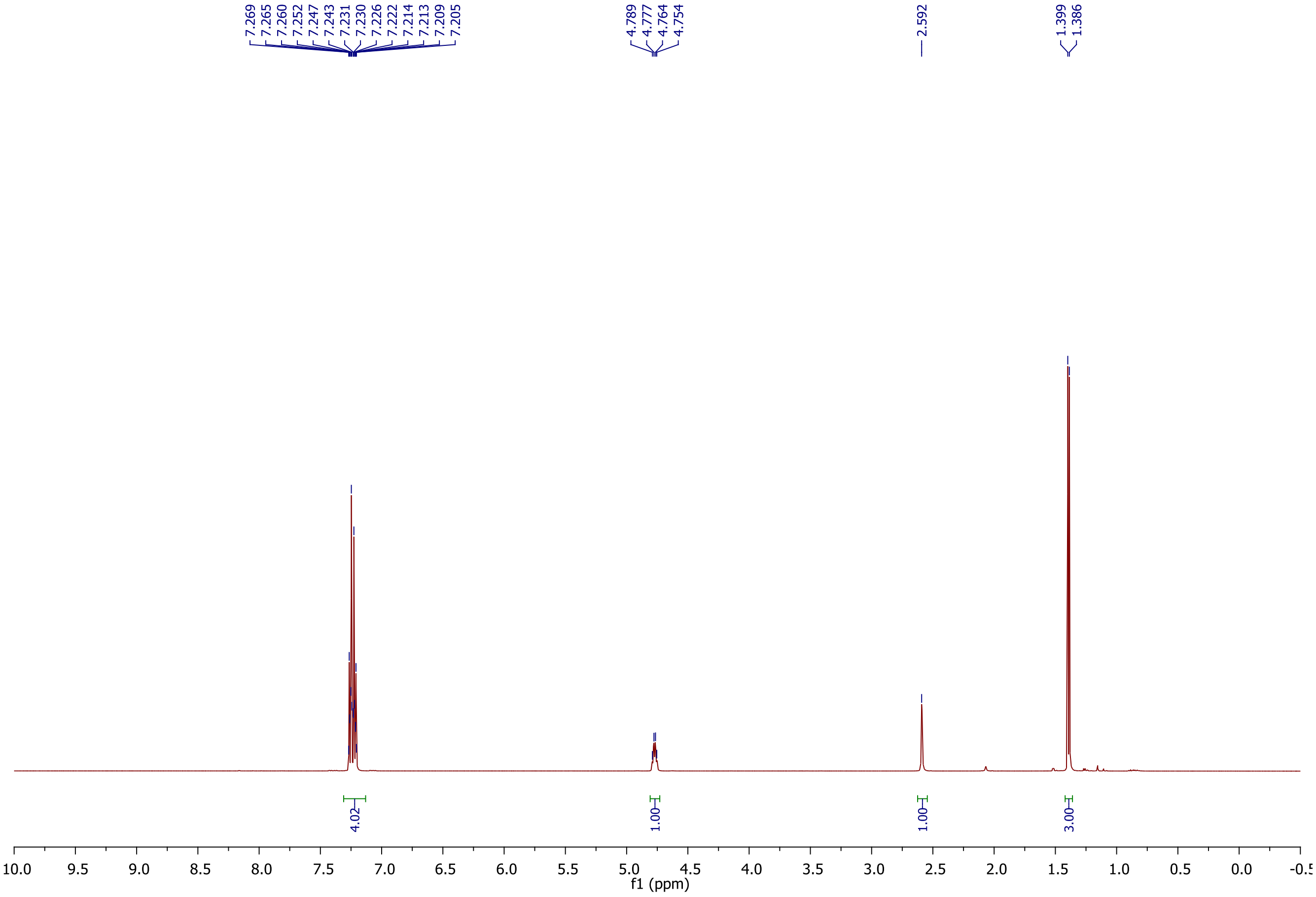

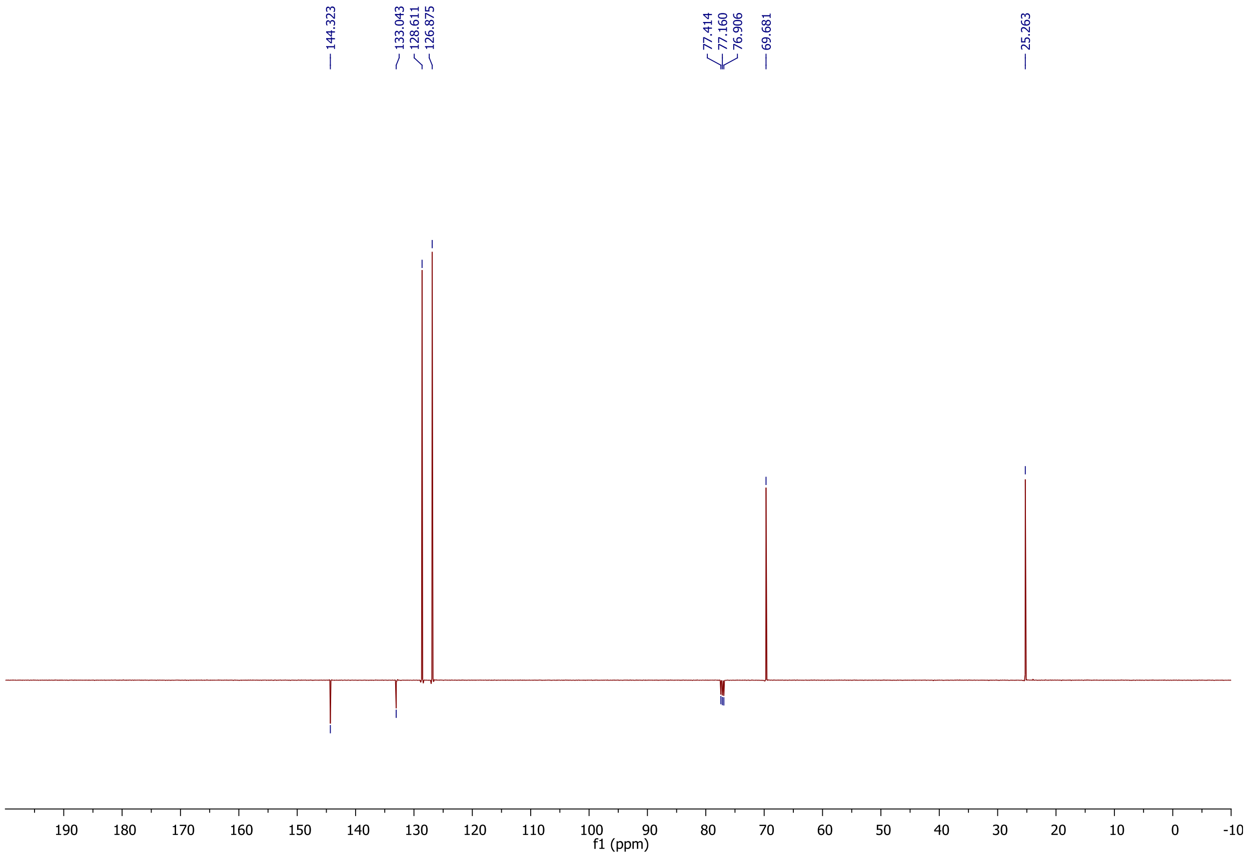

1-(4-Bromophenyl)ethan-1-ol

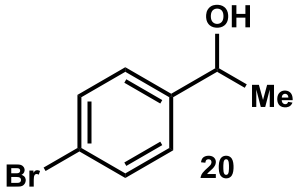

20

The title compound 1-(4-bromophenyl)ethan-1-ol **20** was prepared according to general procedure 3 from ((1-(4-bromophenyl)vinyl)oxy)trimethylsilane (136 mg, 0.50 mmol, 1 equiv), tris(pentafluorophenyl)borane (25.6 mg, 0.05 mmol, 10 mol %), 2,2,6,6-tetramethylpiperidine (8.4 µL, 0.05 mmol, 10 mol %) and γ-terpinene (104 µL, 0.65 mmol, 1.3 equiv), followed by TBAF (0.50 mmol, 0.5 mL, 1 equiv) and purified by flash silica column chromatography (eluent = 10% EtOAc in hexanes) to give a colorless oil (83.2 mg, 83% yield). R_f = 0.34 (eluent = 20% EtOAc in hexanes); ν_{max} / cm^{-1} (film) 3269, 2976, 1573, 1487, 1292, 1070, 819, 528; ^{1}H NMR (500 MHz, $CDCl_3$) δ_H: 1.44 (3H, t, *J* 6.5), 2.24 (1H, br s), 4.82 (1H, q, *J* 6.5), 7.20-7.23 (2H, m), 7.44-7.46 (2H, m); $^{13}C\{^{1}H\}$ NMR (126 MHz, $CDCl_3$) δ_C: 25.3, 69.8, 121.2, 127.3, 131.6, 144.9; HRMS (ASAP$^+$) calculated for $[C_8H_8BrO]^+$ (M–H)$^+$: m/z 198.9759, found 198.9755 (-2.0 ppm).

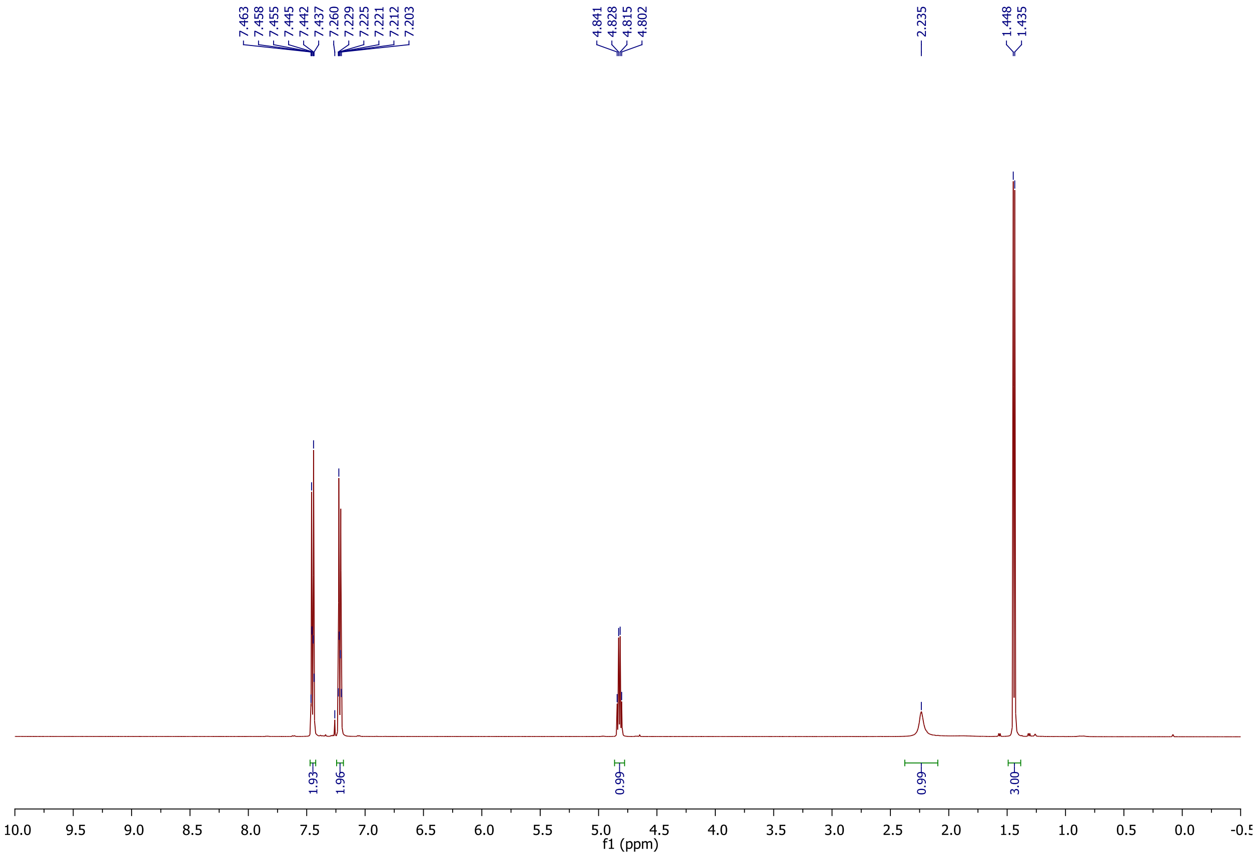
7.463
7.458
7.455
7.445
7.442
7.437
7.260
7.229
7.225
7.221
7.212
7.203
4.841
4.828
4.815
4.802
2.235
1.448
1.435
1.93
1.96
0.99
0.99
3.00
10.0 9.5 9.0 8.5 8.0 7.5 7.0 6.5 6.0 5.5 5.0 4.5 4.0 3.5 3.0 2.5 2.0 1.5 1.0 0.5 0.0
f1 (ppm)

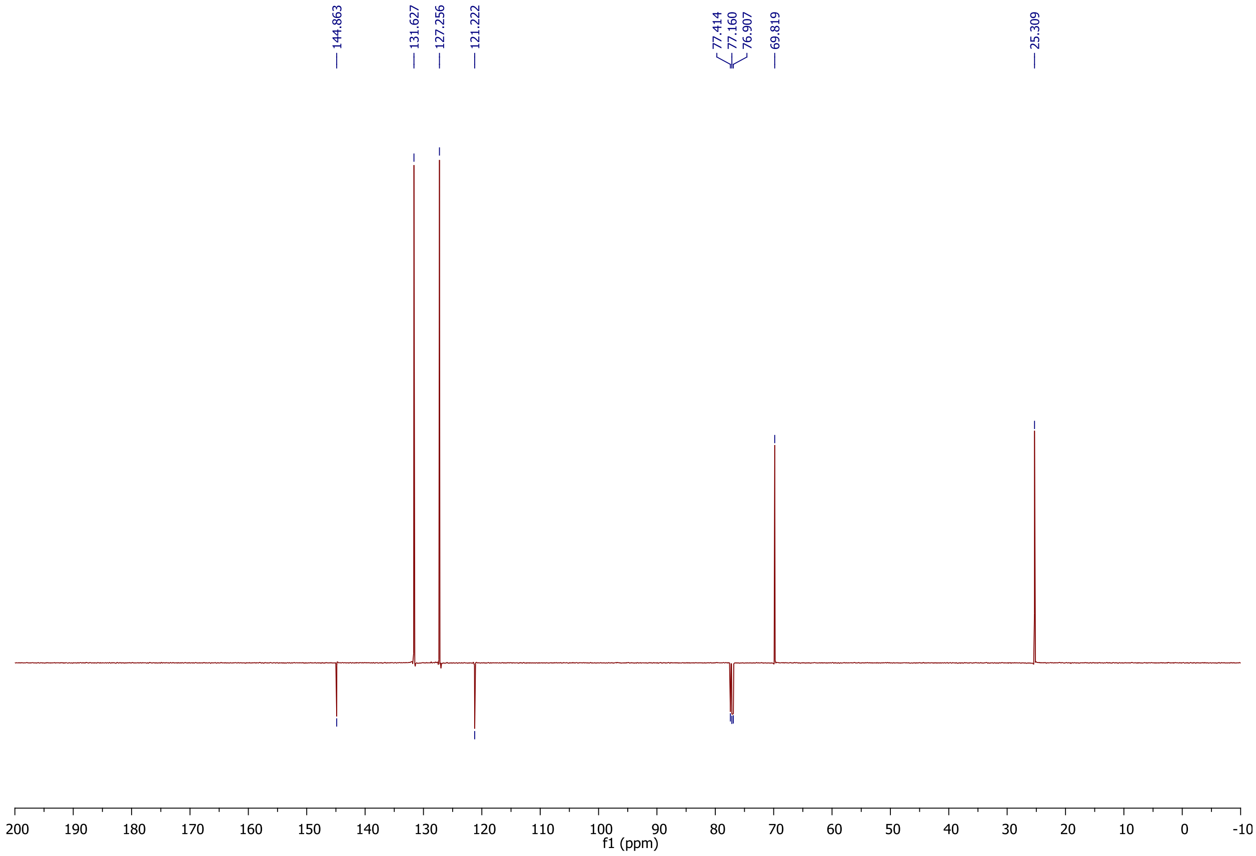
144.863
131.627
127.256
121.222
77.414
77.160
76.907
69.819
25.309
200 190 180 170 160 150 140 130 120 110 100 90 80 70 60 50 40 30 20 10 0 -10
f1 (ppm)

1-(4-Iodophenyl)ethan-1-ol

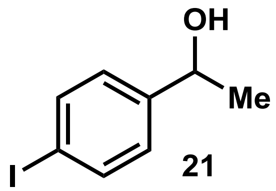

21

The title compound 1-(4-iodophenyl)ethan-1-ol **21** was prepared according to general procedure 3 from ((1-(4-iodophenyl)vinyl)oxy)trimethylsilane (159 mg, 0.50 mmol, 1 equiv), tris(pentafluorophenyl)borane (25.6 mg, 0.05 mmol, 10 mol %), 2,2,6,6-tetramethylpiperidine (8.4 μL, 0.05 mmol, 10 mol %) and γ-terpinene (104 μL, 0.65 mmol, 1.3 equiv), followed by TBAF (0.50 mmol, 0.5 mL, 1 equiv) and purified by flash silica column chromatography (eluent = 10% EtOAc in hexanes) to give a colorless oil (107 mg, 86% yield). R_f = 0.31 (eluent = 20% EtOAc in hexanes); ν_{max} / cm^{-1} (film) 3284, 2972, 1585, 1475, 1074, 813, 526; ^{1}H NMR (500 MHz, $CDCl_3$) δ_H: 1.45 (3H, t, *J* 6.5), 2.09 (1H, br s), 4.82 (1H, q, *J* 6.5), 7.09-7.11 (2H, m), 7.65-7.67 (2H, m); $^{13}C\{^{1}H\}$ NMR (126 MHz, $CDCl_3$) δ_C: 25.3, 69.9, 92.8, 127.5, 137.6, 145.6; HRMS (ASAP$^+$) calculated for $[C_8H_8O^{127}I]^+$ $(M–H)^+$: m/z 246.9620, found 246.9621 (+0.4 ppm).

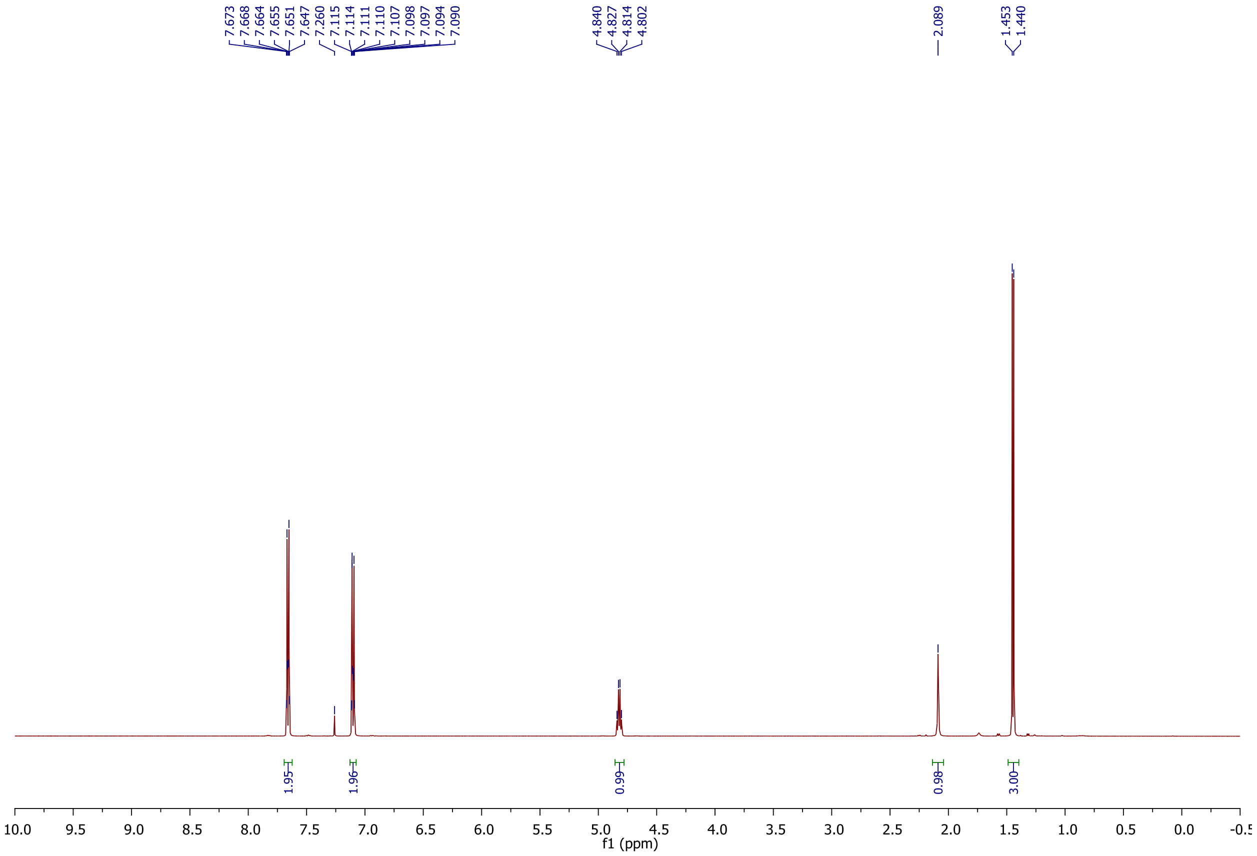

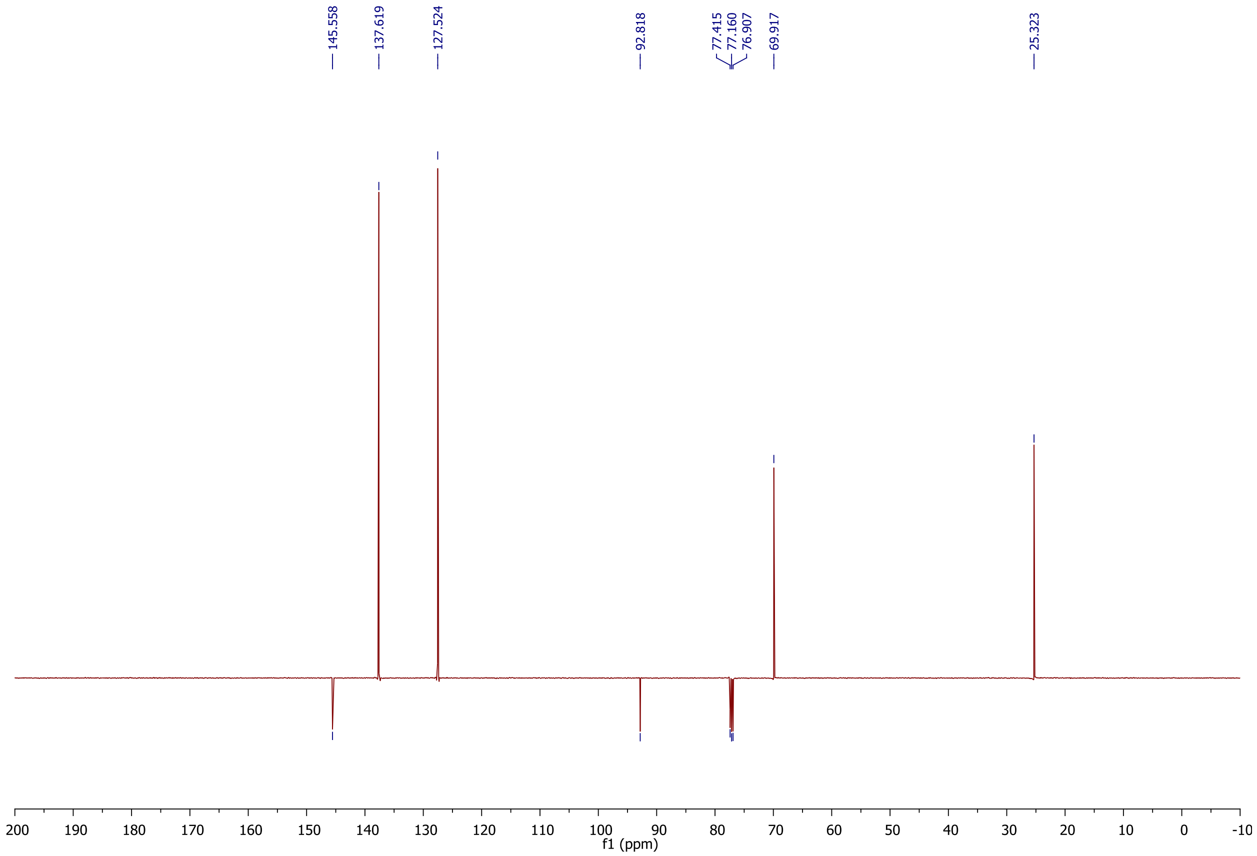

1-(Naphthalen-1-yl)ethan-1-ol

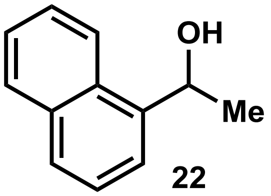

22

The title compound 1-(naphthalen-1-yl)ethan-1-ol **22** was prepared according to general procedure 3 from trimethyl((1-(naphthalen-1-yl)vinyl)oxy)silane (121 mg, 0.50 mmol, 1 equiv), tris(pentafluorophenyl)borane (25.6 mg, 0.05 mmol, 10 mol %), 2,2,6,6-tetramethylpiperidine (8.4 μL, 0.05 mmol, 10 mol %) and γ-terpinene (104 μL, 0.65 mmol, 1.3 equiv), followed by TBAF (0.50 mmol, 0.5 mL, 1 equiv) and purified by flash silica column chromatography (eluent = 10% EtOAc in hexanes) to give a colorless oil (81 mg, 94% yield). R_f = 0.35 (eluent = 20% EtOAc in hexanes); ν_{max} / cm^{-1} (film) 3255, 2970, 1595, 1226, 1012, 800, 775; ^1H NMR (500 MHz, $CDCl_3$) δ_H: 1.67 (3H, t, *J* 6.5), 2.14 (1H, br s), 5.65 (1H, q, *J* 6.5), 7.47-7.55 (3H, m), 7.67 (1H, d, *J* 7.1), 7.79 (1H, d, *J* 8.2), 7.88-7.90 (1H, m), 8.11 (1H, d, *J* 8.1); $^{13}C\{^1H\}$ NMR (126 MHz, $CDCl_3$) δ_C: 24.4, 67.2, 122.1, 123.3, 125.6, 125.6, 126.1, 128.0, 129.0, 130.4,

133.9, 141.5; HRMS (ASAP$^+$) calculated for $[C_{12}H_{11}O]^+$ (M–H)$^+$: m/z 171.0810, found 171.0814 (+2.3 ppm).

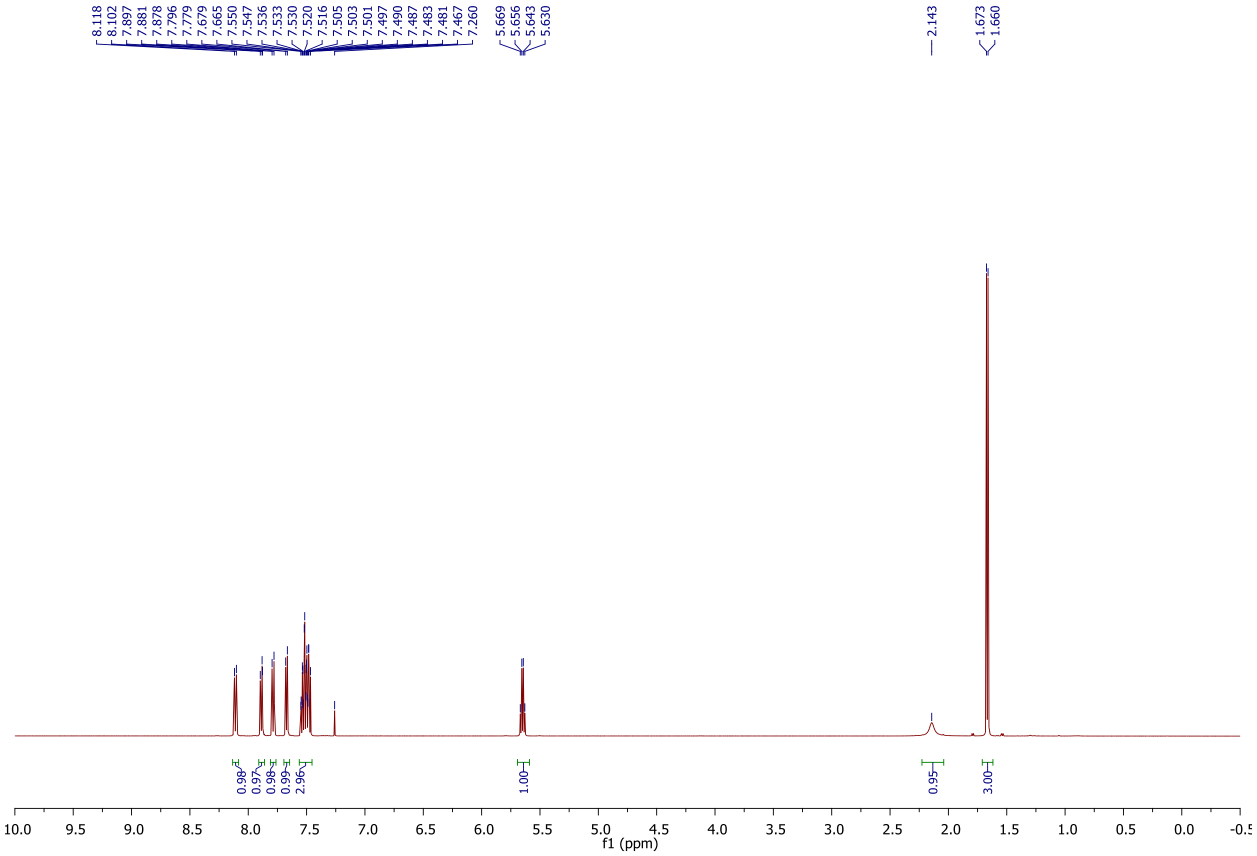

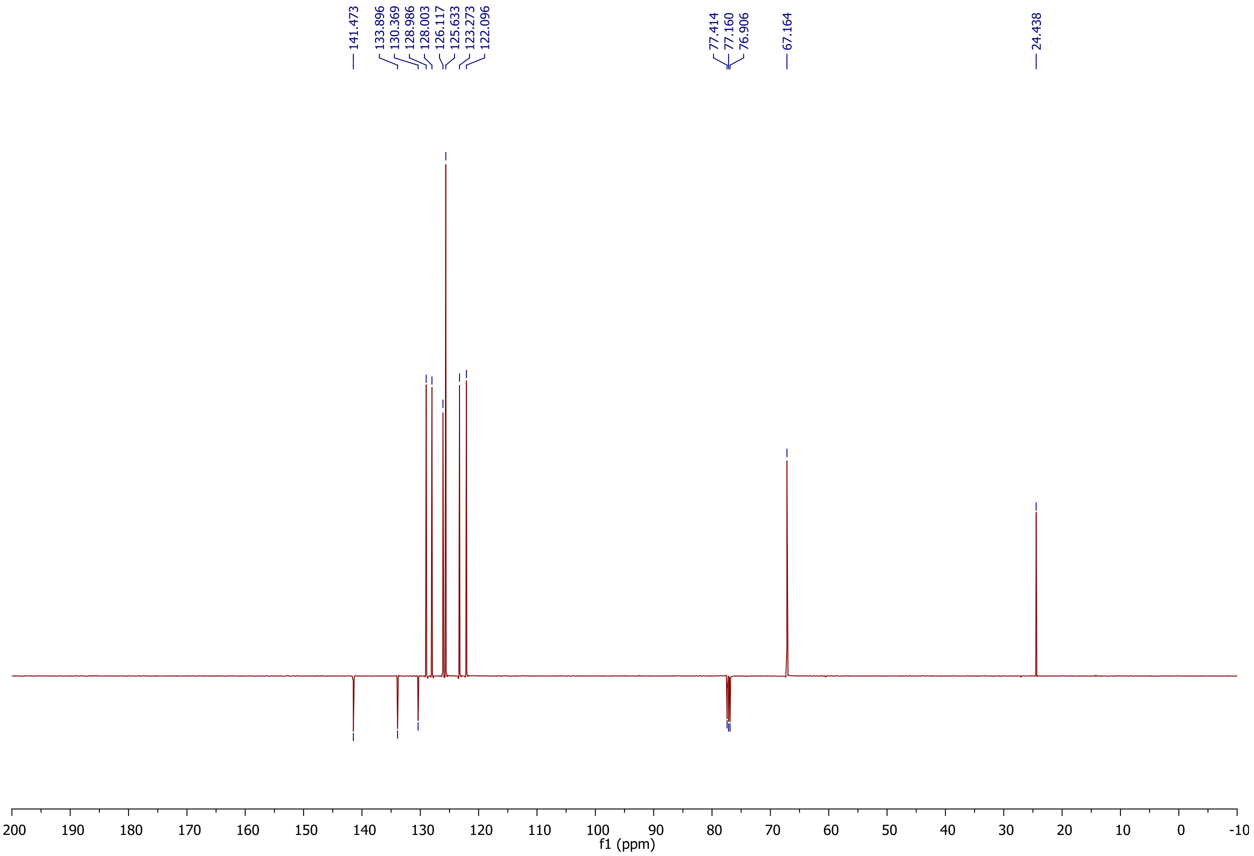

1-(Naphthalen-2-yl)ethan-1-ol

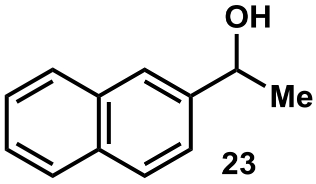

23

The title compound 1-(3-methoxyphenyl)ethan-1-ol **23** was prepared according to general procedure 3 from trimethyl((1-(naphthalen-2-yl)vinyl)oxy)silane (121 mg, 0.50 mmol, 1 equiv), tris(pentafluorophenyl)borane (25.6 mg, 0.05 mmol, 10 mol %), 2,2,6,6-tetramethylpiperidine (8.4 µL, 0.05 mmol, 10 mol %) and γ-terpinene (104 µL, 0.65 mmol, 1.3 equiv), followed by TBAF (0.50 mmol, 0.5 mL, 1 equiv) and purified by flash silica column chromatography (eluent = 10% EtOAc in hexanes) to give a colorless oil (75 mg, 87% yield). R_f = 0.28 (eluent = 20% EtOAc in hexanes); ν_{max} / cm^{-1} (film) 3302, 2970, 1598, 1361, 1274, 1072, 823, 740; ^1H NMR (500 MHz, $CDCl_3$) δ_H: 1.58 (3H, t, *J* 6.5), 2.11 (1H, br s), 5.06 (1H, q, *J* 6.5), 7.46-7.52 (3H, m), 7.80 (1H, s), 7.83-7.85 (3H, m); $^{13}C\{^1H\}$ NMR (126 MHz, $CDCl_3$) δ_c: 25.2, 70.6, 123.9, 123.9, 125.9, 126.3, 127.8, 128.1, 128.4, 133.0, 133.4, 143.3; HRMS (CI^+) calculated for $[C_{12}H_{16}ON]^+$ $(M+NH_4)^+$: m/z 190.1226, found 190.1224 (-1.3 ppm).

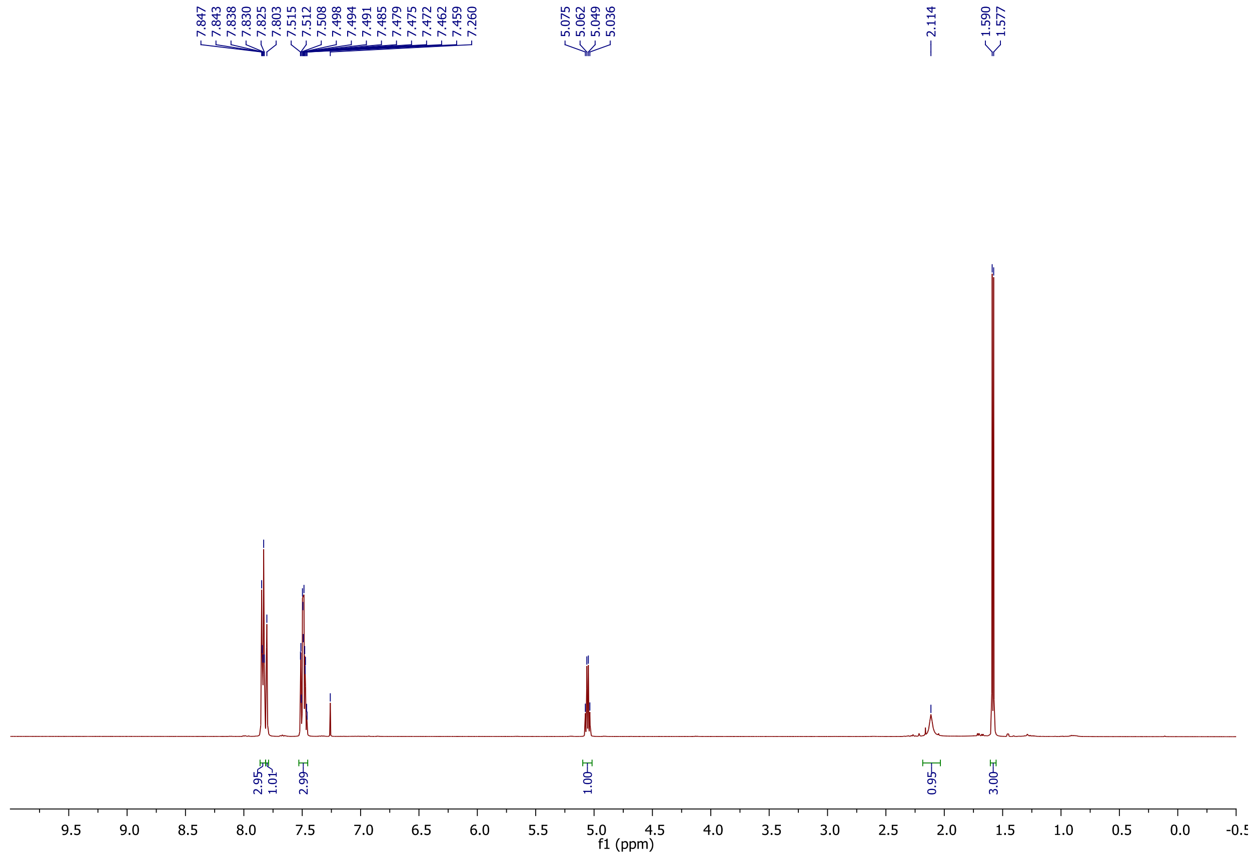
7.847
7.843
7.838
7.830
7.825
7.803
7.515
7.512
7.508
7.498
7.494
7.491
7.485
7.479
7.475
7.472
7.462
7.459
7.260
5.075
5.062
5.049
5.036
2.114
1.590
1.577
2.95
1.01
2.99
1.00
0.95
3.00
f1 (ppm)

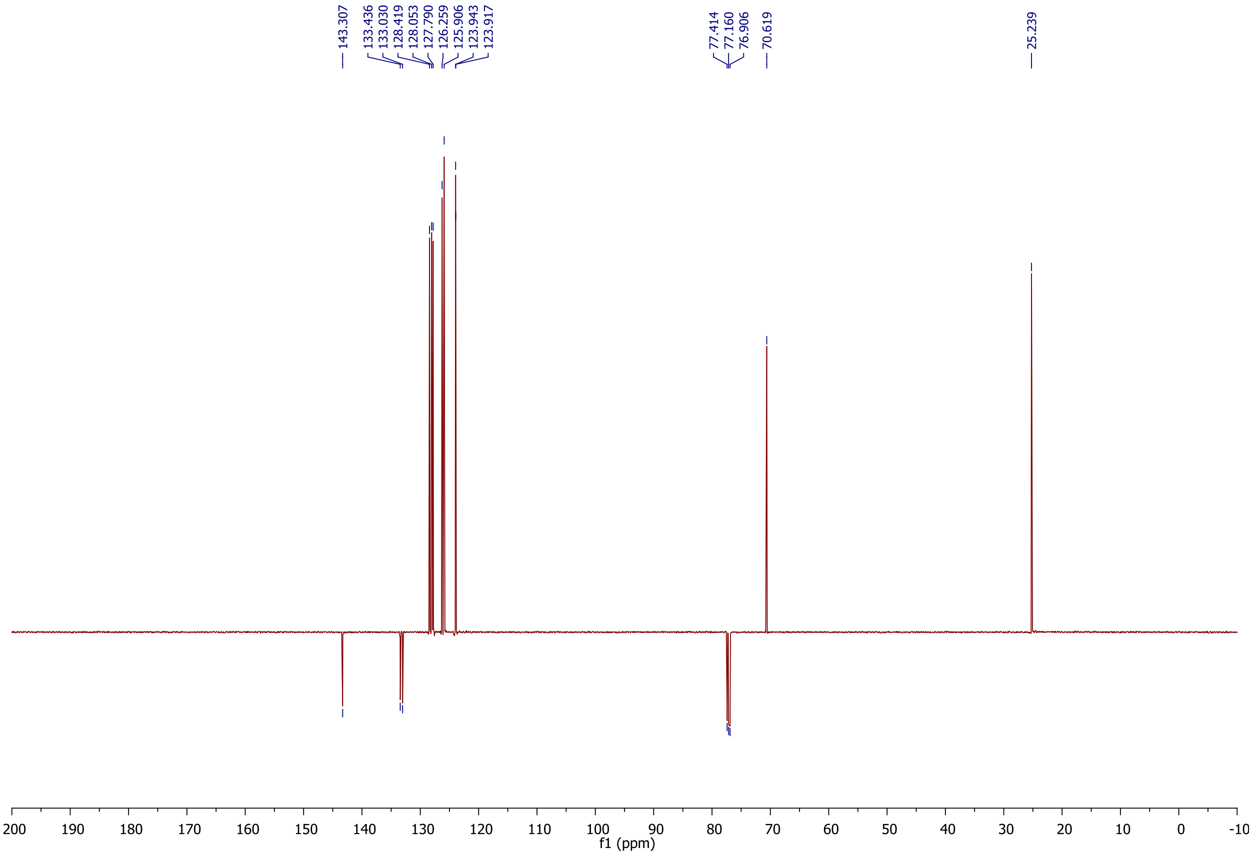
143.307
133.436
133.030
128.419
128.053
127.790
126.259
125.906
123.943
123.917
77.414
77.160
76.906
70.619
25.239
f1 (ppm)

1-(Phenanthren-9-yl)ethan-1-ol

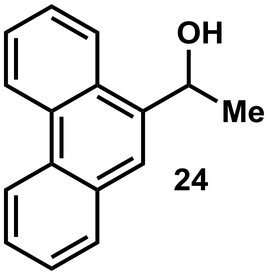

24

The title compound 1-(phenanthren-9-yl)ethan-1-ol **24** was prepared according to general procedure 3 from trimethyl((1-(phenanthren-9-yl)vinyl)oxy)silane (146 mg, 0.50 mmol, 1 equiv), tris(pentafluorophenyl)borane (25.6 mg, 0.05 mmol, 10 mol %), 2,2,6,6-tetramethylpiperidine (8.4 μL, 0.05 mmol, 10 mol %) and γ-terpinene (104 μL, 0.65 mmol, 1.3 equiv), followed by TBAF (0.50 mmol, 0.5 mL, 1 equiv) and purified by flash silica column chromatography (eluent = 10% EtOAc in hexanes) to give an off-white solid (101 mg, 91% yield). R_f = 0.31 (eluent = 20% EtOAc in hexanes); ν_{max} / cm^{-1} (film) 3257, 2912, 1606, 1492, 1249, 1072, 881, 723; ^1H NMR (500 MHz, $CDCl_3$) δ_H: 1.73 (3H, t, *J* 4.8), 2.06 (1H, br s), 5.66 (1H, br s), 7.60-7.65 (4H, m), 7.88-7.92 (2H, m), 8.14 (1H, d, *J* 6.8), 8.66 (1H, d, *J* 6.8), 8.75 (1H, d, *J* 6.8); $^{13}C\{^1H\}$ NMR (126 MHz, $CDCl_3$) δ_c: 24.3, 67.3, 122.6, 122.8, 123.5, 124.0, 126.4, 126.7, 126.7, 126.9, 128.9, 129.7, 130.1, 130.9, 131.6, 139.6; HRMS (ASAP$^+$) calculated for $[C_{16}H_{14}O]^+$ $(M)^+$: m/z 222.1045, found 222.1044 (-0.5 ppm).

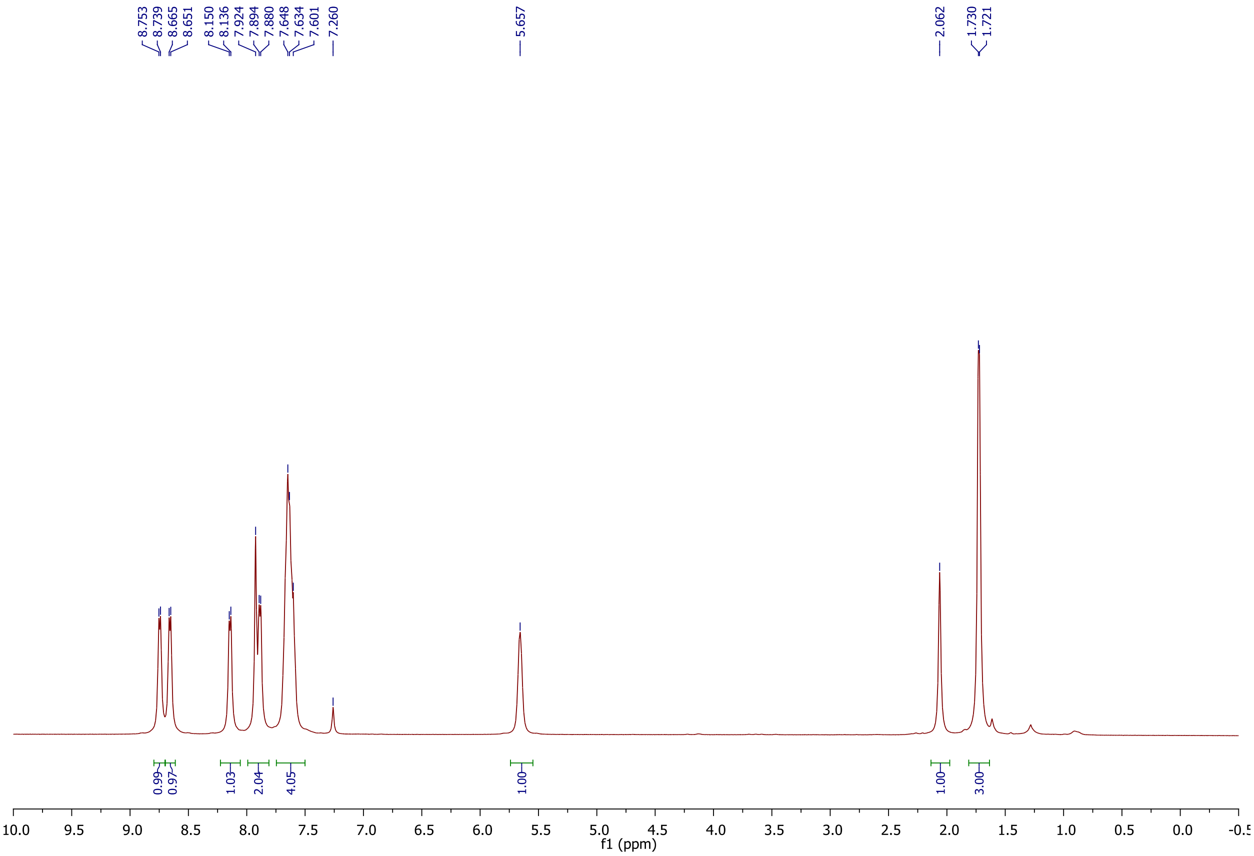
8.753
8.739
8.665
8.651
8.150
8.136
7.924
7.894
7.880
7.648
7.634
7.601
7.260
5.657
2.062
1.730
1.721
0.99
0.97
1.03
2.04
4.05
1.00
1.00
3.00
10.0 9.5 9.0 8.5 8.0 7.5 7.0 6.5 6.0 5.5 5.0 4.5 4.0 3.5 3.0 2.5 2.0 1.5 1.0 0.5 0.0 -0.5
f1 (ppm)

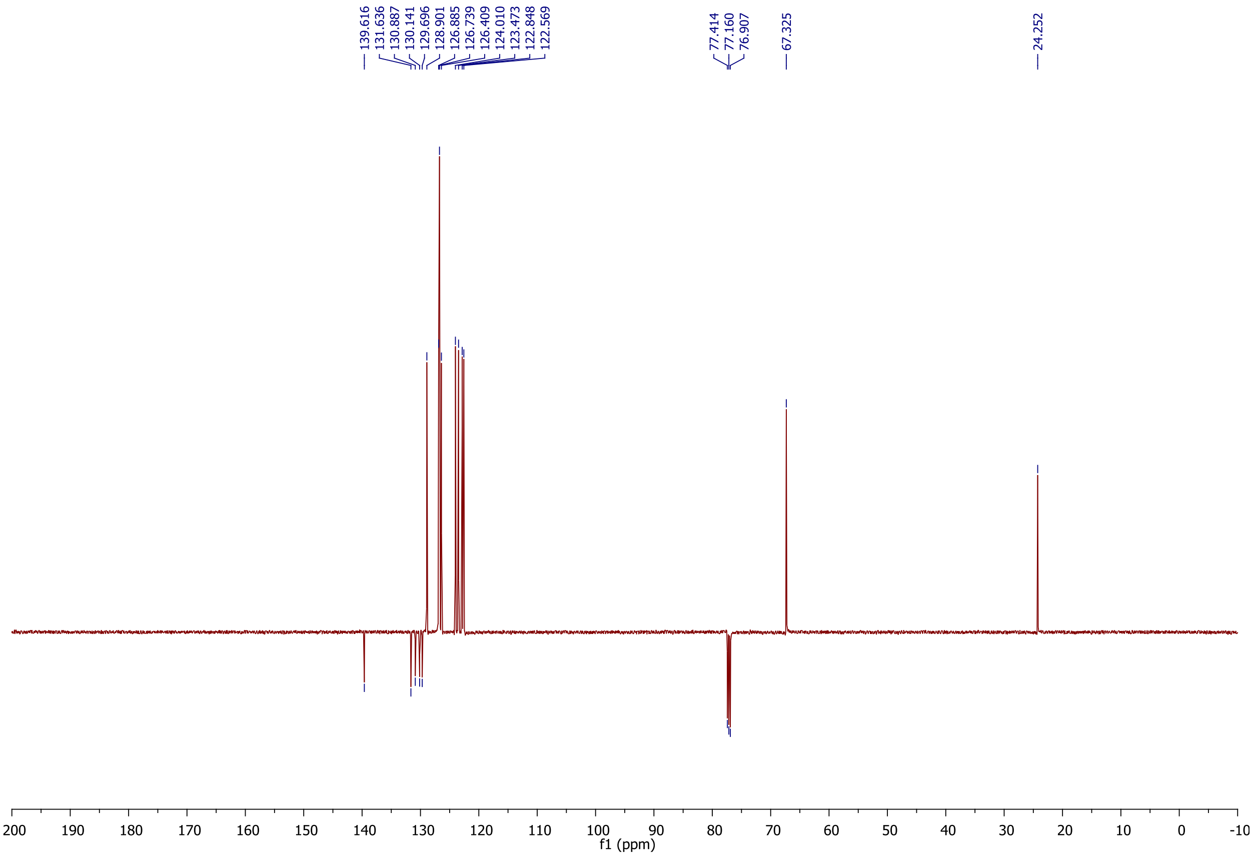
139.616
131.636
130.887
130.141
129.696
128.901
126.885
126.739
126.409
124.010
123.473
122.848
122.569
77.414
77.160
76.907
67.325
24.252
200 190 180 170 160 150 140 130 120 110 100 90 80 70 60 50 40 30 20 10 0 -10
f1 (ppm)

1-(Thiophen-2-yl)ethan-1-ol

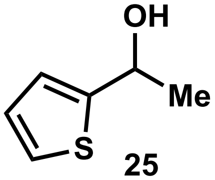

25

The title compound 1-(thiophen-2-yl)ethan-1-ol **25** was prepared according to general procedure 4 from *tert*-butyldimethyl((1-(thiophen-2-yl)vinyl)oxy)silane (120 mg, 0.50 mmol, 1 equiv), tris(pentafluorophenyl)borane (25.6 mg, 0.05 mmol, 10 mol %), 2,2,6,6-tetramethylpiperidine (8.4 µL, 0.05 mmol, 10 mol %) and γ-terpinene (104 µL, 0.65 mmol, 1.3 equiv), followed by TBAF (0.50 mmol, 0.5 mL, 1 equiv) and purified by flash silica column chromatography (eluent = 10% EtOAc in hexanes) to give a light yellow oil (53 mg, 83% yield). R_f = 0.37 (eluent = 20% EtOAc in hexanes); ν_{max} / cm^{-1} (film) 3334, 2980, 1373, 1234, 1072, 848, 698; ^{1}H NMR (400 MHz, CD_3OD) δ_H: 1.53 (3H, t, *J* 6.4), 4.89 (1H, br s), 5.06 (1H, q, *J* 6.4), 6.93-6.95 (1H, m), 6.96-6.97 (1H, m), 7.27 (1H, dd, *J* 5.0, 1.3); $^{13}C\{^{1}H\}$ NMR (101 MHz, CD_3OD) δ_C: 25.7, 66.7, 124.0, 124.9, 127.4, 151.6; HRMS (CI^+) calculated for $[C_6H_{12}ONS]^+$ $(M+NH_4)^+$: m/z 146.0634, found 146.0634 (0.0 ppm).

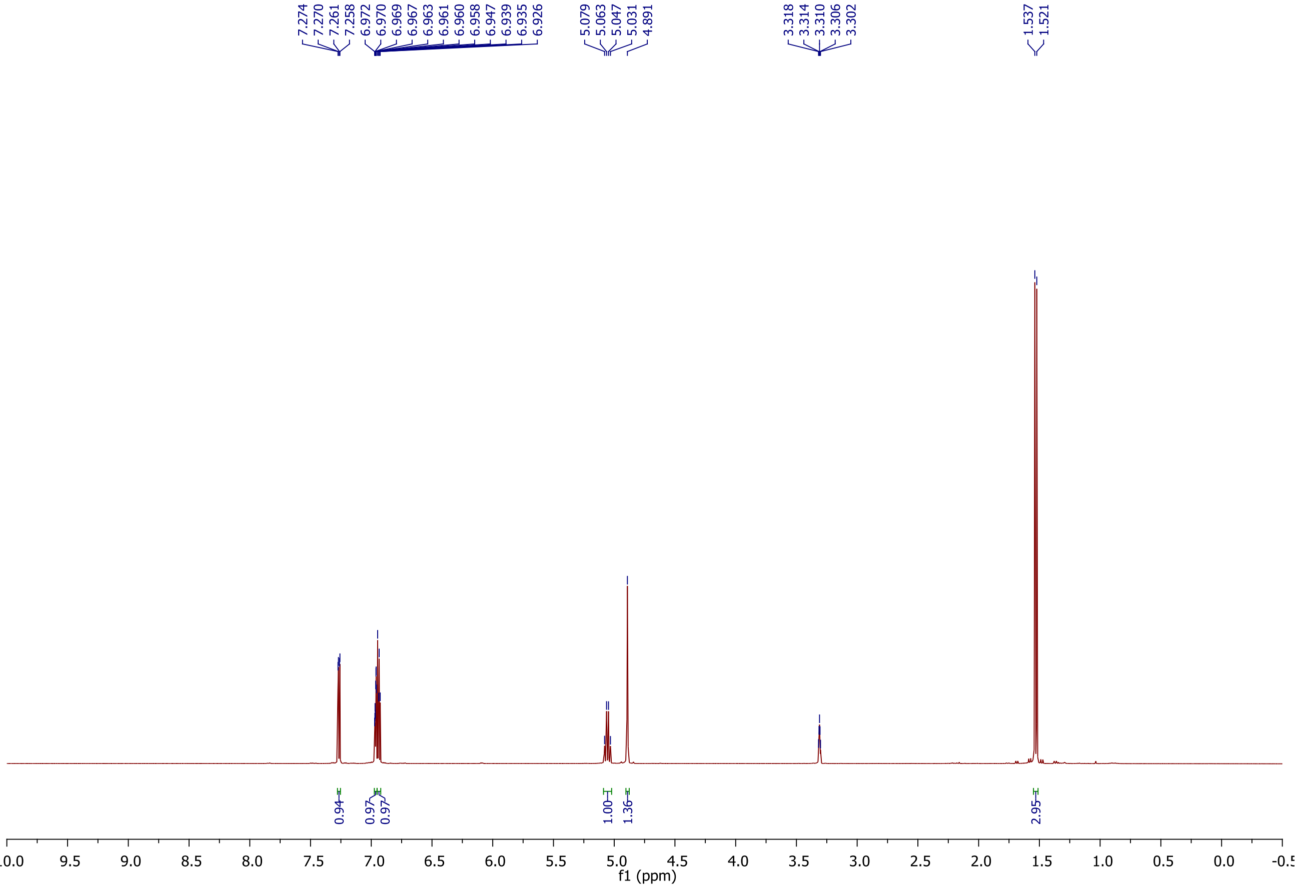

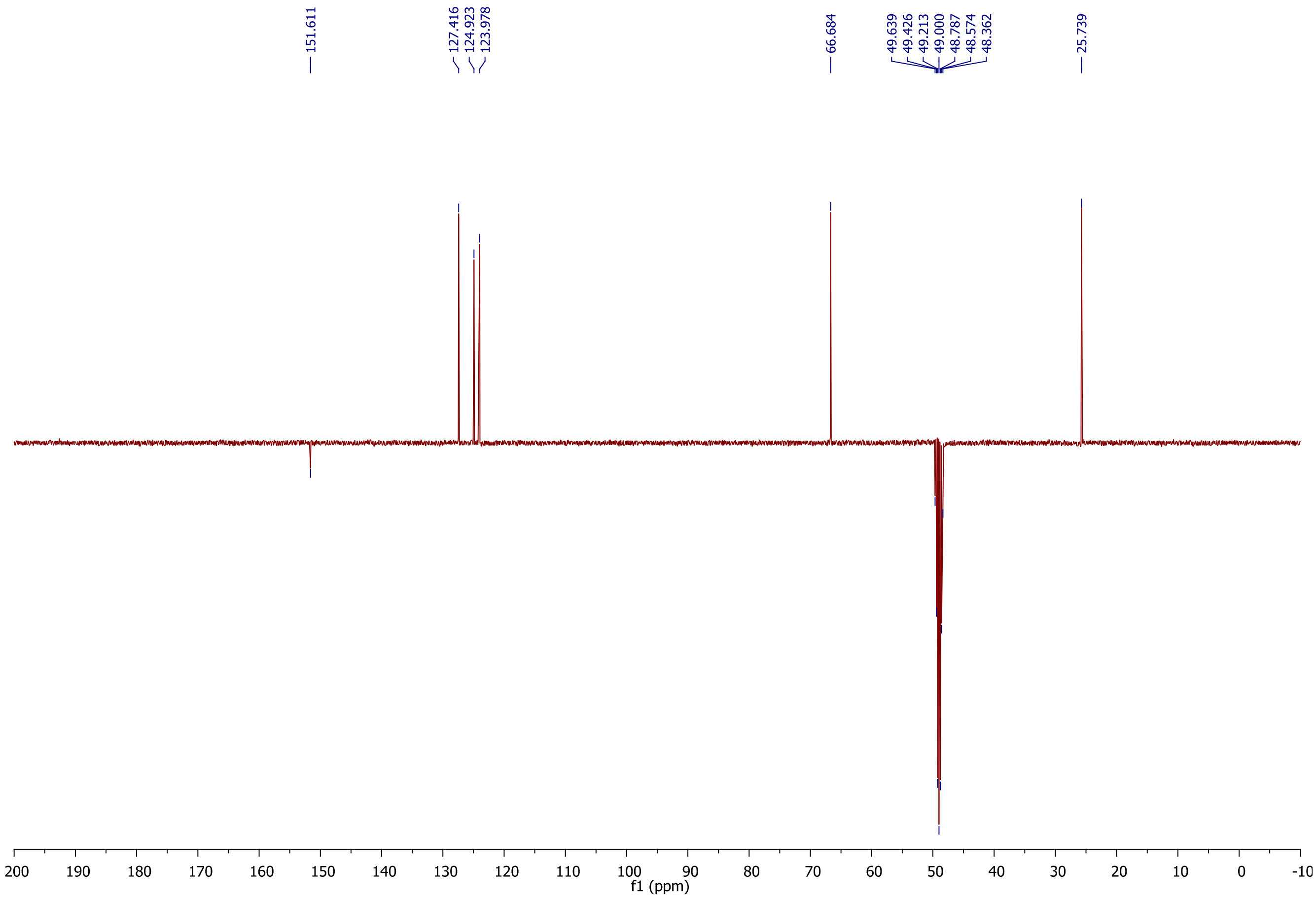

1-(Benzo[*b*]thiophen-2-yl)ethan-1-ol

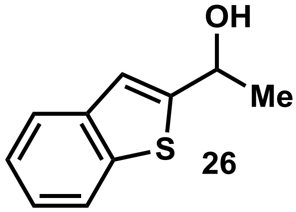

26

The title compound 1-(benzo[*b*]thiophen-2-yl)ethan-1-ol **26** was prepared according to general procedure 4 from ((1-(benzo[*b*]thiophen-2-yl)vinyl)oxy)(*tert*-butyl)dimethylsilane (145 mg, 0.50 mmol, 1 equiv), tris(pentafluorophenyl)borane (25.6 mg, 0.05 mmol, 10 mol %), 2,2,6,6-tetramethylpiperidine (8.4 µL, 0.05 mmol, 10 mol %) and γ-terpinene (104 µL, 0.65 mmol, 1.3 equiv), followed by TBAF (0.50 mmol, 0.5 mL, 1 equiv) and purified by flash silica column chromatography (eluent = 10% EtOAc in hexanes) to give a yellow solid (81 mg, 91% yield). R_f = 0.31 (eluent = 20% EtOAc in hexanes); ν_{max} / cm^{-1} (film) 3257, 2980, 1456, 1141, 829, 725; ^{1}H NMR (500 MHz, $CDCl_3$) δ_H: 1.66 (3H, t, *J* 6.4), 2.24 (1H, br s), 5.19 (1H, q, *J* 6.4), 7.18 (1H, t, *J* 0.7), 7.29-7.36 (2H, m), 7.71-7.72 (1H, m), 7.80-7.83 (1H, m); $^{13}C\{^{1}H\}$ NMR

(126 MHz, $CDCl_3$) δ_C: 25.2, 67.0, 119.6, 122.6, 123.6, 124.3, 124.4, 139.4, 139.7, 150.6; HRMS (ASAP$^+$) calculated for $[C_{10}H_9OS]^+$ $(M–H)^+$: m/z 177.0374, found 177.0376 (+1.1 ppm).

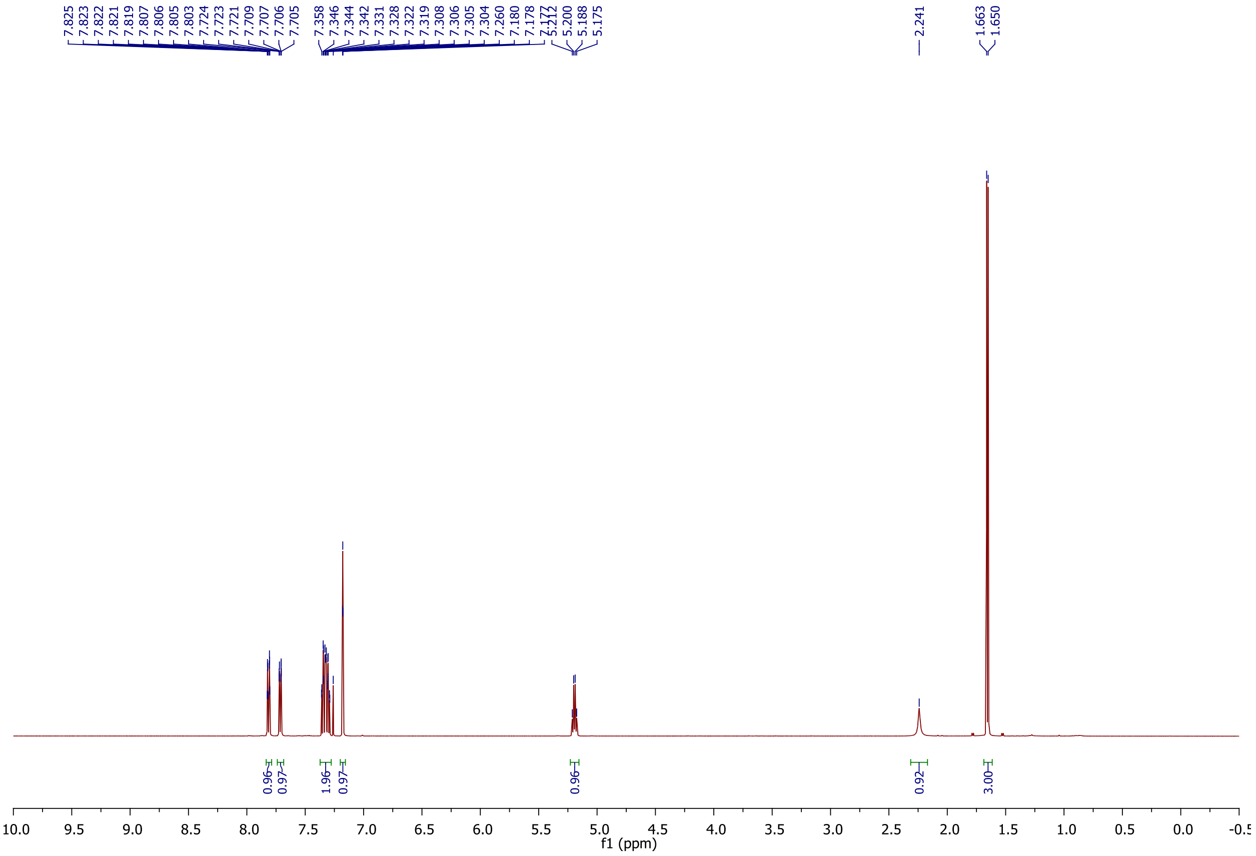

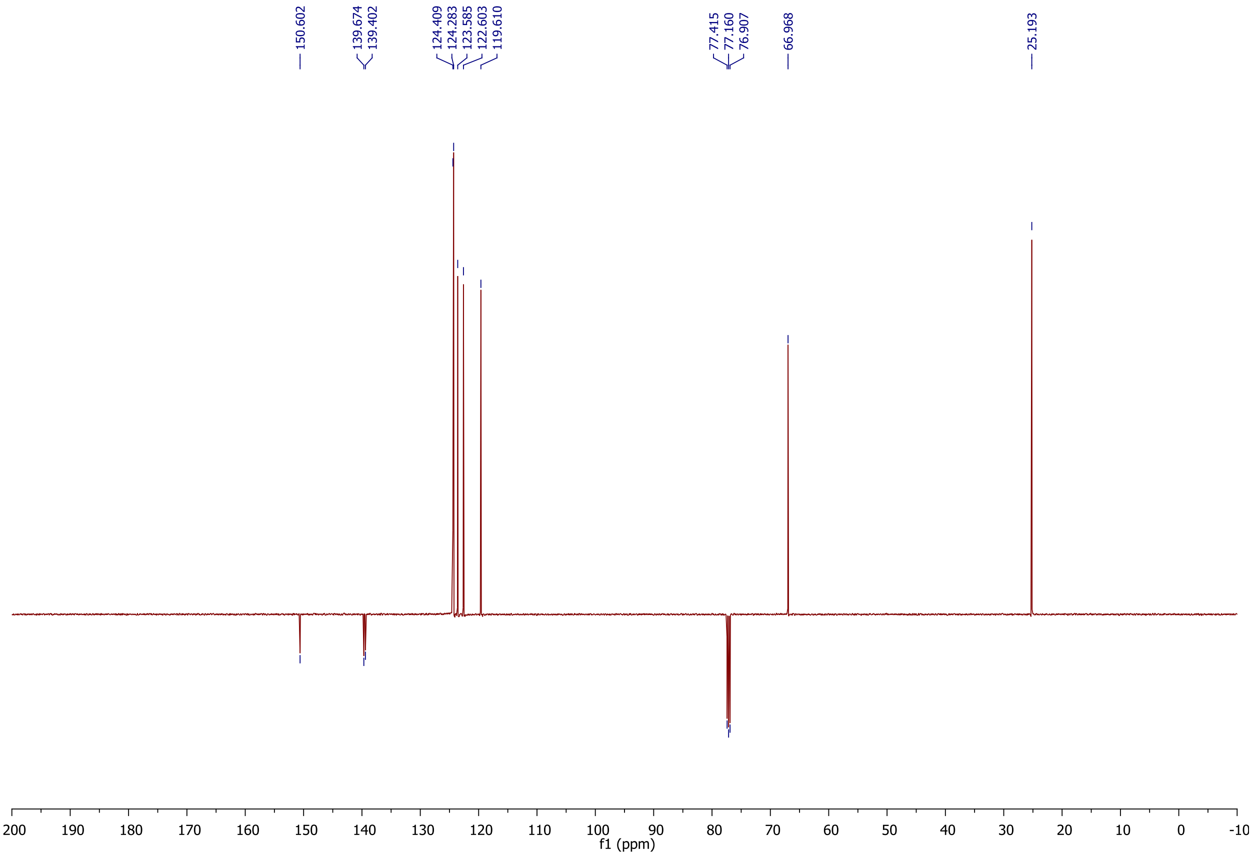

1-(Benzofuran-2-yl)ethan-1-ol

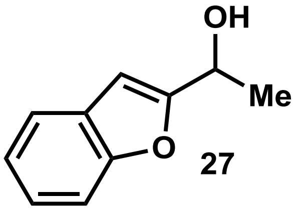

27

The title compound 1-(benzofuran-2-yl)ethan-1-ol **27** was prepared according to general procedure 4 from ((1-(benzofuran-2-yl)vinyl)oxy)(*tert*-butyl)dimethylsilane (137 mg, 0.50 mmol, 1 equiv), tris(pentafluorophenyl)borane (25.6 mg, 0.05 mmol, 10 mol %), 2,2,6,6-tetramethylpiperidine (8.4 μL, 0.05 mmol, 10 mol %) and γ-terpinene (104 μL, 0.65 mmol, 1.3 equiv), followed by TBAF (0.50 mmol, 0.5 mL, 1 equiv) and purified by flash silica column chromatography (eluent = 10% EtOAc in hexanes) to give a yellow oil (72 mg, 89% yield). R_f = 0.35 (eluent = 20% EtOAc in hexanes); ν_{max} / cm^{-1} (film) 3275, 2972, 1581, 1452, 1255, 1024, 821, 738; ^{1}H NMR (500 MHz, $CDCl_3$) δ_H: 1.64 (3H, t, *J* 6.6), 2.17 (1H, br s), 5.02 (1H, q, *J* 6.6), 6.61 (1H, s), 7.20-7.24 (1H, m), 7.26-7.29 (1H, m), 7.46 (1H, d, *J* 8.1), 7.53-7.55 (1H, m); ^{13}C{^{1}H} NMR (126 MHz, $CDCl_3$) δ_C: 21.5, 64.3, 101.9, 111.3, 121.2, 122.9, 124.3, 128.3,

154.9, 160.3; HRMS (ASAP$^+$) calculated for $[C_{10}H_9O_2]^+$ (M–H)$^+$: m/z 161.0603, found 161.0603 (0.0 ppm).

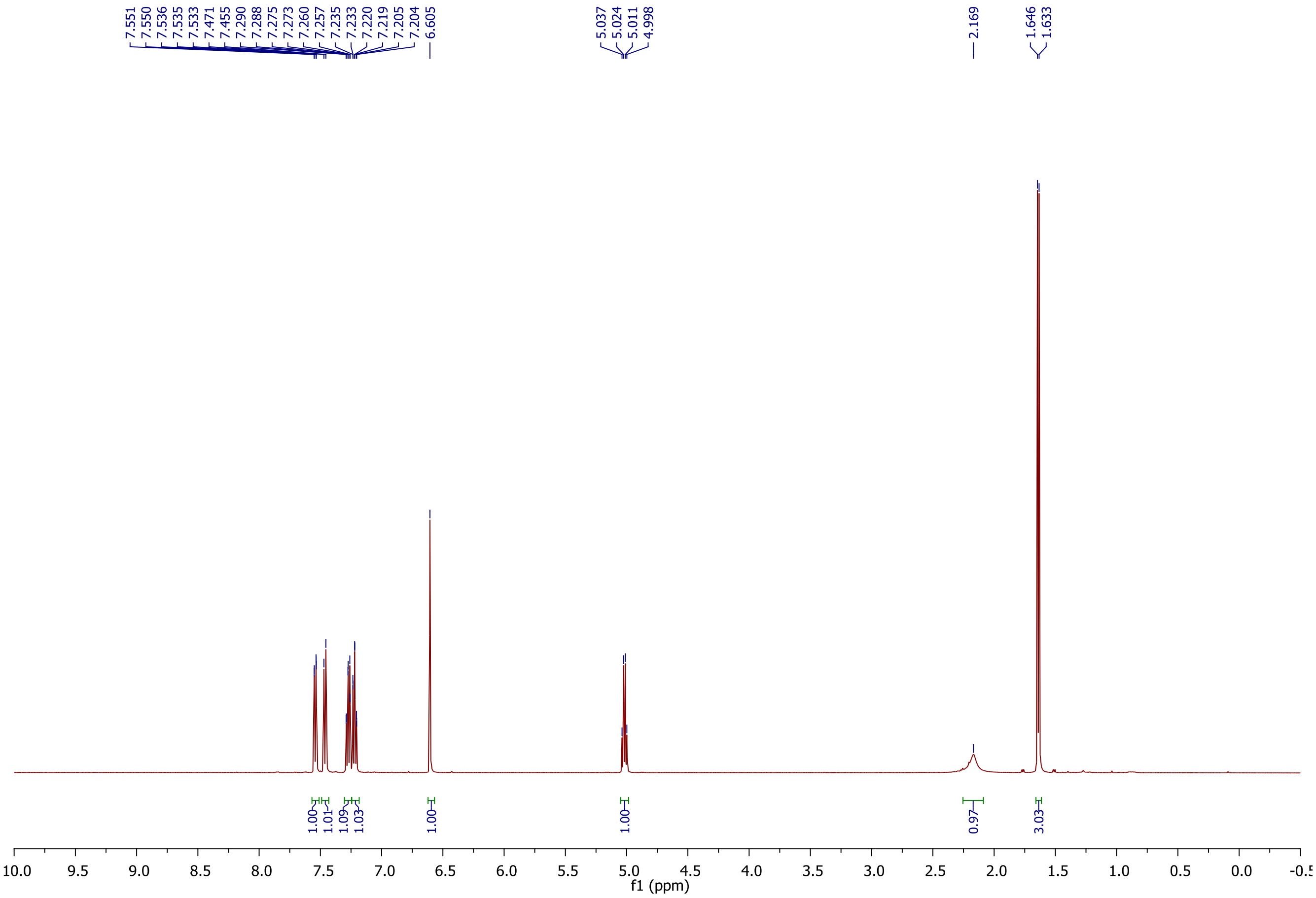

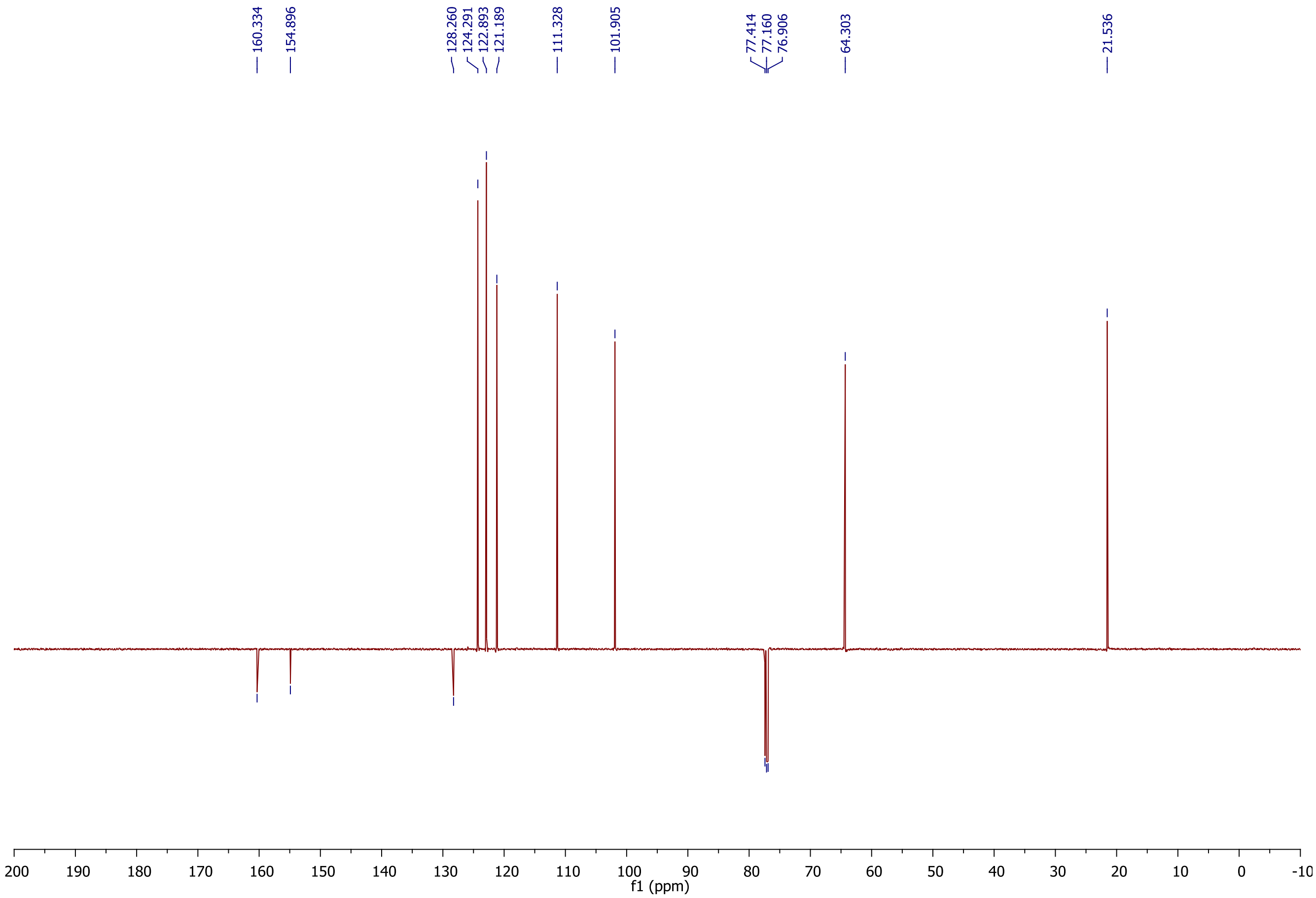

2,3-Dihydro-1*H*-inden-1-ol

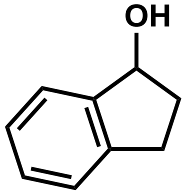

28

The title compound 2,3-dihydro-1*H*-inden-1-ol **28** was prepared according to general procedure 4 from ((1*H*-inden-3-yl)oxy)(*tert*-butyl)dimethylsilane (123 mg, 0.50 mmol, 1 equiv), tris(pentafluorophenyl)borane (25.6 mg, 0.05 mmol, 10 mol %), 2,2,6,6-tetramethylpiperidine (8.4 μL, 0.05 mmol, 10 mol %) and γ-terpinene (104 μL, 0.65 mmol, 1.3 equiv), followed by TBAF (0.50 mmol, 0.5 mL, 1 equiv) and purified by flash silica column chromatography (eluent = 10% EtOAc in hexanes) to give a light yellow oil (56 mg, 84% yield). R_f = 0.30 (eluent = 20% EtOAc in hexanes); ν_{max} / cm^{-1} (film) 3309, 2980, 1606, 1477, 1153, 1049, 740; ^{1}H NMR (500 MHz, CD_3OD) δ_H: 1.87-1.94 (1H, m), 2.38-2.44 (1H, m), 2.75-2.81 (1H, m), 2.99-3.05 (1H, m), 4.87 (1H, br s), 5.16 (1H, t, *J* 6.2), 7.17-7.22 (3H, m), 7.36-7.38 (1H, m);

$^{13}C\{^{1}H\}$ NMR (126 MHz, CD_3OD) δ_C: 30.6, 36.4, 76.8, 125.3, 125.6, 127.5, 129.0, 144.4, 146.4; HRMS (ASAP$^+$) calculated for $[C_9H_9O]^+$ $(M–H)^+$: m/z 133.0653, found 133.0653 (0.0 ppm).

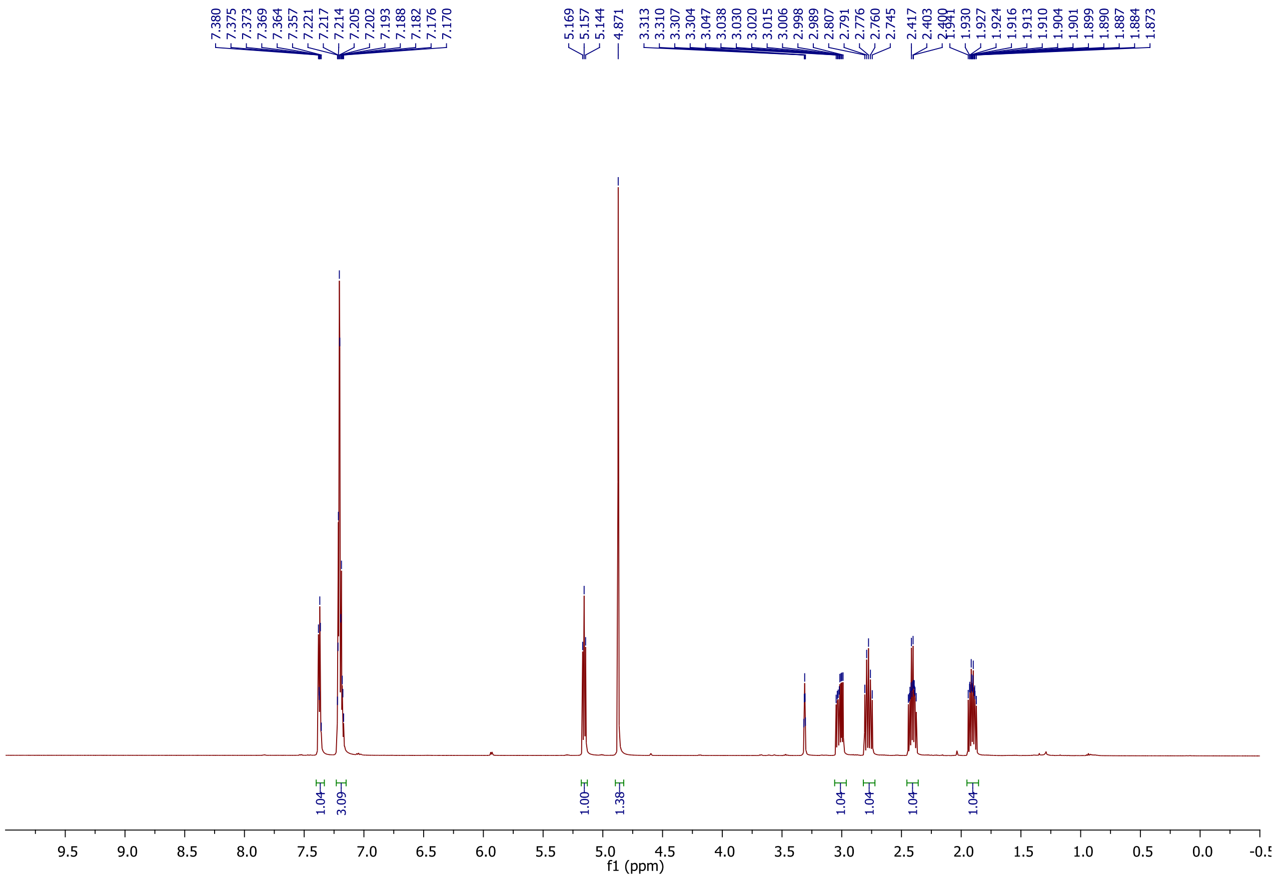

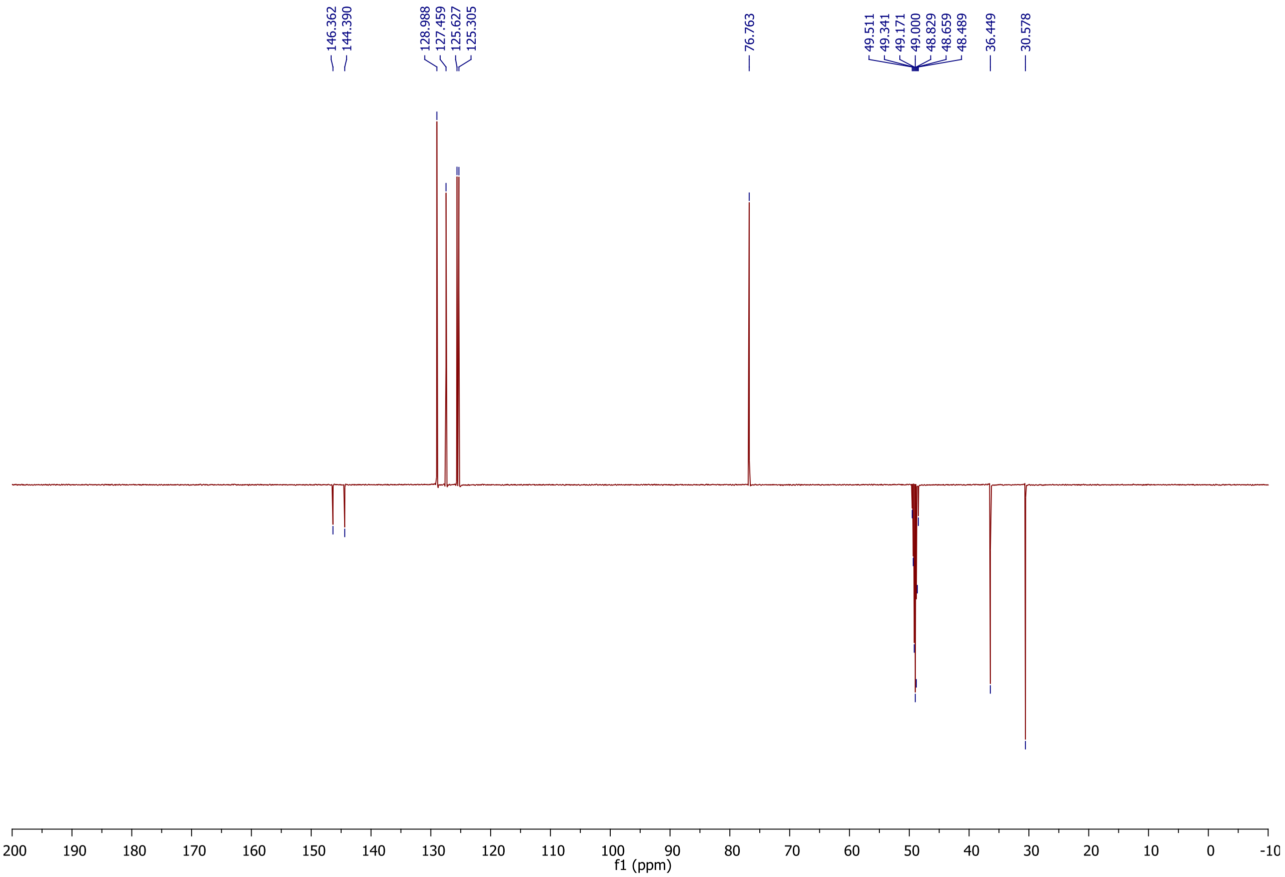

Cyclohexanol

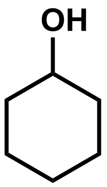

29

The title compound cyclohexanol **29** was prepared according to general procedure 3 from (cyclohex-1-en-1-yloxy)trimethylsilane (85 mg, 0.50 mmol, 1 equiv), tris(pentafluorophenyl)borane (25.6 mg, 0.05 mmol, 10 mol %), 2,2,6,6-tetramethylpiperidine (8.4 μL, 0.05 mmol, 10 mol %) and γ-terpinene (104 μL, 0.65 mmol, 1.3 equiv), followed by TBAF (0.50 mmol, 0.5 mL, 1 equiv) and purified by flash silica column chromatography (eluent = 10% EtOAc in hexanes) to give a colorless oil (35 mg, 70% yield). R_f = 0.23 (eluent = 20% EtOAc in hexanes); ν_{max} / cm^{-1} (film) 3309, 2927, 2852, 1450, 1361, 1064, 968, 889, 555; ^{1}H NMR (500 MHz, $CDCl_3$) δ_H: 1.11-1.31 (5H, m), 1.50-1.55 (1H, m), 1.68-1.75 (3H, m), 1.85-1.89 (2H, m), 3.56-3.61 (1H, m); ^{13}C{^{1}H} NMR (126 MHz, $CDCl_3$) δ_c: 24.3, 25.6, 35.6,

70.4; HRMS (CI$^+$) calculated for $[C_6H_{16}ON]^+$ $(M+NH_4)^+$: m/z 118.1226, found 118.1226 (0.0 ppm).

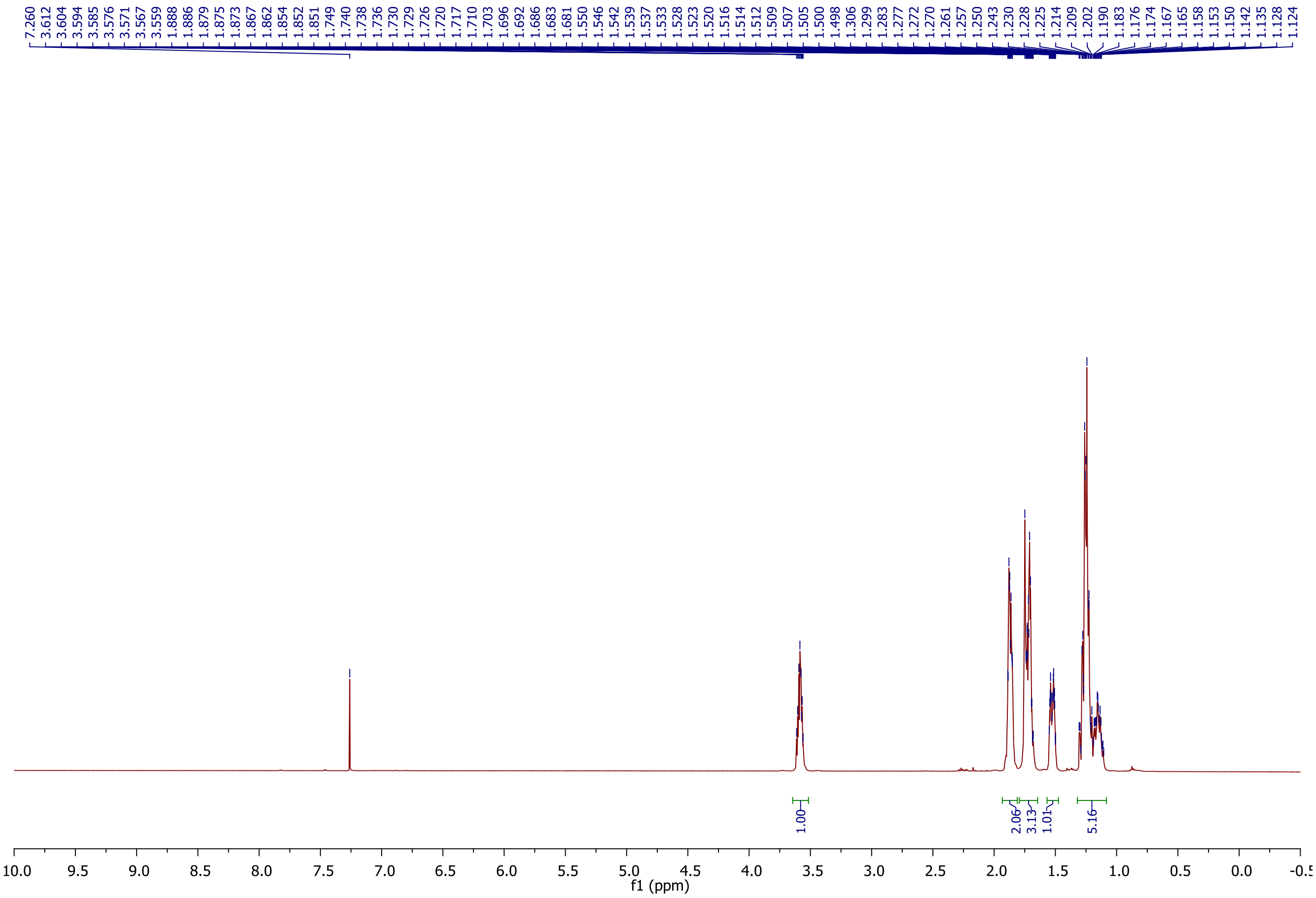

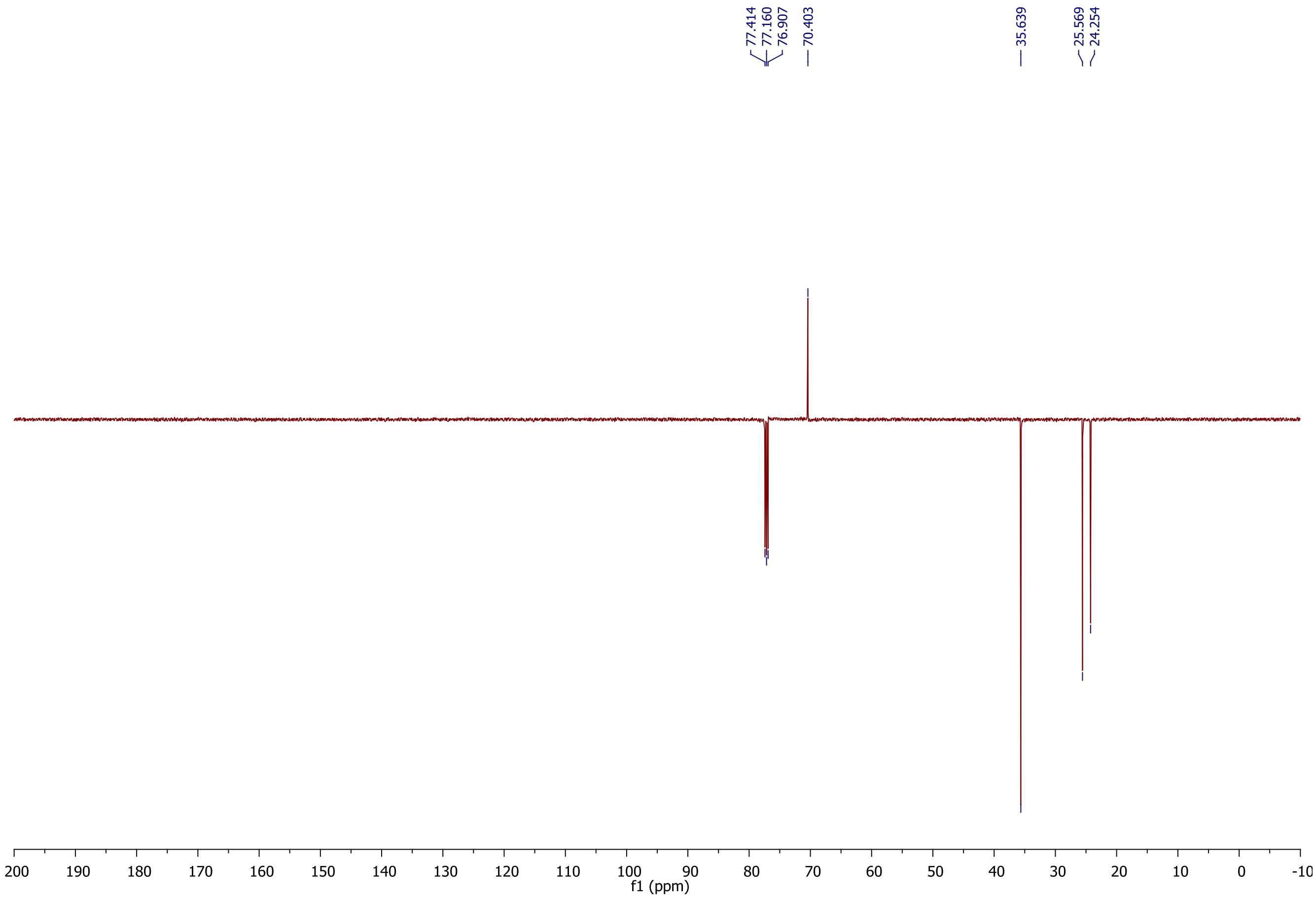

3,3-Dimethylbutan-2-ol

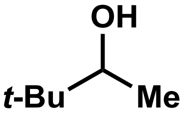

30

The title compound 3,3-dimethylbutan-2-ol **30** was prepared according to general procedure 3 from ((3,3-dimethylbut-1-en-2-yl)oxy)trimethylsilane (86 mg, 0.50 mmol, 1 equiv), tris(pentafluorophenyl)borane (25.6 mg, 0.05 mmol, 10 mol %), 2,2,6,6-tetramethylpiperidine (8.4 µL, 0.05 mmol, 10 mol %) and γ-terpinene (104 µL, 0.65 mmol, 1.3 equiv), followed by TBAF (0.50 mmol, 0.5 mL, 1 equiv). Due to high volatility of the product, NMR yield of the crude reaction mixture with 1,3,5-trimethylbenzene (70 µL, 0.50 mmol, 1 equiv) as the internal standard is reported. Yield = >98%. ^{1}H NMR (500 MHz, $CDCl_3$) δ_H: 3.42-3.46 (1H, m).

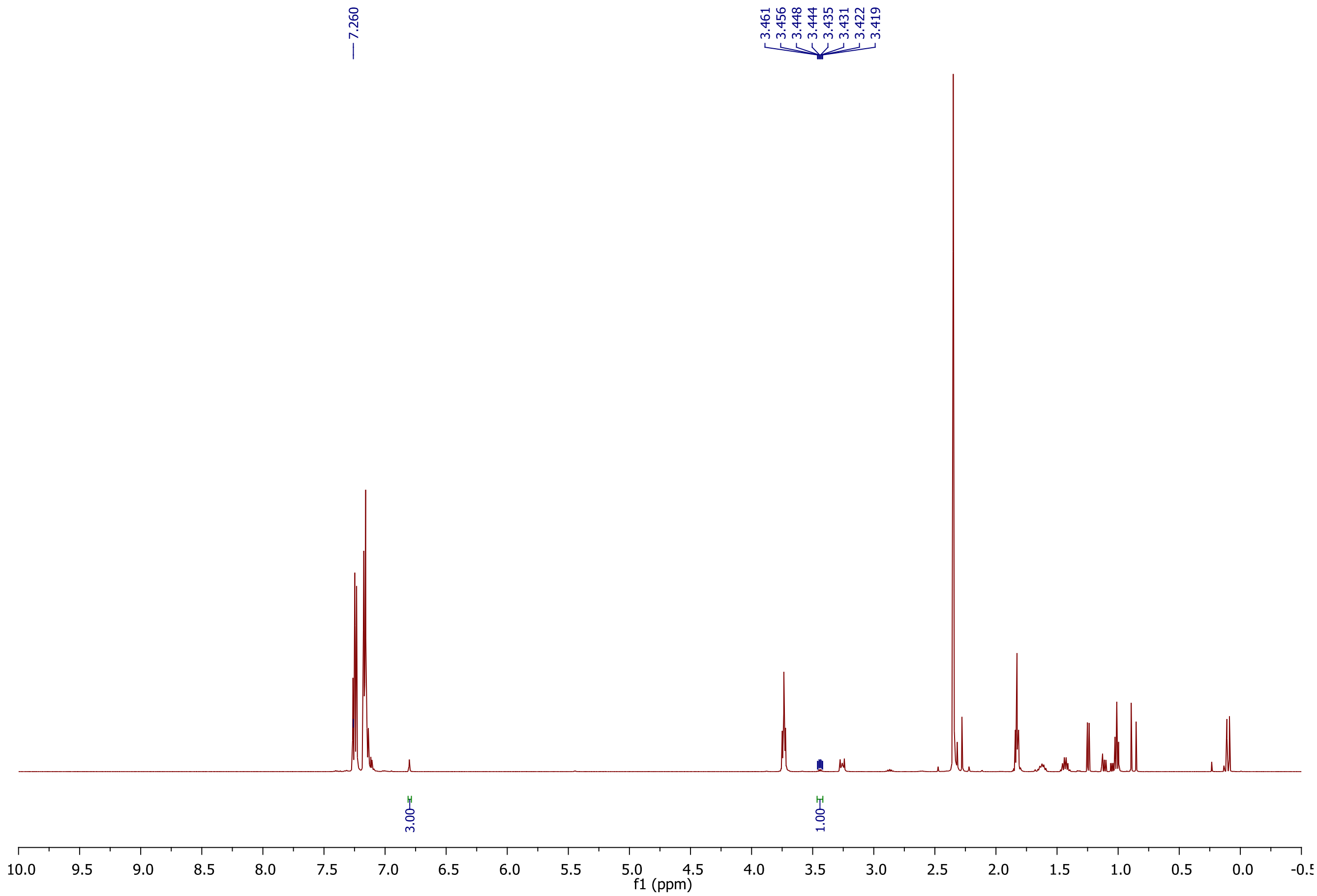

1.2.5. FLP-catalyzed transfer hydrogenation of enamines

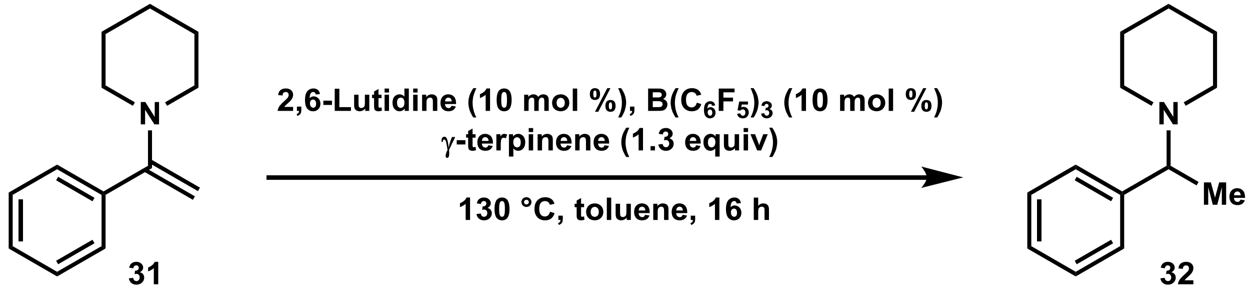

In a nitrogen-filled glove box, to an appropriate reaction vessel containing a magnetic follower was charged the 1-(1-phenylvinyl)piperidine (94 mg, 0.50 mmol, 1 equiv), tris(pentafluorophenyl)borane (25.6 mg, 0.05 mmol, 10 mol %), 2,6-lutidine (5.82 µL, 0.05 mmol, 10 mol %) and γ-terpinene (104 µL, 0.65 mmol, 1.3 equiv) in toluene (3 mL). The reaction mixture was heated to 130 °C for 16 h. The reaction resulted in 23% conversion to reduced product **32**. ^{1}H NMR (500 MHz, $CDCl_3$) δ_H: 3.49 (1H, q, *J* 6.8).

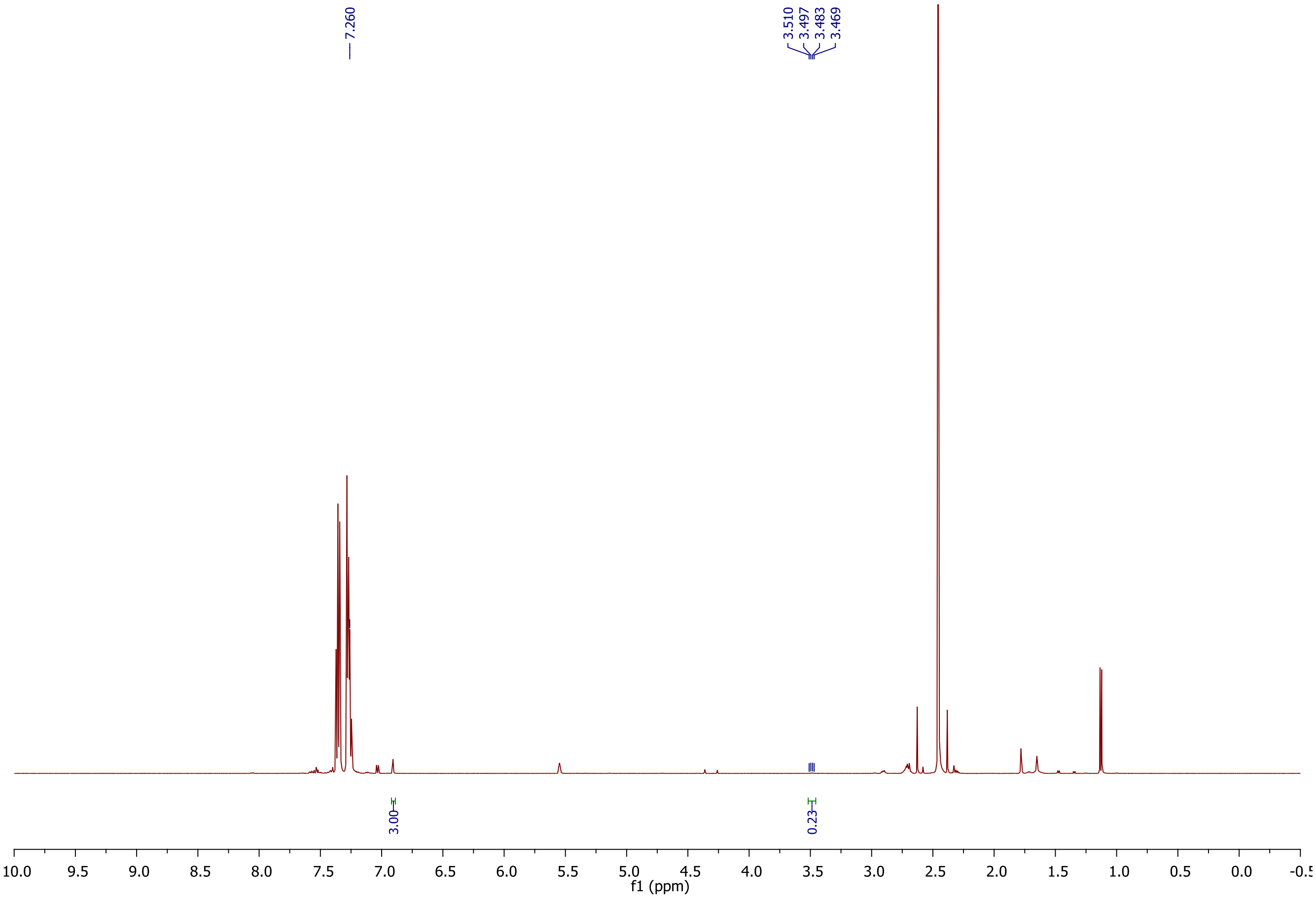

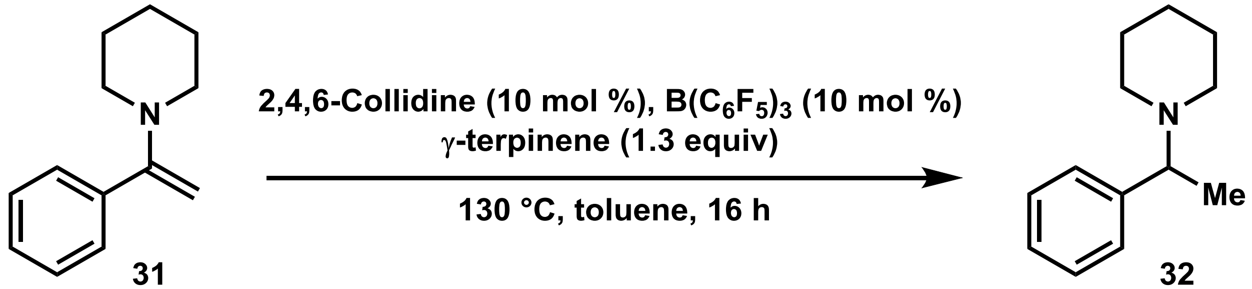

In a nitrogen-filled glove box, to an appropriate reaction vessel containing a magnetic follower was charged the 1-(1-phenylvinyl)piperidine (94 mg, 0.50 mmol, 1 equiv), tris(pentafluorophenyl)borane (25.6 mg, 0.05 mmol, 10 mol %), 2,4,6-collidine (6.61 µL, 0.05 mmol, 10 mol %) and γ-terpinene (104 µL, 0.65 mmol, 1.3 equiv) in toluene (3 mL). The reaction mixture was heated to 130 °C for 16 h. The reaction resulted in 23% conversion to reduced product **32**. ^{1}H NMR (500 MHz, $CDCl_3$) δ_H: 3.45 (1H, q, *J* 6.8).

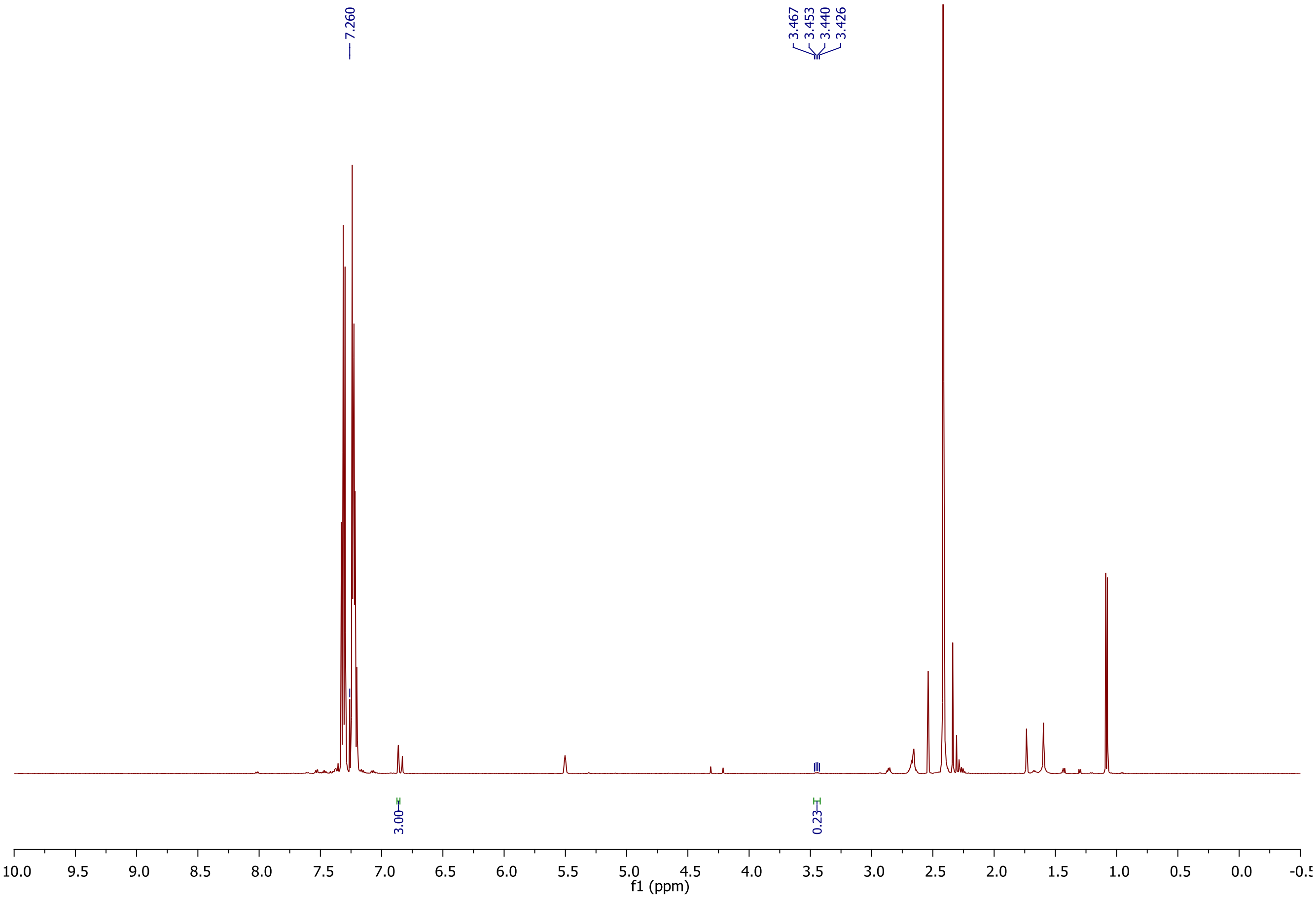

7.260
3.467
3.453
3.440
3.426
3.00
0.23
10.0
9.5
9.0
8.5
8.0
7.5
7.0
6.5
6.0
5.5
5.0
4.5
4.0
3.5
3.0
2.5
2.0
1.5
1.0
0.5
0.0
-0.5
f1 (ppm)

References

1. I. Khan, M. Manzotti, G. J. Tizzard, S. J. Coles, R. L. Melen, L. C. Morrill, *ACS Catal*. **2017**, *7*, 7748-7752.
2. S. Xie, S. A. Lopez, O. Ramström, M. Yan, K. N. Houk, *J. Am. Chem. Soc*. **2015**, *137*, 2958-2966.
3. G. Stork, A. Brizzolara, H. Landesman, J. Szmuszkovicz, R. Terrell, *J. Am. Chem. Soc*. **1963**, *85*, 207-222.
4. J.-M. Lin, B.-S. Liu, *Synth. Commun*. **1997**, *27*, 739-749.
5. B. Hurlocker, M. R. Miner, K. A. Woerpel, *Org. Lett*. **2014**, *16*, 4280-4283.
6. J.-F. Zhao, B.-H. Tan, T.-P. Loh, *Chem. Sci*. **2011**, *2*, 349-352.
7. D. L. Ventura, Z. Li, M. G. Coleman, H. M. L. Davies, *Tetrahedron*, **2009**, *65*, 3052-3061.
8. Y. Hiraiwa, K. Ishihara, H. Yamamoto, *Eur. J. Org. Chem*. **2006**, *8*, 1837-1844.
9. T. Nagata, H. Matsubara, K. Kiyokawa, S. Minakata, *Org. Lett*. **2017**, *19*, 4672-4675.
10. N. Kamigata, K. Udodaira, M. Yoshikawa, T. Shimizu, *J. Organomet. Chem*. **1998**, *552*, 39-43.
11. B. G. Lenz, H. Regeling, H. L. M. Van Rozendaal, B. Zwanenburg, *J. Org. Chem*. **1985**, *50*, 2930-2934.
12. S. Pramanik, S. Rej, S. Kando, H. Tsurugi, K. Mashima, *J. Org. Chem*. **2018**, *83*, 2409-2417.
13. M. Pouliot, P. Renaud, A. Studer, T. Vogler, K. Schenk, *Angew. Chem., Int. Ed*. **2009**, *48*, 6037-6040.
14. C. K. Lee, I.-S. H. Lee, W. Noland, *Heterocycles*, **2007**, *71*, 419-428.
15. M. A. Tandiary, M. Asano, T. Hattori, S. Takehira, Y. Masui, M. Onaka, *Tetrahedron Lett*. **2017**, *58*, 1925-1928.
16. P. Cazeau, F. Duboudin, F. Moulines, O. Babot, J. Dunogues, *Tetrahedron*, **1987**, *43*, 2075-2088.
17. B. A. Sparling, D. C. Moebius, M. D. Shair, *J. Am. Chem. Soc*. **2013**, *135*, 644-647.
